# Thermal stability of *N*-heterocycle-stabilized iodanes – a systematic investigation

**DOI:** 10.3762/bjoc.15.223

**Published:** 2019-09-27

**Authors:** Andreas Boelke, Yulia A Vlasenko, Mekhman S Yusubov, Boris J Nachtsheim, Pavel S Postnikov

**Affiliations:** 1 Institute for Organic and Analytical Chemistry, University of Bremen, 28359 Bremen, Germany; 2 Research School of Chemistry and Applied Biomedical Sciences, Tomsk Polytechnic University, 634050 Tomsk, Russian Federation; 3 Department of Solid State Engineering, University of Chemistry and Technology, 16628 Prague, Czech Republic

**Keywords:** differential scanning calorimetry, hypervalent iodine, *N*-heterocycle, stability, thermogravimetry

## Abstract

The thermal stability of pseudocyclic and cyclic *N*-heterocycle-stabilized (hydroxy)aryl- and mesityl(aryl)-λ^3^-iodanes (NHIs) through thermogravimetric analysis (TGA) and differential scanning calorimetry (DSC) is investigated. Peak decomposition temperatures (*T*_peak_) were observed within a wide range between 120 and 270 °C. Decomposition enthalpies (Δ*H*_dec_) varied from −29.81 to 141.13 kJ/mol. A direct comparison between pseudocyclic and cyclic NHIs revealed high *T*_peak_ but also higher Δ*H*_dec_ values for the latter ones. NHIs bearing *N*-heterocycles with a high N/C-ratio such as triazoles show among the lowest *T*_peak_ and the highest Δ*H*_dec_ values. A comparison of NHIs with known (pseudo)cyclic benziodoxolones is made and we further correlated their thermal stability with reactivity in a model oxygenation.

## Introduction

**Figure 1:**
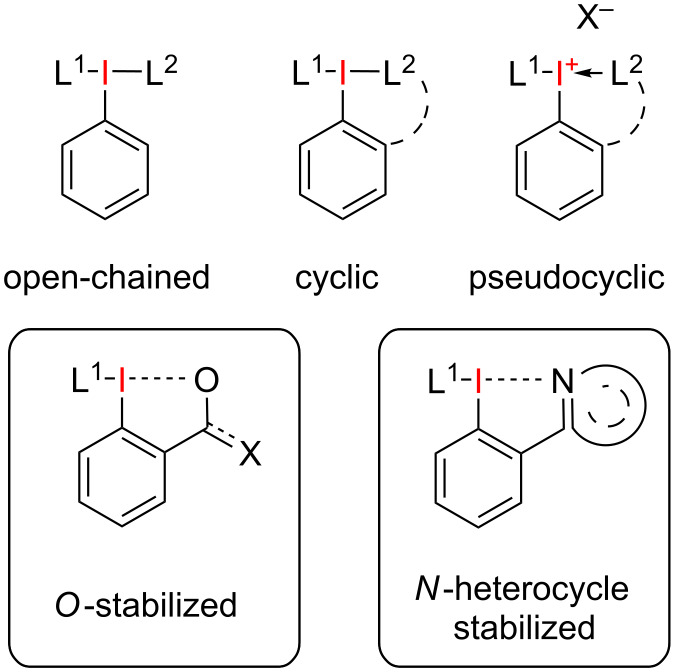
General structure of aryl-λ^3^-iodanes.

Hypervalent iodine compounds, in particular aryl-λ^3^-iodanes, have found wide spread applications as oxidants and electrophilic group transfer reagents in organic synthesis [1–11]. A 3-center 4-electron bond connects the central iodine atom, providing two electrons, with two carbon- or heteroatom ligands L^1^ and L^2^, providing one electron each (Figure 1). These ligands can be arranged along the hypervalent iodine atom through an open-chained, a pseudocyclic or a cyclic structure.

One of the ligands gets either substituted during an iodane-mediated transformation or is directly transferred in an electrophilic group-transfer reaction onto a nucleophilic substrate. The other ligand stabilizes the electrophilic hypervalent iodine atom in its ground state or directly influences its reactivity by stabilizing reactive intermediates or transition states. In recent years, a plethora of cyclic and pseudocyclic iodanes have been developed with covalently attached stabilizing ligands L^2^ and applied in a variety of group-transfer reactions. Prominent examples include ethynyl benziodoxolones (EBX, L^1^ = alkyne) [12], the Zhdankin reagent azidobenziodoxole (ABX, L^1^ = N_3_) [13], cyanobenziodoxole (CBX, L^1^ = CN) [14] or Togni’s reagent (L^1^ = CF_3_) [15]. Even though the transferable ligand (L^1^) has been varied extensively, the chemical design of the stabilizing donor ligand L^2^ has not been investigated as intensive. In general, carboxylic acid derivatives, ethers or free alcohols are utilized to stabilize the hypervalent iodine center by an oxygen–iodine bond or through dative oxygen–iodine interactions in (pseudo)cyclic iodanes. Albeit aryl-λ^3^-iodanes are viewed as safe and stable under ambient temperatures, systematic thermal degradation studies of hypervalent iodine reagents are still rare. In 1992 Varvoglis and co-workers investigated the thermal degradation of a variety of aryl iodine(III) dicarboxylates into alkyl and aryl radicals through thermogravimetry [16–17]. In 2013 Kumar and co-workers compared the thermal properties of open-chained aryl-λ^3^-iodanes with their polymer bound derivatives and found an endothermic decomposition behavior [18]. In the same year Haller and co-workers investigated the explosive properties of Togni’s reagent and very recently, Williams and co-workers analyzed the sensitivity of common oxidants including 2-iodoxybenzoic acid (IBX) and Dess–Martin periodinane (DMP) [19–20]. Waser and co-workers examined the thermal stability of the Zhdankin reagent ABX and compared it with the amide-stabilized derivative ABZ (azidobenziodazolone). They found a remarkable higher thermal stability of the latter compound by DSC analysis indicated by a higher onset temperature and a lower heat release during decomposition [21]. ABZ is a rare example of a nitrogen-stabilized iodane, showing promising properties in terms of reactivity and stability. However, iodanes stabilized by nitrogen-based ligands, in particular *N*-heterocycles are still underexplored [22–28]. Recently, our groups investigated systematically cyclic and pseudocyclic *N*-heterocycle-stabilized iodanes (NHIs). We demonstrated that the *N*-heterocycle significantly influences the important I–L^1^ bond length and subsequently has a profound impact on the reactivity of the iodane in oxygen transfer reactions [29–30]. Since the combination of a highly oxidized hypervalent iodine species with *N*-heterocycles with a high N/C-ratio might result in potential hazardous high energy materials, we herein investigated their thermal stability by thermogravimetric analysis (TGA) and differential scanning calorimetry (DSC).

## Results and Discussion

**Figure 2:**
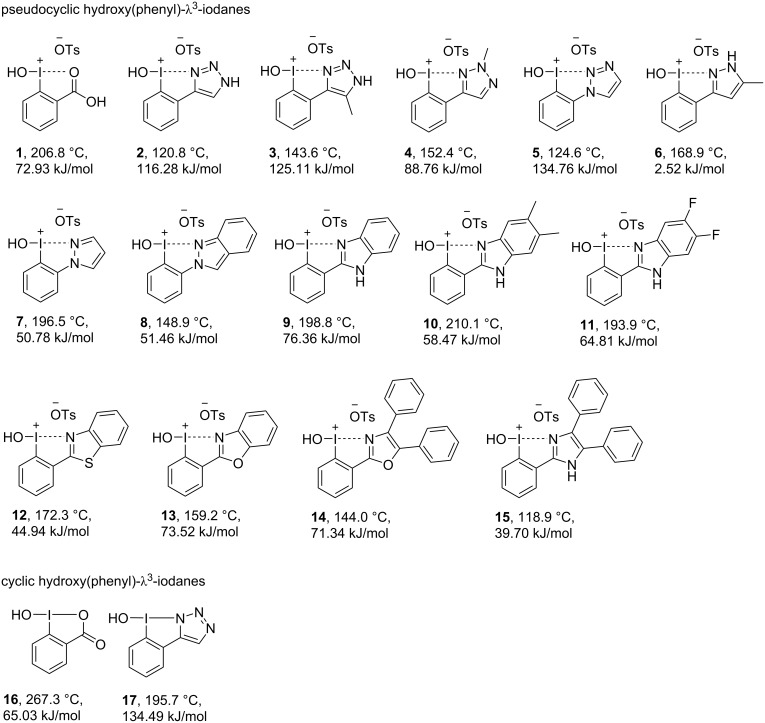
*T*_peak_ and Δ*H*_dec_-values for a range of N- and O-substituted iodanes.

We started our experiments with the preparation of appropriate λ^3^-iodanes bearing N- and O-centered ligands according to our reported procedures [29–30]. The synthesized compounds then have been divided in the following discussion by their principle structural features into pseudocyclic and cyclic hydroxy(phenyl)-λ^3^-iodanes (Figure 2) and pseudocyclic and cyclic mesityl(phenyl)-λ^3^-iodanes (Figure 6).

**Figure 3:**
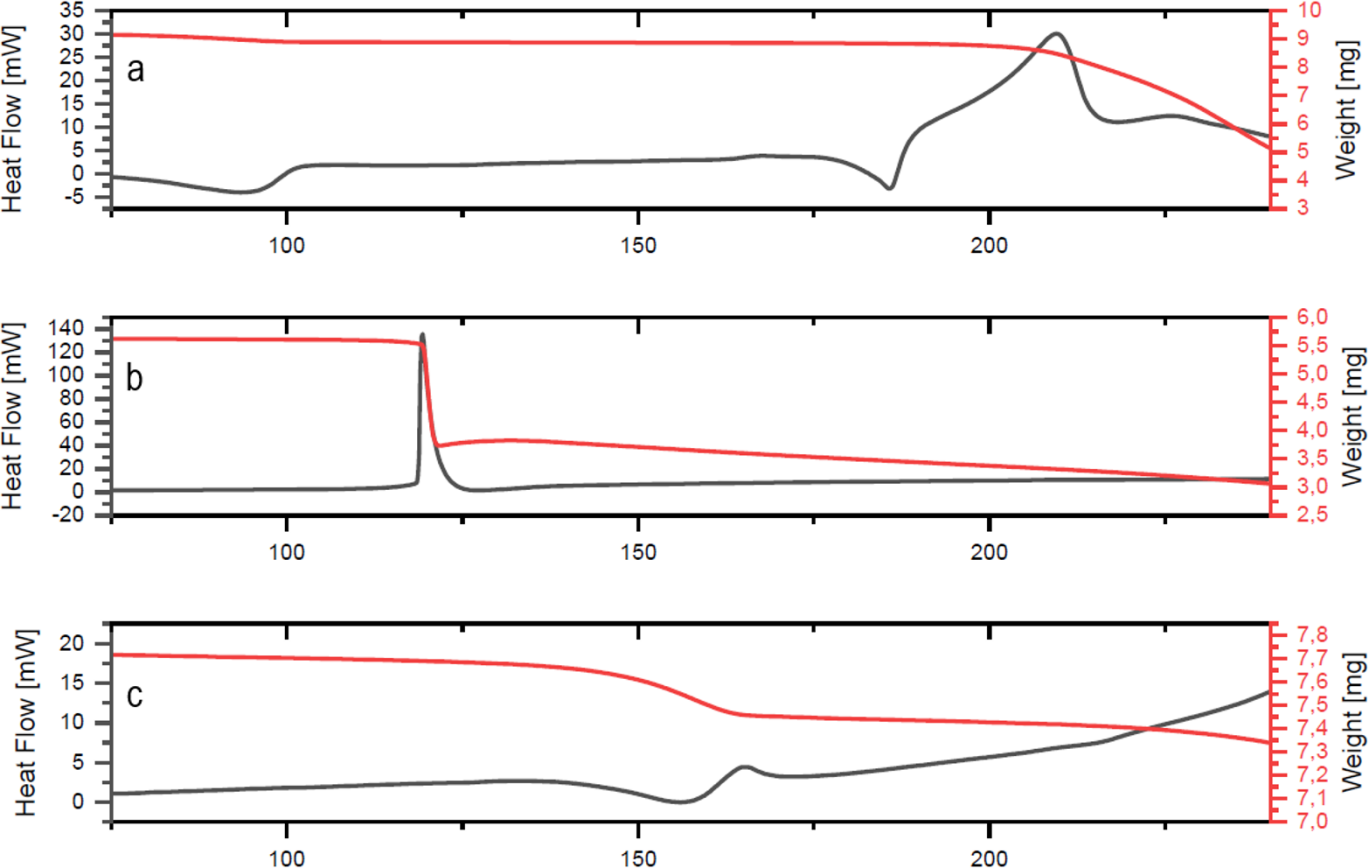
TGA/DSC curves of (a) benziodoxolone **1**, (b) triazole **2** and (c) pyrazole **6**.

We initially investigated the thermal decomposition of *N*-heterocycle-stabilized pseudocyclic (hydroxy)aryl iodanes **2**–**15** (Figure 2 and Table S1, Supporting Information File 1). As the model oxygen-stabilized derivative, we evaluated the thermal decomposition behavior of pseudocyclic iodosobenziodoxolone **1**. The decomposition of **1** has a two-step character and includes initial endothermic melting at 185.1 °C followed by exothermal decomposition at 206.8 °C with an Δ*H*_dec_ of 72.9 kJ/mol (Figure 3a). For the pseudocyclic NHI **2** with a triazole ligand no initial melting process was detected. Instead the solid degraded with a pronounced and narrow (less than 1 °C) exothermal peak at 120.8 °C (Figure 3b). Decomposition was associated with a higher Δ*H*_dec_ of 116.3 kJ/mol.

A similar decomposition behavior was observed for the other triazole-containing pseudocyclic NHIs **3**–**5**. Introduction of a methyl-substituent at C5 of the triazole **3** was accompanied by an increased decomposition enthalpy (Δ*H*_dec_ = 125.1 kJ/mol). However, thermal stability as indicated by a higher *T*_peak_, significantly increased. Methyl substitution at N2 resulted in an even higher *T*_peak_ at 152.4 °C and a decreased decomposition enthalpy. If the triazole is connected to the iodoarene via N1 as in **5**, *T*_peak_ decreases and Δ*H*_dec_ increases. It should be concluded that triazole **4** has the most advantageous decomposition behavior: It is thermally the most stable among the pseudocyclic triazoles with the lowest Δ*H*_dec_ value. However, even the triazoles **2**, **3**, and **5** can be considered as safe compounds, but still deserve a common precaution due to the narrow decomposition process.

Pyrazoles **6** and **7** are thermally more stable (*T*_peak_ = 168.9 and 196.5 °C) with a remarkably lower Δ*H*_dec_ value. NH-pyrazole **6** shows the lowest Δ*H*_dec_ value among all investigated pseudocyclic iodanes (Δ*H*_dec_ = 2.5 kJ/mol). Interestingly, the exothermal decomposition of **6** is superimposed by an endothermal melting process (Figure 3c). In direct comparison, indazole **8** is thermally less stable than **7** with a similar Δ*H*_dec_ value. The throughout higher thermal stability of pyrazoles and indazoles (**6–8**) in direct comparison to triazoles (**2–5**) is very likely connected with the lower C/N ratio.

**Figure 4:**
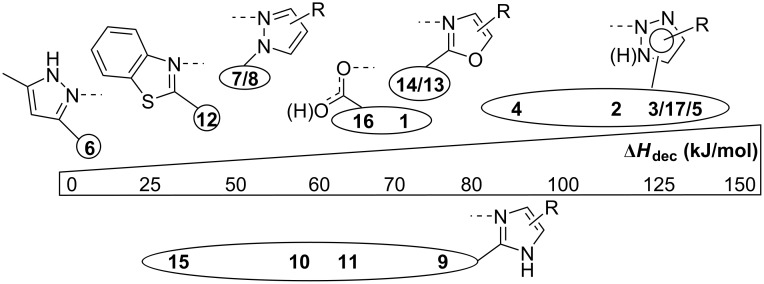
Decomposition enthalpy (Δ*H*_dec_) scale for pseudocyclic tosylates **1**–**15** and cyclic iodoso species **16** and **17**.

Benzimidazoles **9**–**11** showed increased Δ*H*_dec_ values (58.5–76.4 kJ/mol) in comparison with pyrazoles **6**–**8**. Broad decomposition peaks (up to 14 °C peak width – see Supporting Information File 1) were observed at remarkable high *T*_peak_ values (193.9–210.1 °C). Following these results, it has been intriguing to analyze the influence of the heteroatom in the heterocyclic moiety on the thermal decomposition process. The change of one nitrogen atom to sulfur as in thiazole **12** resulted in a drastic decrease of Δ*H*_dec_ to 44.9 kJ/mol. In contrast, oxazoles **13** and **14** both had a comparable Δ*H*_dec_ to **9**, however, these NHIs are thermally more labile (*T*_peak_ = 159.2 and 144.0 °C). Compared to **9**, diphenylimidazole-substituted NHI **15** exhibited a considerably lower Δ*H*_dec_ value (39.7 kJ/mol). Among the 1,3-azoles **15** is thermally the most labile one with a *T*_peak_ of 118.9 °C. For an improved overview of the discussed Δ*H*_dec_ values a graphical comparison sorted by the respective coordinating unit is given in Figure 4.

**Figure 5:**
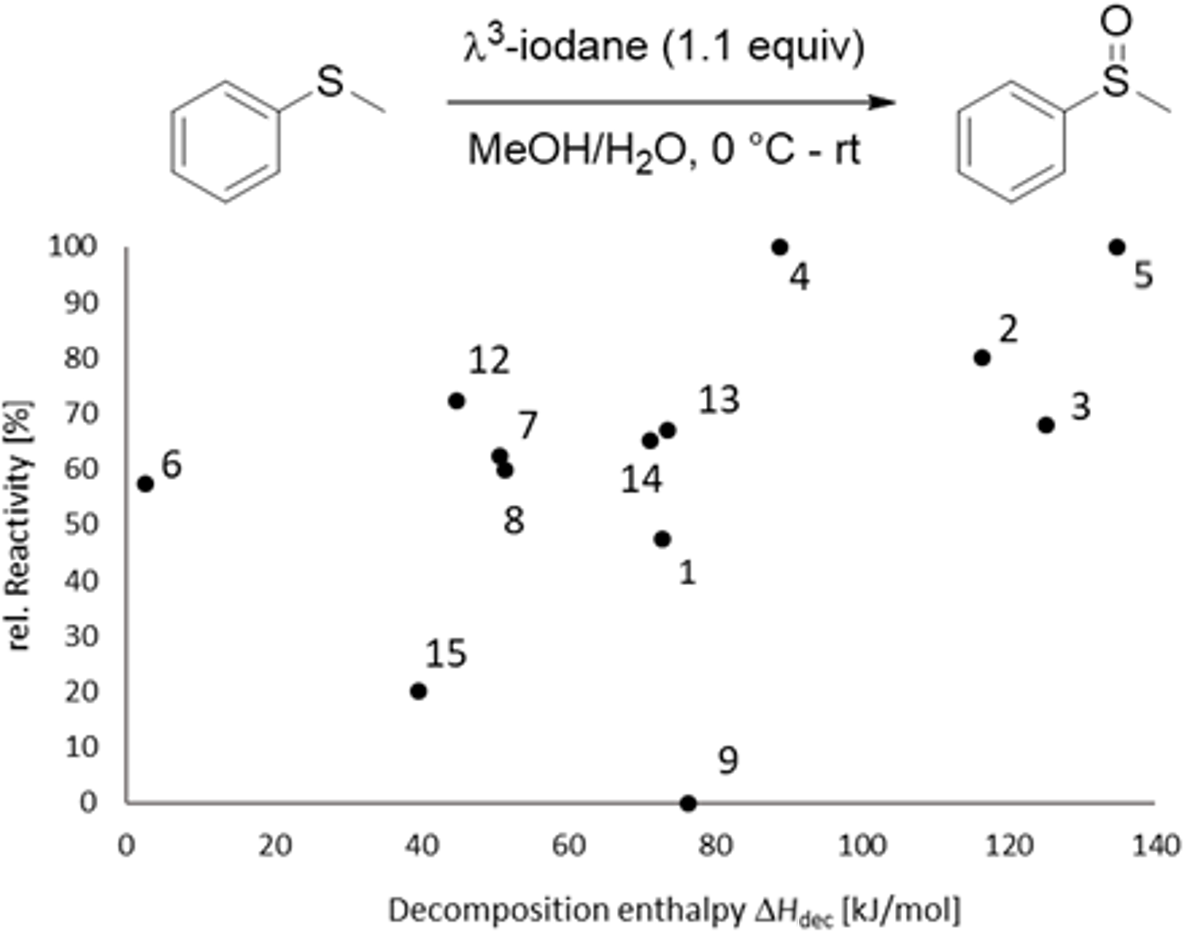
Correlation between the relative reactivity for pseudocyclic NHIs based on the reaction time in the oxidation of thioanisole with the corresponding decomposition enthalpy Δ*H*_dec_. Relative reactivity is based on the negative normalized logarithm.

After obtaining decomposition energies and temperatures for these pseudocyclic NHIs, we were intended to relate these findings with their reactivity. For this purpose, we chose the oxidation of thioanisole at room temperature as the initial model reaction. This reaction shows quantitative conversion and therefore the reactivity of the substrates can be described by the ascertained reaction times [29]. However, because the reaction times differed widely a relative reactivity (based on the negative logarithm followed by normalization) was used for a better comparison (Figure 5).

As expected, the thermally least stable triazoles **2**–**5** are also the most reactive iodanes in this model reaction, especially *N*-substituted triazoles **4** and **5**. In contrast, the reactivity of triazole **3** is comparable to that of benzoxazoles **13** and **14** as well as pyrazole **7** and indazole **8**. In our view thiazole **12** is the best compromise in this regard since it is thermally even more stable than **7** and **8** with a significant higher reactivity. However, pyrazole **6** still shows a good reactivity in this model reaction with a concurrent outstanding thermal stability. If safety issues are a major concern, for example on a very large-scale synthesis, NHIs **6** or **12** should be the first choices. Except of **15** and **9**, all diazoles are more stable and more reactive than the well-established benziodoxolone **1**. It is also worth mentioning, that even the least stable NHI **5** can be still regarded as “safe” to use [31]. Further investigations are needed to fully capture the synthetic potential of these pseudocyclic NHIs.

We also evaluated the decomposition of cyclic hypervalent iodanes. Here, iodosobenzoic acid (IBA, **16**) was chosen as an oxygen-bonded model substrate. In comparison to its pseudocyclic congener **1**, **16** demonstrated a higher *T*_peak_ (267.3 °C [19]) and a slightly lower Δ*H*_dec_ (65.0 kJ/mol vs 72.9 kJ/mol). Cyclic triazole **17** has a significantly higher *T*_peak_ but also a higher Δ*H*_dec_ compared to the corresponding pseudocycle **2** (134.5 kJ/mol vs 116.3 kJ/mol).

**Figure 6:**
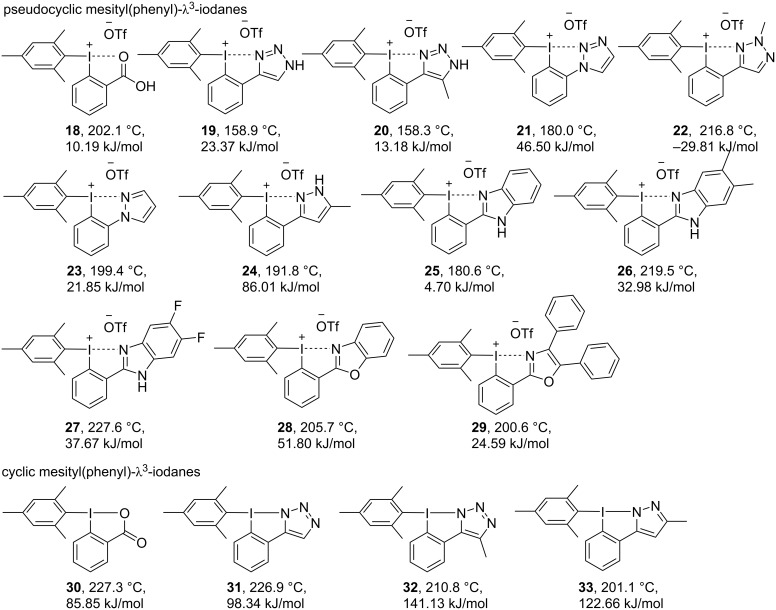
*T*_peak_ and Δ*H*_dec_ values for a range of N- and O-substituted iodanes.

Besides pseudocyclic and cyclic hydroxy(aryl)-λ^3^-iodanes, mesityl(phenyl)- λ^3^-iodanes **18**–**33** were systematically investigated by thermogravimetric analysis. Initially, the thermal decomposition of pseudocyclic diaryliodonium salts **18** and **19** was measured. For both salts, initial endothermic melting was followed by exothermal decomposition (Supporting Information File 1 and Figure 6).

**Figure 7:**
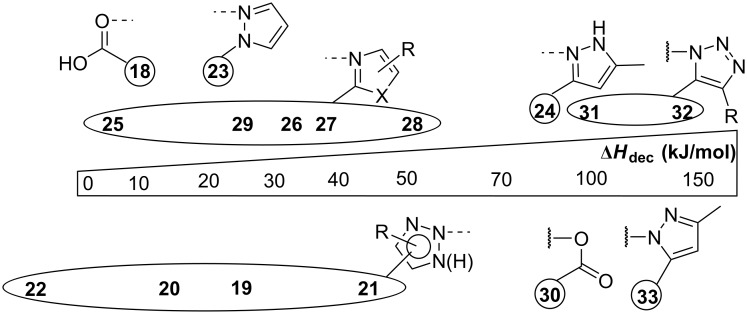
Decomposition enthalpy (Δ*H*_dec_) scale for (pseudo)cyclic mesitylen(phenyl)- λ^3^-iodanes **18**–**33**.

Compared to hydroxy(phenyl)-λ^3^-iodanes **1** and **2***,* the pseudocyclic diaryliodonium salts **18** and **19** show a significantly decreased Δ*H*_dec_ from 72.9 kJ/mol to 10.2 kJ/mol for benziodoxolones **1** and **18** and from 116.3 kJ/mol to 23.4 kJ/mol for the triazole derivatives **2** and **19**. The same trend of a higher thermal stability and lower Δ*H*_dec_ for diaryliodonium salts compared the their hydroxy-substituted congeners can be observed for all other pseudocyclic *N*-heterocycle-substituted derivatives **20–29**. Pyrazole **24** is the only exception. All investigated diaryliodonium salts can be defined as safe due to *T*_peak_ values of usually above 180 °C and Δ*H*_dec_ values of less than 50 kJ/mol. A graphical comparison of the discussed Δ*H*_dec_ values for mesityl(phenyl)- λ^3^-iodanes is given in Figure 7. In comparison with Figure 4 it clearly shows that the relative stability of these heterocycle-stabilized diaryliodonium salts do not show the same trend as observed for the initially discussed hydroxy(phenyl)-λ^3^-iodanes. In particular, the (pseudo)cyclic pyrazole derivatives **24** and **33** show a comparable high Δ*H*_dec_ value.

**Figure 8:**
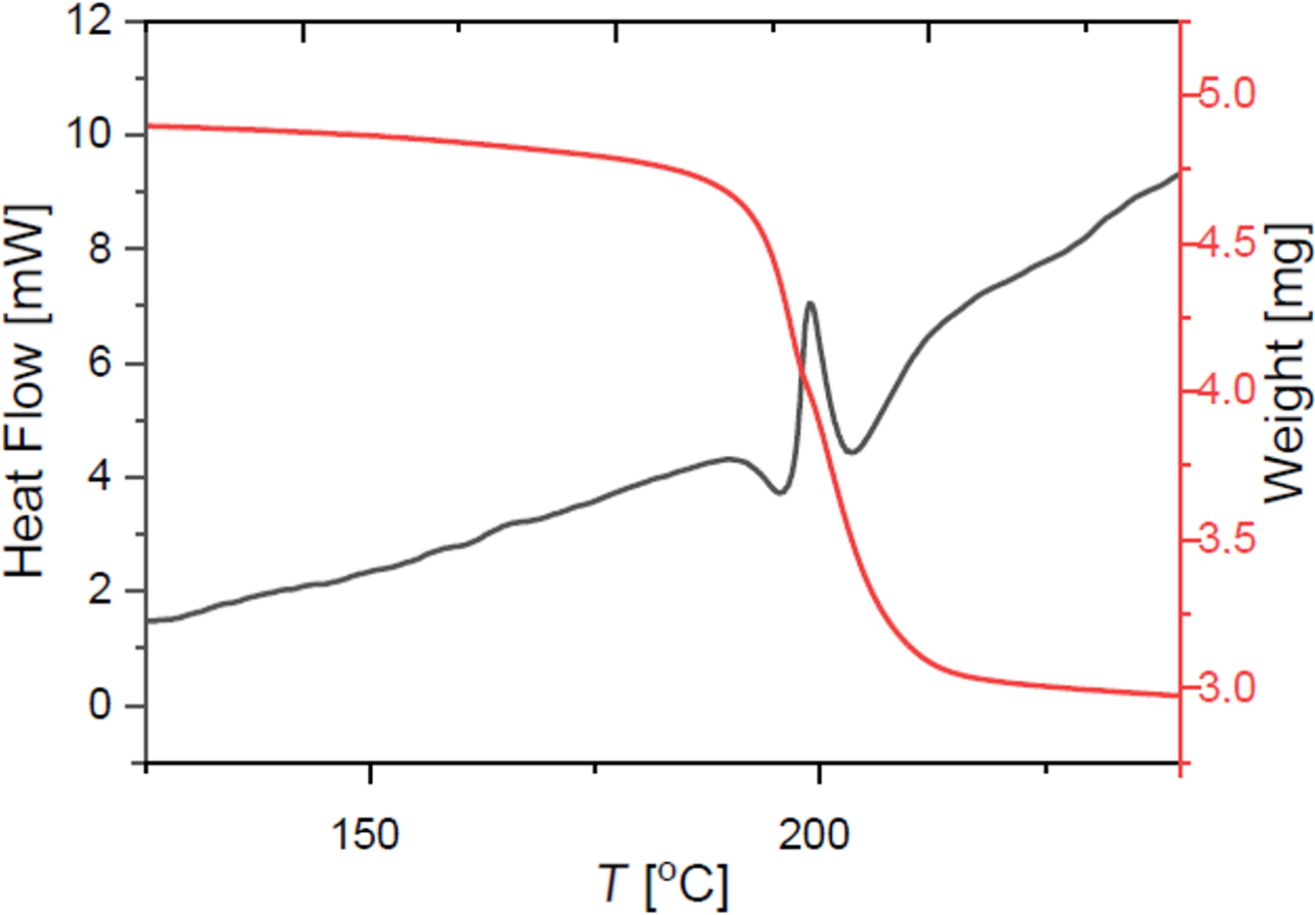
TGA/DSC curves for the benzimidazole based diaryliodonium salt **25**.

These overall significant lower decomposition energies are in good agreement with published data in the field of reactivity and stability of hypervalent iodine compounds [32]. As another common key characteristic, the exothermic decomposition of diaryliodonium salts occurs during an endothermic melting process as shown in Figure 8. Only the phenylbenzimidazoles **26** and **27** do not show this apparent melting-associated endothermic effect. Interestingly, **22** shows a very unusual endothermal decomposition of −29.81 kJ/mol.

**Figure 9:**
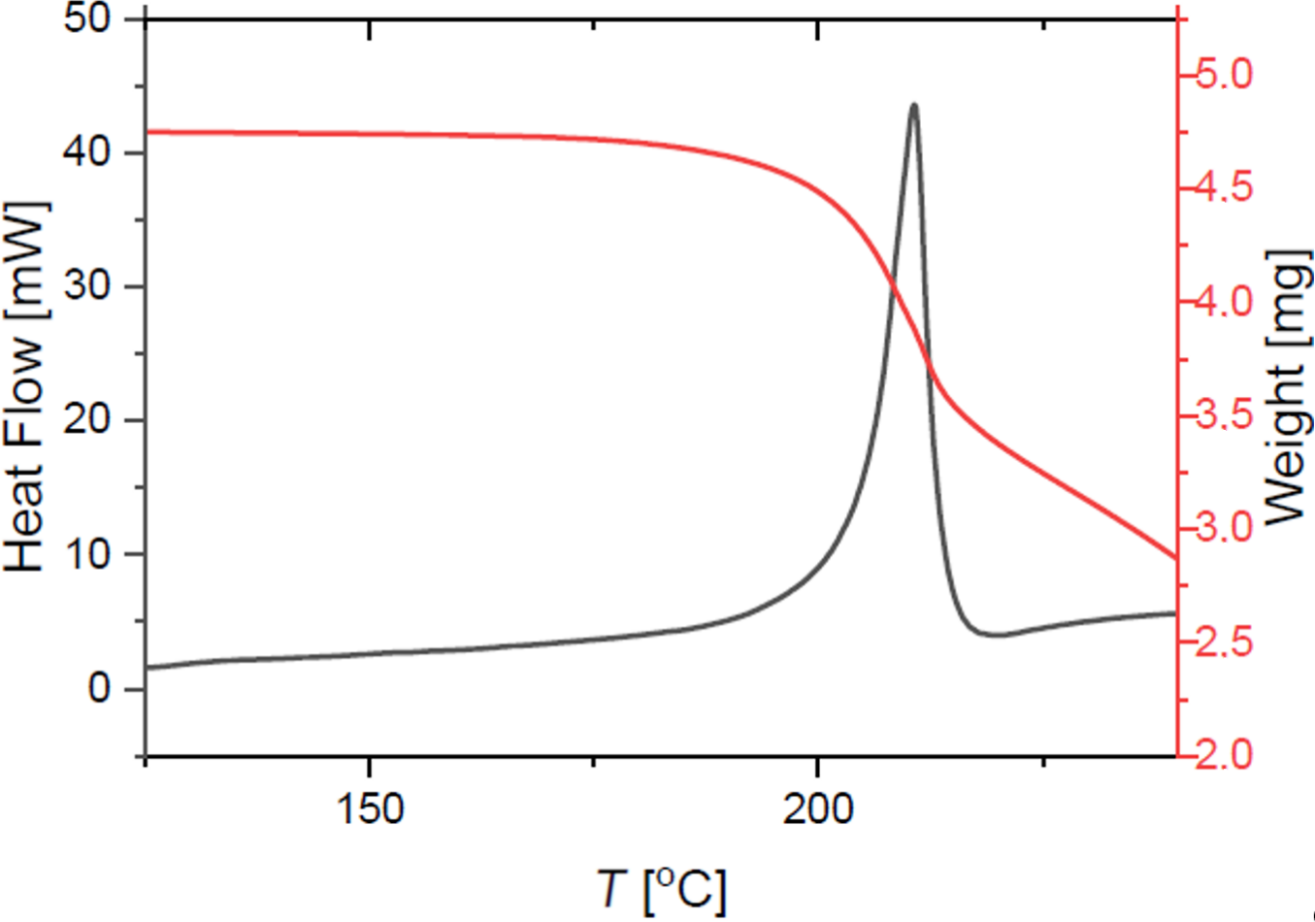
TGA/DSC curves for the cyclic triazole **32**.

Cyclic mesitylene derivatives **30**–**33** have significantly increased Δ*H*_dec_ values. Their TGA/DSC curves reveal a decomposition behavior similar to the pseudocyclic hydroxy(phenyl)-λ^3^-iodanes with an exothermal decomposition without initial melting as exemplarily shown in Figure 9 for compound **32**.

We can thus conclude that the formation of cyclic iodanes results in an undesired increase of Δ*H*_dec_ value and therefore, their pseudocyclic precursors should be used whenever possible.

**Scheme 1:**
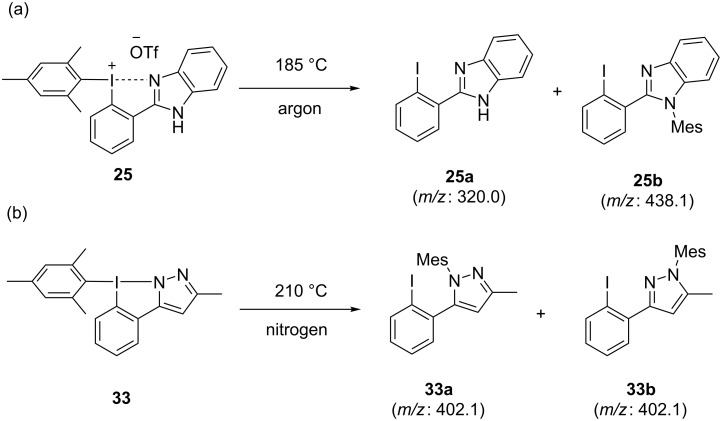
The thermal decomposition of (pseudo)cyclic *N*-heterocycle-stabilized mesityl(aryl)-λ^3^-iodanes **25** and **33**.

Finally, we investigated the decomposition products of one pseudocyclic (**25**) and one cyclic (**33**) diaryliodonium salt. Pseudocyclic salt **25** was heated to 185 °C and the resulting oily residue was analyzed by GC–MS (Scheme 1a). Besides dearylation to aryl iodide **25a** we observed the formation of an *N*-arylated product **25b** in significant amounts. In a similar experiment compound **33** was heated to 160 °C and 210 °C according to the two exothermic peaks observed in the TGA/DSC curve (see Supporting Information File 1). The resulting product mixture was further analyzed by TLC–MS. While at 160 °C no significant decomposition was visible, pronounced decomposition has been observed at 210 °C. The MS analysis revealed the formation of the *N*-arylated pyrazoles **33a** and **33b** as the main products (Scheme 1b).

Both decomposition studies let us conclude, that intermolecular *N*-arylation is the major decomposition pathway of (pseudo)cyclic *N-*heterocycle-stabilized mesityl(phenyl)-λ^3^-iodanes.

## Conclusion

Based on these comprehensive thermoanalytic studies we conclude that (pseudo)cyclic NHIs are throughout safe to use reagents with a sufficient thermal stability. Only the triazole derivatives (**2**, **3**, and **5**) deserve common precautions due to the relatively narrow decomposition peak. In particular, benzothiazole- and pyrazole-substituted hydroxy(phenyl)-λ^3^-iodanes (**12** and **6–8**) show an excellent relation between thermal stability and reactivity, in particular in direct comparison with well-known benziodoxolones. We can also conclude that the pseudocyclic forms of aryl(phenyl)-λ^3^-iodanes should be the reagents of choice as electrophilic aryl group transfer reagents. Thermal decomposition studies indicate that they should be potent electrophilic arene donors.

## Supporting Information

File 1 Synthetic procedures as well as TGA/DSC curves and NMR spectra for all investigated iodanes.
