# Supplementary material for: Thermal stability of N-heterocycle-stabilized iodanes – a systematic investigation
Source: Beilstein J Org Chem. 2019 Sep 27;15:2311–8. doi: 10.3762/bjoc.15.223 (PMC6774080; doi:10.3762/bjoc.15.223)
Supplement: File 1 — Synthetic procedures as well as TGA/DSC curves and NMR spectra for all investigated iodanes. [file Beilstein_J_Org_Chem-15-2311-s001.pdf]

BEILSTEIN JOURNAL OF ORGANIC CHEMISTRY

Supporting Information

for

Thermal stability of *N*-heterocycle-stabilized iodanes – a systematic investigation

Andreas Boelke, Yulia A. Vlasenko, Mekhman S. Yusubov, Boris J. Nachtsheim and Pavel S. Postnikov

Beilstein J. Org. Chem. **2019,** *15,* 2311–2318. doi:10.3762/bjoc.15.223

Synthetic procedures as well as TGA/DSC curves and NMR spectra for all investigated iodanes

License and Terms: This is a supporting information file under the terms of the Creative Commons Attribution License (http://creativecommons.org/licenses/by/4.0). Please note that the reuse, redistribution and reproduction in particular requires that the authors and source are credited.
The license is subject to the *Beilstein Journal of Organic Chemistry* terms and conditions: (https://www.beilstein-journals.org/bjoc)

Table of Content

General information S2

Synthesis of [hydroxy(tosyloxy)iodo]arenes **2–8**, **12–15** S3

Synthesis of [hydroxy(tosyloxy)iodo]arenes **9–11** S9

Synthesis of pseudocyclic diaryliodonium salts **19**, **20**, **22**, **23**, **25–29**: S12

Preparation of carboxylic acid stabilized λ^3-iodanes (**1**, **16**, **18**, **30**) S19

Decomposition studies... S22

Literature S25

TGA/DSC-Curves S26

NMR-Spectra.. S60

General information

All reagents and solvents were purchased from commercial sources and used without further purification from freshly opened containers. ^{1}H NMR spectra were recorded at 360 MHz, 400 MHz and 600 MHz; ^{13}C NMR spectra at 91 MHz, 100 MHz and 150 MHz; and ^{19}F NMR spectra at 188 MHz, 376 MHz and 564 MHz. Chemical shifts for ^{1}H NMR spectra were reported as δ (parts per million) relative to the residual signal of $CHCl_3$ at 7.26 ppm (s), d_4-MeOH 3.31 ppm (quin.) or d_6-DMSO at 2.50 ppm (quin). Chemical shifts for ^{13}C NMR spectra were reported as δ (parts per million) relative to the signal of $CDCl_3$ at 77.0 ppm (t), d_4-MeOH 49.0 (sept.) or d_6-DMSO at 39.5 ppm (sept.). ^{19}F NMR spectra were reported as δ (parts per million) relative to $CFCl_3$ at 0.00 ppm as external standard. HRESI mass spectra were recorded on a Bruker impact II. All signals were reported with the quotient from mass to charge *m/z*.

Simultaneous TGA-DSC testing was carried out on a Mettler Toledo TGA/DSC 3+ STAR^{e} system (and SDT Q600 Thermal Gravimetric Analyser) using 3–13 mg of compounds **1**–**33** under a constant flow of nitrogen gas (20 mL/min and 100 mL/min for **16**). The temperature of oven was held at rt for 1 minute, then heated from rt to 260 °C (or 335 °C) at 10 °C per minute. A reference (empty crucible) was run each day directly prior to measurements. Thin layer chromatography was performed on fluorescence indicator marked precoated silica gel 60 plates (Macherey-Nagel, ALUGRAM Xtra SIL G/UV_{254}) and visualized by UV light (254 nm/366 nm). Flash column chromatography was performed on silica gel (0.040–0.063 mm) with the solvents given in the procedures. TLC-MS: APCI mass spectra were recorded on a Advion Expression CMS^{L} via direct inlet. IR spectra were recorded on a Nicolet Thermo iS10 scientific spectrometer with a diamond ATR unit. The absorption bands were reported in cm^{-1}. Melting points were determined on a Büchi M-5600 Melting Pint apparatus with a heating rate of 5 °C/min. The melting points were reported in °C.

All *ortho*-*N*-heterocyclic substituted iodoarenes were prepared according reported procedures.[1,2] Carboxylic acid stabilized λ^{3}-iodanes were prepared according to reported procedures: **1**[3], **16**[4].

Synthesis of [hydroxy(tosyloxy)iodo]arenes 2–8, 12–15

General Procedure 1.

Following a literature procedure,[1] the corresponding iodoarene (1.0 equiv) was dissolved in DCM (0.25-0.50 mL/mmol) and *m*CPBA (85%, 1.1 equiv) followed by *p*-TsOH·H_2O (1.0 equiv) were added. The reaction mixture was stirred at room temperature for the indicated time. Afterwards the solvent was removed under reduced pressure und Et_2O (10 mL/mmol) was added. The suspension was sonicated for 15 min and was afterwards stored at 4 °C for 30 min. The precipitate was filtered and further washed with Et_2O (3 × 10 mL/mmol). If further purification was needed, the solid was washed with small amounts of EtOAc and/or MeCN (as indicated) to give pure λ^3-iodanes **2–8 + 12–15**.

(2-(1*H*-1,2,3-Triazol-4-yl)phenyl)(hydroxy)iodonium 4-methylbenzenesulfonate (2)

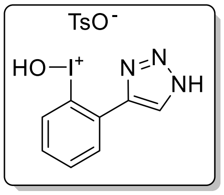

Following GP1, 4-(2-iodophenyl)-1*H*-1,2,3-triazole (542 mg, 2.00 mmol) was stirred in DCM (10 mL) with *m*CPBA (85%, 446 mg, 2.20 mmol) and TsOH·H_2O (380 mg, 2.00 mmol) for 3 h to give (2-(1*H*-1,2,3-triazol-4-yl)phenyl)(hydroxy)iodonium 4-methylbenzenesulfonate (**2**, 848 mg, 1.85 mmol, 93%) as a colourless solid.

^{1}H-NMR (d_4-MeOH, 360 MHz) δ (ppm) 8.77 (s, 1H), 8.17 (d, *J* = 5.4 Hz, 1H), 7.93 (d, *J* = 6.8 Hz, 1H), 7.83 – 7.63 (m, 4H), 7.20 (d, *J* = 6.3 Hz, 2H), 2.34 (s, 3H). ^{13}C-NMR (d_4-MeOH, 91 MHz): δ (ppm) 145.6, 143.5, 141.7, 133.3, 132.8, 129.8, 129.5, 128.5, 128.3, 126.9, 123.4, 118.3, 21.3. IR (ATR): $\tilde{\nu}$ (cm^{-1}) 2776, 1601, 1497, 1449, 1171, 1121, 1031, 1006, 990, 811. HR-MS (ESI) Calculated for $C_9H_9IN_3O^+$ [M-OH-TsO$^-$+MeO]$^+$: *m/z* = 301.97849, found: *m/z* 301.97815. Mp. 136–138 °C.

Hydroxy(2-(5-methyl-1*H*-1,2,3-triazol-4-yl)phenyl)iodonium 4-methylbenzenesulfonate (3)

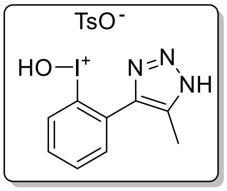

Following GP1, 4-(2-iodophenyl)-5-methyl-1*H*-1,2,3-triazole (1.14 g, 4.00 mmol) was stirred in DCM (20 mL) with *m*CPBA (85%, 0.89 g, 4.40 mmol) and TsOH•H_2O (0.76 g, 4.00 mmol) for 2 h to give hydroxy(2-(5-methyl-1*H*-1,2,3-

triazol-4-yl)phenyl)iodonium 4-methylbenzenesulfonate (**3**, 1.66 g, 3.51 mmol, 88%) as a colourless solid.

^{1}H-NMR (d_6-DMSO, 360 MHz) δ (ppm) 9.73 (brs, 1H), 8.09 (dd, *J* = 7.2, 1.7 Hz, 1H), 7.95 (d, *J* = 7.7 Hz, 1H), 7.83 – 7.70 (m, 2H), 7.47 (d, *J* = 8.0 Hz, 2H), 7.10 (d, *J* = 7.9 Hz, 2H), 2.70 (s, 3H), 2.27 (s, 3H). ^{13}C-NMR (d_6-DMSO, 91 MHz): δ (ppm) 145.4, 139.9, 137.8, 132.8, 131.2, 131.1, 128.1, 127.7, 127.1 (2 x C), 125.5, 117.7, 20.8, 9.3. IR (ATR): $\tilde{\nu}$ (cm^{-1}) 2789, 2458, 1611, 1440, 1242, 1230, 1177, 1117, 1029, 814. HR-MS (ESI) Calculated for $C_{10}H_{11}IN_3O^+$ [M-OH-TsO$^-$+MeO]$^+$: *m/z* = 315.99414, found: *m/z* 315.99403. Mp. 162–163 °C.

Hydroxy(2-(2-methyl-2*H*-1,2,3-triazol-4-yl)phenyl)iodonium 4-methylbenzenesulfonate (4)

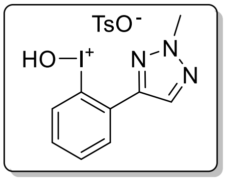

Following GP1, 4-(2-iodophenyl)-2-methyl-2*H*-1,2,3-triazole (356 mg, 1.25 mmol) was stirred in DCM (7 mL) with *m*CPBA (85%, 279 mg, 1.38 mmol) and TsOH•H_2O (238 mg, 1.25 mmol) for 36 h. After washing with Et_2O, the crude solid was suspended in MeCN (10 mL), filtered, washed with additional MeCN (2 mL) and EtOAc (4 mL) to give hydroxy(2-(2-methyl-2*H*-1,2,3-triazol-4-yl)phenyl)iodonium 4-methylbenzenesulfonate (**4**, 337 mg, 0.71 mmol, 57%) as a colourless solid.

^{1}H-NMR (d_6-DMSO, 360 MHz) δ (ppm) 10.57 (brs, 1H), 8.60 (s, 1H), 8.22 (d, *J* = 6.8 Hz, 1H), 8.06 (d, *J* = 7.3 Hz, 1H), 7.86 – 7.72 (m, 2H), 7.46 (d, *J* = 7.8 Hz, 2H), 7.10 (d, *J* = 7.8 Hz, 2H), 4.34 (s, 3H), 2.27 (s, 3H). ^{13}C-NMR (d_6-DMSO, 91 MHz): δ (ppm) 146.0, 145.7, 137.6, 132.0 (3 x C), 130.2, 129.2, 128.3, 128.1, 125.5, 120.1, 42.4, 20.8. IR (ATR): $\tilde{\nu}$ (cm^{-1}) 3360, 2919, 2477, 1443, 1392, 1221, 1140, 1118, 1003, 817. HR-MS (ESI) Calculated for $C_9H_9IN_3O^+$ [M-TsO$^-$]$^+$: *m/z* = 301.97848, found: *m/z* 301.97854. Mp. 151–153°C.

(2-(1*H*-1,2,3-Triazol-1-yl)phenyl)(hydroxy)iodonium 4-methylbenzenesulfonate (5)

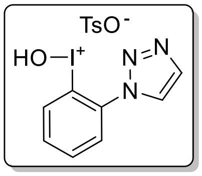

Following GP1, 1-(2-iodophenyl)-1*H*-1,2,3-triazole (515 mg, 1.90 mmol) was stirred in DCM (10 mL) with *m*CPBA (85%, 426 mg, 2.10 mmol) and TsOH•H_2O (361 mg, 1.90 mmol) for 11 h. After washing with Et_2O, the crude product was washed with EtOAc (10 mL) and afterwards was suspended in MeCN (5 mL), filtered and washed with MeCN (2 mL) and Et_2O (3 x 10 mL) to give (2-(1*H*-1,2,3-triazol-1-yl)phenyl)(hydroxy)iodonium 4-methylbenzenesulfonate (**5**, 711 mg, 1.55 mmol, 82%) as a colourless solid.

^{1}H-NMR (d_6-DMSO, 360 MHz) δ (ppm) 10.91 (brs, 1H), 9.38 (s, 1H), 8.39 – 8.29 (m, 2H), 8.06 (d, J = 7.6 Hz, 1H), 7.90 (t, J = 7.2 Hz, 1H), 7.84 (t, J = 7.4 Hz, 1H), 7.47 (d, J = 8.0 Hz, 2H), 7.10 (d, J = 7.8 Hz, 2H), 2.28 (s, 3H). ^{13}C-NMR (d_6-DMSO, 91 MHz): δ (ppm) 145.6, 138.0, 137.0, 133.6, 132.9, 131.8, 130.3, 128.3, 125.7, 124.8, 121.8, 111.2, 21.0. IR (ATR): $\tilde{\nu}$ (cm^{-1}) 3081, 1497, 1445, 1235, 1217, 1146, 1111, 1026, 994, 814. HR-MS (ESI) Calculated for $C_8H_7IN_3O^+$ [M-TsO$^-$]$^+$: *m/z* = 287.96283, found: *m/z* 287.96309. Mp. 132–133°C.

Hydroxy(2-(5-methyl-1*H*-pyrazol-3-yl)phenyl)iodonium 4-methylbenzenesulfonate (6)

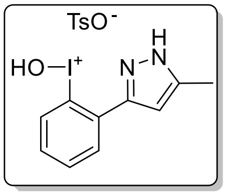

Following GP1 with slight modifications, 5-(2-iodophenyl)-3-methylpyrazole (120 mg, 0.42 mmol) was stirred in MeCN/DCM (4:1, 5 mL) with *m*CPBA (85%, 93.3 mg, 0.46 mmol) and TsOH•H_2O (79.9 mg, 0.42 mmol) for 24 h to give hydroxy(2-(5-methyl-1*H*-pyrazol-3-yl)phenyl)iodonium 4-methylbenzenesulfonate (**6**, 163 mg, 0.35 mmol, 82%) as a colourless solid.

^{1}H-NMR (d_4-MeOH, 360 MHz) δ (ppm) 8.12 (dd, J = 6.8, 1.9 Hz, 1H), 7.91 (d, J = 7.9 Hz, 1H), 7.80 – 7.67 (m, 4H), 7.23 (d, J = 7.9 Hz, 2H), 6.83 (s, 1H), 2.45 (s, 3H), 2.35 (s, 3H). ^{13}C-NMR (d_4-MeOH, 91 MHz): δ (ppm) 151.3, 148.7, 143.4, 141.8, 133.0, 132.6, 131.0, 129.9, 129.4, 128.3, 127.0, 117.8, 102.5, 21.3, 11.5. IR (ATR): $\tilde{\nu}$ (cm^{-1}) 3061, 2913, 2721, 1581, 1560, 1446, 1188, 1164, 1080, 847. HR-

MS (ESI) Calculated for $C_{11}H_{12}IN_2O^+$ [M-OH-TsO$^-$+MeO]$^+$: *m/z* = 314.99889, found: *m/z* 314.99867. Mp. 169–171 °C.

(2-(1*H*-Pyrazol-1-yl)phenyl)(hydroxy)iodonium 4-methylbenzenesulfonate (7)

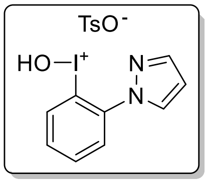

Following GP1, 1-(2-iodophenyl)-1*H*-pyrazole (540 mg, 2.00 mmol) was stirred in DCM (5 mL) with *m*CPBA (85%, 446 mg, 2.20 mmol) and TsOH•H_2O (380 mg, 2.00 mmol) for 24 h to give (2-(1*H*-pyrazol-1-yl)phenyl)(hydroxy)iodonium 4-methylbenzenesulfonate (**7**, 884 mg, 1.93 mmol, 96%) as a colourless solid.

^{1}H-NMR (d_6-DMSO, 360 MHz) δ (ppm) 10.02 (brs, 1H), 9.16 (d, *J* = 2.7 Hz, 1H), 8.32 (dd, *J* = 8.1, 1.2 Hz, 1H), 8.23 (d, *J* = 2.1 Hz, 1H), 7.86 (dd, *J* = 8.2, 1.2 Hz, 1H), 7.81 (td, *J* = 7.7, 1.2 Hz, 1H), 7.75 – 7.69 (m, 1H), 7.47 (d, *J* = 8.1 Hz, 2H), 7.10 (d, *J* = 7.9 Hz, 2H), 6.92 (t, *J* = 2.5 Hz, 1H), 2.28 (s, 3H). ^{13}C-NMR (d_6-DMSO, 91 MHz): δ (ppm) 145.3, 139.2, 137.9, 134.3, 132.1, 129.4, 129.0, 128.2, 127.7, 125.5, 118.2, 110.8, 106.5, 20.8. IR (ATR): $\tilde{\nu}$ (cm^{-1}) 3114, 3046, 1708, 1586, 1506, 1395, 1342, 1164, 1057, 951. HR-MS (ESI) Calculated for $C_9H_8IN_2O^+$ [M-TsO$^-$]$^+$: *m/z* = 286.96759, found: *m/z* 286.96776. Mp. 158–160°C.

(2-(2*H*-Indazol-2-yl)phenyl)(hydroxy)iodonium 4-methylbenzenesulfonate (8)

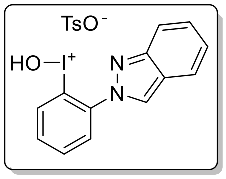

Following GP1, 2-(2-iodophenyl)-2*H*-indazole (128 mg, 0.40 mmol) was stirred in DCM (2 mL) with *m*CPBA (85%, 89.2 mg, 0.44 mmol) and TsOH•H_2O (76.1 mg, 0.40 mmol) for 24 h. After washing with Et_2O, the crude product was further washed with EtOAc (2 x 5 mL) and MeCN (3 mL) to give (2-(2*H*-indazol-2-yl)phenyl)(hydroxy)iodonium 4-methylbenzenesulfonate (**8**, 146 mg, 0.29 mmol, 72%) as a colourless solid.

^{1}H-NMR (d_6-DMSO, 360 MHz) δ (ppm) 10.10 (brs, 1H), 9.85 (s, 1H), 8.60 (d, *J* = 7.6 Hz, 1H), 8.03 (d, *J* = 8.5 Hz, 1H), 8.00 – 7.79 (m, 4H), 7.70 (t, *J* = 7.5 Hz, 1H), 7.47 (d, *J* = 7.6 Hz, 2H), 7.39 (t, *J* =

7.5 Hz, 1H), 7.10 (d, *J* = 7.6 Hz, 2H), 2.28 (s, 3H). ^{13}C-NMR (d_6-DMSO, 91 MHz): δ (ppm) 145.5, 144.2, 137.7, 134.4, 132.2, 131.0, 130. 9, 128.1, 128.1, 125.5, 124.4, 124.1, 122.4, 122.2, 119.9, 114.7, 108.4, 20.7. IR (ATR): $\tilde{\nu}$ (cm^{-1}) 3106, 1662, 1515, 1495, 1379, 12.05, 1123, 1034, 1012, 986. HR-MS (ESI) Calculated for $C_{13}H_{10}IN_2O^+$ [M-TsO$^-$]$^+$: *m/z* = 336.98323, found: *m/z* 336.98303. Mp. 155–157 °C.

(2-(Benzo[*d*]thiazol-2-yl)phenyl)(hydroxy)iodonium 4-methylbenzenesulfonate (12)

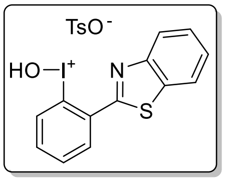

Following GP1, 2-(2-iodophenyl)benzo[*d*]thiazole (337 mg, 1.00 mmol) was stirred in DCM (5 mL) with *m*CPBA (85%, 223 mg, 1.10 mmol) and TsOH•H_2O (190 mg, 1.00 mmol) for 72 h. After washing with Et_2O, the solid was further washed with EtOAc (2 x 5 mL) to give (2-(1*H*-benzo[d]thiazol-2-yl)phenyl)(hydroxy)iodonium 4-methylbenzenesulfonate (**12**, 342 mg, 0.65 mmol, 65%) as an off-white solid.

^{1}H-NMR (d_6-DMSO, 360 MHz) δ (ppm) 9.79 (brs, 1H), 8.55 (d, *J* = 7.2 Hz, 1H), 8.41 (d, *J* = 7.9 Hz, 1H), 8.35 (d, *J* = 8.0 Hz, 1H), 8.17 – 8.00 (m, 2H), 7.90 (t, *J* = 7.1 Hz, 1H), 7.80 (t, *J* = 7.6 Hz, 1H), 7.69 (t, *J* = 7.7 Hz, 1H), 7.47 (d, *J* = 7.9 Hz, 2H), 7.10 (d, *J* = 7.7 Hz, 2H), 2.27 (s, 3H). ^{13}C-NMR (d_6-DMSO, 91 MHz): δ (ppm) 168.8, 146.0, 145.5, 137.9, 135.2, 132.9, 131.6, 130.8, 129.3, 128.6, 128.2, 127.6, 127.6, 125.6, 124.4, 120.6, 120.0, 20.8. IR (ATR): $\tilde{\nu}$ (cm^{-1}) 2917, 2457, 1561, 1456, 1251, 1152, 1034, 990, 820, 757. HR-MS (ESI) Calculated for $C_{13}H_9INOS^+$ [M-TsO$^-$]$^+$: *m/z* = 353.94441, found: *m/z* 353.94396. Mp. 177–179°C.

(2-(Benzo[*d*]oxazol-2-yl)phenyl)(hydroxy)iodonium 4-methylbenzenesulfonate (13)

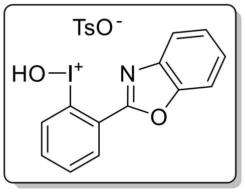

Following GP1, 2-(2-iodophenyl)benzo[*d*]oxazole (401 mg, 1.25 mmol) was stirred in DCM (6 mL) with *m*CPBA (85%, 280 mg, 1.38 mmol) and TsOH•H_2O (238 mg, 1.25 mmol) for 7 h. After washing with Et_2O, the solid was further

washed with EtOAc (2 x 5 mL) to give (2-(1*H*-benzo[d]oxazol-2-yl)phenyl)(hydroxy)iodonium 4-methylbenzenesulfonate (**13**, 608 mg, 1.19 mmol, 95%) as a colourless solid.

^{1}H-NMR (d_6-DMSO, 360 MHz) δ (ppm) 10.22 (brs, 1H), 8.51 (d, *J* = 7.6 Hz, 1H), 8.16 – 8.03 (m, 3H), 8.03 – 7.98 (m, 1H), 7.95 (t, *J* = 7.2 Hz, 1H), 7.73 – 7.58 (m, 2H), 7.48 (d, *J* = 7.9 Hz, 2H), 7.11 (d, *J* = 7.9 Hz, 2H), 2.28 (s, 3H). ^{13}C-NMR (d_6-DMSO, 91 MHz): δ (ppm) 163.9, 150.2, 145.7, 137.7, 135.7, 135.7, 131.5, 129.4, 128.1, 127.7, 127.5, 126.9, 125.5, 123.2, 121.8, 118.5, 112.5, 20.8. IR (ATR): $\tilde{\nu}$ (cm^{-1}) 3103, 2870, 2454, 1584, 1470, 1367, 1244, 1121, 1030, 809. HR-MS (ESI) Calculated for $C_{13}H_9INO_2^+$ [M-TsO$^-$]$^+$: *m/z* = 337.96725, found: *m/z* 337.96719. Mp. 169–170 °C.

(2-(4,5-Diphenyloxazol-2-yl)phenyl)(hydroxy)iodonium 4-methylbenzenesulfonate (14)

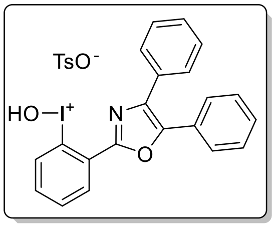

Following GP1, 2-(2-iodophenyl)-4,5-diphenyloxazole (212 mg, 0.50 mmol) was stirred in DCM (2.5 mL) with *m*CPBA (85%, 112 mg, 0.55 mmol) and TsOH•H_2O (95.1 mg, 0.50 mmol) for 1 h to give (2-(4,5-diphenyloxazol-2-yl)phenyl)(hydroxy)iodonium 4-methylbenzenesulfonate (**14**, 260 mg, 0.43 mmol, 86%) as a colourless solid.

^{1}H-NMR (d_6-DMSO, 360 MHz) δ (ppm) 10.52 (brs, 1H), 8.44 (d, *J* = 7.5 Hz, 1H), 8.04 (d, *J* = 3.5 Hz, 2H), 7.94 – 7.86 (m, 1H), 7.76 – 7.62 (m, 4H), 7.62 – 7.50 (m, 6H), 7.47 (d, *J* = 7.9 Hz, 2H), 7.09 (d, *J* = 7.8 Hz, 2H), 2.27 (s, 3H). ^{13}C-NMR (d_6-DMSO, 91 MHz): δ (ppm) 160.4, 147.0, 145.6, 137.7, 134.6, 133.2, 131.4, 130.5, 130.0, 129.5, 129.4, 128.2, 128.1, 128.1, 127.9, 127.6, 126.8, 126.4, 125.5, 123.1, 120.2, 20.8. IR (ATR): $\tilde{\nu}$ (cm^{-1}) 2863, 2479, 1595, 1384, 1241, 1146, 1113, 1028, 1004, 842. HR-MS (ESI) Calculated for $C_{21}H_{15}INO_2^+$ [M-TsO$^-$]$^+$: *m/z* = 440.01420, found: *m/z* 440.01405. Mp. 153–154°C.

(2-(4,5-Diphenyl-1*H*-imidazol-2-yl)phenyl)(hydroxy)iodonium 4-methylbenzenesulfonate (15)

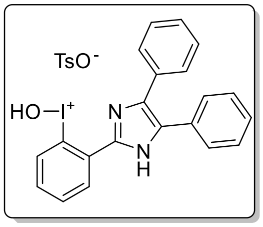

Following GP1, 2-(2-iodophenyl)-4,5-diphenyl-1*H*-imidazole (844 mg, 2.00 mmol) was stirred in DCM (10 mL) with *m*CPBA (85%, 446 mg, 2.20 mmol) and $TsOH•H_2O$ (380 mg, 2.00 mmol) for 71 h. After washing with Et_2O, the solid was further washed with EtOAc (2 x 5 mL) and MeCN (2 x 5 mL) to give (2-(4,5-diphenyl-1*H*-imidazol-2-yl)phenyl)(hydroxy)iodonium 4-methylbenzene-sulfonate (**15**, 1.10 g, 1.80 mmol, 90%) as a colourless solid.

^{1}H-NMR (d_6-DMSO, 360 MHz) δ (ppm) 14.40 (brs, 1H), 9.31 (brs, 1H), 8.48 – 8.38 (m, 1H), 8.06 (d, *J* = 6.6 Hz, 1H), 7.95 – 7.76 (m, 2H), 7.53 – 7.42 (m, 12H), 7.10 (d, *J* = 7.5 Hz, 2H), 2.28 (s, 3H). ^{13}C-NMR (d_6-DMSO, 91 MHz): δ (ppm) 145.5, 145.4, 137.8, 133.7, 132.2, 131.1, 130.4, 130.2, 129.4, 129.2, 129.1, 128.6, 128.5, 128.5, 128.3, 128.1, 127.7, 127.6, 126.5, 125.5, 118.6, 20.8. IR (ATR): $\tilde{\nu}$ (cm^{-1}) 2998, 2666, 1600, 1474, 1447, 1231, 1143, 1031, 1004, 820. HR-MS (ESI) Calculated for $C_{22}H_{18}IN_2O^+$ [M-OH-TsO$^-$+MeO]$^+$: *m/z* = 453.04584, found: *m/z* 453.04552. Mp. 121–123 °C.

Synthesis of [Hydroxy(tosyloxy)iodo]arenes 9-11

General Procedure 2.

Following a literature procedure,[2] to a stirred solution of 2-(2-iodophenyl)-1*H*-benzo[*d*]imidazoles (1.0 mmol) in 5 mL of DCM-TFE mixture (1:1) was added mCPBA (77%, 0.69 g, 2.0 mmol) and p-$TsOH•H_2O$ (0.27 g, 1.4 mmol). The stirring was continued at room temperature until full conversion of starting material (monitored by TLC, eluent hexane: EtOAc = 2:7), and solvents was evaporated at reduced pressure. The products were precipitated by the addition of Et_2O. The solids were filtered off, washed with Et_2O three times and dried in a vacuum.

(2-(1*H*-Benzo[*d*]imidazol-2-yl)phenyl)(hydroxy)iodonium 4-methylbenzenesulfonate (9)

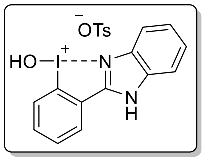

Reaction of 2-(2-iodophenyl)-1*H*-benzo[*d*]imidazole (0.32 g, 1.0 mmol) with mCPBA and p-TsOH•H_2O according to the GP 2 afforded 0.503 g (99%) of product **9**, isolated as a beige solid: mp 191−192 °C; ^{1}H NMR (400 MHz, DMSO-d_6): δ = 14.68 (s, 1H), 8.50 (dd, *J* = 7.6, 1.6 Hz, 1H), 8.11 (dd, *J* = 8.0, 1.6 Hz, 1H), 8.01-7.91 (m, 3H), 7.83-7.80 (m, 1H), 7.51-7.47 (m, 4H), 7.11 (d, *J* = 8.0 Hz, 2H), 2.28 (s, 3H); ^{13}C NMR (100 MHz, DMSO-d_6): δ = 151.2, 145.7, 139.7, 137.8, 136.6, 134.4, 133.9, 131.3, 128.1, 127.8, 125.5, 125.3, 124.6, 121.0, 116.8, 113.5, 20.8.

(2-(5,6-Dimethyl-1*H*-benzo[*d*]imidazol-2-yl)phenyl)(hydroxy)iodonium 4-methylbenzenesulfonate (10)

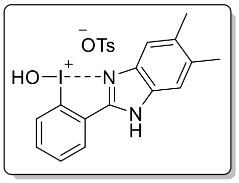

Reaction of 2-(2-iodophenyl)-1*H*-benzo[*d*]imidazole (0.35 g, 1.0 mmol) with mCPBA and p-TsOH•H_2O according to the GP 2 afforded 0.509 g (95%) of product **10**, isolated as a beige solid: mp 186−187 °C; ^{1}H NMR (400 MHz, DMSO-d_6): δ = 14.41 (s, 1H), 8.44 (dd, *J* = 7.6, 1.6 Hz, 1H), 8.08 (d, *J* = 8.0 Hz, 1H), 7.97 - 7.88 (m, 2H), 7.71 (s, 1H), 7.54 (s, 1H), 7.48 (d, *J* = 8.0 Hz, 2H), 7.10 (d, *J* = 7.6 Hz, 2H), 2.38 (s, 3H), 2.36 (s, 3H) 2.28 (s, 3H); ^{13}C NMR (100 MHz, DMSO-d_6): δ = 150.1, 145.7, 137.7, 135.1, 135.0, 133.6, 133.5, 132.5, 131.2, 128.1, 127.7, 125.5, 125.4, 120.8, 116.5, 113.1, 20.8, 20.1, 20.0.

(2-(5,6-Difluoro-1*H*-benzo[*d*]imidazol-2-yl)phenyl)(hydroxy)iodonium 4-methylbenzenesulfonate (11)

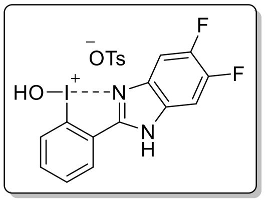

Reaction of 2-(2-iodophenyl)-1*H*-benzo[*d*]imidazole (0.36 g, 1.0. mmol) with mCPBA and p-TsOH•H_2O according to the GP 2 afforded 0.50 g (92%) of

product **11**, isolated as a light brown solid: mp 167−168 °C; ^{1}H NMR (400 MHz, DMSO-d_6): δ = 14.95 (s, 1H), 8.46 (dd, *J* = 7.6, 1.6 Hz, 1H), 8.08-8.03 (m, 2H), 7.99-7.93 (m, 2H), 7.90 (td, *J* = 7.6, 0.8 Hz, 1H), 7.51 (d, *J* = 8.0 Hz, 2H), 7.12(d, *J* = 7.6 Hz, 2H), 2.28 (s, 3H); ^{13}C NMR (100 MHz, DMSO-d_6): δ =152.9, 149.7 (d, $J_{C\text{-}F}$ = 15.2 Hz), 149.0 (d, $J_{C\text{-}F}$ = 14.9 Hz), 147.3 (d, $J_{C\text{-}F}$ = 15.2 Hz), 146.6 (d, $J_{C\text{-}F}$ = 15.2 Hz), 145.3, 138.0, 134.0, 132.4 (d, $J_{C\text{-}F}$ = 11.5 Hz), 131.3, 129.8 (d, $J_{C\text{-}F}$ = 11.7 Hz), 128.2, 128.1, 127.5, 125.5, 125.0, 120.7, 104.9 (d, $J_{C\text{-}F}$ = 22.0 Hz), 102.1 (d, $J_{C\text{-}F}$ = 22.9 Hz), 20.8; ^{19}F NMR (376 MHz, DMSO-d6): δ = -138.6 (d, *J* = 22.6 Hz), -139.9 (d, *J* = 22.6 Hz).

8*H*-8λ^3-Benzo[4,5][1,2]iodazolo[2,3-*c*][1,2,3]triazol-8-ol (17)

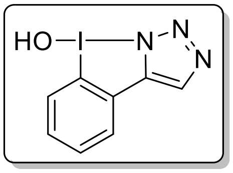

Following a described procedure,[1] to (2-(1*H*-1,2,3-triazol-4-yl)phenyl)(hydroxy)iodonium 4-methylbenzenesulfonate (**2**, 459 mg, 1.00 mmol) was added dropwise a 10% NaOH-solution in H_2O (4 mL) over the course of 5 min under stirring, forming a clear orange solution which was stirred at room temperature for 1 h. H_2O (4 mL) was added and stirring continued for 2 h. The reaction mixture was neutralised with dropwise addition of 1 M HCl-solution in H_2O upon which a white precipitate was formed. The flask was stored at 4 °C for 30 min and then filtered. The solid was washed with H_2O (2 x 15 mL) and Et_2O (2 x 10 mL). Drying *in vacuo* afforded 8*H*-8λ^3-benzo[4,5][1,2]iodazolo[2,3-*c*][1,2,3]triazol-8-ol (**21**, 230 mg, 0.800 mmol, 80%) as an off-white solid.

^{1}H-NMR (d_4-MeOH, 360 MHz) δ (ppm) 8.14 (s, 1H), 8.03 (d, *J* = 7.2 Hz, 1H), 7.91 (d, *J* = 7.9 Hz, 1H), 7.75 – 7.54 (m, 2H). ^{13}C-NMR (d_4-MeOH, 91 MHz): δ (ppm) 143.9, 132.4, 131.6, 130.4, 128.9, 128.4, 128.0, 119.5. IR (ATR): $\tilde{\nu}$ (cm^{-1}) 2833, 2395, 1594, 1443, 1361, 1150, 1076, 1001, 973, 755. HR-MS (ESI) Calculated for $C_9H_9IN_3O^+$ $[M\text{-}OH\text{+}MeO\text{+}H]^+$: *m/z* = 301.97849, found: *m/z* 301.97837. Mp. 170–172 °C.

Synthesis of pseudocyclic diaryliodonium salts 19, 20, 22, 23, 25–29:

General Procedure 3.

Following a literature procedure,[2] to a stirred solution of [hydroxy(tosyloxy)iodo]arenes (0.200 mmol) in 1.5 mL TFE were added trifluoromethanesulfonic acid (0.026 mL, 0.300 mmol) and reaction mixture was stirred during 10 min. Then mesitylene (0.056 mL, 0.048 g, 0.400 mmol) was added and stirring was continued until full conversion of starting material according to NMR (temperature and reaction times are provided in the text). The solvent was evaporated at room temperature. The products were precipitated by the addition of Et_2O. The solids were filtered off, washed with Et_2O three times and dried in a vacuum.

Mesityl(2-(1*H*-1,2,3-triazol-4-yl)phenyl)iodonium trifluoromethanesulfonate (19)

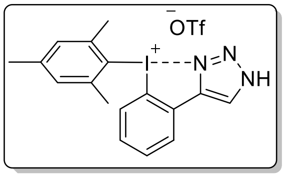

Reaction of [hydroxy(tosyloxy)iodo]arene **2** (0.092 g, 0.200 mmol) with mesitylene according to the GP 3 at room temperature during 3 h afforded 0.102 g (95%) of product **20**, isolated as a colourless solid: mp 142−144 °C; ^{1}H NMR (601 MHz, DMSO-d$_6$) δ = δ 8.97 (s, 1H), 8.17 (d, *J* = 7.6 Hz, 1H), 7.70 (t, *J* = 7.5 Hz, 1H), 7.41 (t, *J* = 7.8 Hz, 1H), 7.37 (s, 2H), 6.92 (d, *J* = 8.1 Hz, 1H), 2.49 (s, 6H), 2.40 (s, 3H); ^{13}C NMR (151 MHz, DMSO-d$_6$): δ = 144.3, 143.9, 142.9, 132.0, 131.5, 130.2, 130.1, 130.0, 129.4, 120.7 (q, *J* = 322.4 Hz), 120.1, 109.7, 26.1, 20.8; ^{19}F NMR (565 MHz, DMSO-d$_6$): δ = -77.8; IR (ATR): $\tilde{\nu}$ (cm^{-1}) 3125, 1600, 1452, 1280, 1222, 1155, 1020, 988, 757. HRMS (ESI-positive ionization): calcd for $C_{17}H_{17}IN_3^+$ ([M-OTf]$^+$): 390.04617, found: 390.04596.

Mesityl(2-(5-methyl-1*H*-1,2,3-triazol-4-yl)phenyl)iodonium trifluoromethanesulfonate (20)

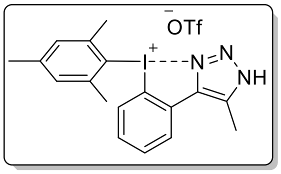

Reaction of [hydroxy(tosyloxy)iodo]arene **3** (0.095 g, 0.200 mmol) with mesitylene according to the GP 3 at room temperature during 3 h afforded 0.105 g (95%) of product **20**, isolated as a colourless solid: mp 149−151 °C; ^{1}H NMR (601 MHz, DMSO-d$_6$) δ = 7.95 (d, *J* = 7.5 Hz, 1H), 7.72 (t, *J* = 7.5 Hz, 1H), 7.43 (t, *J* = 7.7 Hz, 1H), 7.34 (s, 2H), 7.08 (d, *J* = 8.1 Hz, 1H), 2.57 (s, 3H), 2.46 (s, 6H), 2.39 (s, 3H); ^{13}C NMR (151 MHz, DMSO-d$_6$): δ = 144.1, 142.6, 140.0, 131.6, 131.4, 131.0, 130.4, 130.0, 129.6, 120.8, 120.7 (q, *J* = 322.3 Hz), 111.4, 26.0, 20.8, 9.7. ^{19}F NMR (564 MHz, DMSO-d$_6$): δ = -77.8; IR (ATR): $\tilde{\nu}$ (cm^{-1}) 2647, 1637, 1457, 1286, 1221, 1157, 1019, 991, 766. HRMS (ESI-positive ionization): calcd for $C_{18}H_{19}IN_3^+$ ([M-OTf]$^+$): 404.06182, found: 404.06160.

Mesityl(2-(2-methyl-2*H*-1,2,3-triazol-4-yl)phenyl)iodonium trifluoromethanesulfonate (22)

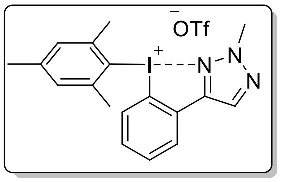

Reaction of [hydroxy(tosyloxy)iodo]arene **4** (0.095 g, 0.200 mmol) with mesitylene according to the GP 3 at room temperature during 4.5 h afforded 0.093 g (81%) of product **22**, isolated as a beige solid: mp 211−212 °C; ^{1}H NMR (600 MHz, DMSO-d$_6$) δ = 8.57 (s, 1H), 8.17 (d, *J* = 7.8 Hz, 1H), 7.73 (t, *J* = 7.2 Hz, 1H), 7.45 (t, *J* = 7.8 Hz, 1H), 7.35 (s, 2H), 7.11 (d, *J* = 8.4 Hz, 1H), 4.34 (s, 3H), 2.47 (s, 6H), 2.39 (s, 3H); ^{13}C NMR (100 MHz, DMSO-d$_6$): δ = 145.2, 144.2, 142.6, 132.8, 132.3, 131.6, 130.8, 130.6, 130.2, 130.0, 120.69 (q, J_{CF} = 320 Hz, $CF_3SO_3^-$), 120.4, 111.1, 42.3, 26.1, 20.7; ^{19}F NMR (564 MHz, DMSO-d$_6$): δ = -77.8; HRMS (ESI-positive ionization): calcd for $C_{18}H_{19}IN_3^+$ ([M-OTf]$^+$): 404.06182, found: 404.06148.

(2-(1*H*-Pyrazol-1-yl)phenyl)(mesityl)iodonium trifluoromethanesulfonate (23)

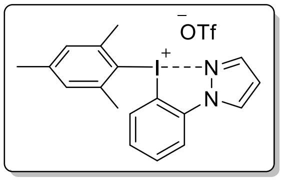

Reaction of [hydroxy(tosyloxy)iodo]arene **7** (0.092 g, 0.200 mmol) with mesitylene according to the GP 3 at room temperature during 4.5 h afforded 0.094 g (84%) of product **23**, isolated as a beige solid: mp 201−202 °C; ^{1}H NMR (600 MHz, DMSO-d$_6$) δ = 8.93 (d, *J* = 2.4 Hz, 1H), 8.21 (d, *J* = 8.4 Hz, 1H), 8.14 (s, 1H), 7.75 (t, *J* = 7.8 Hz, 1H), 7.37 – 7.34 (m, 3H), 6.89 – 6.87 (m, 2H), 2.51 (s, 6H), 2.41 (s, 3H); ^{13}C NMR (100 MHz, DMSO-d$_6$): δ = 144.3, 142.8, 140.5, 137.0, 132.2, 130.0, 129.8, 129.5, 121.5, 120.69 (q, J_{CF} = 320 Hz, $CF_3SO_3^-$), 110.1, 101.0, 26.0, 20.8; ^{19}F NMR (564 MHz, DMSO-d$_6$): δ = -77.8; HRMS (ESI-positive ionization): calcd for $C_{18}H_{18}IN_2^+$ ([M-OTf]$^+$): 389.05092, found: 389.05062.

(2-(1*H*-Benzo[*d*]imidazol-2-yl)phenyl)(mesityl)iodonium trifluoromethanesulfonate (25)

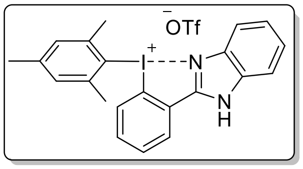

Reaction of [hydroxy(tosyloxy)iodo]arene **9** (0.102 g, 0.200 mmol) with mesitylene according to the GP 3 at room temperature during 8 h afforded 0.116 g (99%) of product **25**, isolated as a beige solid: mp 195−196 °C; ^{1}H NMR (600 MHz, DMSO-d$_6$) δ = 13.88 (s, 1H), 8.47 (d, *J* = 7.8 Hz, 1H), 7.86 (t, *J* = 7.2 Hz, 1H), 7.77 (br.s, 2H), 7.58 (t, *J* = 7.8 Hz, 1H), 7.43-7.41 (m, 2H), 7.37 (s, 2H), 6.92 (d, *J* = 8.4 Hz, 1H), 2.53 (s, 6H), 2.42 (s, 3H); ^{13}C NMR (150 MHz, DMSO-d$_6$): δ = 149.3, 144.0, 143.0, 133.8, 131.5, 130.0, 129.4, 128.9, 127.8, 121.0, 120.71 (q, J_{CF} = 320 Hz, $CF_3SO_3^-$), 114.6, 111.6, 26.0, 20.9; ^{19}F NMR (564 MHz, DMSO-d$_6$): δ = -77.8.

(2-(5,6-Dimethyl-1*H*-benzo[d]imidazol-2-yl)phenyl)(mesityl)iodonium trifluoromethanesulfonate (26)

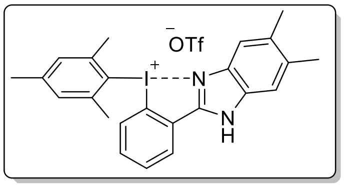

Reaction of [hydroxy(tosyloxy)iodo]arene **10** (0.107 g, 0.200 mmol) with mesitylene according to the GP 3 at room temperature during 8 h afforded 0.113 g (92%) of product **26**, isolated as a beige solid: mp 215−216 °C; ^{1}H NMR (400 MHz, DMSO-d$_6$) δ = 13.64 (s, 1H), 8.43 (dd, *J* = 8.0, 1.6 Hz, 1H), 7.84 (td, *J* = 7.6, 0.4 Hz,

1H), 7.57 – 7.52 (m, 2H), 7.49 (s, 1H), 7.37 (s, 2H), 6.90 (dd, J = 8.4, 0.4 Hz, 1H), 2.52 (s, 6H), 2.42 (s, 3H), 2.40 (s, 3H), 2.39 (s, 3H); ^{13}C NMR (150 MHz, DMSO-d$_6$): δ = 148.3, 144.0, 143.0, 139.6, 138.8,134.0, 133.4, 133.1, 132.3, 131.4, 130.0, 129.1, 128.8, 127.9, 121.1, 120.70 (q, J_{CF} = 320 Hz, $CF_3SO_3^-$), 118.2, 112.3, 111.3, 26.0, 20.9, 20.2, 20.1; ^{19}F NMR (376 MHz, DMSO-d$_6$): δ = -77.8; HRMS (ESI-positive ionization): calcd for $C_{24}H_{24}IN_2^+$ ([M-OTf]$^+$): 467.09787, found: 467.09757.

(2-(5,6-Difluoro-1*H*-benzo[*d*]imidazol-2-yl)phenyl)(mesityl)iodonium trifluoromethanesulfonate (27)

Reaction of [hydroxy(tosyloxy)iodo]arene **11** (0.109 g, 0.200 mmol) with mesitylene according to the GP 3 at room temperature during 8 h afforded 0.113 g (91%) of product **27**, isolated as a beige solid: mp 166-167 °C; ^{1}H NMR (400 MHz, DMSO-d$_6$) δ = 14.14 (s, 1H), 8.45 (dd, J = 7.6, 1.2 Hz, 1H), 7.88 – 7.83 (m, 3H), 7.60 – 7.56 (m, 1H), 7.38 (s, 2H), 6.92 (dd, J = 8.4, 0.8 Hz, 1H), 2.52 (s, 6H), 2.42 (s, 3H); ^{13}C NMR (100 MHz, DMSO-d$_6$): δ = 151.3, 144.1, 142.9, 135.8 (d, J_{CF} = 11.7 Hz), 134.0, 131.5, 130.3 (d, J_{CF} = 12.1 Hz), 130.0, 129.6, 129.0, 127.5, 120.9, 120.69 (q, J_{CF} = 320 Hz, $CF_3SO_3^-$), 111.5, 106.0 (d, J_{CF} = 20.1 Hz), 100.8 (d, J_{CF} = 22.6 Hz), 26.0, 20.8; ^{19}F NMR (376 MHz, DMSO-d6): δ = -77.8, -139.9 (d, J = 22.18 Hz), -142.1 (d, J = 21.81 Hz).

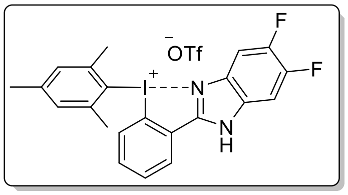

(2-(Benzo[*d*]oxazol-2-yl)phenyl)(mesityl)iodonium trifluoromethanesulfonate (28)

Reaction of [hydroxy(tosyloxy)iodo]arene **13** (0.102 g, 0.200 mmol) with mesitylene according to the GP 3 at room temperature during 6 h afforded 0.098 g (80%) of product **28**, isolated as a beige solid: mp 199–200 °C; ^{1}H NMR (600 MHz, DMSO-d$_6$) δ = 8.51 (d, J = 7.2 Hz, 1H), 7.99 (d, J = 7.8 Hz, 1H), 7.95 (d, J = 7.2 Hz, 1H), 7.87 (t, J = 7.8 Hz, 1H), 7.69 (t, J = 7.8 Hz, 1H), 7.63 – 7.58 (m, 2H), 7.41 (s, 2H), 6.97 (d, J = 8.4 Hz, 1H), 2.55 (s, 6H), 2.43 (s, 3H); ^{13}C NMR (100 MHz, DMSO-d$_6$): δ = 161.0, 150.0, 144.5, 143.1, 138.9, 135.6, 131.7, 131.0, 130.2, 129.3, 127.2, 126.1, 125.6, 120.69 (q, J_{CF} = 320 Hz, $CF_3SO_3^-$

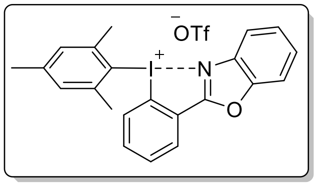

), 119.9, 119.8, 112.2, 111.8, 26.2, 20.8; ^{19}F NMR (564 MHz, DMSO-d_6): δ = -77.8; HRMS (ESI-positive ionization): calcd for $C_{22}H_{19}INO^+$ ([M-OTf]$^+$): 440.05059, found: 440.05028.

(2-(4,5-Diphenyloxazol-2-yl)phenyl)(mesityl)iodonium trifluoromethanesulfonate (29)

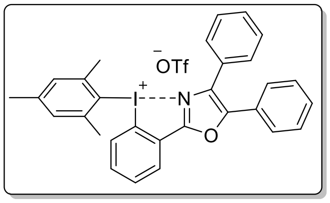

Reaction of [hydroxy(tosyloxy)iodo]arene **14** (0.122 g, 0.200 mmol) with mesitylene according to the GP 3 at room temperature during 6 h afforded 0.021 g (15%) of product **29**, isolated as a beige solid: mp 188−189 °C; ^{1}H NMR (600 MHz, DMSO-d_6) δ = 8.47 (d, *J* = 7.2 Hz, 1H), 7.84 (t, *J* = 7.2 Hz, 1H), 7.78 (t, *J* = 6.0 Hz, 4H), 7.63 (t, *J* = 7.2 Hz, 1H), 7.57 – 7.51 (m, 6H), 7.42 (s, 2H), 6.95 (d, *J* = 8.4 Hz, 1H), 2.56 (s, 6H), 2.44 (s, 3H); ^{13}C NMR (150 MHz, DMSO-d_6): δ = 157.9, 146.6, 144.5, 143.1, 136.4, 134.8, 134.5, 131.6, 130.2, 130.1, 129.7, 129.3, 129.2, 129.1, 127.5, 127.2, 127.0, 125.3, 120.67 (q, J_{CF} = 319 Hz, $CF_3SO_3^-$), 119.8, 117.0, 114.5, 110.9, 26.1, 20.8; ^{19}F NMR (564 MHz, DMSO-d_6): δ = -77.8; HRMS (ESI-positive ionization): calcd for $C_{30}H_{25}INO^+$ ([M-OTf]$^+$): 542.09754, found: 542.09727.

(2-(1*H*-1,2,3-Triazol-1-yl)phenyl)(mesityl)iodonium triflate (21)

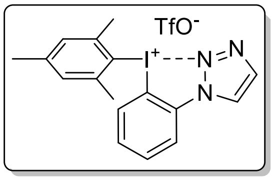

Following a described procedure,[1] mesitylene (28.8 mg, 0.240 mmol) and (2-(1*H*-1,2,3-triazol-1-yl)phenyl)(hydroxy)iodonium 4-methylbenzenesulfonate (**5**, 91.9 mg, 0.200 mmol) were dissolved in TFE (1 mL) and TfOH (52.7 µL, 0.600 mmol) was added dropwise. The red solution was stirred for 2 h at room temperature and was then concentrated under reduced pressure. The residue was dissolved in small amounts of DCM and was purified via a short silica column eluting with DCM/MeOH 100:0→20:1→10:1. After removal of the solvent, the oily residue was dissolved in $CHCl_3$ (1 mL) and Et_2O (3 mL) and the mixture was concentrated *in vacuo* to give (2-(1*H*-1,2,3-triazol-1-yl)phenyl)(mesityl)iodonium triflate (**21**, 94.6 mg, 0.176 mmol, 88%) as a colourless solid.

^{1}H-NMR (d_6-DMSO, 360 MHz) δ (ppm) 9.07 (d, *J* = 1.2 Hz, 1H), 8.24 (d, *J* = 1.2 Hz, 1H), 8.11 (dd, *J* = 8.0, 1.4 Hz, 1H), 7.85 (td, *J* = 7.9, 1.2 Hz, 1H), 7.56 (td, *J* = 7.9, 1.4 Hz, 1H), 7.45 (dd, *J* = 8.2, 1.2

Hz, 1H), 7.31 (s, 2H), 2.45 (s, 6H), 2.36 (s, 3H). ^{13}C-NMR (DMSO-d_6, 91 MHz): δ (ppm) 144.2, 142.4, 135.7, 135.6, 133.0, 132.8, 132.2, 130.1, 125.5, 125.1, 122.0, 120.7 (q, J = 322.6 Hz), 105.8, 26.0, 20.7. ^{19}F-NMR (DMSO-d_6, 188 MHz): δ (ppm) -78.97. IR (ATR): $\tilde{\nu}$ (cm^{-1}) 3109, 2929, 1587, 1497, 1449, 1244, 1162, 1025, 978, 820. HR-MS (ESI) Calculated for $C_{17}H_{17}IN_3^+$ [M-TfO$^-$]$^+$: *m/z* = 390.04617, found: *m/z* 390.04556. Mp. 182–184°C.

Mesityl(2-(5-methyl-1*H*-pyrazol-3-yl)phenyl)iodonium trifluoromethanesulfonate (24)

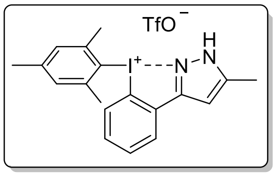

Following a slightly modified procedure,[2] to a solution of [hydroxy(tosyloxy)iodo]arene **6** (354 mg, 0.750 mmol) in TFE (3.5 mL) was added TfOH (99.3 µL, 1.13 mmol) at room temperature. After stirring for 5 min mesitylene (180 mg, 1.50 mmol) was added and stirring continued for 5.5 h. Afterwards the solvent was removed *in vacuo* and the residue was purified via column chromatography on silica (DCM/MeOH 40+1 → 10+1) to give product **24** (390 mg, 0.706 mmol, 94%) as a colourless solid.

^{1}H-NMR (CDCl$_3$, 600 MHz) δ (ppm) 12.39 (s, 1H), 7.86 (dd, J = 7.6, 1.7 Hz, 1H), 7.57 (t, J = 7.4 Hz, 1H), 7.20 (t, J = 7.8 Hz, 1H), 7.17 (s, 2H), 6.77 (d, J = 8.4 Hz, 1H), 6.48 (s, 1H), 2.52 (s, 6H), 2.43 (s, 6H). ^{13}C-NMR (CDCl$_3$, 151 MHz): δ (ppm) 147.1, 144.9, 143.9, 142.7, 132.2, 131.1, 130.8, 130.2, 129.2, 127.4, 120.6 (q, J = 319.7 Hz), 118.0, 108.2, 100.9, 26.7, 21.4, 11.1. ^{19}F-NMR (CDCl$_3$, 565 MHz): δ (ppm) -77.8. IR (ATR): $\tilde{\nu}$ (cm^{-1}) 3221, 1578, 1446, 1251, 1223, 1156, 1026, 963, 760. HR-MS (ESI) Calculated for $C_{19}H_{20}IN_2^+$ [M-TfO$^-$]$^+$: *m/z* = 403.06657, found: *m/z* 403.06637. Mp. 87–88°C.

8-Mesityl-8*H*-8λ^3-benzo[4,5][1,2]iodazolo[2,3-*c*][1,2,3]triazole (31)

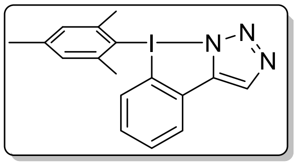

Iodonium salt **19** (108 mg, 0.200 mmol) was suspended in CHCl$_3$ (5 mL) and an aqueous 10% NaOH solution (1 mL) was added. The mixture was stirred vigorously for 1 h at room temperature, before it was diluted with CHCl$_3$ and water (5 mL each). The phases were separated and the aqueous phase was extracted with CHCl$_3$

(2 x 5 mL). The combined organic phases were concentrated *in vacuo* to give product **31** (77.7 mg, 0.200 mmol, 100%) as an off-white solid.

^{1}H-NMR ($CDCl_3$, 600 MHz) δ (ppm) 8.00 (s, 1H), 7.84 (dd, *J* = 7.7, 1.6 Hz, 1H), 7.44 (td, *J* = 7.4, 1.1 Hz, 1H), 7.23 (s, 2H), 7.02 (ddd, *J* = 8.5, 7.1, 1.6 Hz, 1H), 6.85 (dd, *J* = 8.3, 1.1 Hz, 1H), 2.64 (s, 6H), 2.46 (s, 3H). ^{13}C-NMR ($CDCl_3$, 151 MHz): δ (ppm) 143.2, 142.9, 142.7, 133.6, 130.1, 129.8, 128.6, 128.5, 128.1, 122.4, 113.2, 26.8, 21.4. IR (ATR): $\tilde{\nu}$ (cm^{-1}) 2975, 1588, 1524, 1437, 1298, 1124, 1069, 998, 966, 760. HR-MS (ESI) Calculated for $C_{17}H_{17}IN_3^+$ $[M\text{-}TfO^-]^+$: *m/z* = 390.04617, found: *m/z* 390.04594. Mp. 197–199°C.

8-Mesityl-3-mesityl-8*H*-8λ³-benzo[4,5][1,2]iodazolo[2,3-*c*][1,2,3]triazole (32)

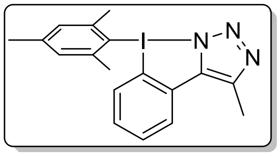

Iodonium salt **20** (111 mg, 0.200 mmol) was suspended in $CHCl_3$ (5 mL) and an aqueous 10% NaOH solution (1 mL) was added. The mixture was stirred vigorously for 1 h at room temperature, before it was diluted with $CHCl_3$ and water (5 mL each). The phases were separated and the aqueous phase was extracted with $CHCl_3$ (2 x 5 mL). The combined organic phases were concentrated *in vacuo* to give product **32** (72.5 mg, 0.180 mmol, 90%) as a colourless solid.

^{1}H-NMR ($CDCl_3$, 600 MHz) δ (ppm) 7.81 (dd, *J* = 7.8, 1.6 Hz, 1H), 7.45 (td, *J* = 7.5, 1.1 Hz, 1H), 7.17 (s, 2H), 6.98 (ddd, *J* = 8.6, 7.2, 1.6 Hz, 1H), 6.78 (dd, *J* = 8.4, 1.1 Hz, 1H), 2.59 (s, 3H), 2.58 (s, 6H), 2.42 (s, 3H). ^{13}C-NMR ($CDCl_3$, 151 MHz): δ (ppm) ^{13}C NMR (151 MHz, $CDCl_3$) δ 143.1, 142.8, 138.2, 137.7, 134.7, 130.1, 129.8, 128.2, 127.5, 127.1, 123.2, 113.5, 26.7, 21.4, 13.2. IR (ATR): $\tilde{\nu}$ (cm^{-1}) 3278, 2914, 1673, 1591, 1437, 1295, 1252, 1167, 1028, 985. HR-MS (ESI) Calculated for $C_{18}H_{19}IN_3^+$ $[M\text{-}TfO^-]^+$: *m/z* = 404.06182, found: *m/z* 404.06154. Mp. 184–187°C.

8-Mesityl-2-methyl-8*H*-8λ³-benzo[*d*]pyrazolo[1,5-*b*][1,2]iodazole (33)

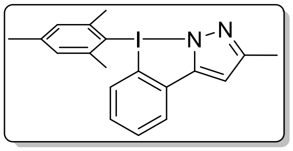

Iodonium salt **24** (110 mg, 0.200 mmol) was suspended in $CHCl_3$ (2 mL) and an aqueous 1 M Na_2CO_3 solution (1 mL) was added. The mixture was stirred vigorously for 4 h at room temperature, before it was diluted with $CHCl_3$ and water (5 mL each). The phases were separated and the aqueous phase was extracted with $CHCl_3$ (2 x 5 mL). The combined organic phases were concentrated *in vacuo* and the residue was washed with hot EtOAc to give product **33** (73.5 mg, 0.158 mmol, 79%) as a yellowish solid.

^{1}H-NMR ($CDCl_3$, 600 MHz) δ (ppm) 7.70 (dd, *J* = 7.7, 1.7 Hz, 1H), 7.32 (t, *J* = 7.4 Hz, 1H), 7.14 (s, 2H), 6.87 (ddd, *J* = 8.6, 7.0, 1.6 Hz, 1H), 6.75 (dd, *J* = 8.3, 1.2 Hz, 1H), 2.58 (s, 6H), 2.43 (s, 3H), 1.49 (s, 3H). ^{13}C-NMR ($CDCl_3$, 151 MHz): δ (ppm) 148.4, 148.2, 142.8, 141.9, 135.8, 129.3, 129.0, 128.9, 126.9, 126.8, 126.6, 113.7, 98.5, 26.7, 21.3, 11.3. IR (ATR): $\tilde{\nu}$ (cm^{-1}) 2950, 2914, 1581, 1439, 1374, 1298, 1125, 998, 963, 852. HR-MS (ESI) Calculated for $C_{17}H_{17}IN_3^+$ $[M-TfO^-]^+$: *m/z* = 403.06657, found: *m/z* 403.06633. Mp. 188–190°C.

Preparation of carboxylic acid stabilized λ³-iodanes 1, 16, 18, 30

Preparation of (2-carboxyphenyl)(hydroxy)iodonium 4-methylbenzenesulfonate 1 from 2-iodosylbenzoic acid and p-toluenesulfonic acid monohydrate [3]: *p*-Toluenesulfonic acid monohydrate (76 mg, 0.4 mmol) was added at room temperature to a stirred mixture of 2-iodosylbenzoic acid **16** (53 mg, 0.2 mmol) with MeCN (1 mL). The reaction was stirred at room temperature for 2 h. After completion of reaction, the solvent was removed under reduced pressure and the solid product was washed with diethyl ether several times then dried in vacuum to give 84 mg (97%) of compound ***1*** as a white solid: mp 115−117 °C; ^{1}H NMR (500 MHz, DMSO-d_6) δ = 8.00 (d, *J* = 7.5 Hz, 1H), 7.94 (t, *J* = 7.5 Hz, 1H), 7.82 (d, *J* = 8.0 Hz, 1H), 7.69 (t, *J* =

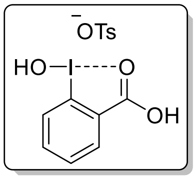

7.5 Hz, 1H), 7.50 (d, J = 7.5 Hz, 2H), 7.13 (d, J = 7.5 Hz, 2H), 2.27 (s, 3H). ^{13}C NMR (100 MHz, DMSO-d$_6$): δ = 167.8, 145.2, 138.0, 134.6, 131.5, 131.1, 130.4, 128.2, 126.4, 125.6, 120.5, 20.8.

Preparation of (1-hydroxy-1λ^3-benzo[d][1,2]iodaoxol-3(1H)-one (16) from 2-iodobenzoic acid [4]:

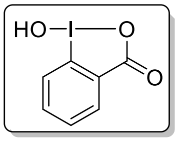

$NaIO_4$ (1.12 g, 5.15 mmol, 1.03 equiv) and 2-iodobenzoic acid (1.24 g, 5.0 mmol, 1.00 equiv) were suspended in AcOH/H_2O (7.5 mL, 1:2 v:v). The mixture was heated under reflux for four hours, diluted with H_2O (20 mL) and then cooled to 25 °C in the dark. After one hour the resulting product was collected by filtration, washed with ice-water (3 × 5 mL) and cold acetone (3 × 5 mL) and then dried open to air in the dark overnight to give **16** (1.25 g, 95%) as a colorless solid: mp.: 174 – 176 °C decomp. ^{1}H-NMR (400 MHz, DMSO-d$_6$): δ = 8.05 (s, 1H), 8.01 (d, J = 7.6 Hz, 1H), 7.96 (t, J = 7.6 Hz, 1H), 7.84 (d, J = 8.0 Hz, 1H), 7.70 (t, J = 7.6 Hz, 1H). ^{13}CNMR (100 MHz, DMSO-d$_6$): δ = 167.8, 134.6, 131.6, 130.5, 126.4, 120.5.

Preparation of (2-carboxyphenyl)(mesityl)iodonium trifluoromethanesulfonate (18) from 2-iodosylbenzoic acid. The compound **18** was prepared according slightly modified reported procedure [5]. Trifluoromethanesulfonic acid (176 µL, 2.0 mmol) was added at room temperature to a stirred mixture of 2-iodosylbenzoic acid **16** (264 mg, 1.0 mmol) with TFE (3 mL). The reaction was stirred at room temperature for 10 min, then mesitylene (278 µL, 2.0 mmol) was added. After completion of reaction, the solvent was removed under reduced pressure and the solid product was washed with diethyl ether several times then dried in vacuum to give 469 mg (91%) of compound **18** as a white solid: mp 206−207 °C decomp; ^{1}H NMR (400 MHz, DMSO-d$_6$) δ = 8.29 (dd, J = 7.6, 1.6 Hz, 1H), 7.75 (td, J = 7.2, 0.8 Hz, 1H), 7.68 (td, J = 7.2, 2.0 Hz, 1H), 7.37 (s, 2H), 6.80 (dd, J = 8.0, 0.8 Hz, 1H), 2.50 (s, 6H), 2.41 (s, 3H). ^{13}C NMR (100 MHz, DMSO-d$_6$): δ = 168.7, 144.0, 143.2, 136.4, 132.9, 131.2, 129.9, 129.8, 127.6, 120.69 (q, J_{CF} = 320 Hz, $CF_3SO_3^-$), 118.5, 113.6, 26.0, 20.8; ^{19}F NMR (376 MHz, DMSO-d6): δ = -77.8.

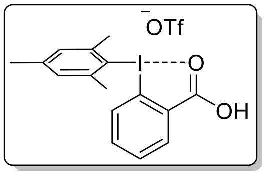

Preparation of 1-Mesityl-1λ³-benzo[d][1,2]iodaoxol-3(1H)-one (30). The compound **30** was prepared by deprotonation of compound **18** according to followed procedure. (2-Carboxyphenyl)(mesityl)iodonium trifluoromethane-sulfonate **18** (103 mg, 0.2 mmol, 1 equiv) was dissolved in methanol until the formation of a clear solution (1.5 mL of MeOH). An aqueous solution of sodium carbonate (0.2 mL of solution containing 25 mg, 0.24 mmol, 2.2 equiv of Na_2CO_3) was added to the solution under vigorous stirring. The stirring was continued for 2 h. The methanol was removed using rotary evaporation and water (3 mL) was added. The stirring was continued for 15 min. The solid was filtered off, washed by water (2 × 1.5 mL), and dried under vacuum to give 71.7 mg (98%) of compound **30** as a white solid: mp 221−222 °C decomp (lit. [6] mp 223−223.5 °C); ^{1}H NMR (400 MHz, DMSO-d_6) δ = 8.16 (dd, *J* = 7.6, 1.6 Hz, 1H), 7.62 (td, *J* = 7.6, 0.4 Hz, 1H), 7.50 (td, *J* = 7.2, 1.6 Hz, 1H), 7.27 (s, 2H), 6.65 (d, *J* = 7.6 Hz, 1H), 2.45 (s, 6H), 2.38 (s, 3H). ^{13}C NMR (100 MHz, DMSO-d_6): δ = 165.6, 142.8, 142.7, 135.3, 133.7, 131.8, 130.5, 129.3, 125.6, 121.1, 113.6, 25.7, 20.8.

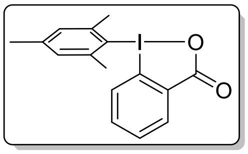

Decomposition studies

Table S1: Decomposition temperatures and enthalpies of compounds **1–15**[a]

Compound	Decomposition temperature (T_{peak}), °C	Decomposition enthalpy (ΔH_{dec}), kJ/mol	Decomposition enthalpy (ΔH_{dec}), J/g
**1**	206.8	72.93	167.2
**2**	120.8	116.28	253.6
**3**	143.6	125.11	264.3
**4**	152.4	88.76	187.3
**5**	124.6	134.76	293.0
**6**	168.9	2.52	5.3
**7**	196.5	50.78	111.3
**8**	148.9	51.46	101.4
**9**	198.8	76.36	150.3
**10**	210.1	58.47	109.5
**11**	193.9	64.81	118.9
**12**	172.3	44.94	85.7
**13**	159.2	73.52	144.1
**14**	144.0	71.34	117.0
**15**	118.9	39.70	65.2
**16**[b]	267.3	65.03	254.9
**17**	195.7	134.49	468.8

[a]Temperature interval from 25 °C to 260 °C, heating speed 10 °C /min, nitrogen stream (20 mL/min), Al crucible. [b]Temperature interval from 25 °C to 335 °C, heating speed 10 °C /min, nitrogen stream (100 mL/min), Al_2O_3 crucible.

Table S2: Decomposition temperatures and enthalpies of compounds **18**–**33**[a]

Compound	Decomposition temperature (T_{peak}), °C	Decomposition enthalpy (ΔH_{dec}), kJ/mol	Decomposition enthalpy (ΔH_{dec}), J/g
**18**	202.1	10.19	19.8
**19**	158.9	23.37	43.3
**20**	158.3	13.18	23.7
**21**	180.0	46.50	86.1
**22**	216.8	-29.81	-54.1
**23**	199.4	21.85	40.8
**24**	191.8	86.01	156.4
**25**	180.6	4.70	8.0
**26**	219.5	32.98	53.7
**27**	227.6	37.67	61.2
**28**	205.7	51.80	87.8
**29**	200.6	24.59	35.6
**30**	227.3	85.85	233.3
**31**	226.9	98.34	252.8
**32**	210.8	141.13	349.6
**33**	201.1	122.66	305.9

[a]Temperature interval from 25 °C to 260 °C, heating speed 10 °C /min, nitrogen stream (20 mL/min), Al crucible.

Thermal decomposition of (2-(1H-benzo[d]imidazol-2-yl)phenyl)(mesityl)iodonium trifluoro-methanesulfonate (25).

The pseudocyclic salt **25** (20 mg) has been added to a Schlenk tube under argon atmosphere and slowly heated up to 185 ^{o}C during 1 hour. After 10 minutes the melted solid has been cooled to room temperature. The resulting oil has been dissolved on EtOAc and analysed by GC–MS.

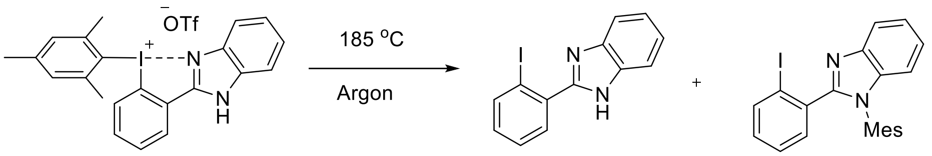

25 **25a** **25b**

Thermal decomposition of 8-mesityl-2-methyl-8H-8λ^3-benzo[d]pyrazolo[1,5-b][1,2]iodazole (33)

Compound **33** (5.7 mg) was slowly heated to 210 °C under a nitrogen atmosphere. After 10 minutes the oily residue was dissolved in chloroform and analyzed via TLC–MS. The TLC showed 4 spots, with the two main spots corresponding to the masses of the N-mesitylated products **33a** and **33b**. The masses of the two lesser concentrated spots proposedly correspond to N-I-coupling products **33c** and **33d** with the latter one in only very low concentration.

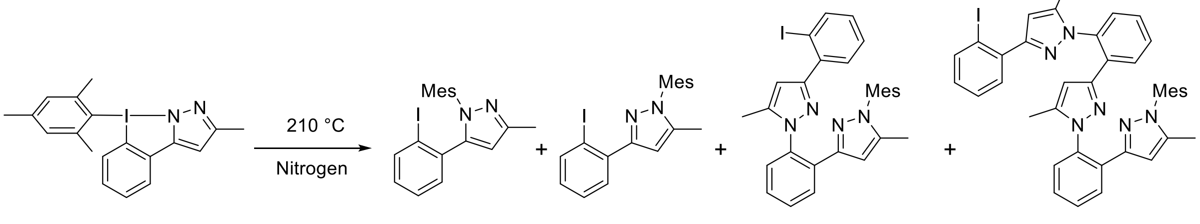

33 **33a** **33b** **33c** **33d**

Literature

1. Boelke, A.; Lork, E.; Nachtsheim, B. J. *Chem. Eur. J.* **2018,** *24*, 18653–18657.
2. Vlasenko, Y. A.; Postnikov, P. S.; Trusova, M. E.; Shafir, A.; Zhdankin, V. V.; Yoshimura, A.; Yusubov, M. S. *J. Org. Chem.* **2018,** *83*, 12056–12070.
3. Yoshimura, A.; Klasen, S. C.; Shea, M. T.; Nguyen, K. C.; Rohde, G. T.; Saito, A.; Postnikov, P. S.; Yusubov, M. S.; Nemykin, V. N.; Zhdankin, V. V. *Chem. Eur. J.* **2017**, *23*, 691–695.
4. D. Göbel, N. Clamor, B. J. Nachtsheim, *Org. Biomol. Chem*., **2018**,16, 4071-4075.
5. Yoshimura, A.; Nguyen, K. C.; Klasen, S. C.; Saito, A.; Nemykin, V. N.; Zhdankin, V. V. *Chem. Commun.* **2015**, *51*, 7835–7838.
6. Yusubov, M. S.; Yusubova, R. Y.; Nemykin, V. N.; Zhdankin, V. V. *J. Org. Chem.* **2013**, *78*, 3767–3773.

TGA/DSC-Curves

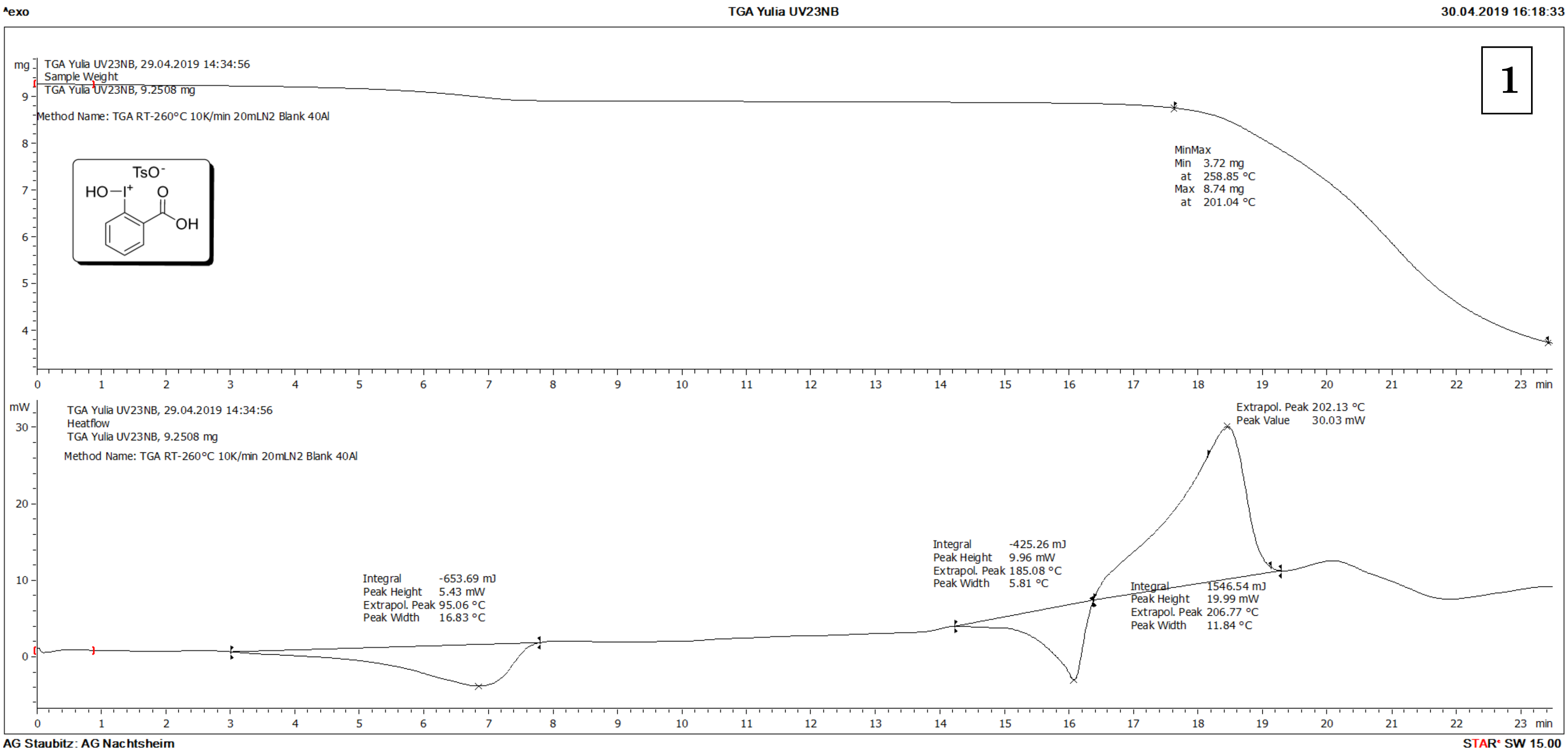

Figure S1: TGA-DSC curve for (2-carboxyphenyl)(hydroxy)iodonium 4-methylbenzenesulfonate (**1**) at heating rate 10 °C/min.

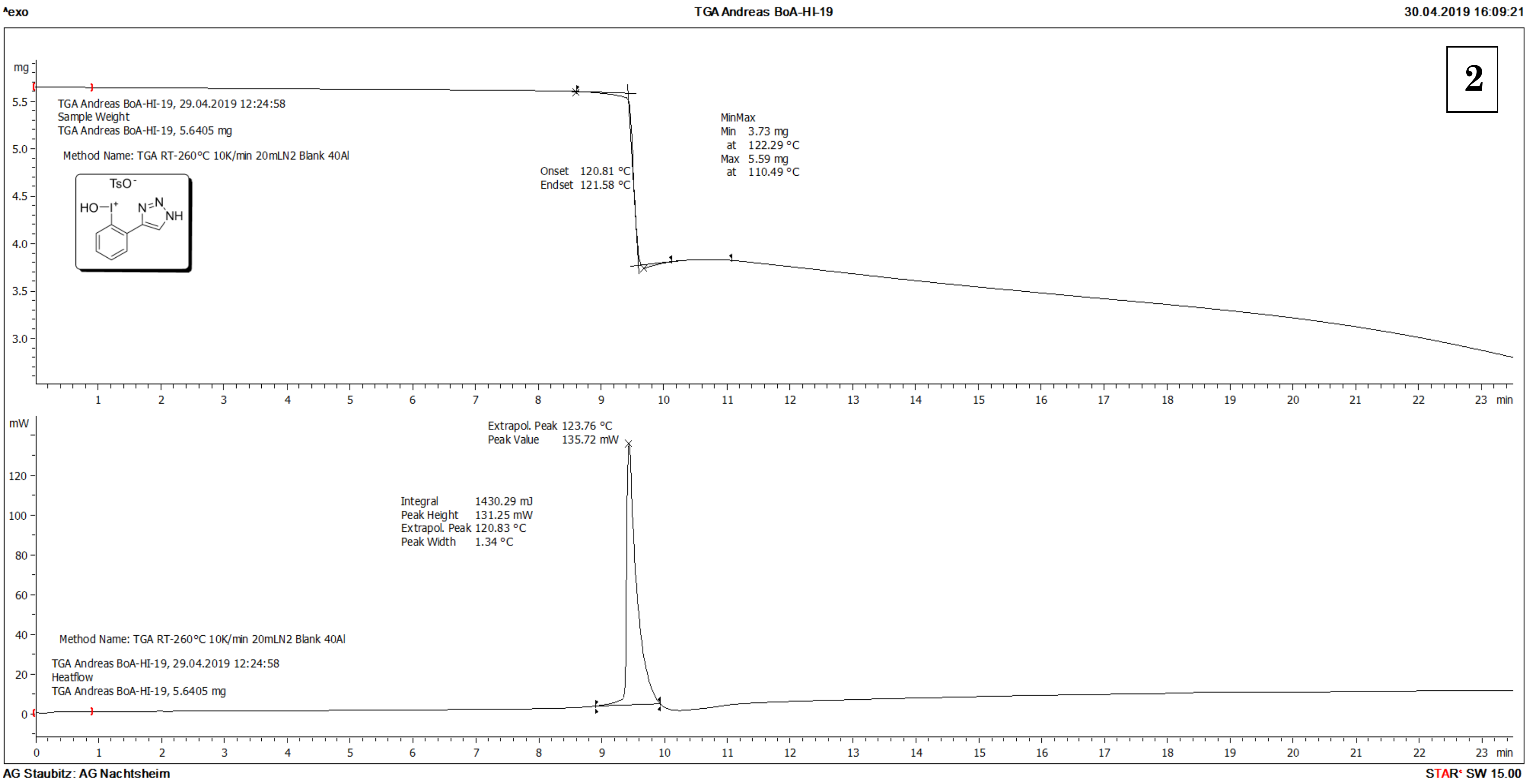

Figure S2: TGA-DSC curve for (2-(1*H*-1,2,3-triazol-4-yl)phenyl)(hydroxy)iodonium 4-methylbenzenesulfonate (**2**) at heating rate 10 °C/min.

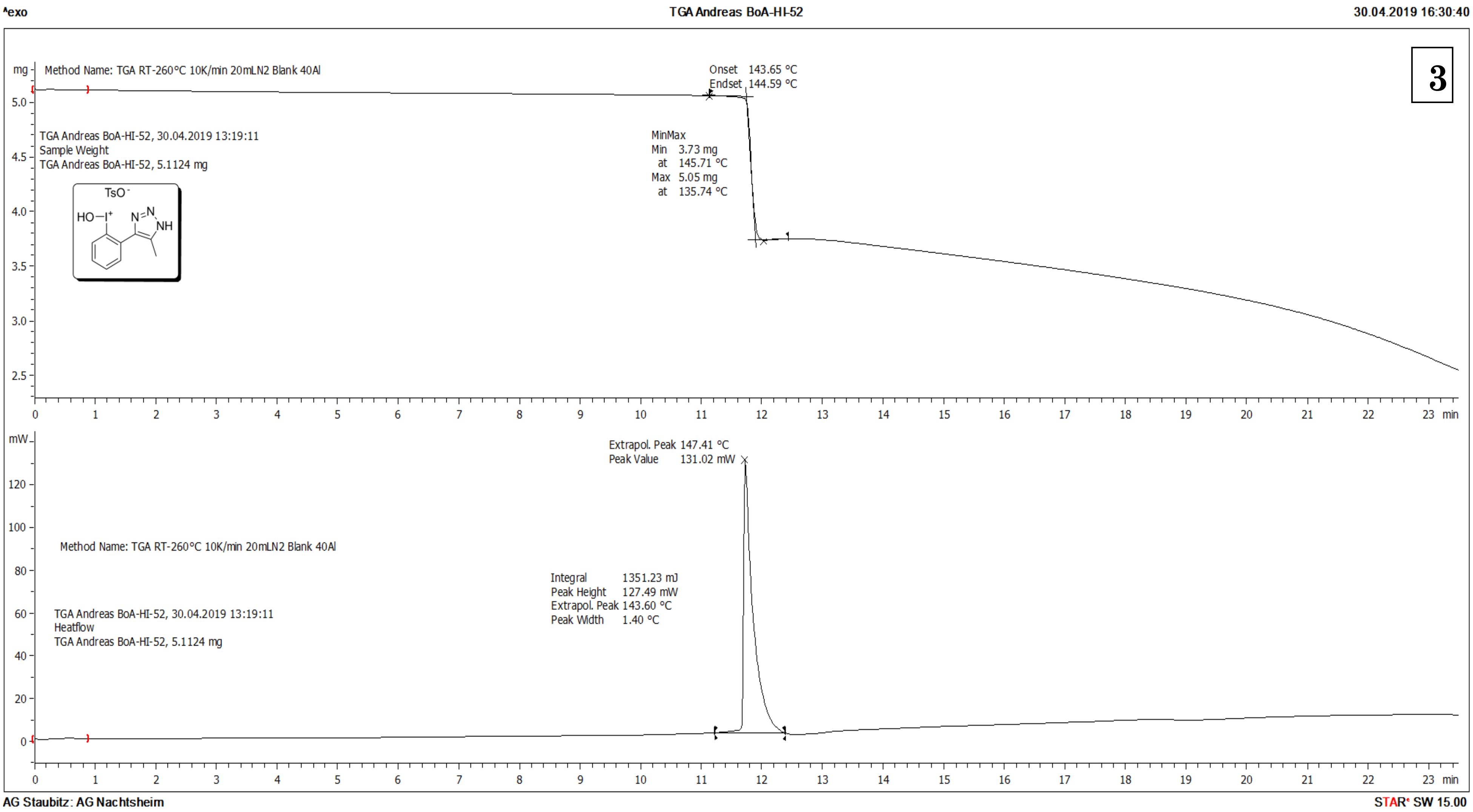

Figure S3: TGA-DSC curve for hydroxy(2-(5-methyl-1*H*-1,2,3-triazol-4-yl)phenyl)iodonium 4-methylbenzenesulfonate (**3**) at heating rate 10 °C/min.

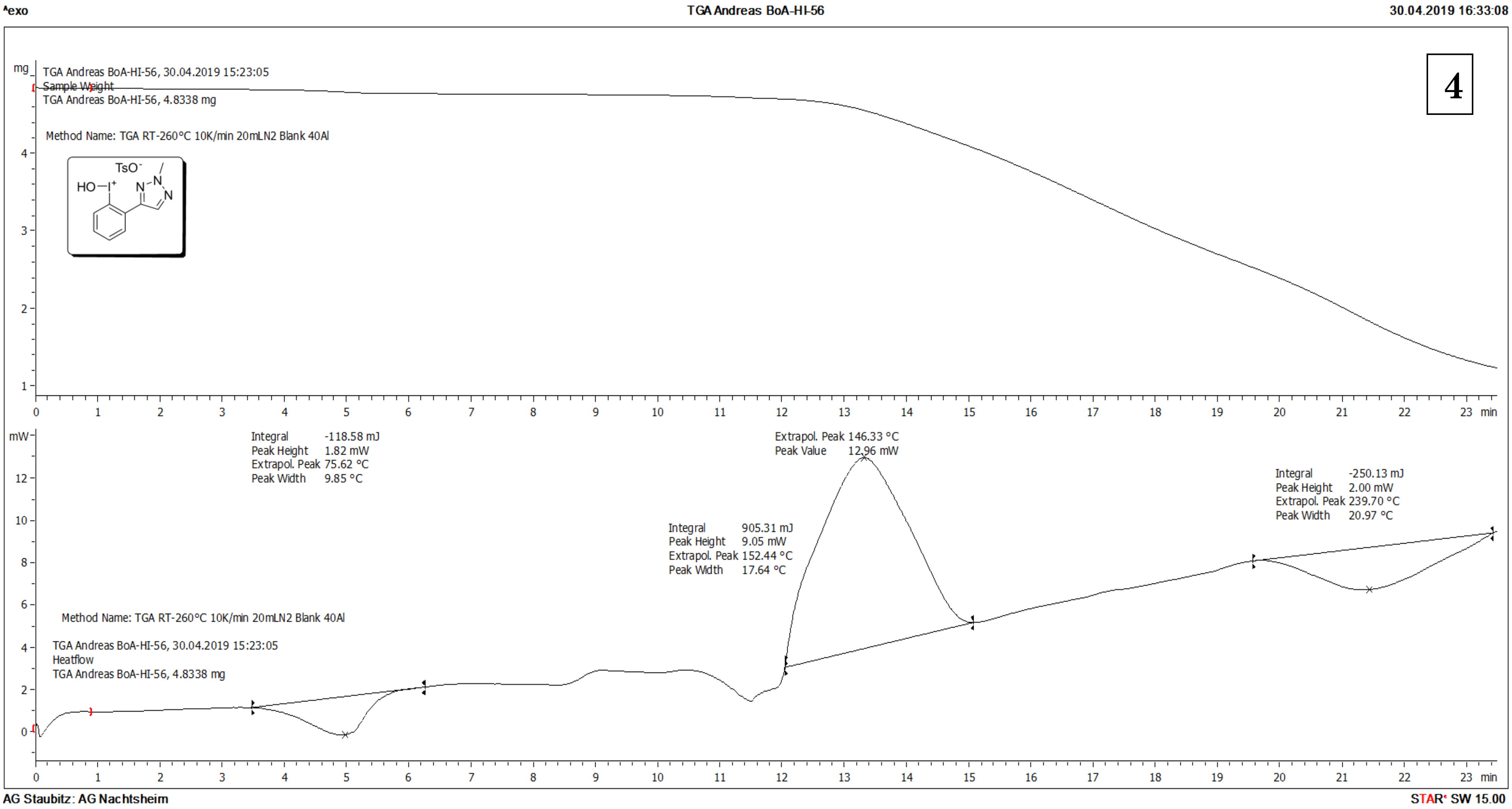

Figure S4: TGA-DSC curve for hydroxy(2-(2-methyl-2*H*-1,2,3-triazol-4-yl)phenyl)iodonium 4-methylbenzenesulfonate (**4**) at heating rate 10 °C/min.

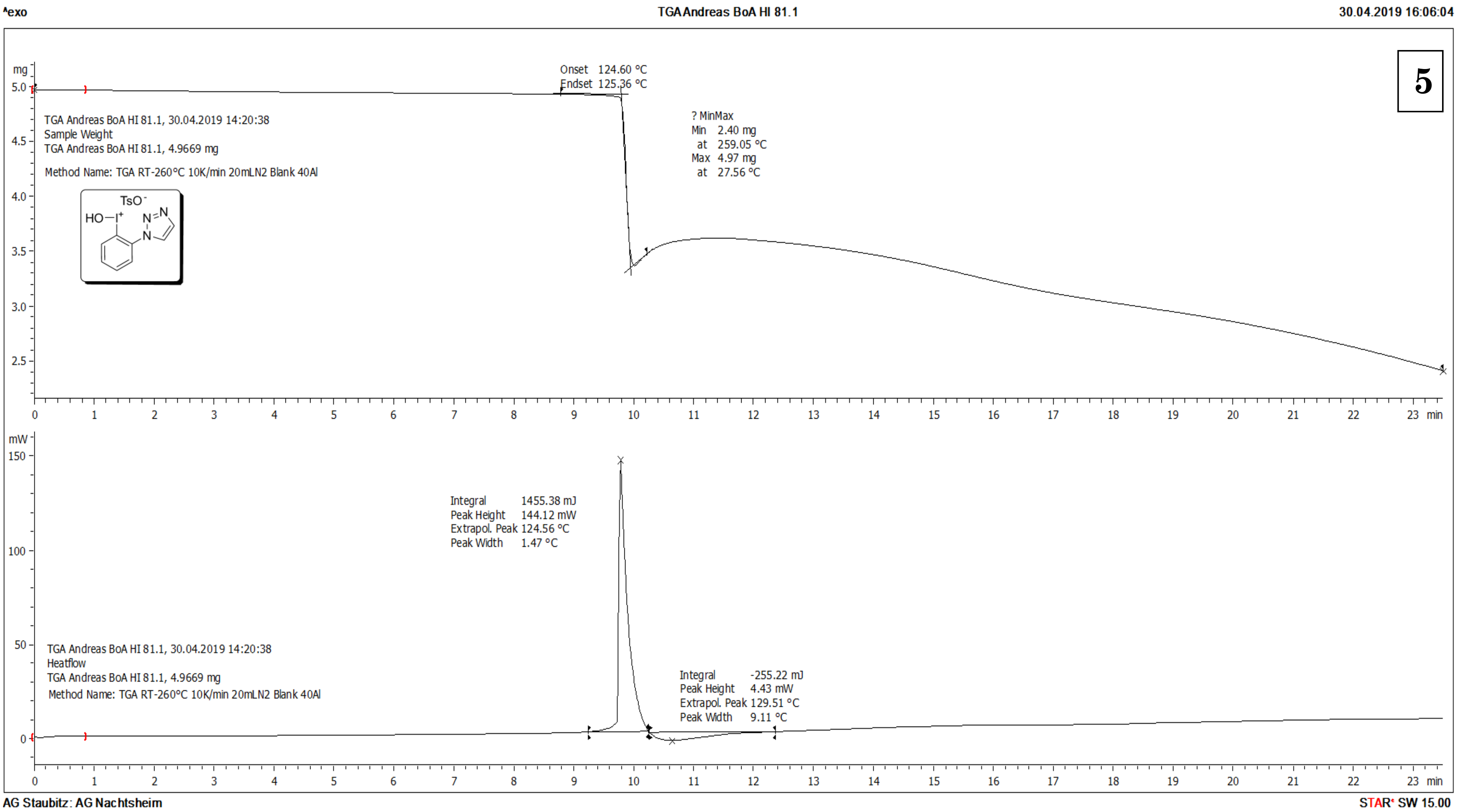

Figure S5: TGA-DSC curve for (2-(1*H*-1,2,3-triazol-1-yl)phenyl)(hydroxy)iodonium 4-methylbenzenesulfonate (**5**) at heating rate 10 °C/min.

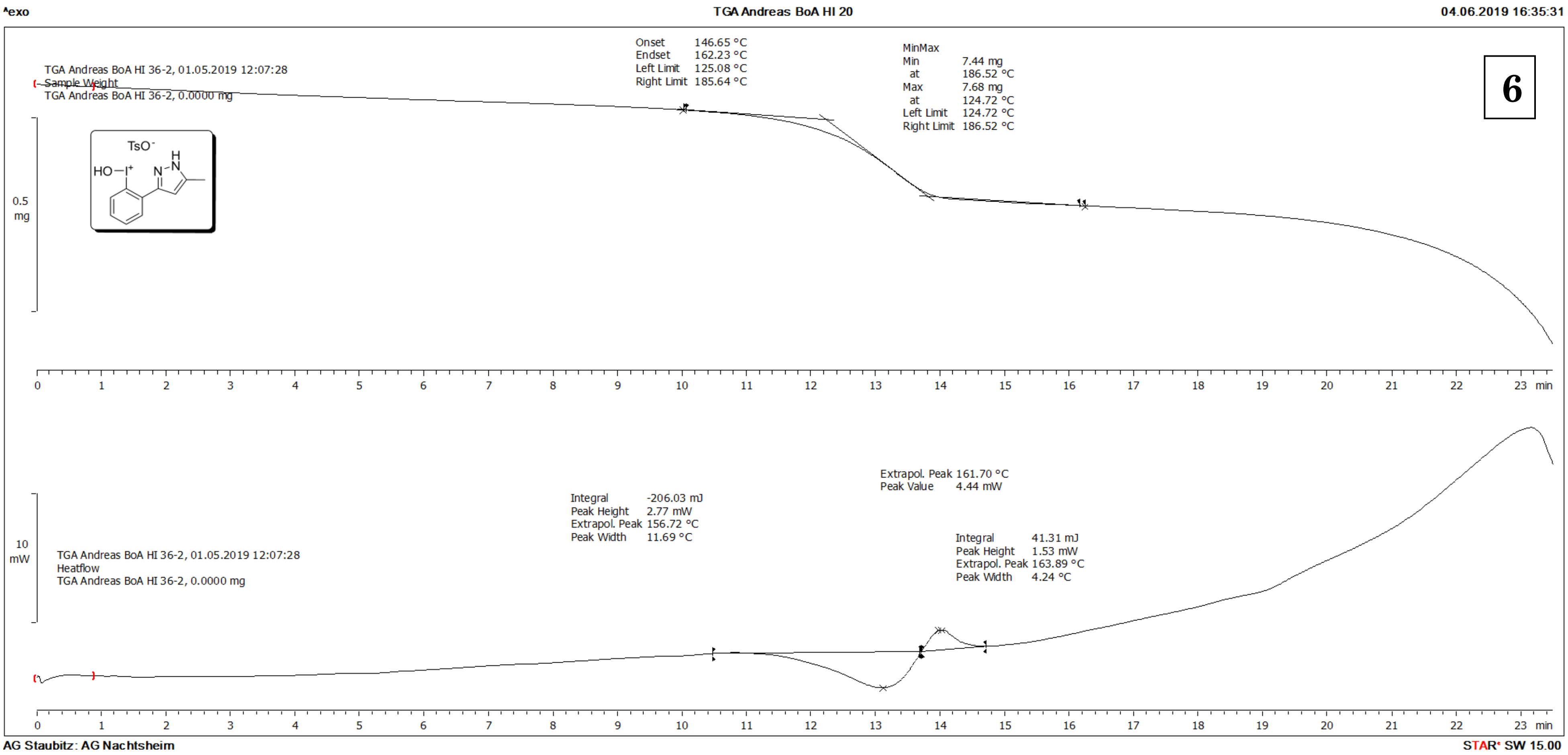

Figure S6: TGA-DSC curve for hydroxy(2-(5-methyl-1*H*-pyrazol-3-yl)phenyl)iodonium 4-methylbenzenesulfonate (**6**) at heating rate 10 °C/min.

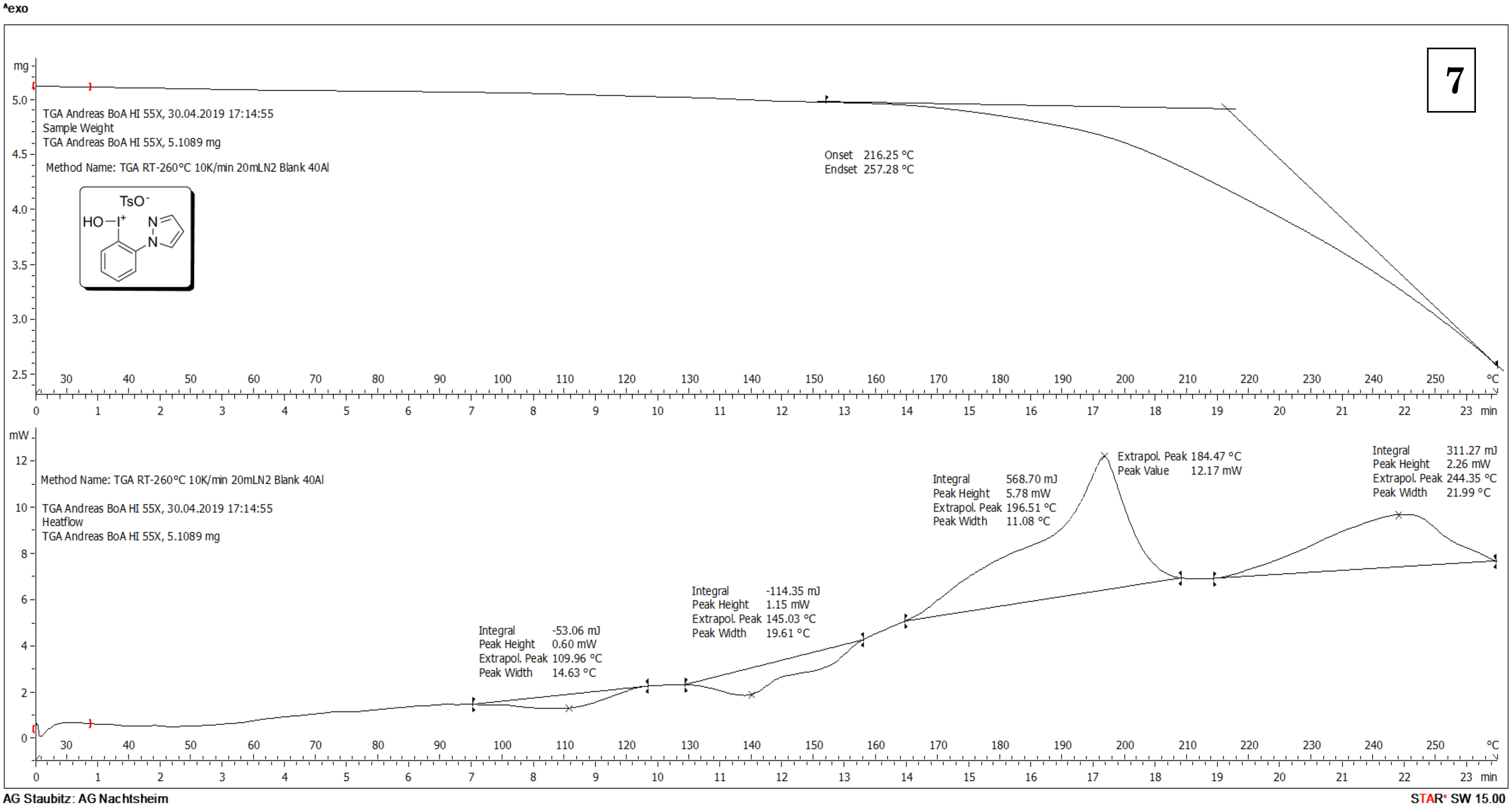

Figure S7: TGA-DSC curve for (2-(1*H*-pyrazol-1-yl)phenyl)(hydroxy)iodonium 4-methylbenzenesulfonate (**7**) at heating rate 10 °C/min.

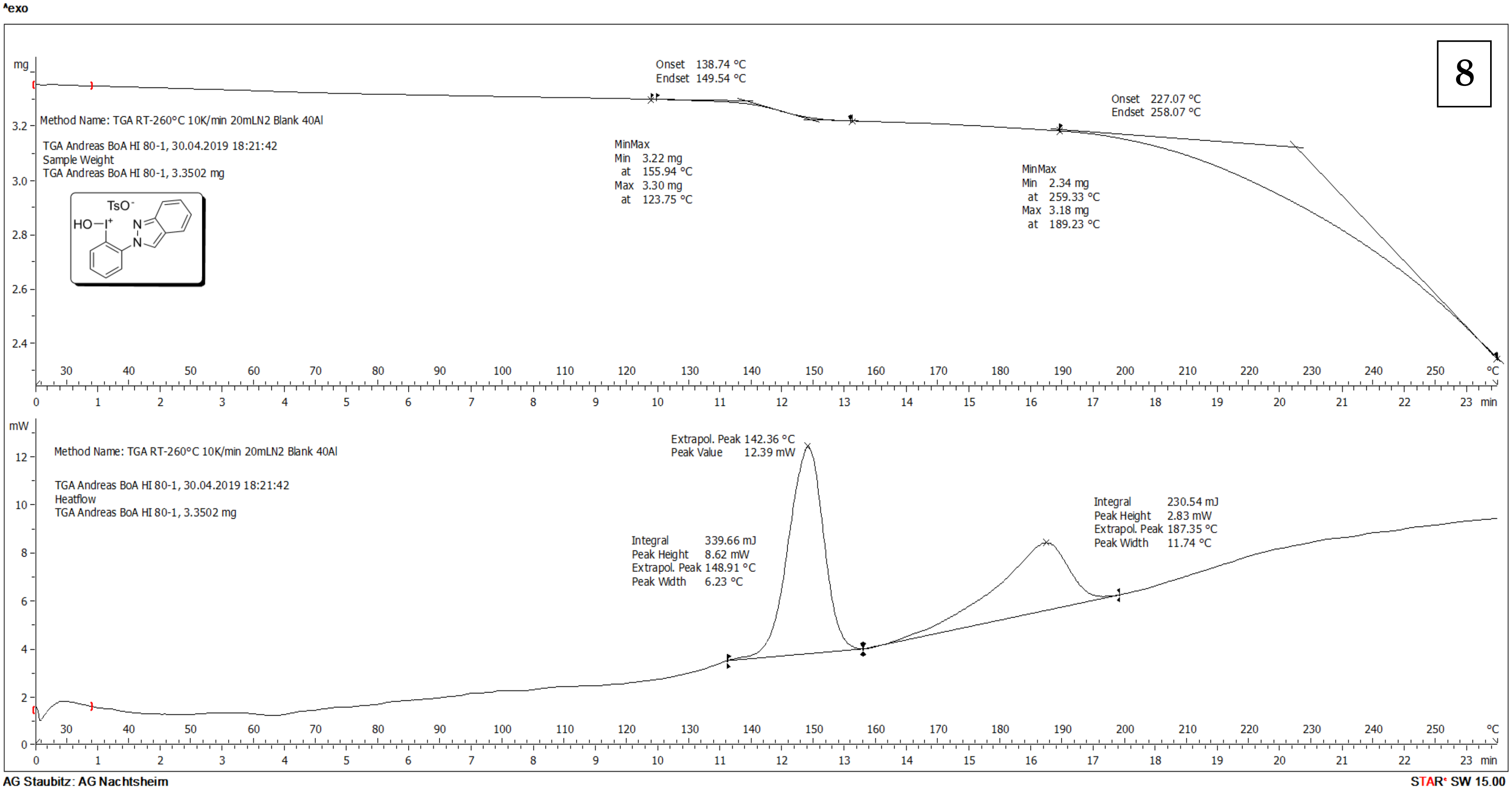

Figure S8: TGA-DSC curve for (2-(2*H*-indazol-2-yl)phenyl)(hydroxy)iodonium 4-methylbenzenesulfonate (**8**) at heating rate 10 °C/min.

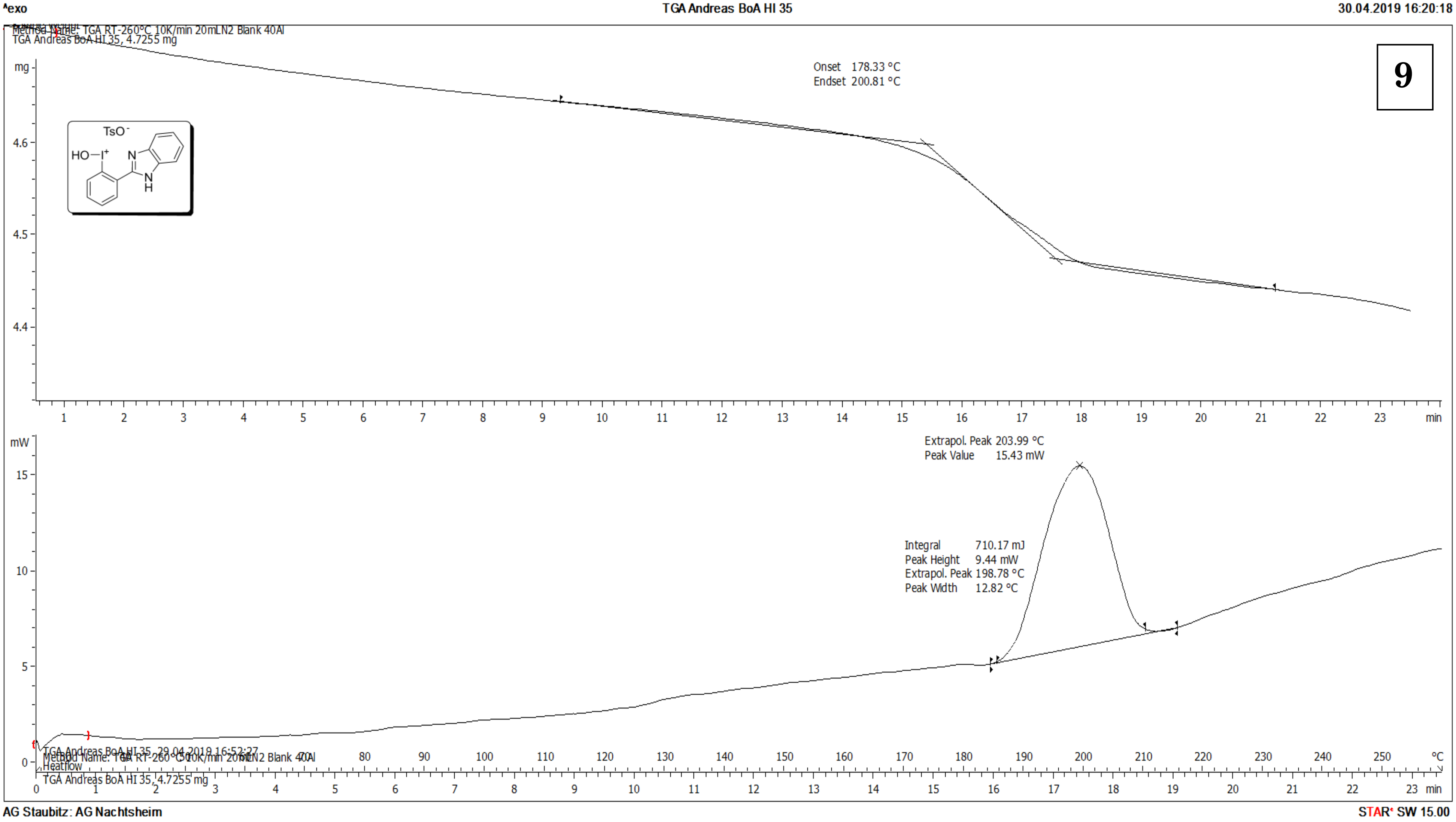

Figure S9: TGA-DSC curve for (2-(1*H*-benzo[*d*]imidazol-2-yl)phenyl)(hydroxy)iodonium 4-methylbenzenesulfonate (**9**) at heating rate 10 °C/min.

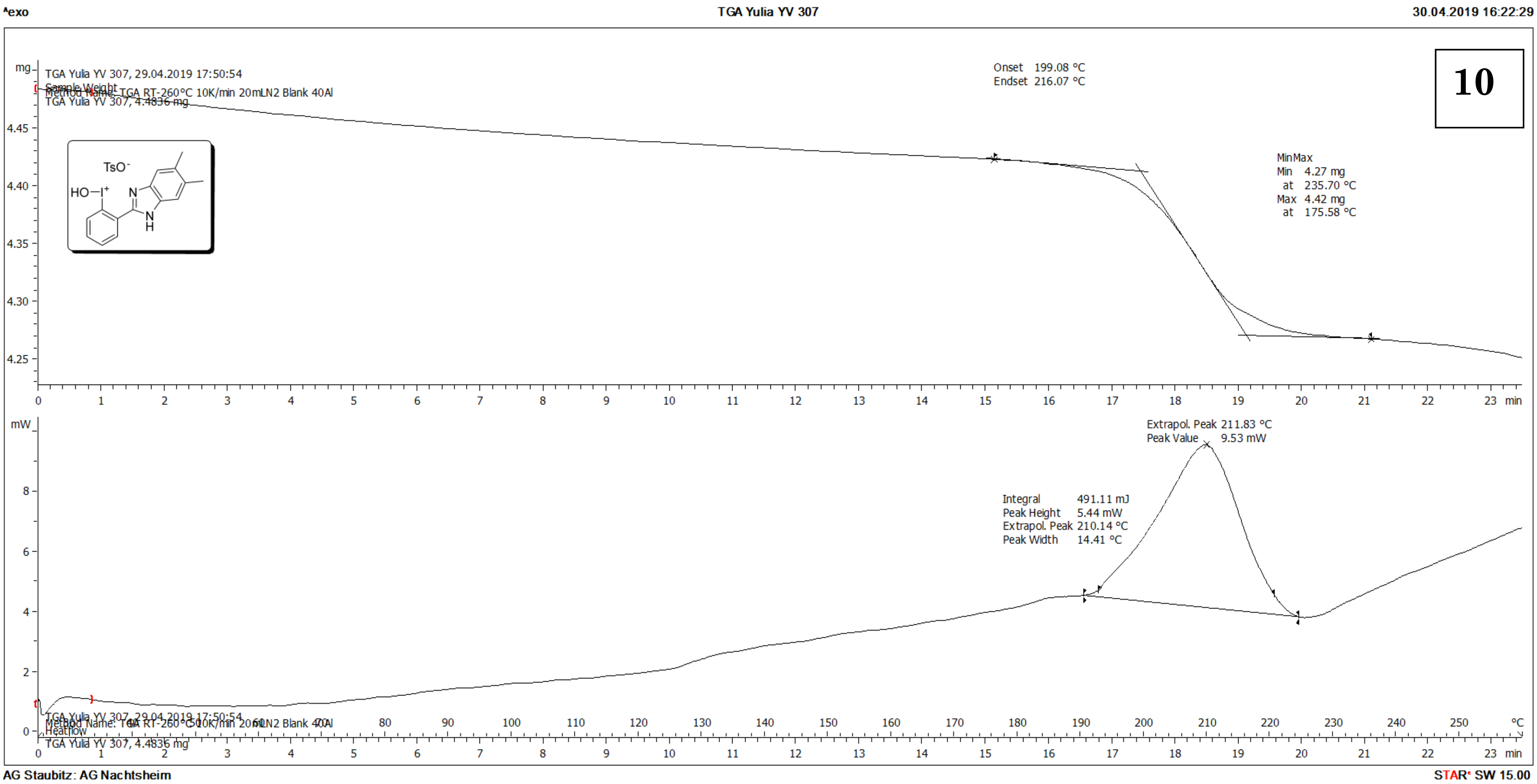

Figure S10: TGA-DSC curve for (2-(5,6-dimethyl-1*H*-benzo[*d*]imidazol-2-yl)phenyl)(hydroxy)iodonium 4-methylbenzenesulfonate (**10**) at heating rate 10 °C/min.

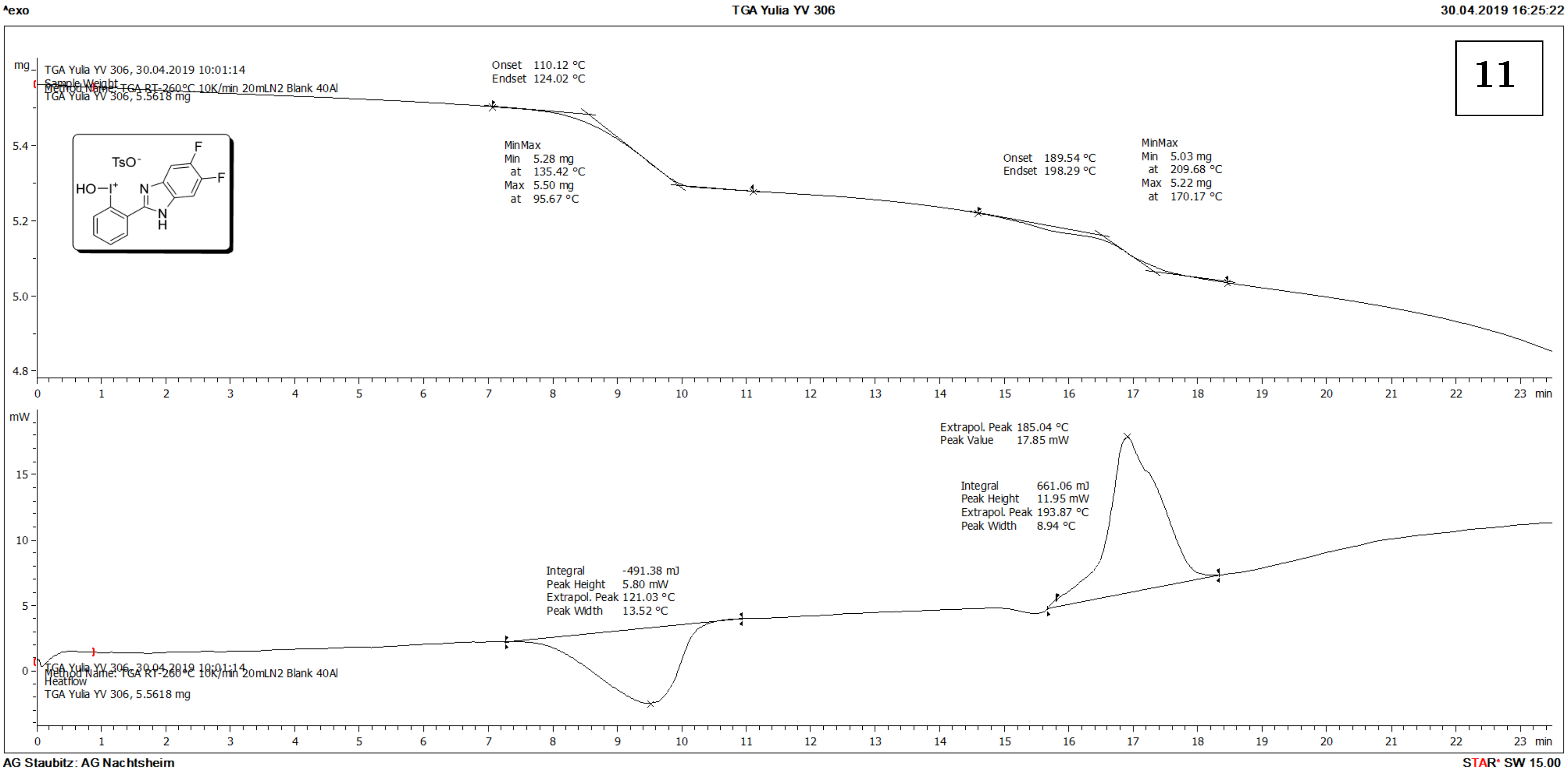

Figure S11: TGA-DSC curve for (2-(5,6-difluoro-1*H*-benzo[*d*]imidazol-2-yl)phenyl)(hydroxy)iodonium 4-methylbenzenesulfonate (**11**) at heating rate 10 °C/min.

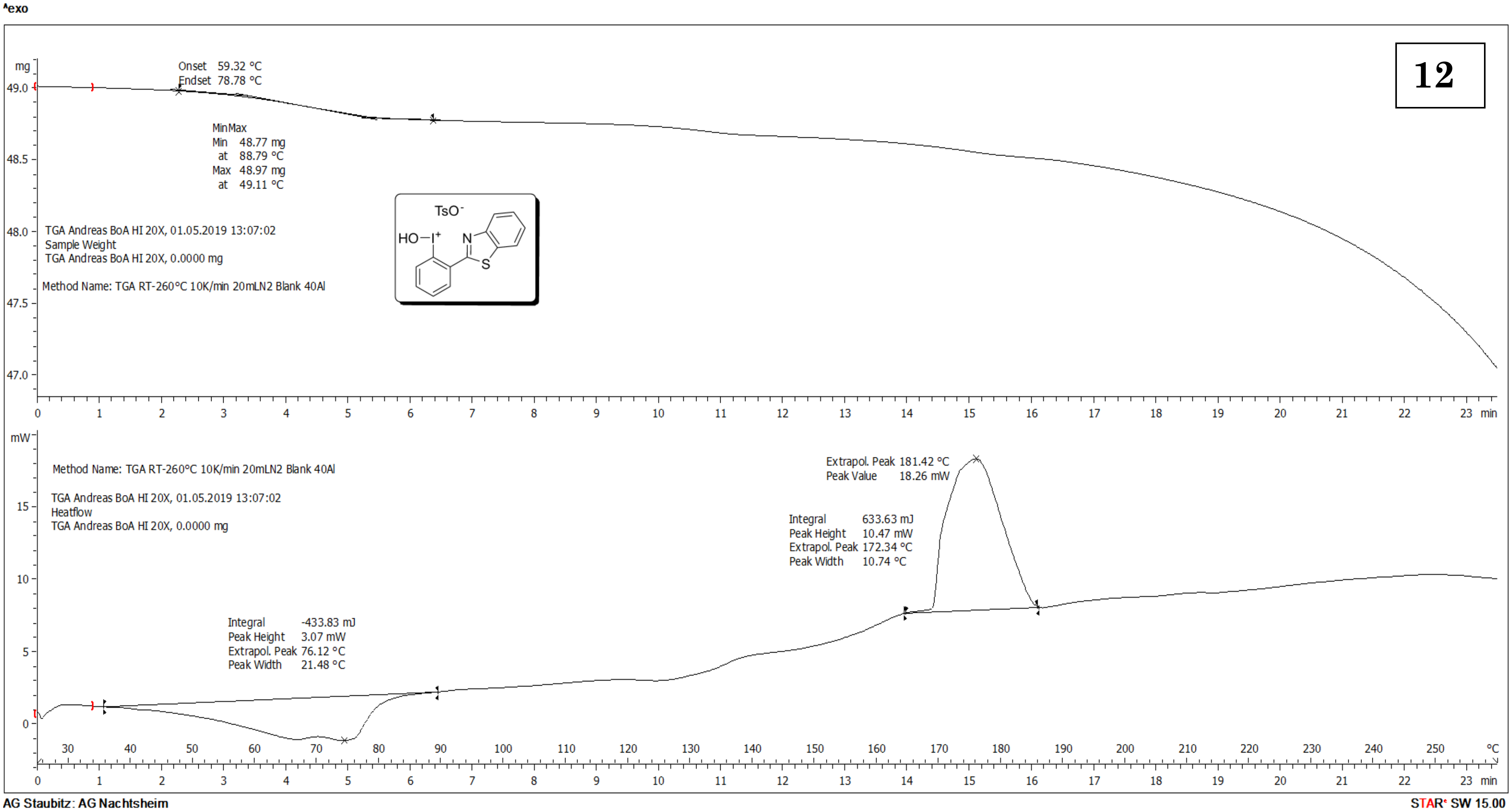

Figure S12: TGA-DSC curve for (2-(benzo[*d*]thiazol-2-yl)phenyl)(hydroxy)iodonium 4-methylbenzenesulfonate (**12**) at heating rate 10 °C/min.

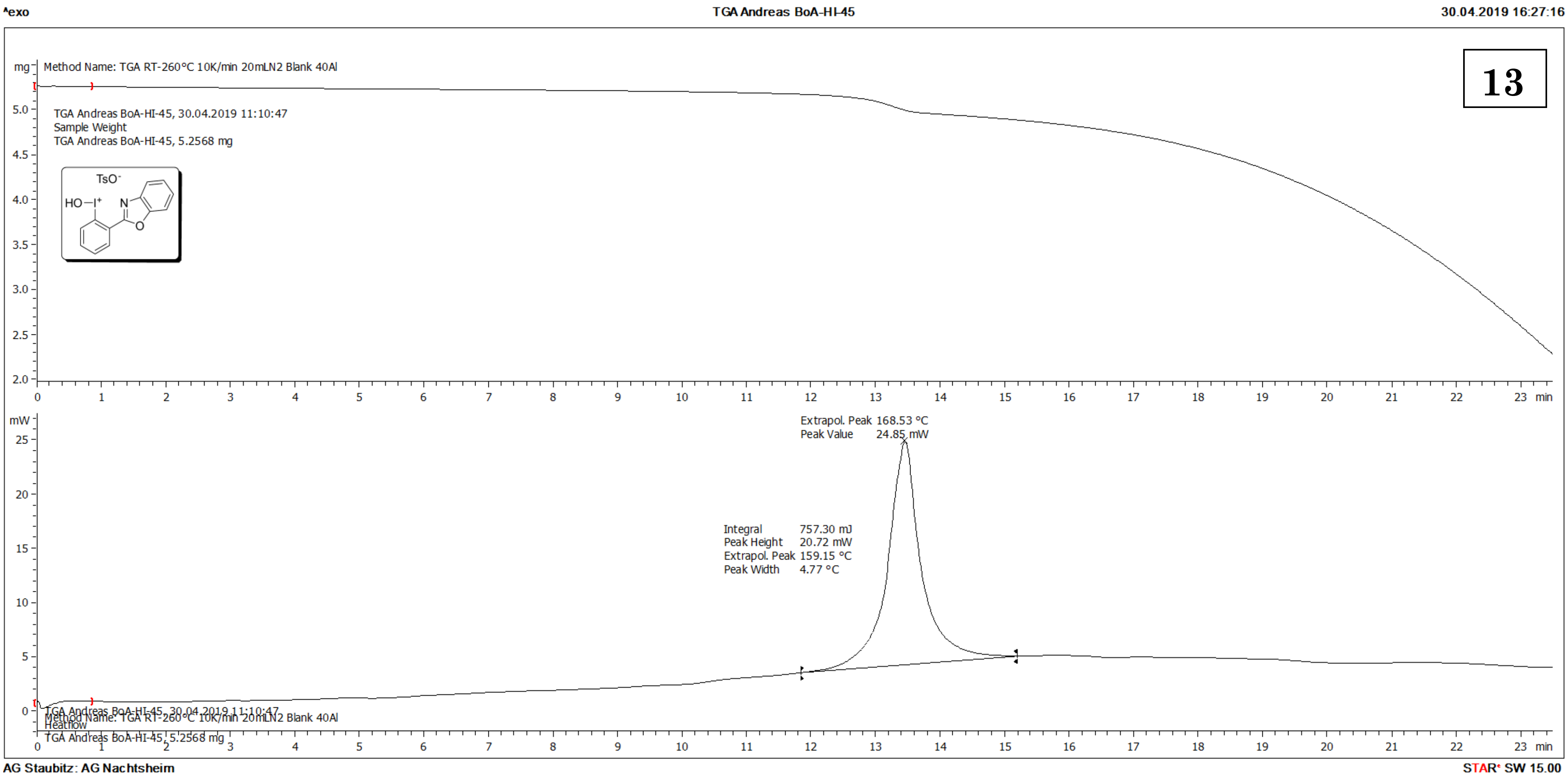

Figure S13: TGA-DSC curve for (2-(benzo[*d*]oxazol-2-yl)phenyl)(hydroxy)iodonium 4-methylbenzenesulfonate (**13**) at heating rate 10 °C/min.

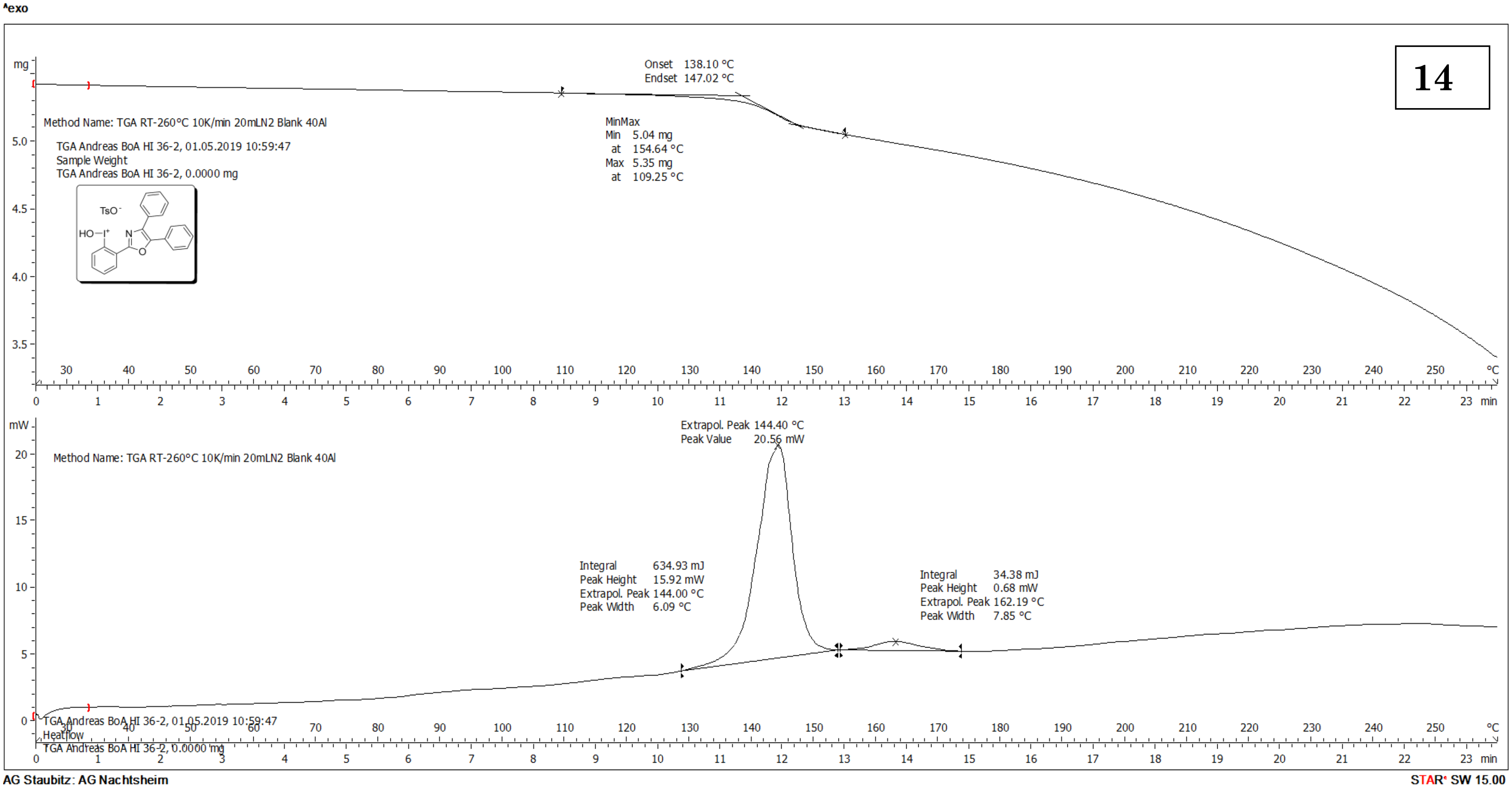

Figure S14: TGA-DSC curve for (2-(4,5-diphenyloxazol-2-yl)phenyl)(hydroxy)iodonium 4-methylbenzenesulfonate (**14**) at heating rate 10 °C/min.

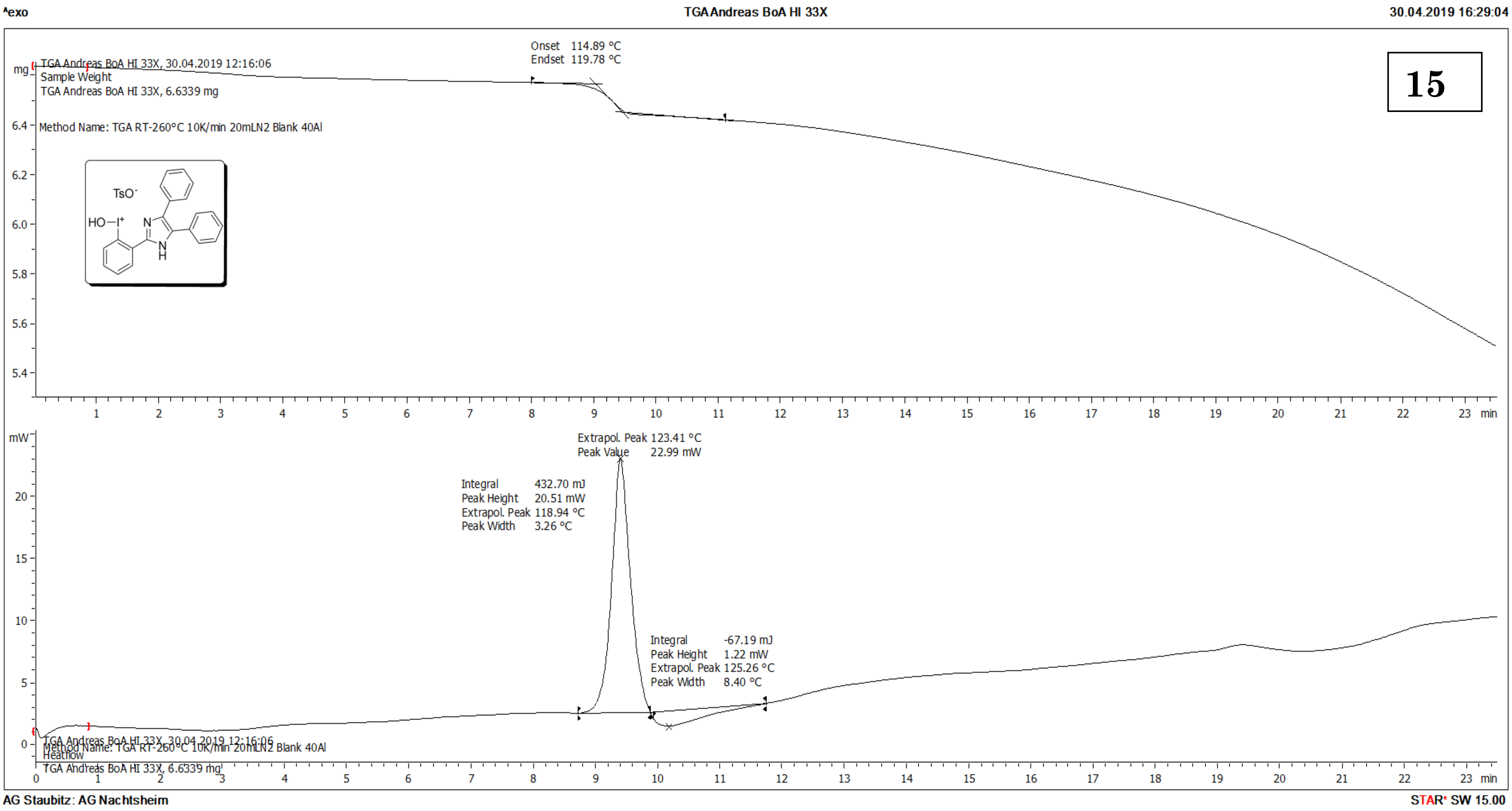

Figure S15: TGA-DSC curve for (2-(4,5-diphenyl-1*H*-imidazol-2-yl)phenyl)(hydroxy)iodonium 4-methylbenzenesulfonate (**15**) at heating rate 10 °C/min.

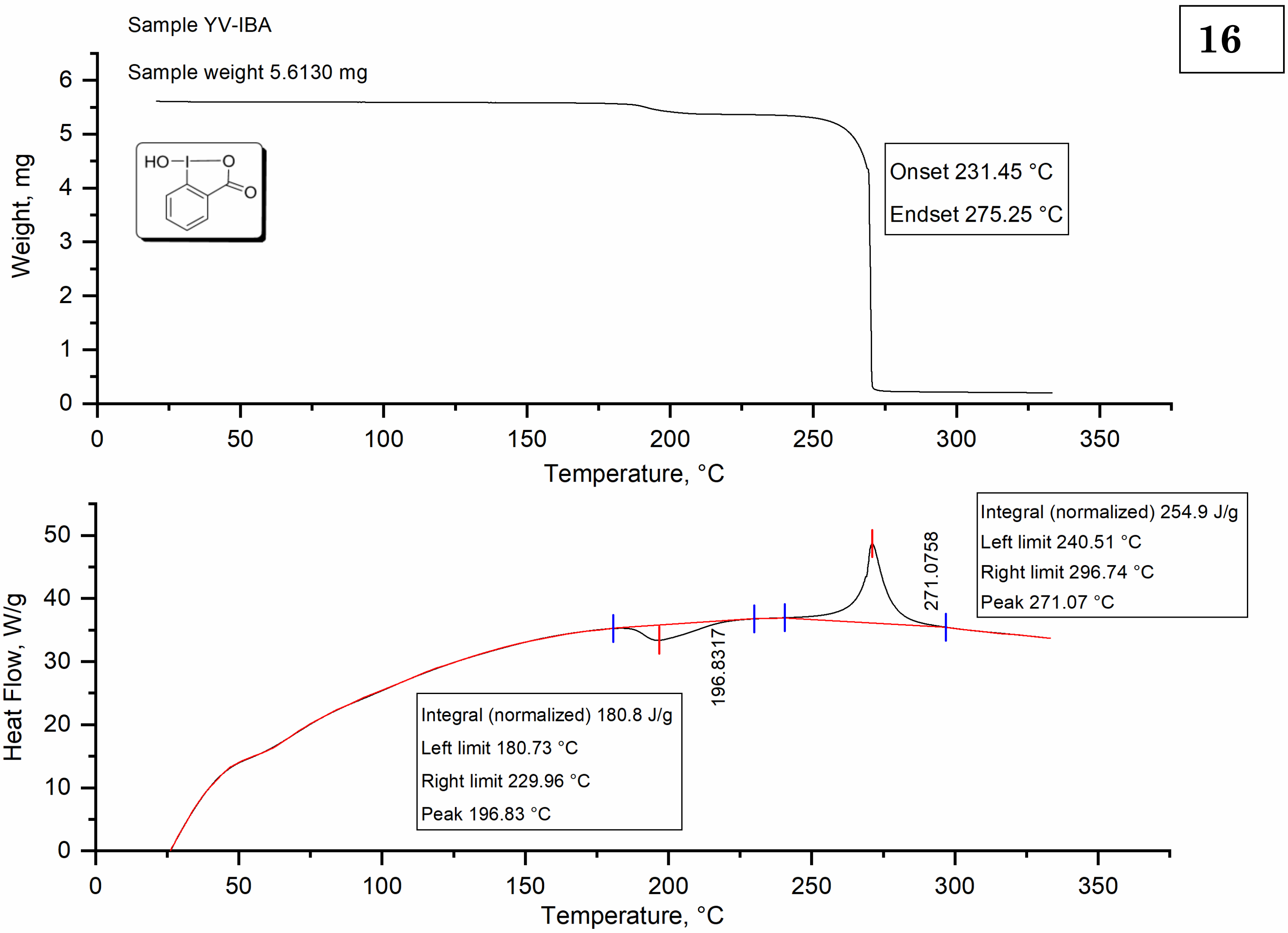

Figure S16: TGA-DSC curve for 1-hydroxy-1λ^3-benzo[*d*][1,2]iodaoxol-3(1*H*)-one (**16**) at heating rate 10 °C/min.

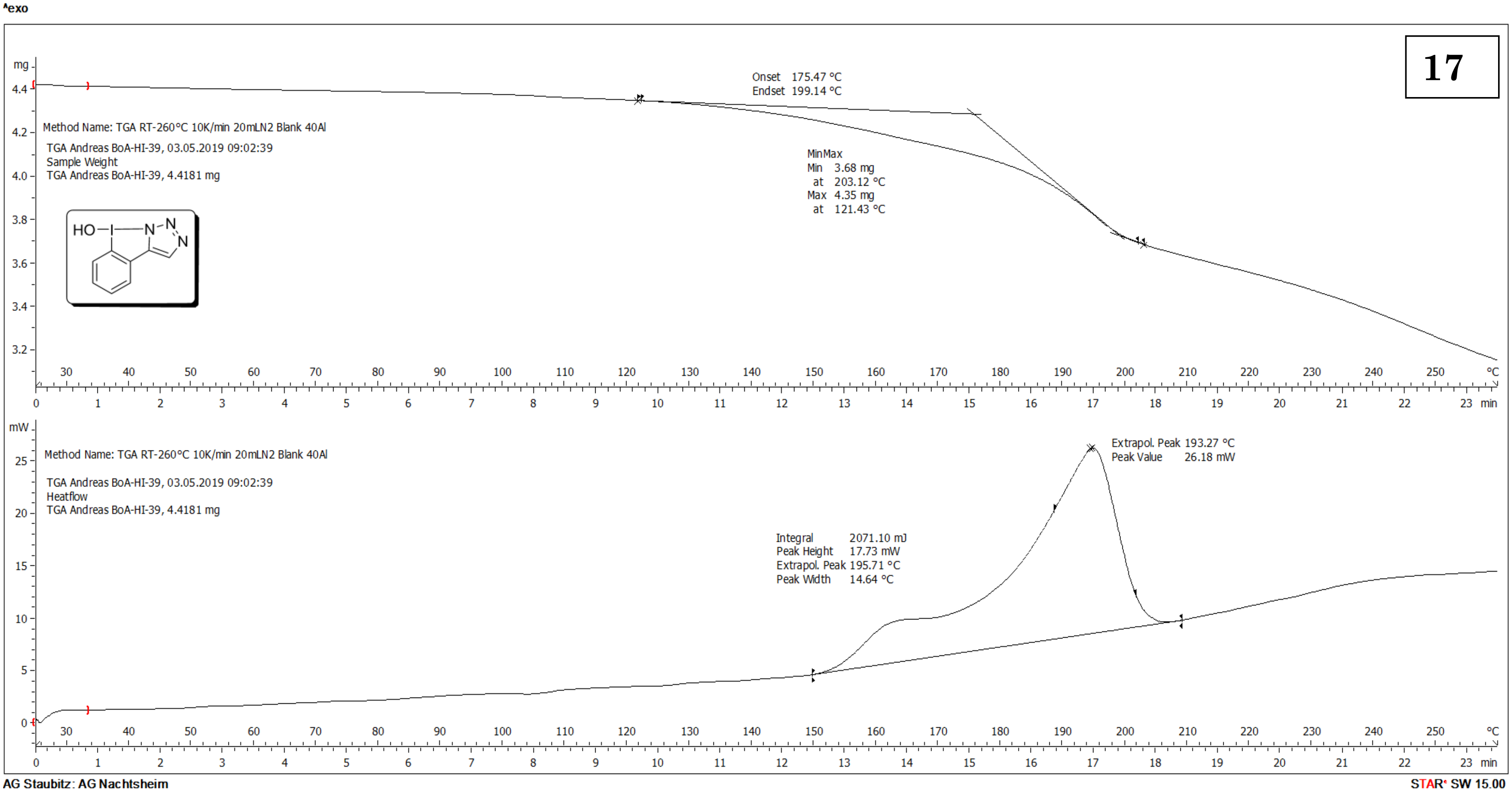

Figure S17: TGA-DSC curve for 8*H*-8λ^3-benzo[4,5][1,2]iodazolo[2,3-*c*][1,2,3]triazol-8-ol (**17**) at heating rate 10 °C/min.

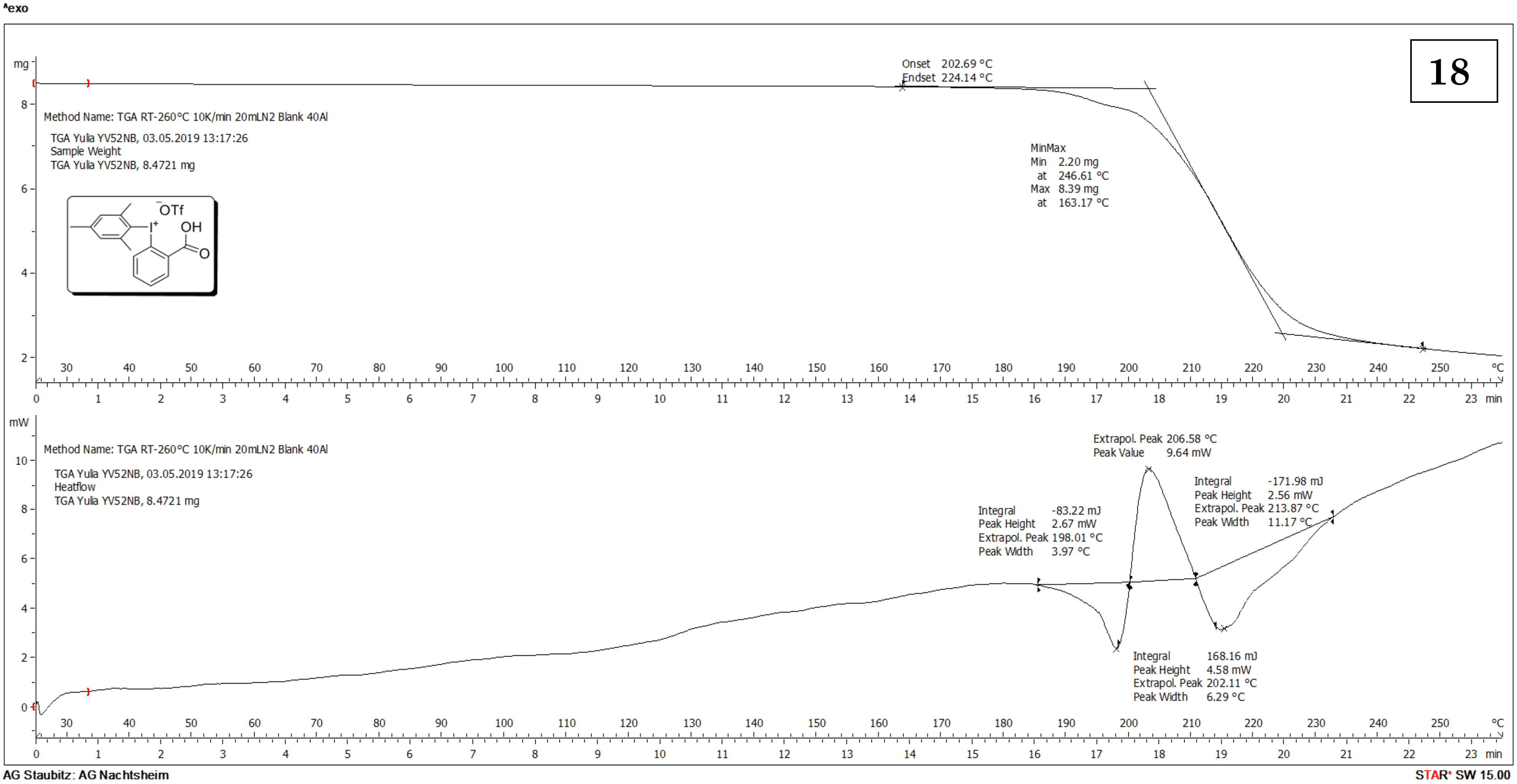

Figure S18: TGA-DSC curve for (2-carboxyphenyl)(mesityl)iodonium trifluoromethanesulfonate (**18**) at heating rate 10 °C/min.

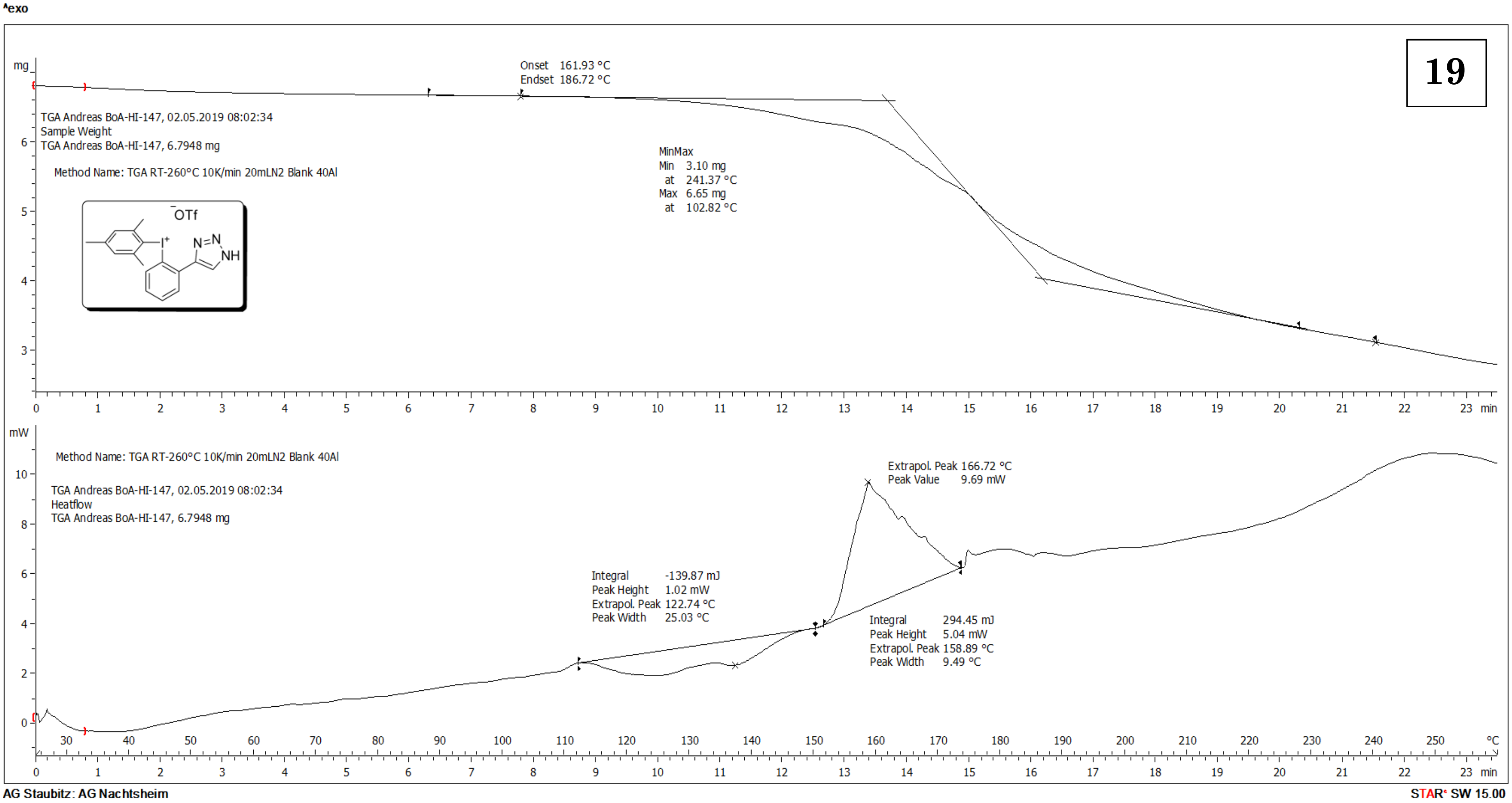

Figure S19: TGA-DSC curve for (2-(1*H*-1,2,3-triazol-4-yl)phenyl)(mesityl)iodonium trifluoromethanesulfonate (**19**) at heating rate 10 °C/min.

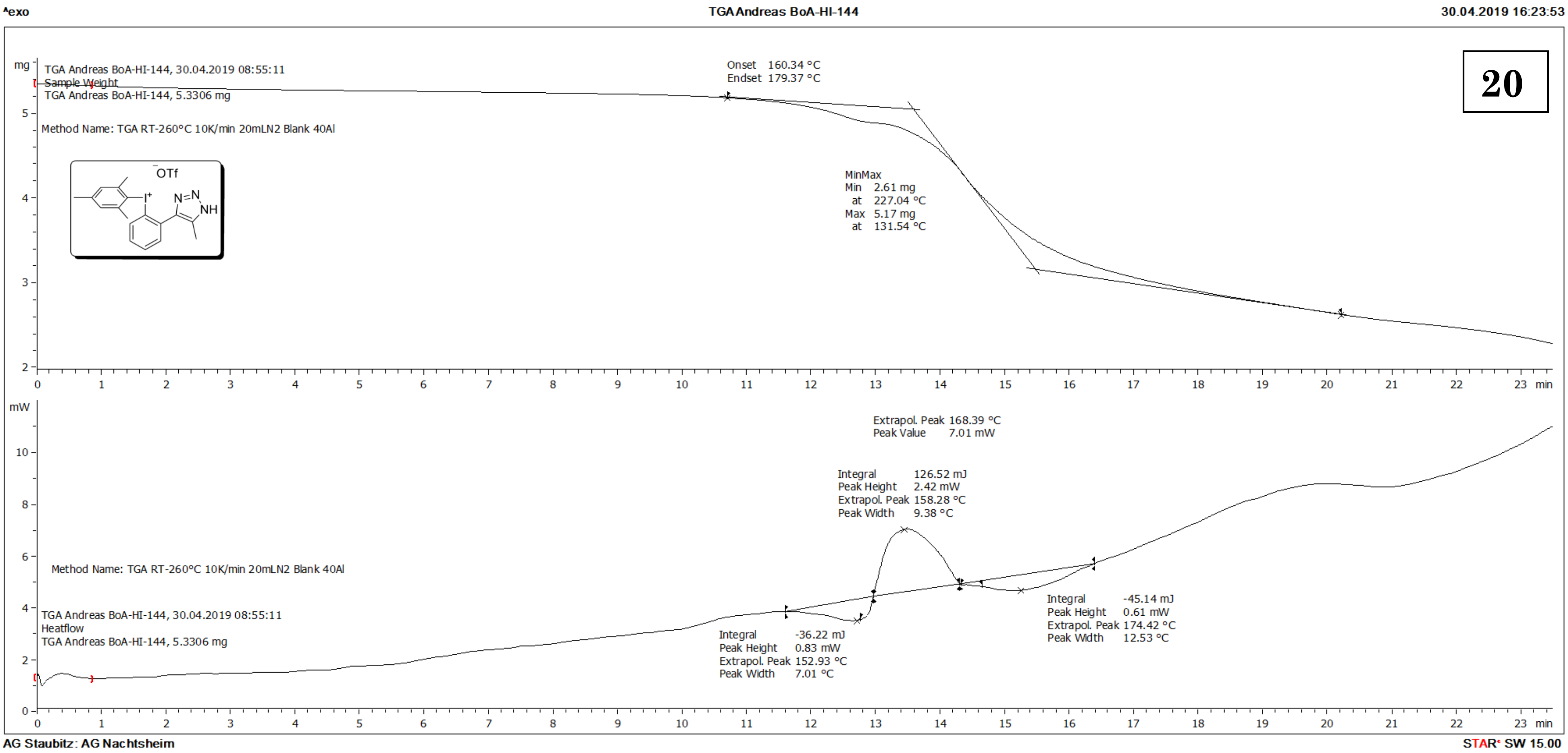

Figure S20: TGA-DSC curve for mesityl(2-(5-methyl-1*H*-1,2,3-triazol-4-yl)phenyl)iodonium trifluoromethanesulfonate (**20**) at heating rate 10 °C/min.

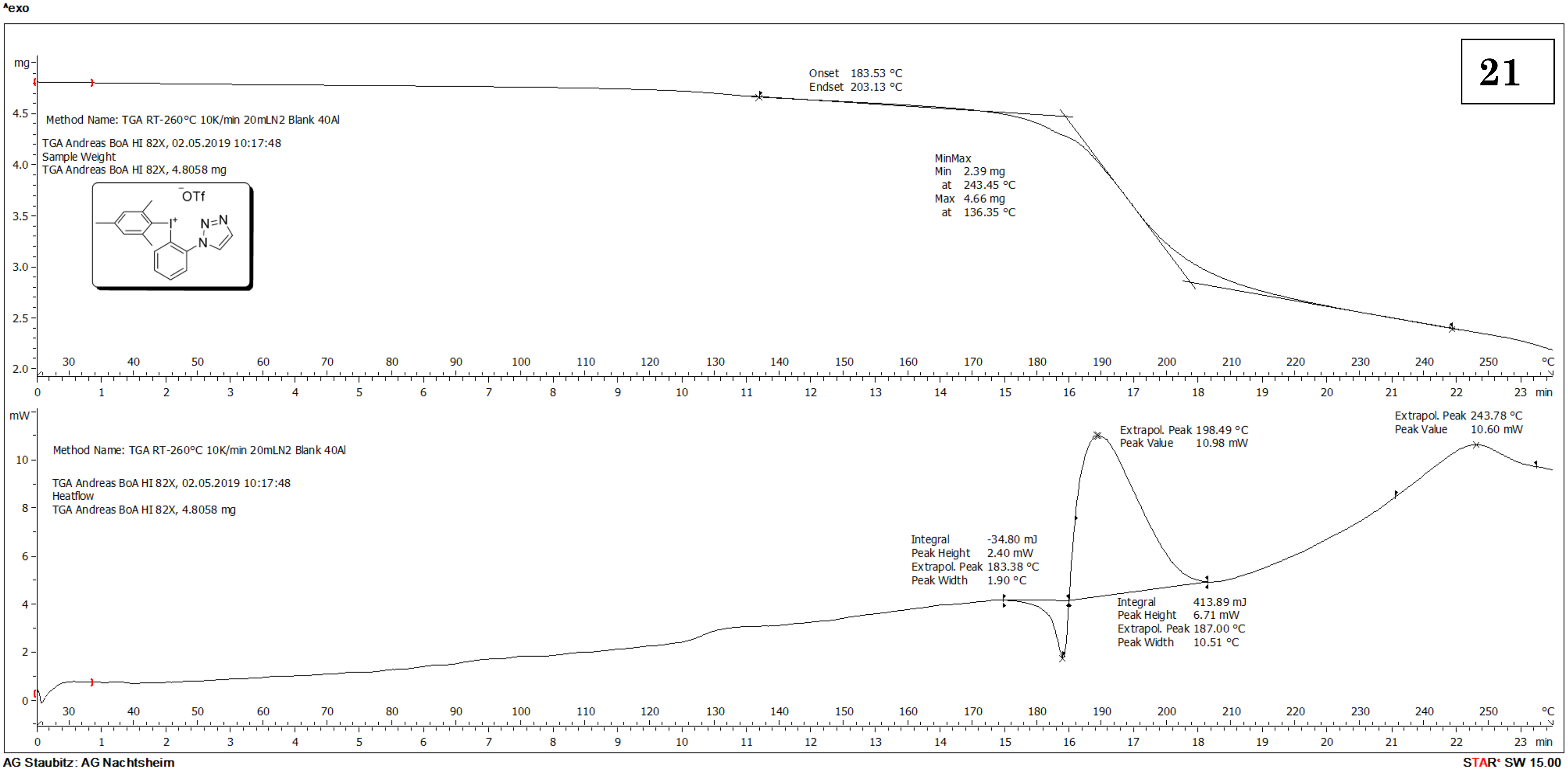

Figure S21: TGA-DSC curve for (2-(1*H*-1,2,3-triazol-1-yl)phenyl)(mesityl)iodonium trifluoromethanesulfonate (**21**) at heating rate 10 °C/min.

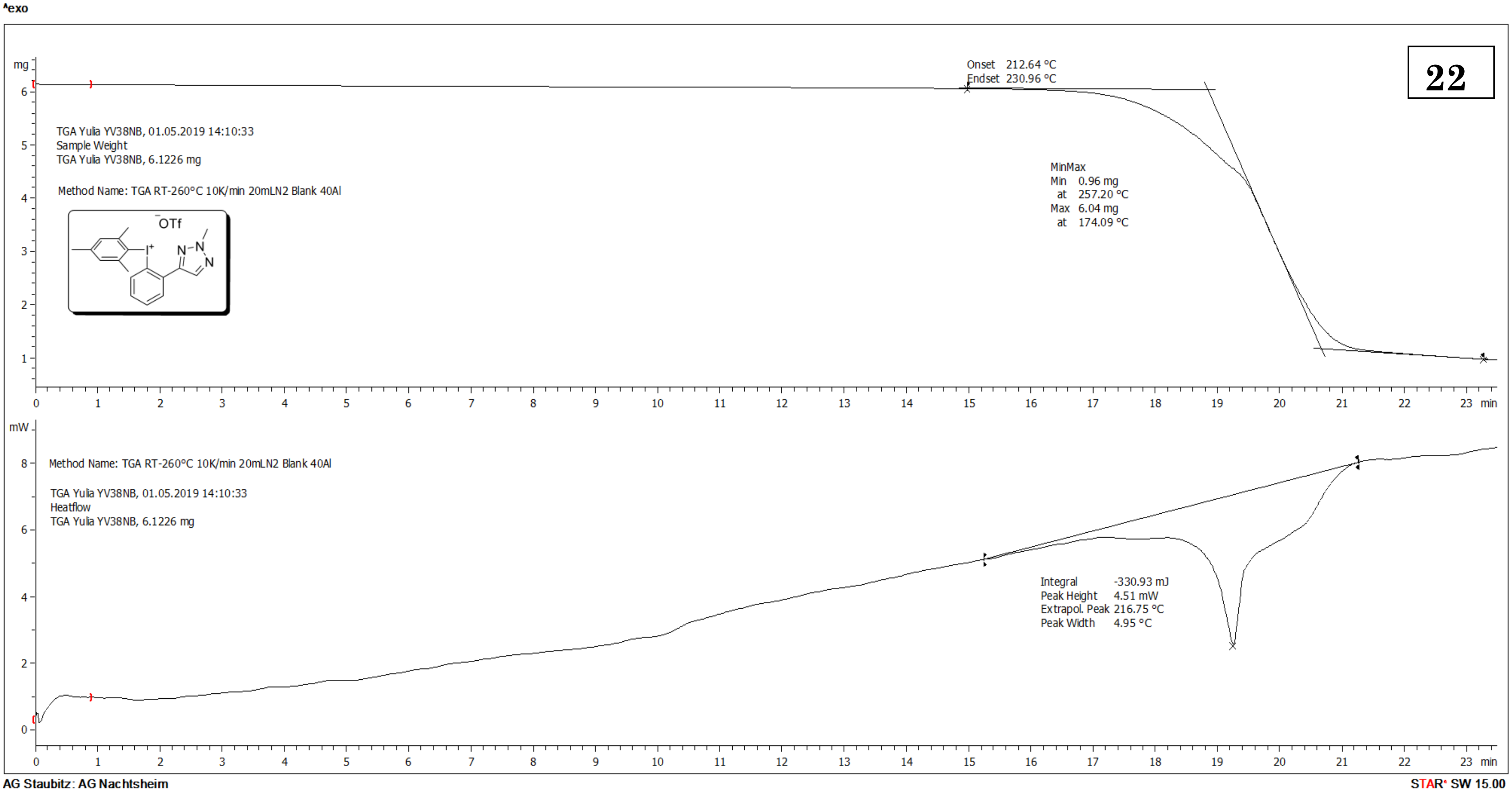

Figure S22: TGA-DSC curve for mesityl(2-(2-methyl-2*H*-1,2,3-triazol-4-yl)phenyl)iodonium trifluoromethanesulfonate (**22**) at heating rate 10 °C/min.

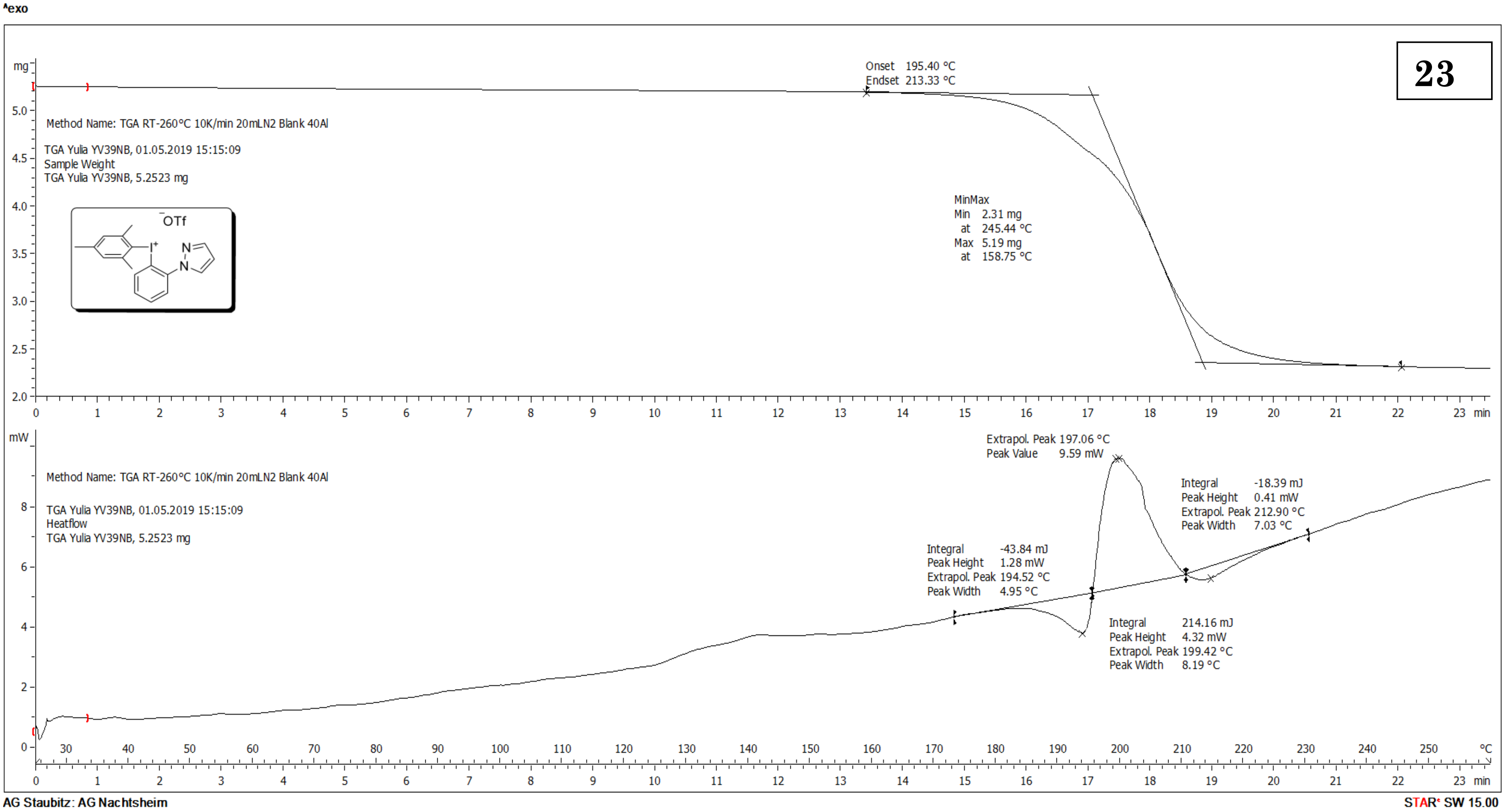

Figure S23: TGA-DSC curve for (2-(1*H*-pyrazol-1-yl)phenyl)(mesityl)iodonium trifluoromethanesulfonate (**23**) at heating rate 10 °C/min.

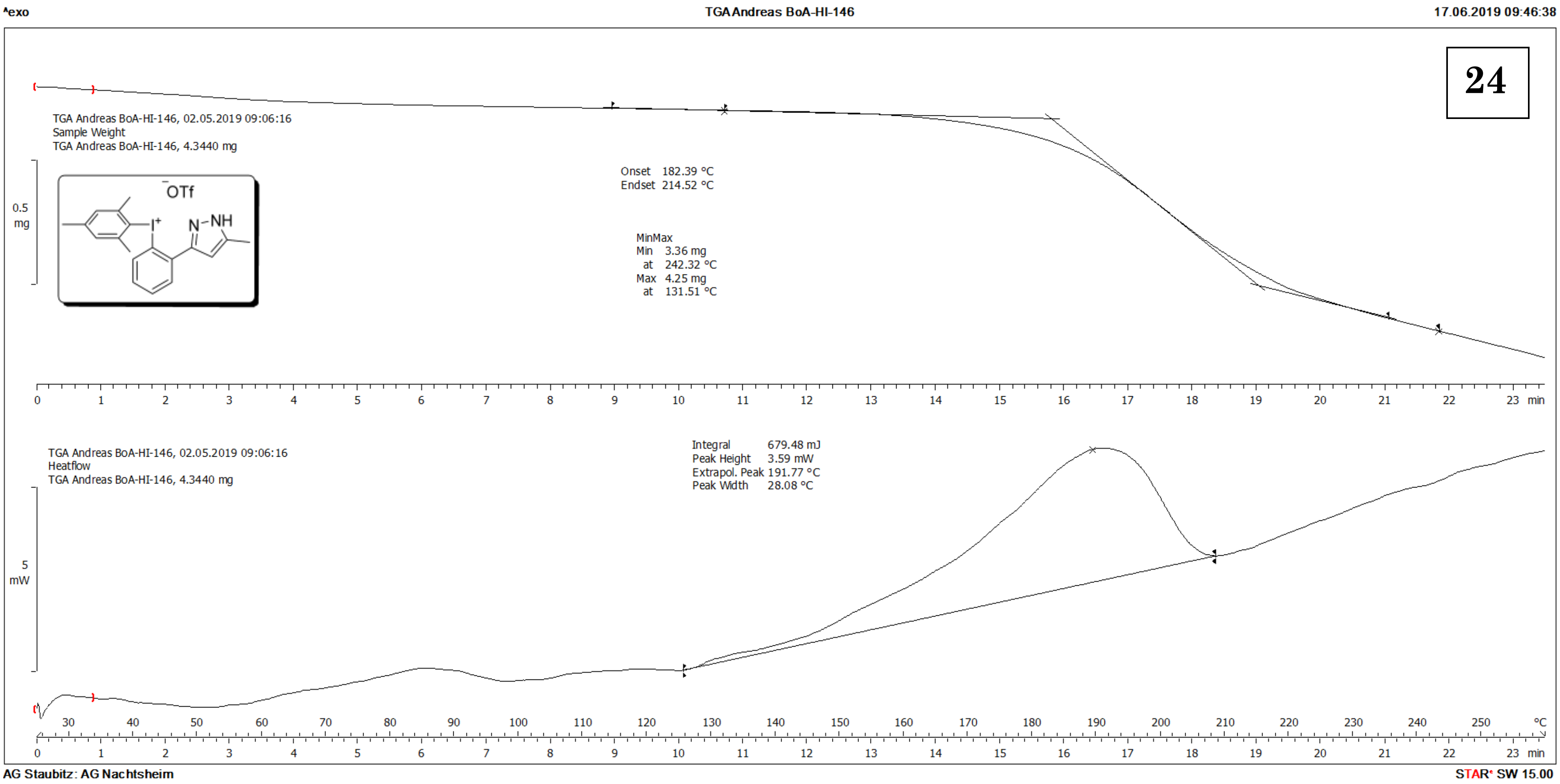

Figure S24: TGA-DSC curve for mesityl(2-(5-methyl-1*H*-pyrazol-3-yl)phenyl)iodonium trifluoromethanesulfonate (**24**) at heating rate 10 °C/min.

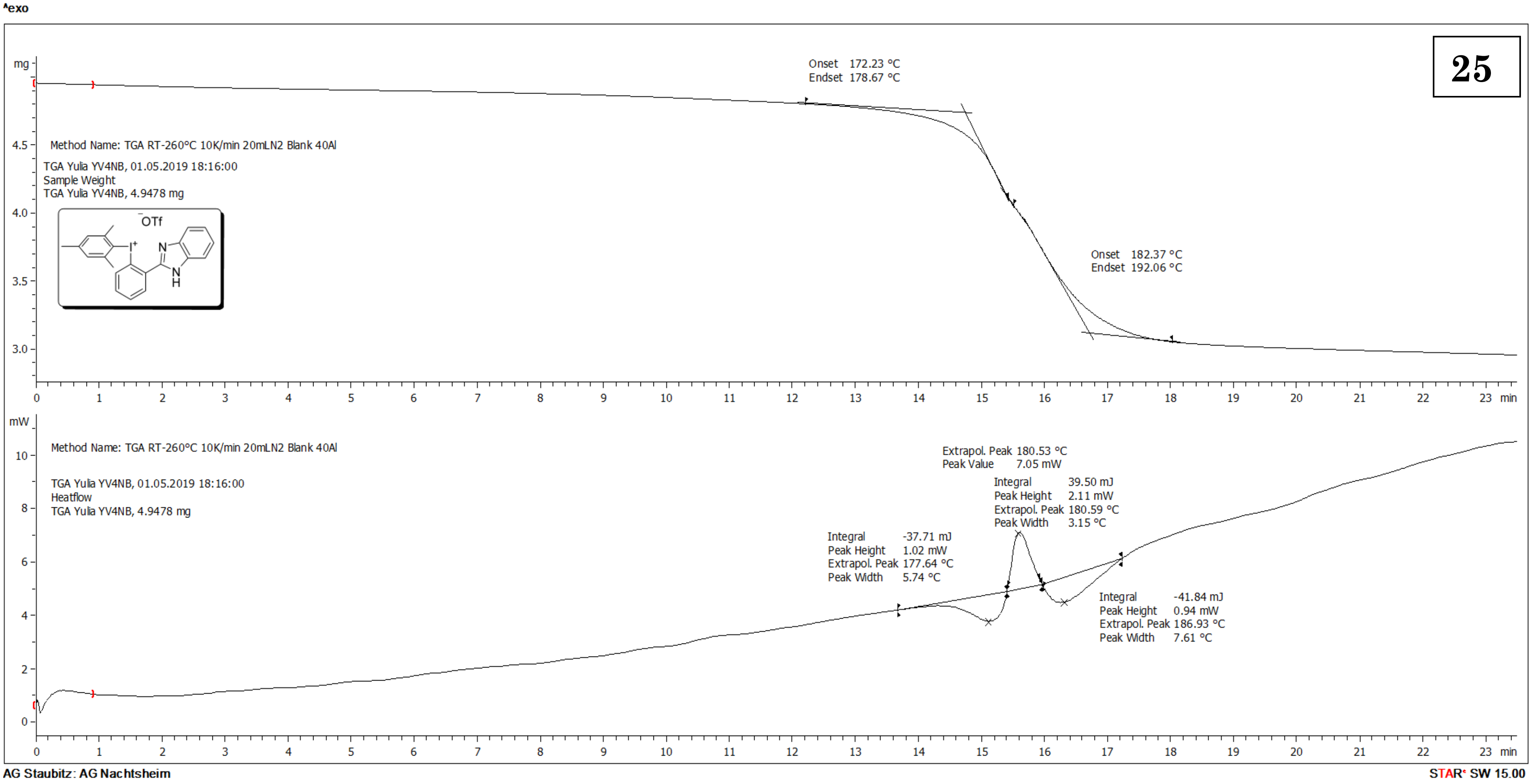

Figure S25: TGA-DSC curve for (2-(1*H*-benzo[*d*]imidazol-2-yl)phenyl)(mesityl)iodonium trifluoromethanesulfonate (**25**) at heating rate 10 °C/min.

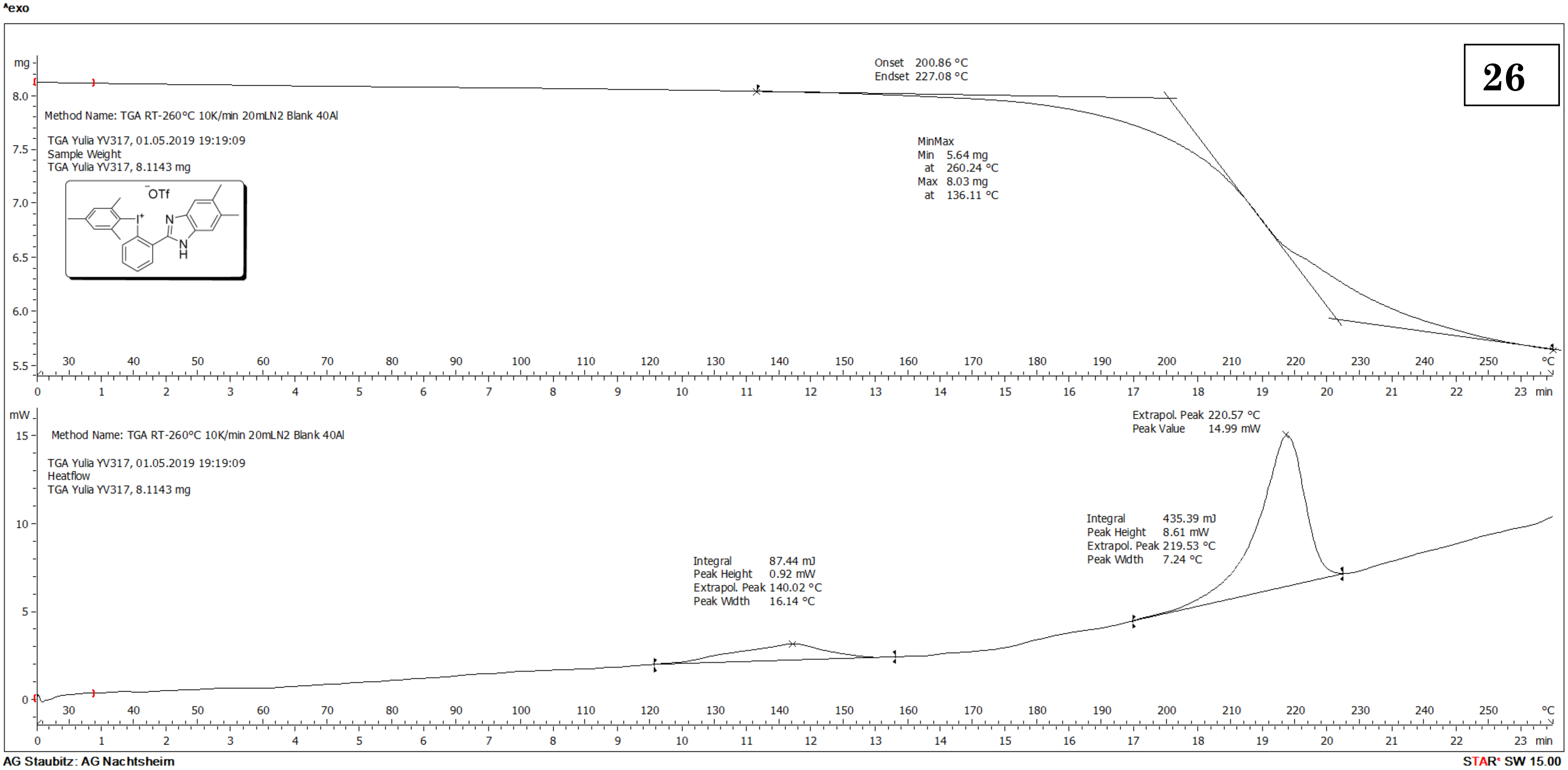

Figure S26: TGA-DSC curve for (2-(5,6-dimethyl-1*H*-benzo[*d*]imidazol-2-yl)phenyl)(mesityl)iodonium trifluoromethanesulfonate (**26**) at heating rate 10 °C/min.

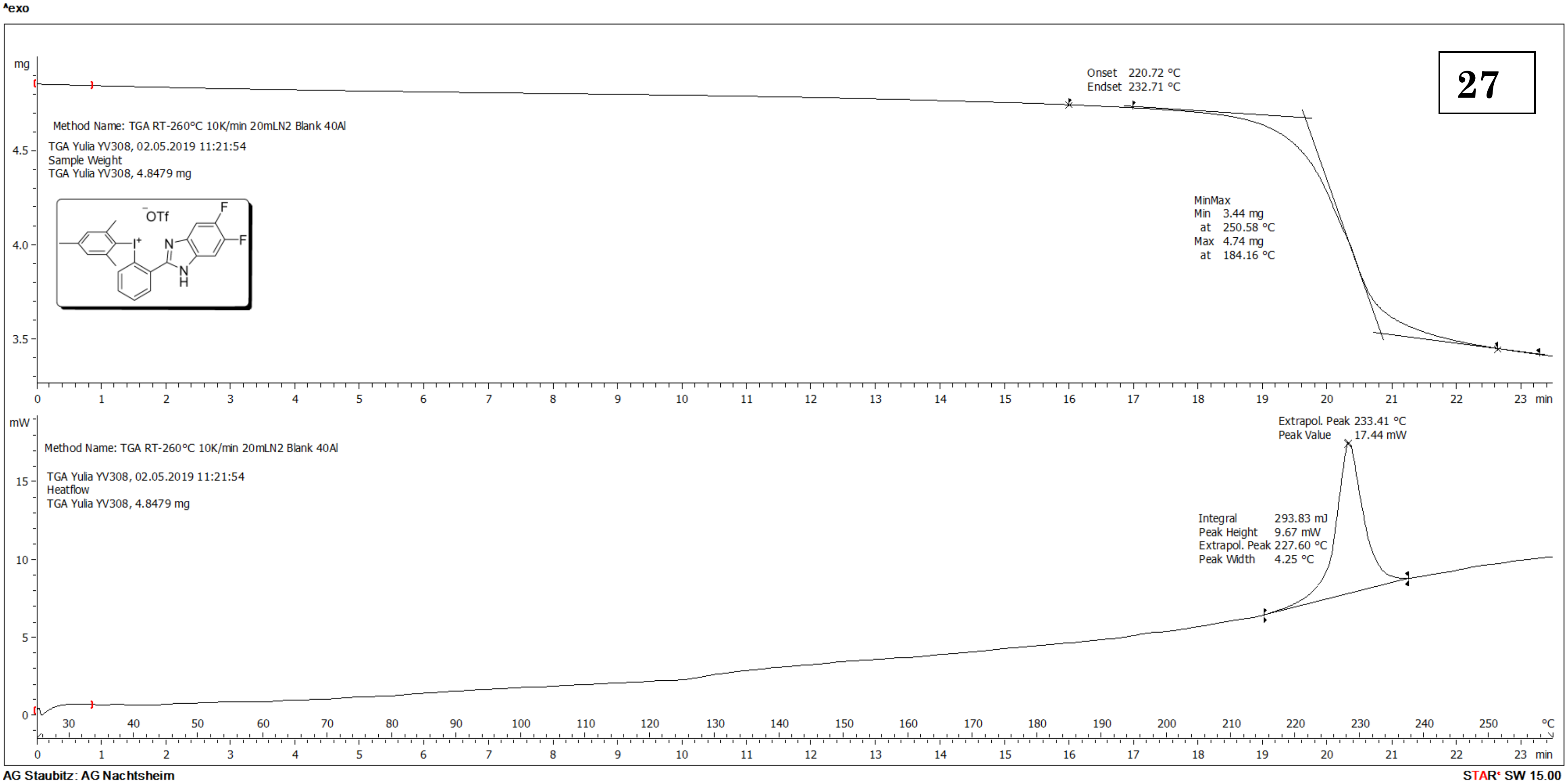

Figure S27: TGA-DSC curve for (2-(5,6-difluoro-1*H*-benzo[*d*]imidazol-2-yl)phenyl)(mesityl)iodonium trifluoromethanesulfonate (**27**) at heating rate 10 °C/min.

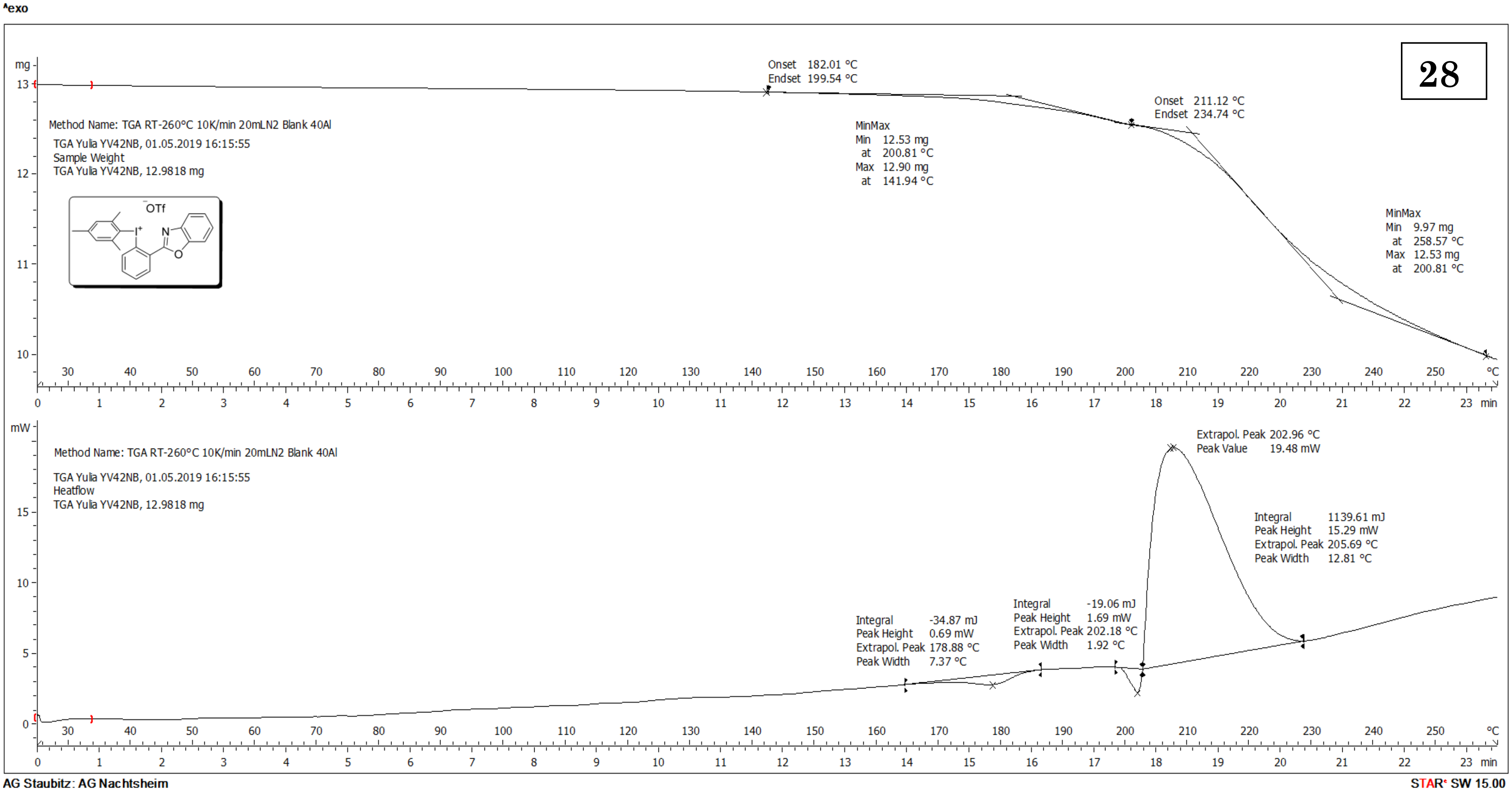

Figure S28: TGA-DSC curve for (2-(benzo[*d*]oxazol-2-yl)phenyl)(mesityl)iodonium trifluoromethanesulfonate (**28**) at heating rate 10 °C/min.

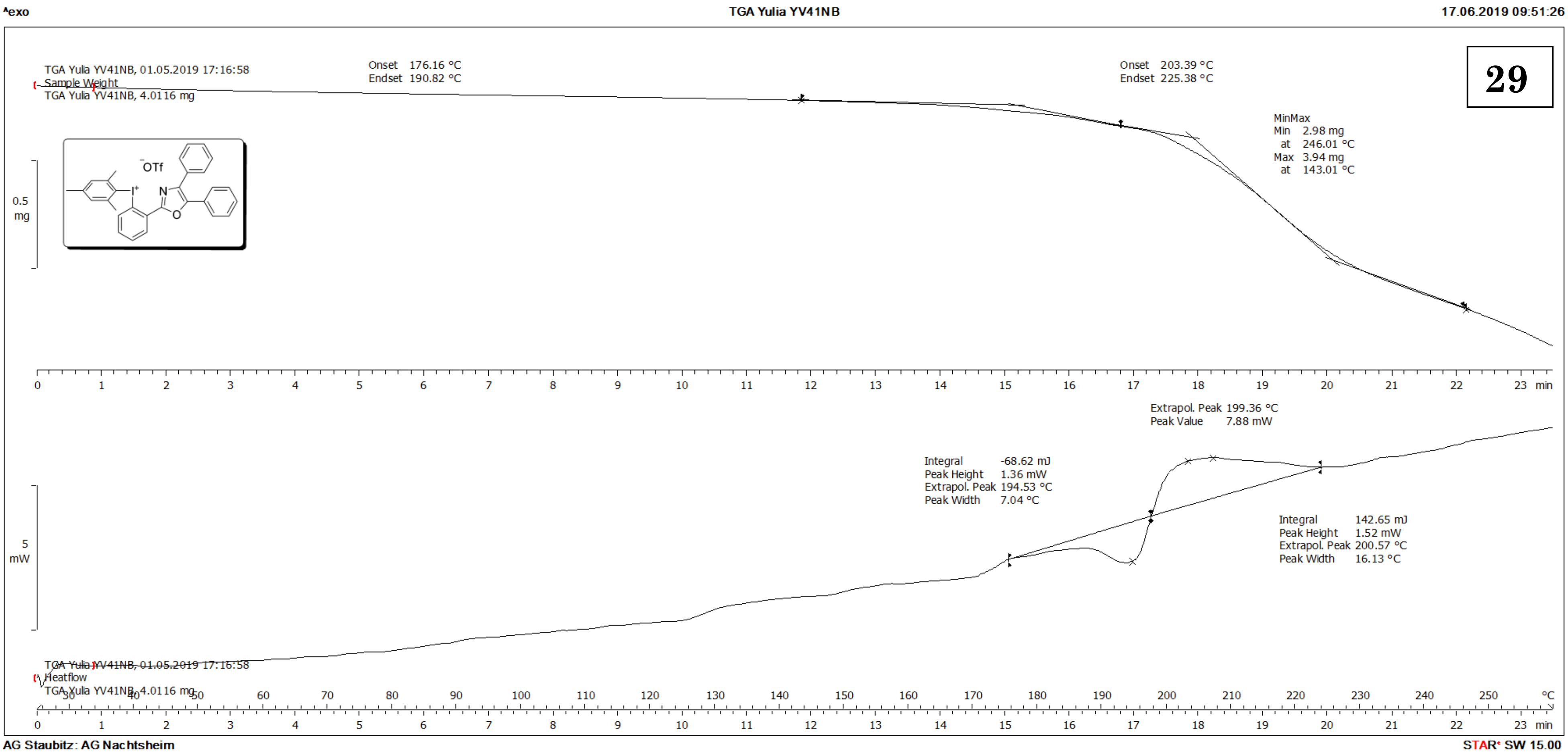

Figure S29: TGA-DSC curve for (2-(4,5-diphenyloxazol-2-yl)phenyl)(mesityl)iodonium trifluoromethanesulfonate (**29**) at heating rate 10 °C/min.

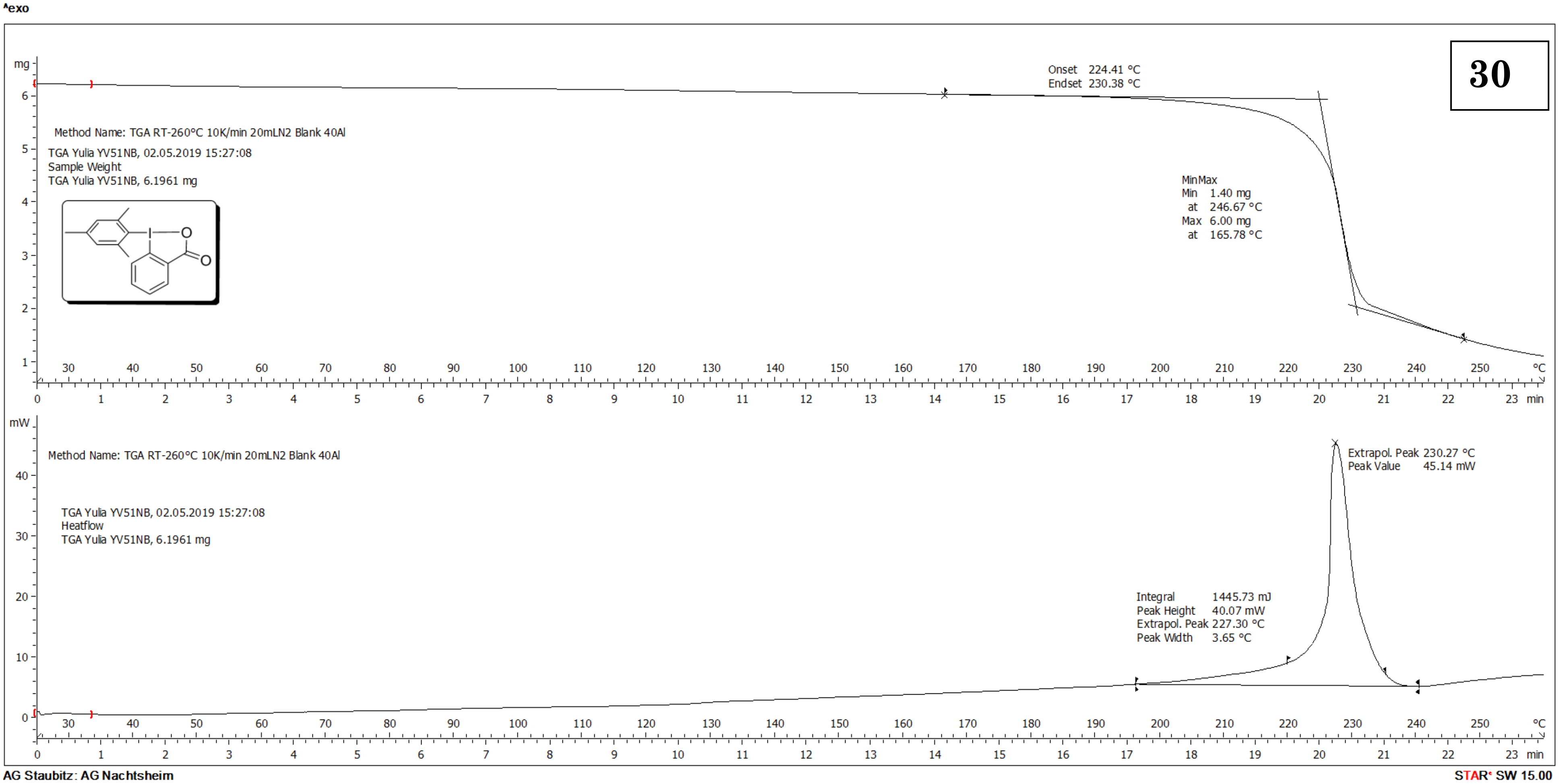

Figure S30: TGA-DSC curve for 1-mesityl-1λ^3-benzo[*d*][1,2]iodaoxol-3(1*H*)-one (**30**) at heating rate 10 °C/min.

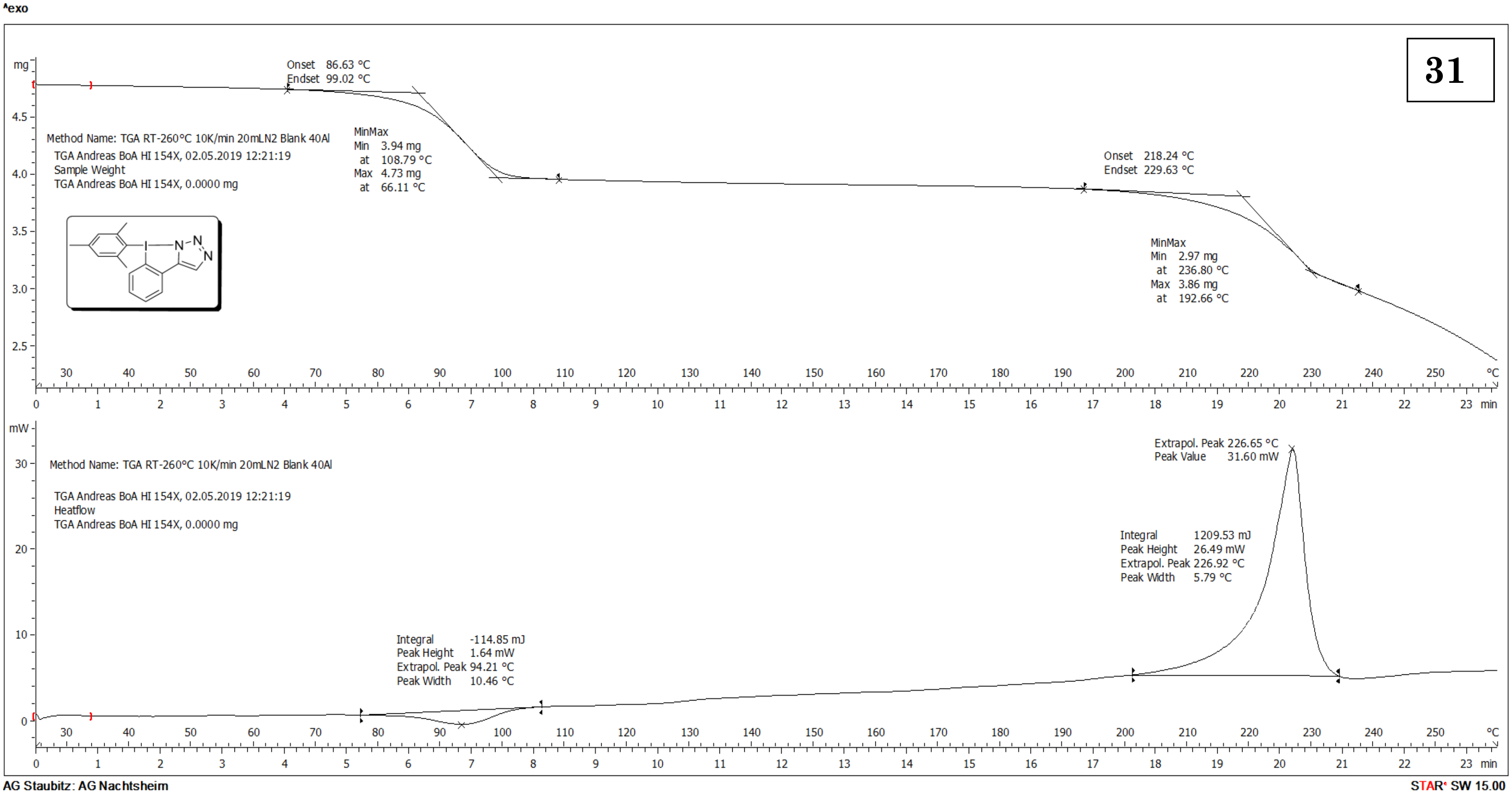

Figure S31: TGA-DSC curve for 8-mesityl-8*H*-8λ^3-benzo[4,5][1,2]iodazolo[2,3-*c*][1,2,3]triazole (**31**) at heating rate 10 °C/min.

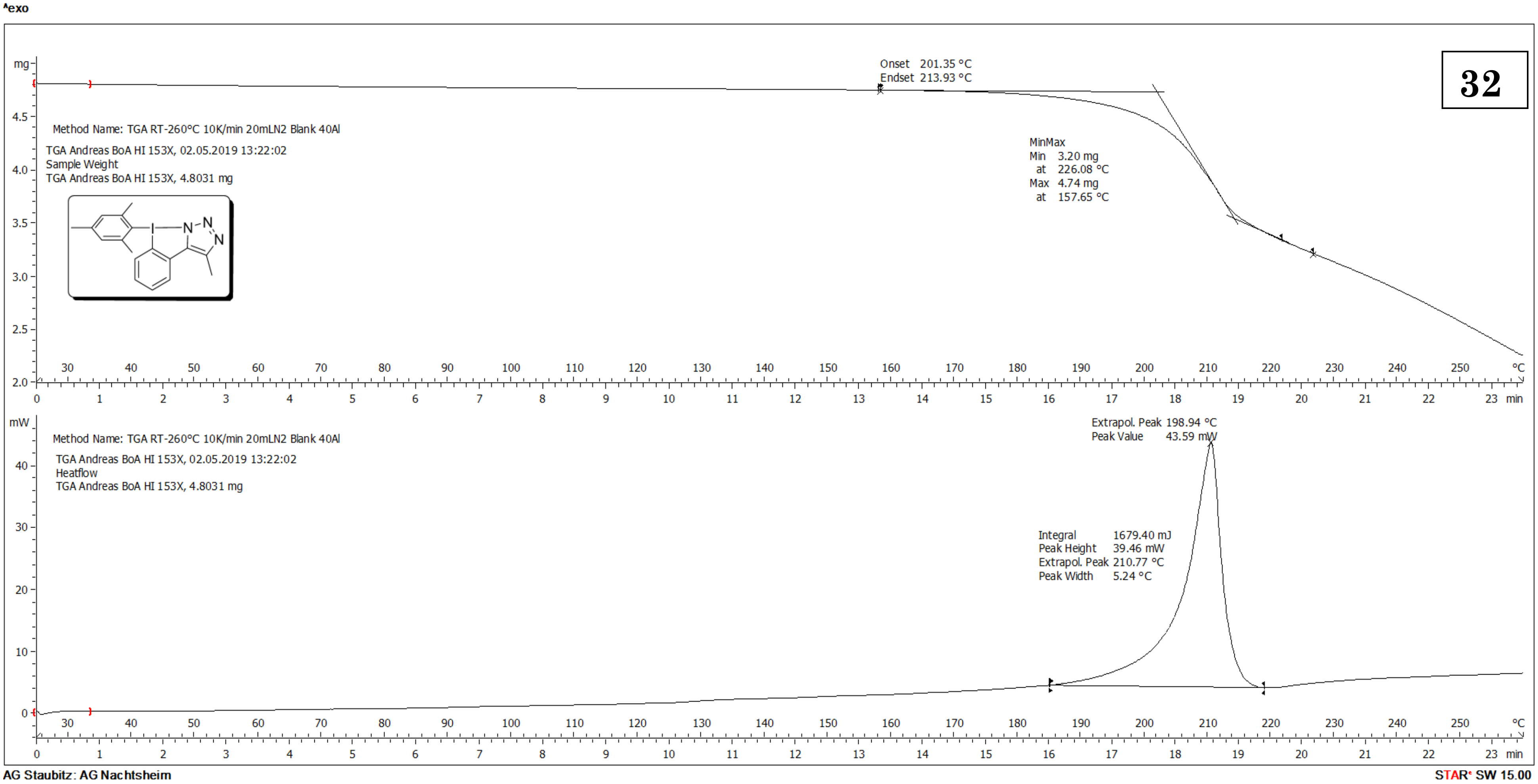

Figure S32: TGA-DSC curve for 8-mesityl-3-methyl-8*H*-8λ^3-benzo[4,5][1,2]iodazolo[2,3-*c*][1,2,3]triazole (**32**) at heating rate 10 °C/min.

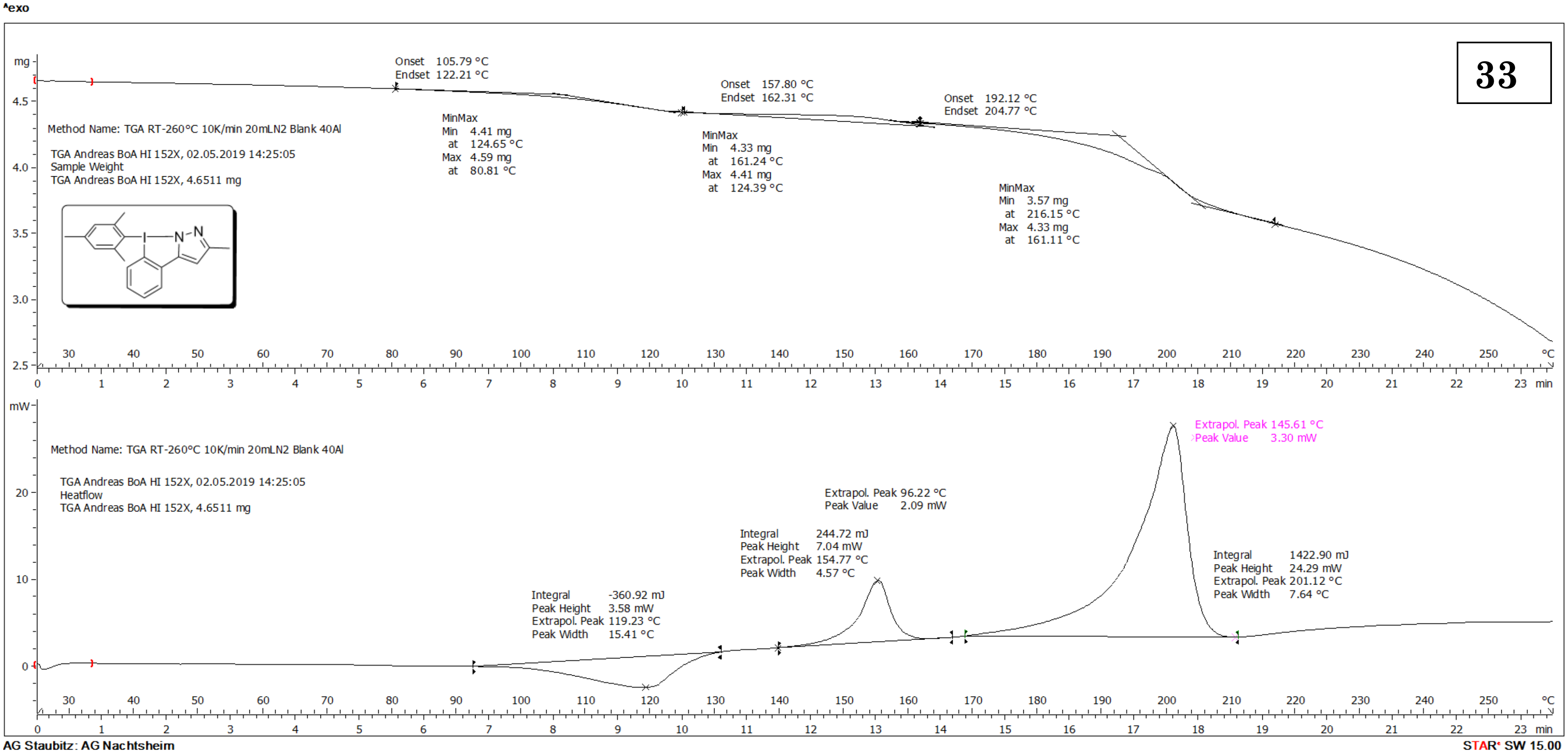

Figure S33: TGA-DSC curve for 8-mesityl-2-methyl-8*H*-8λ^3-benzo[*d*]pyrazolo[1,5-*b*][1,2]iodazole (**33**) at heating rate 10 °C/min.

NMR-Spectra

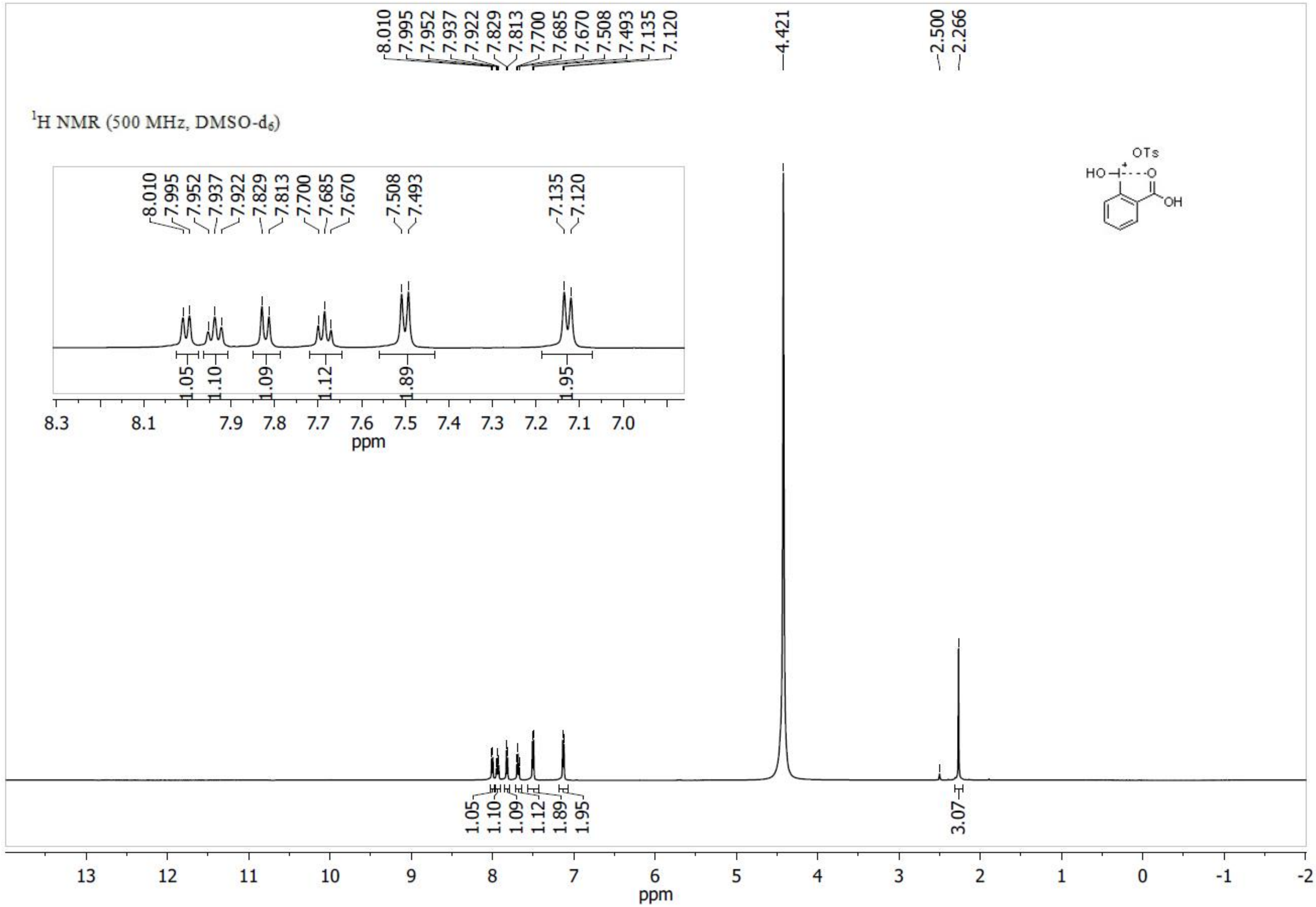

Figure S34: ^{1}H NMR spectra of **1** in d_6-DMSO.

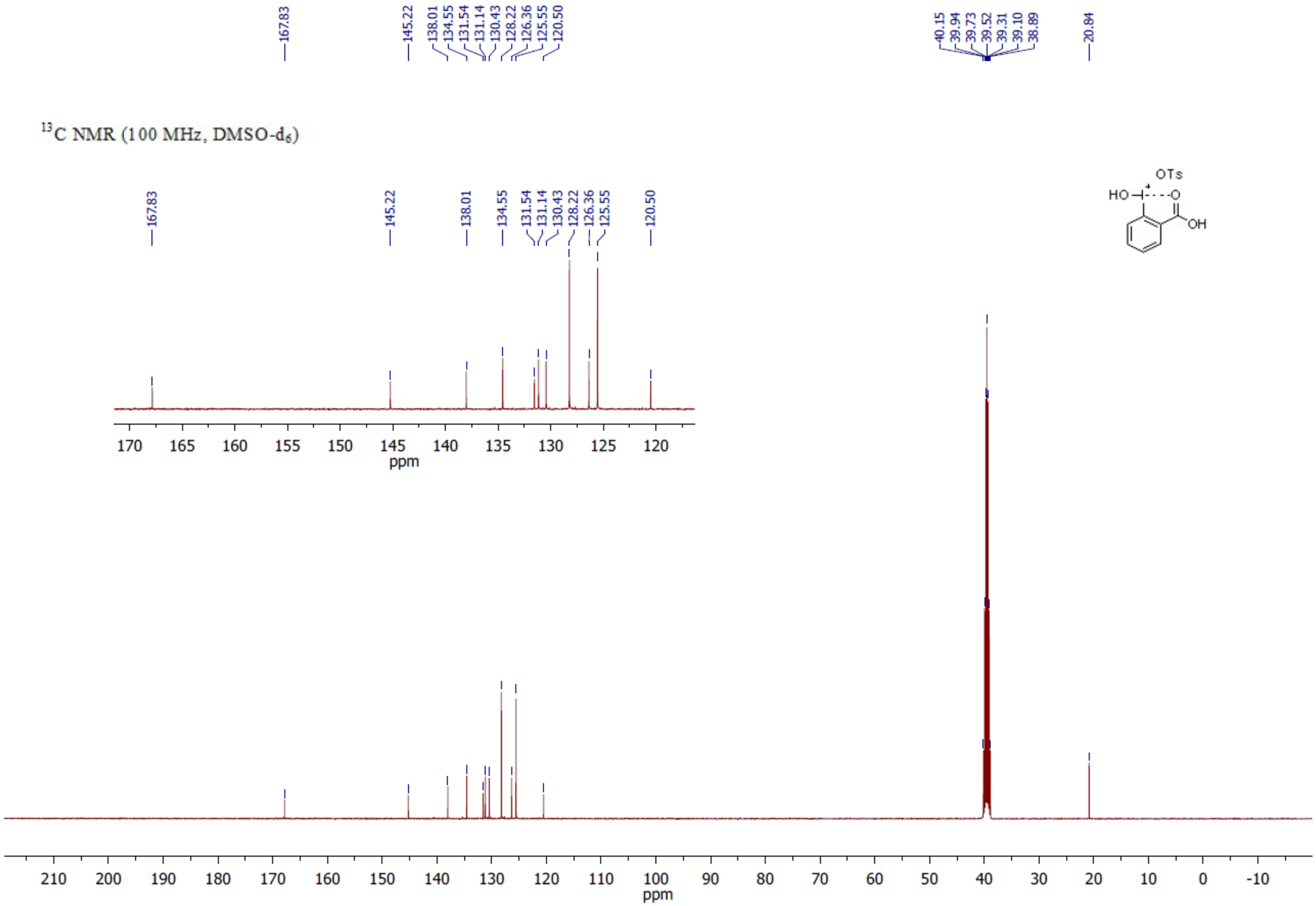

Figure S35: ^{13}C NMR spectra of **1** in *d*$_6$-DMSO.

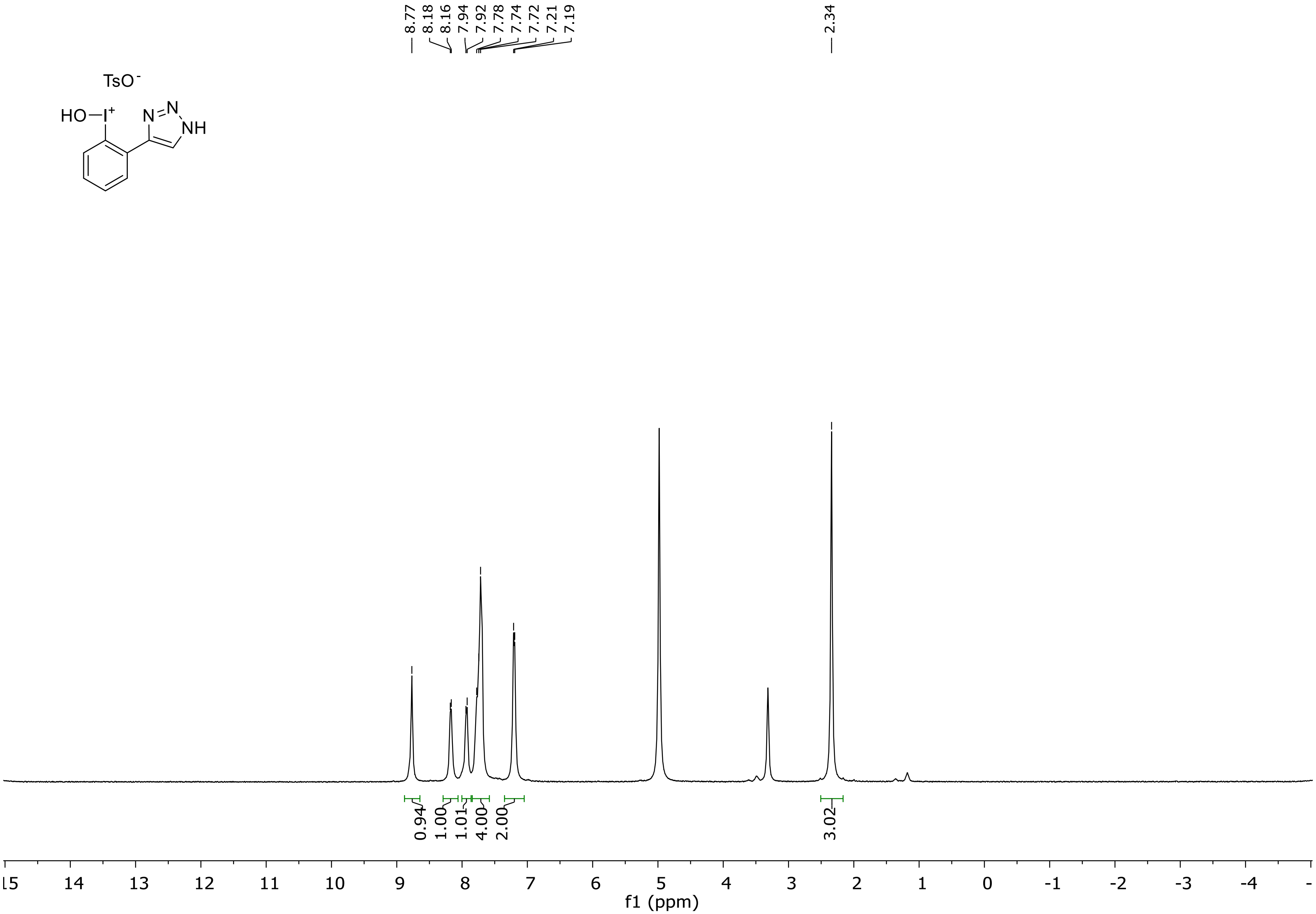

Figure S36: ^{1}H NMR spectra of **2** in d_4-MeOH.

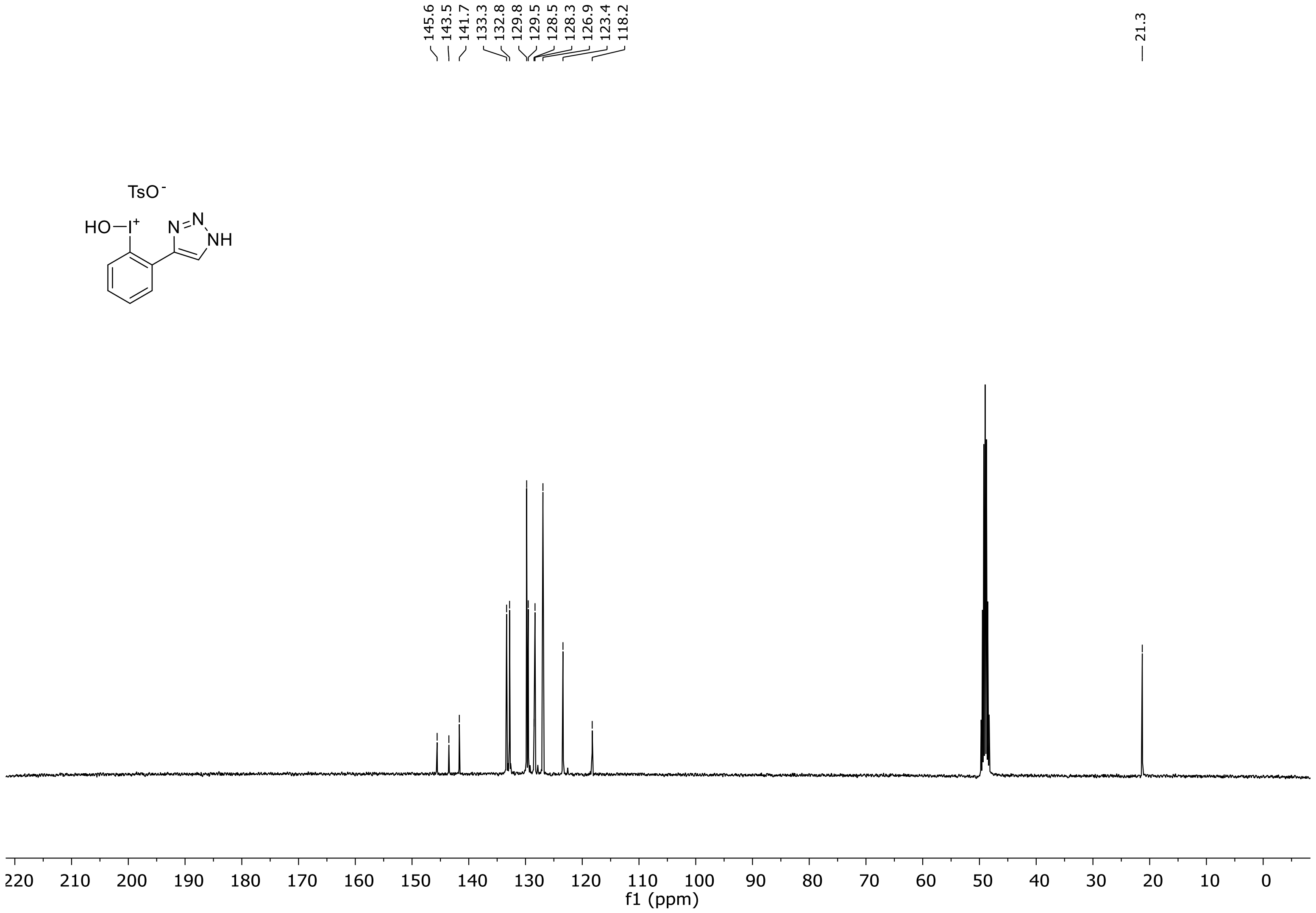

Figure S37: ^{13}C NMR spectra of **2** in *d*$_4$-MeOH.

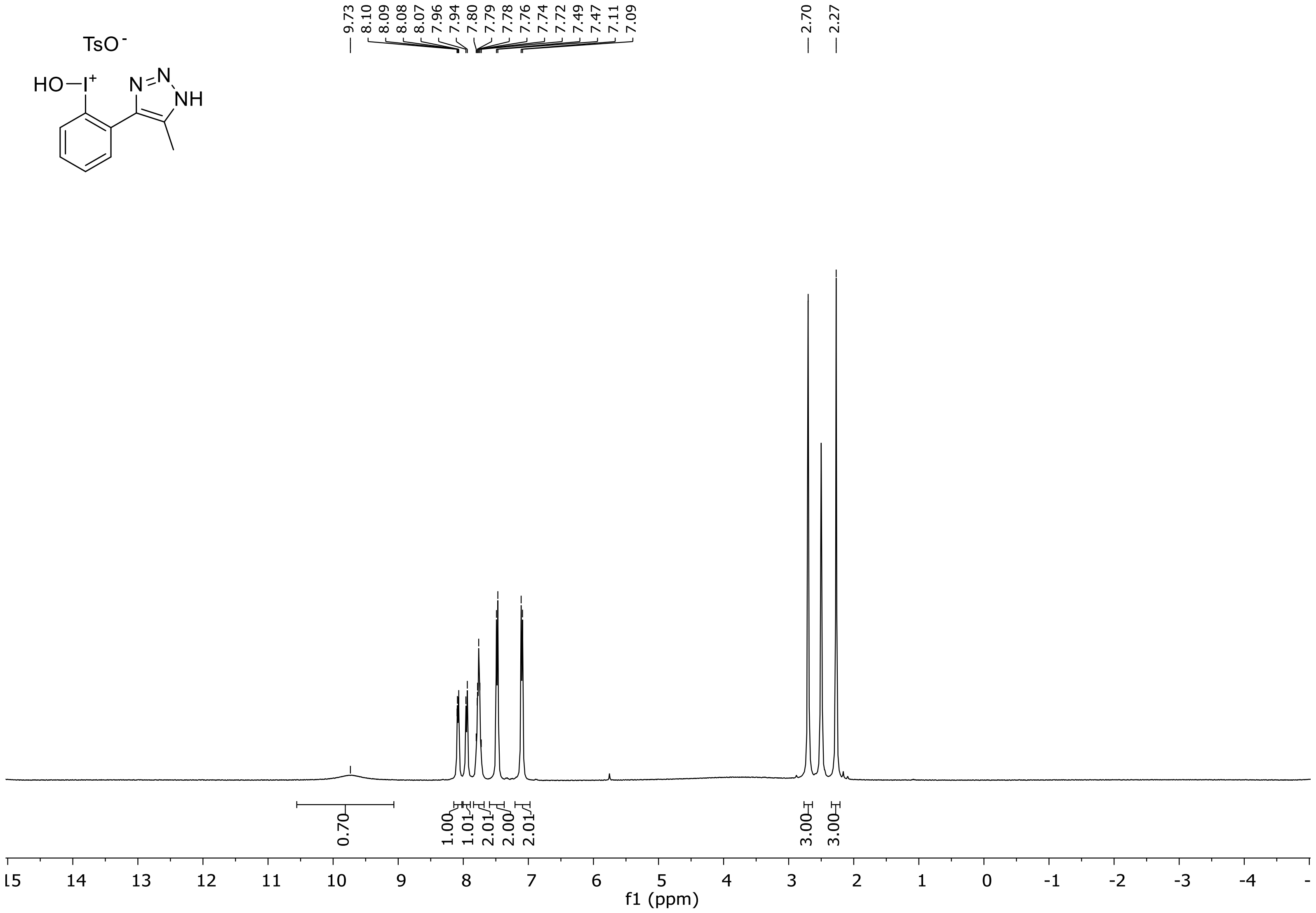

Figure S38: ^{1}H NMR spectra of **3** in d_6-DMSO.

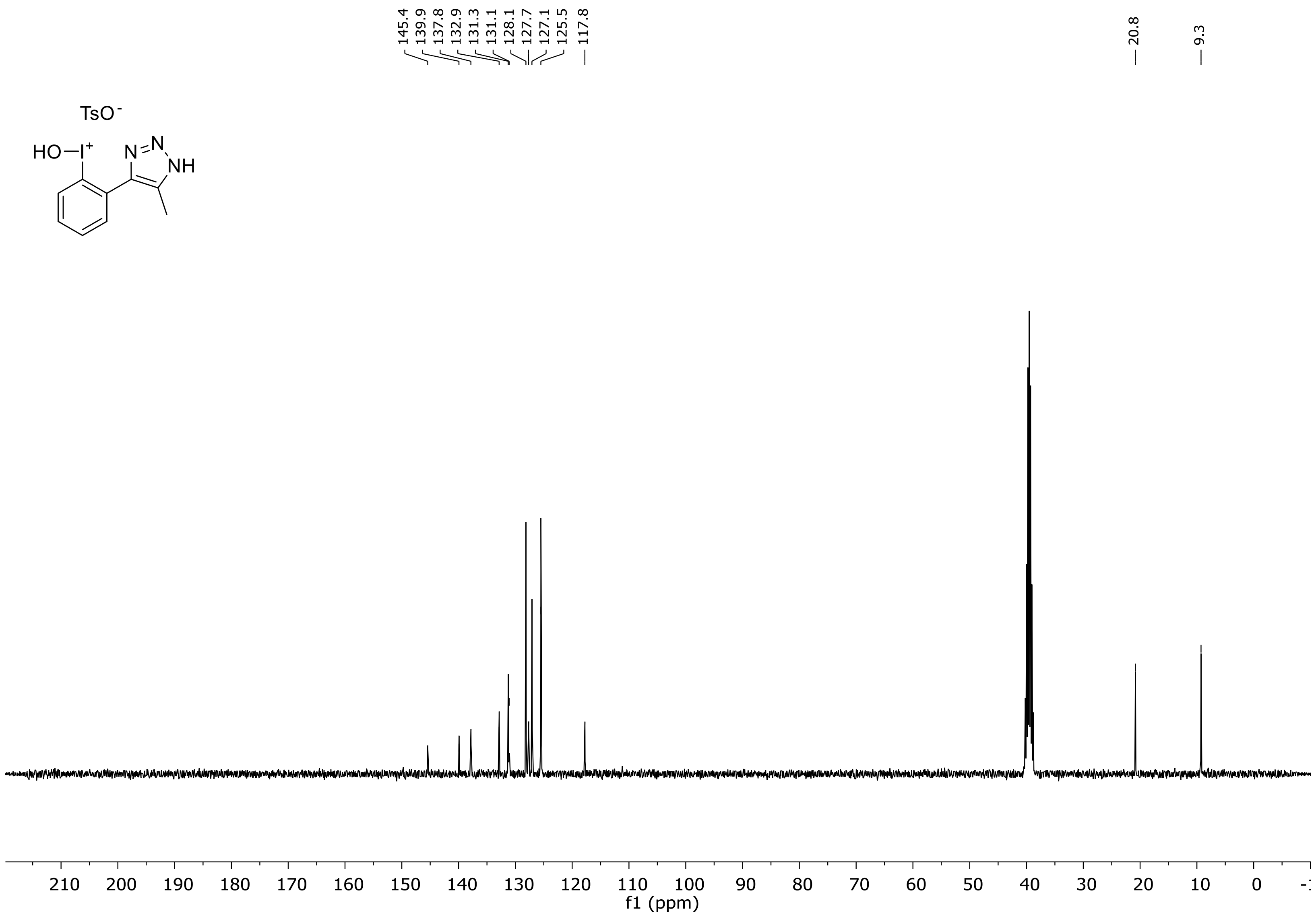

Figure S39: ^{13}C NMR spectra of **3** in d_6-DMSO.

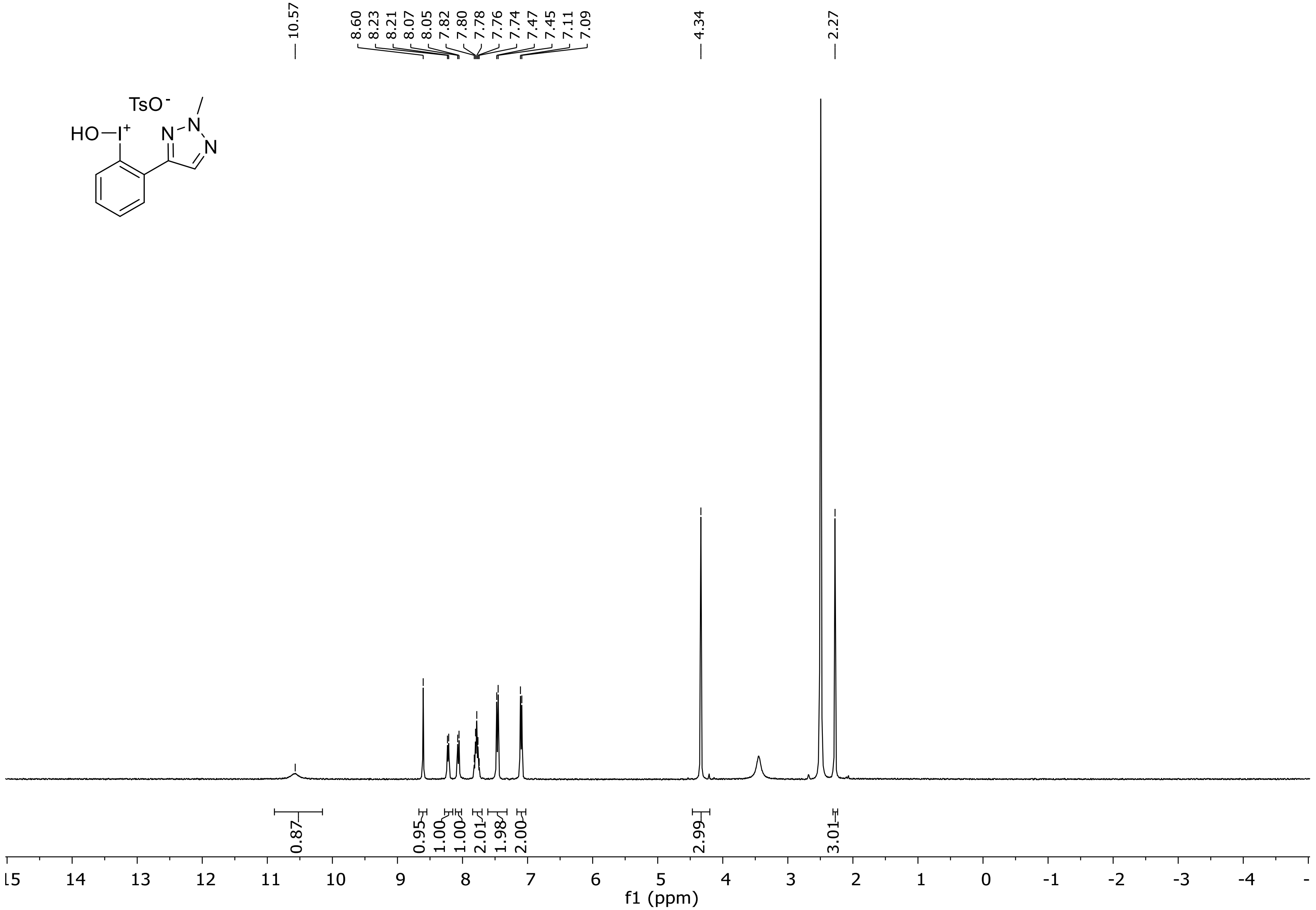

Figure S40: ^{1}H NMR spectra of **4** in d_6-DMSO.

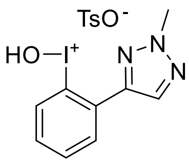

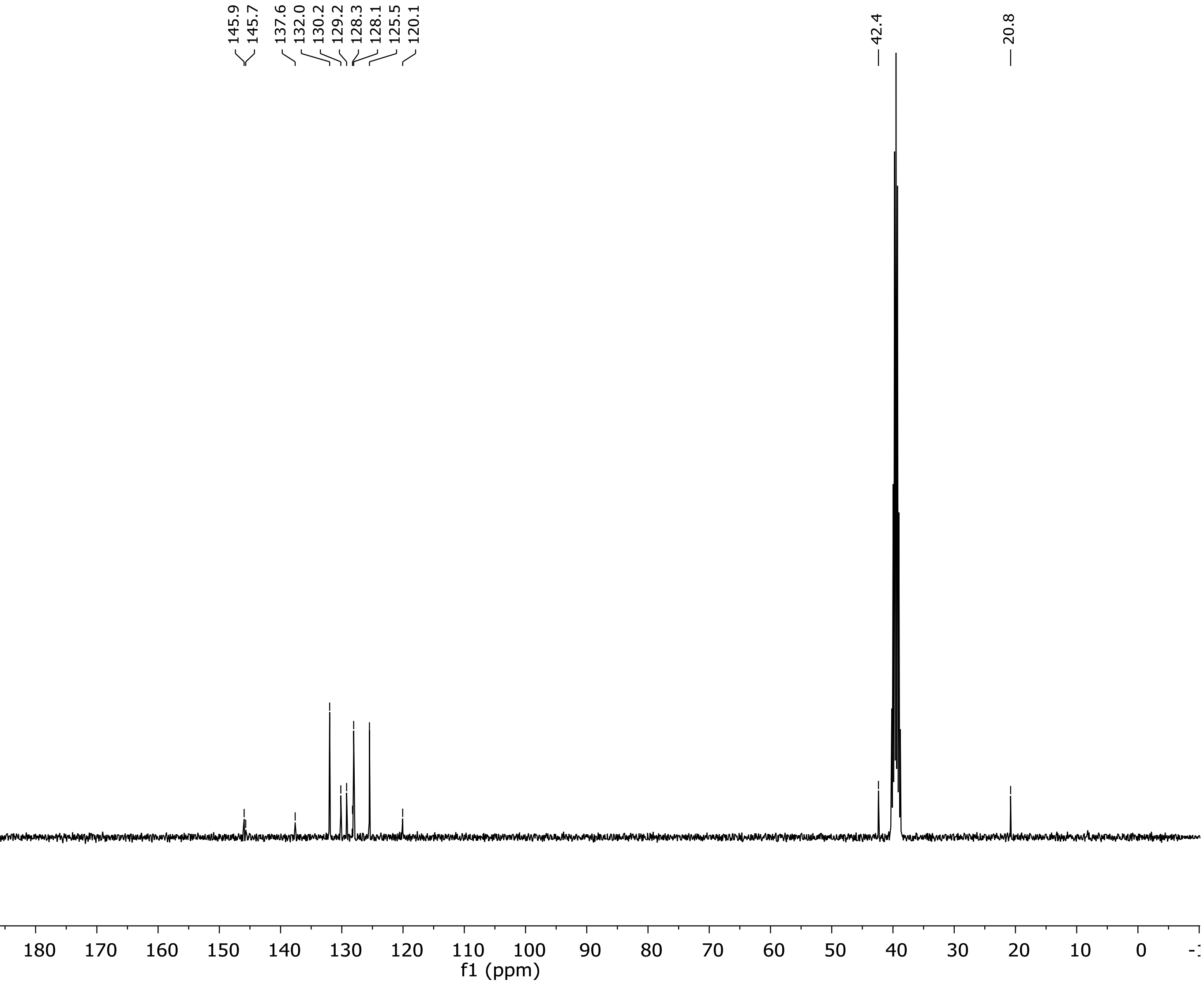

Figure S41: ^{13}C NMR spectra of **4** in *d*$_6$-DMSO.

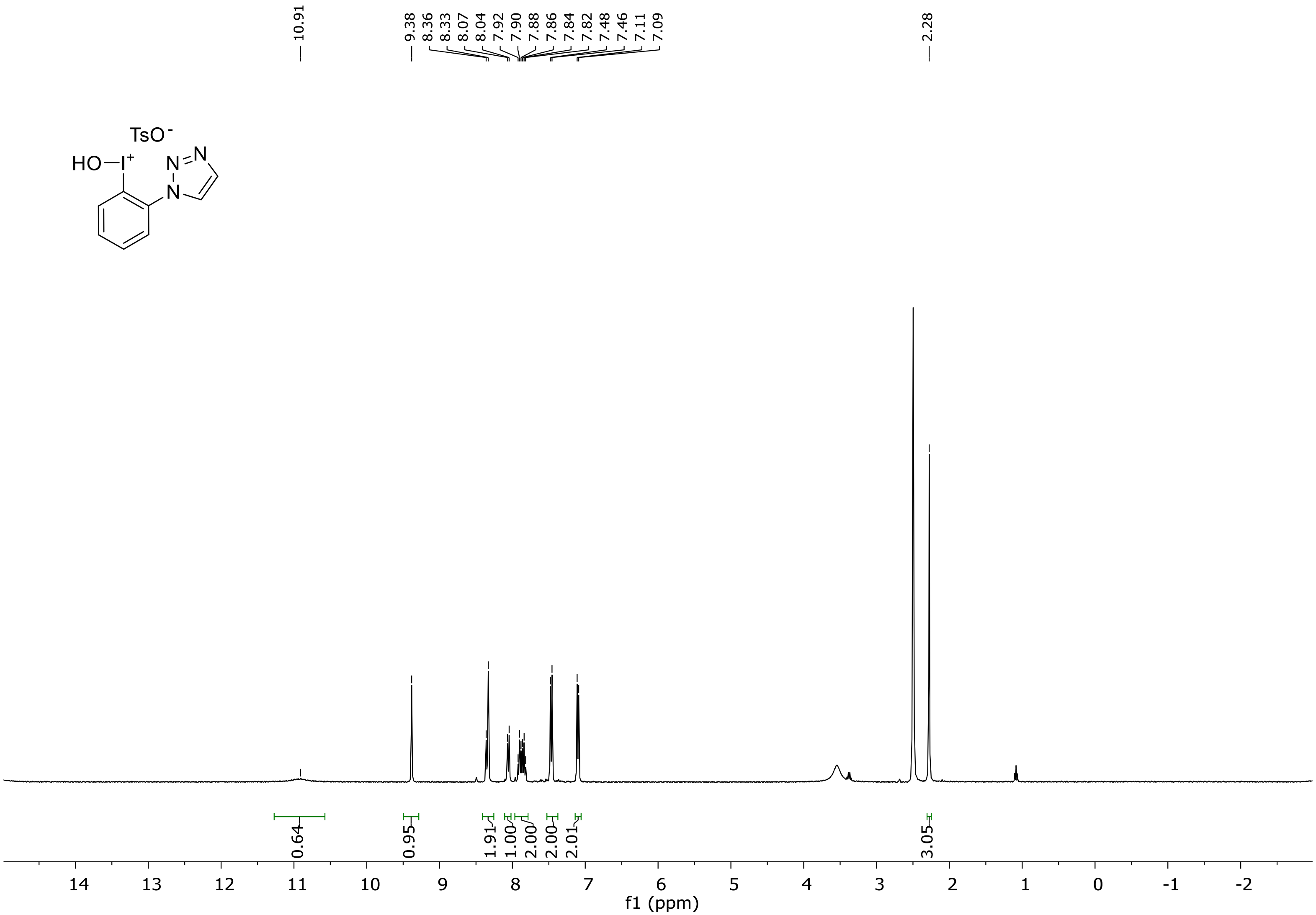

Figure S42: ^{1}H NMR spectra of **5** in d_6-DMSO.

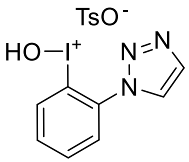

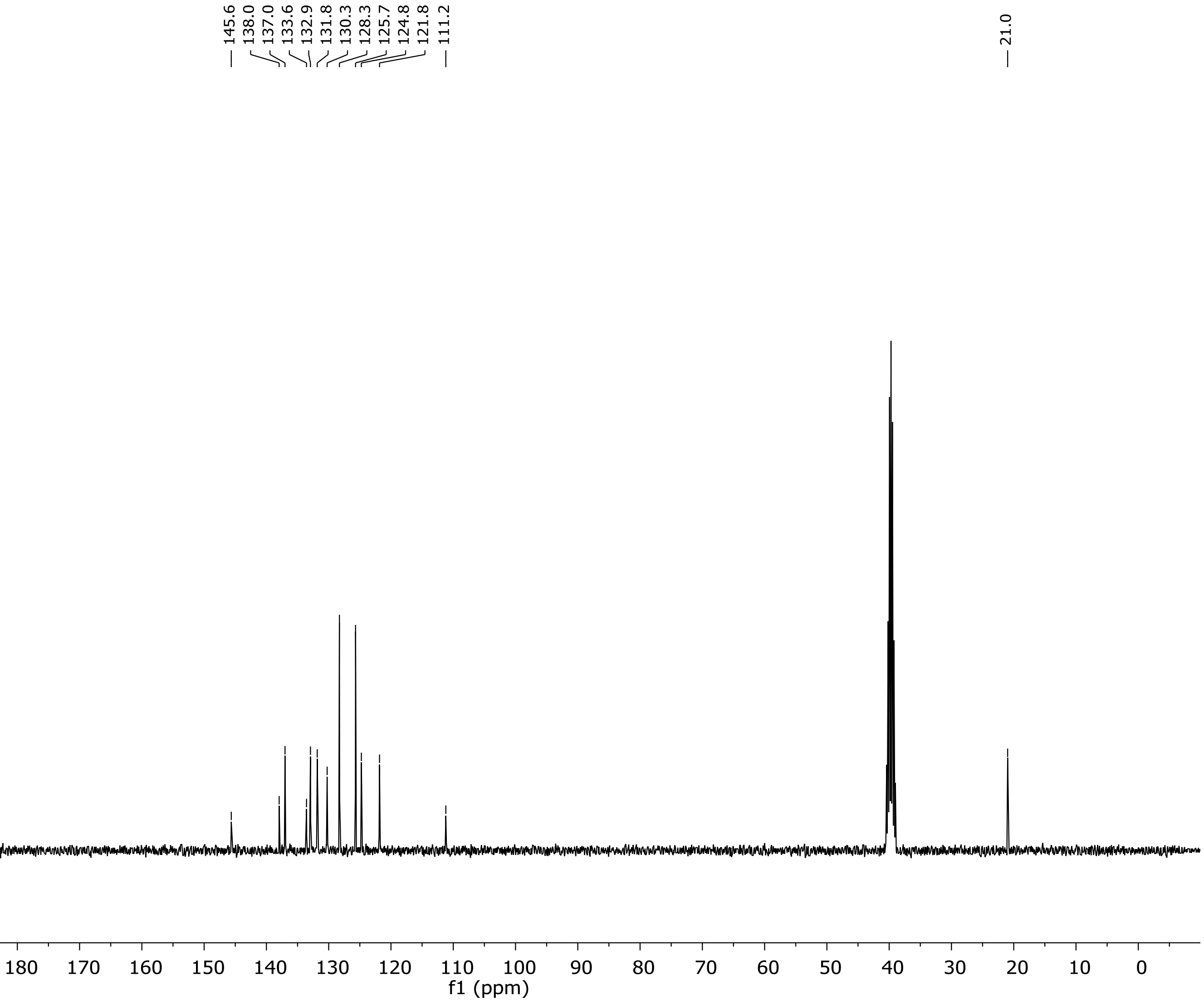

Figure S43: ^{13}C NMR spectra of **5** in d_6-DMSO.

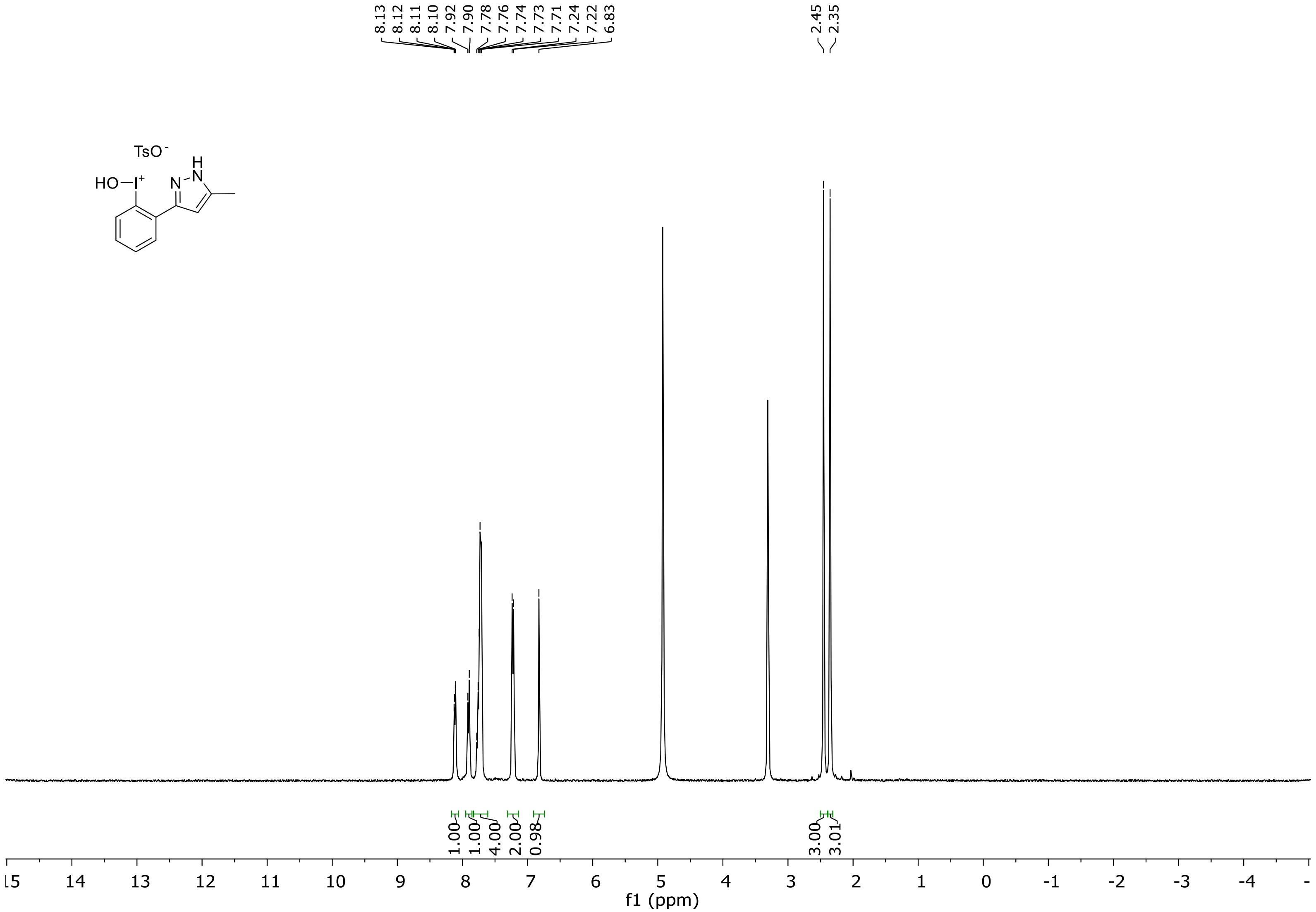

Figure S44: ^{1}H NMR spectra of **6** in d_4-MeOH.

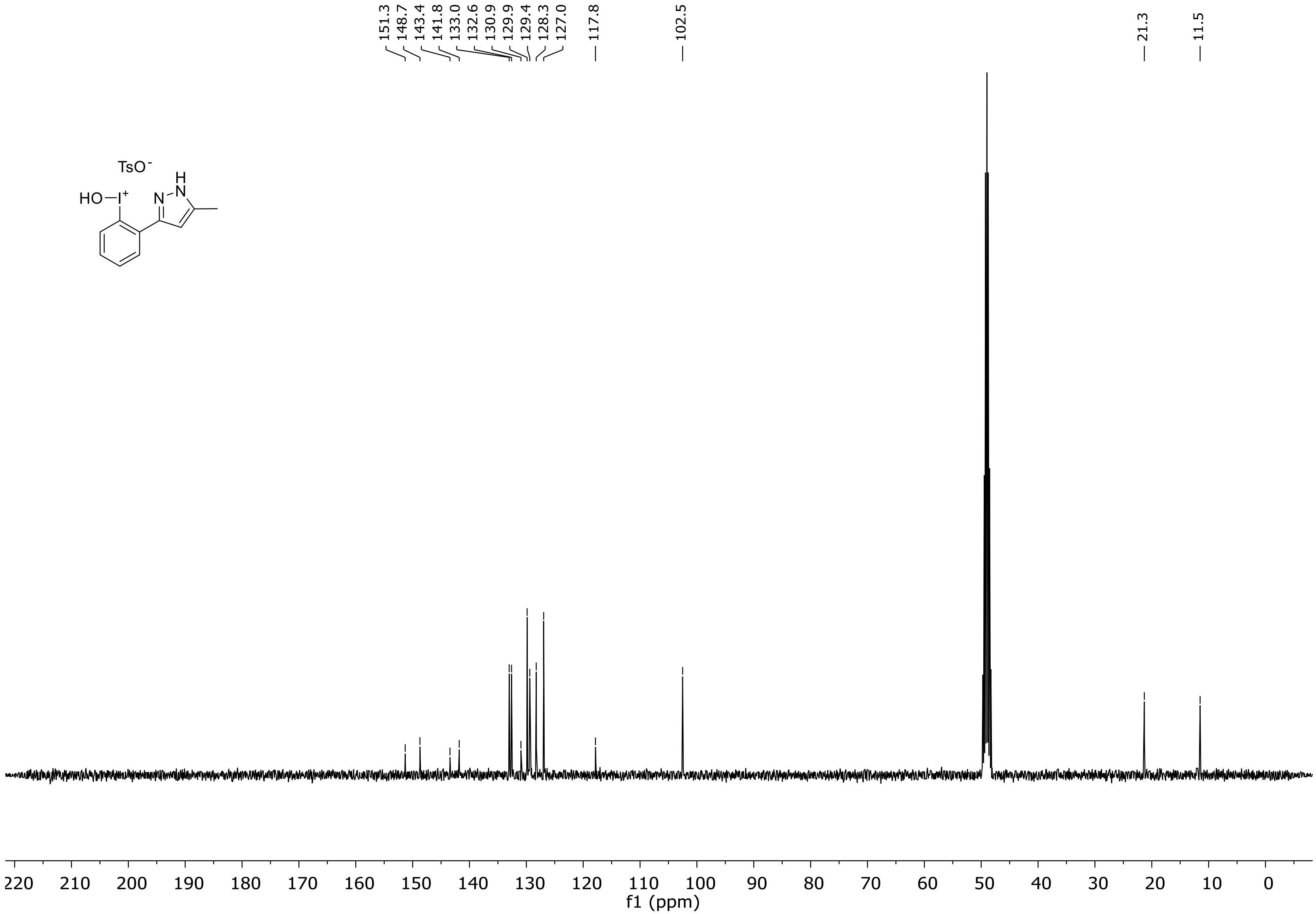

Figure S45: ^{13}C NMR spectra of **6** in d_4-MeOH.

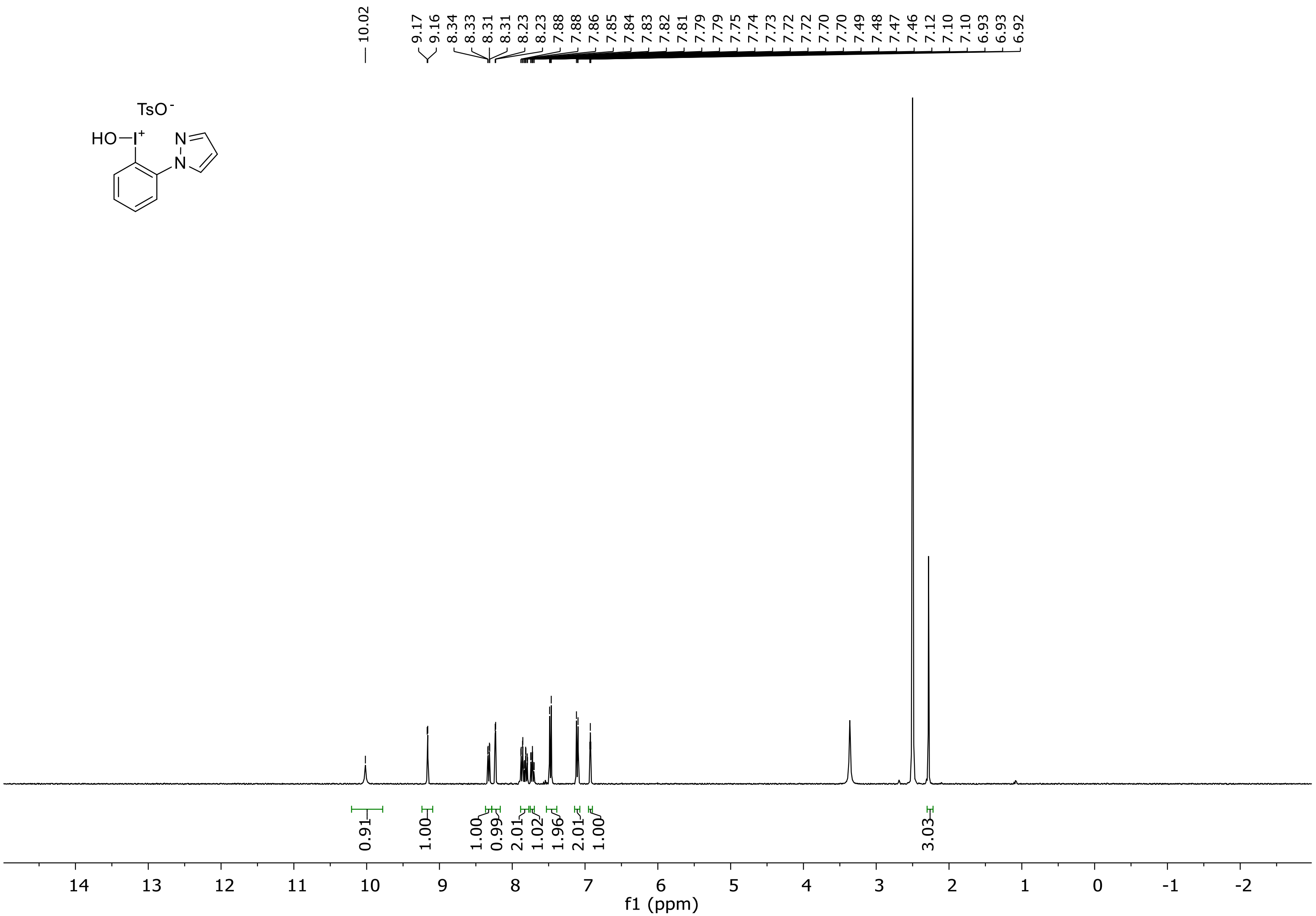

Figure S46: ^{1}H NMR spectra of **7** in d_6-DMSO.

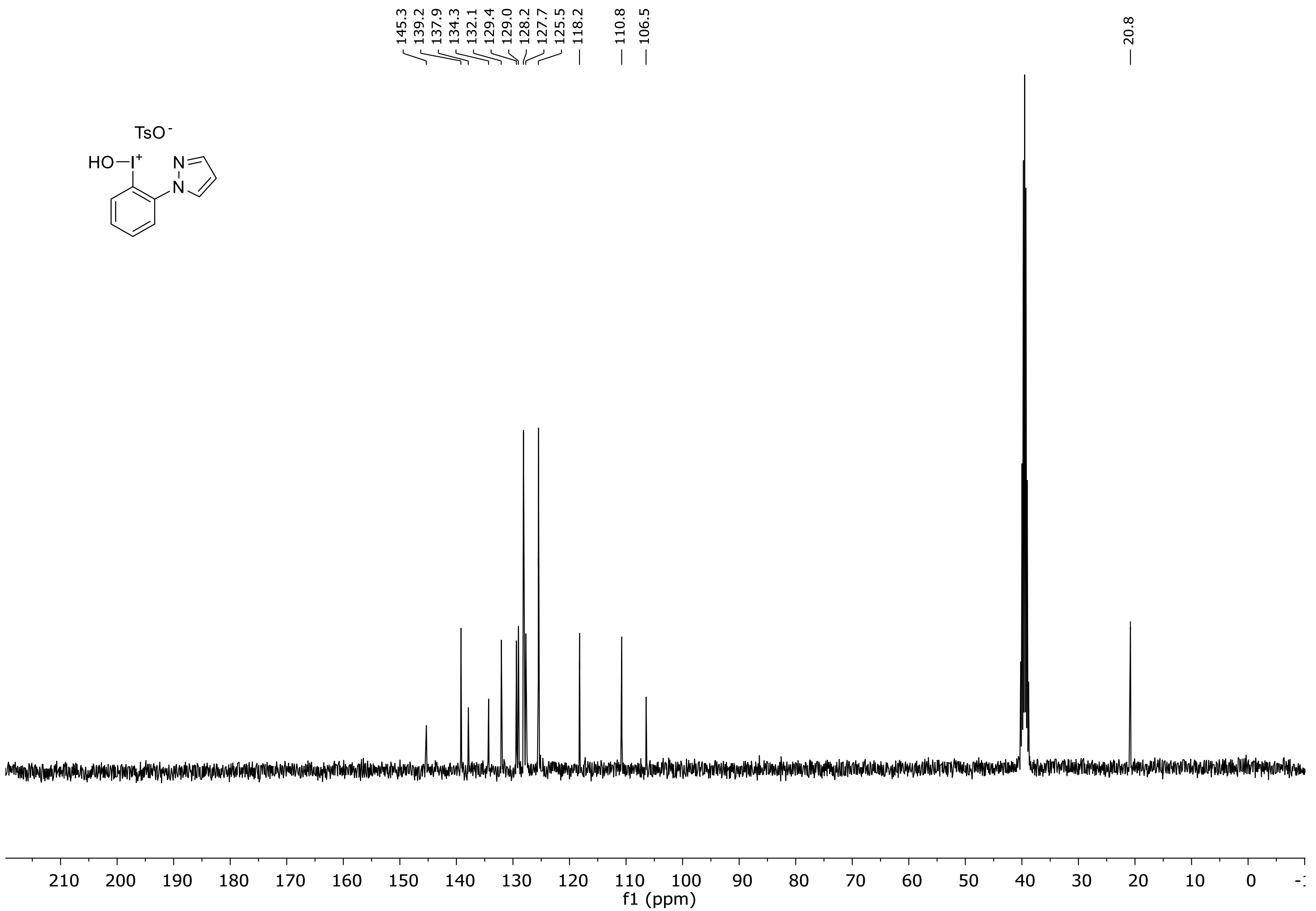

Figure S47: ^{13}C NMR spectra of **7** in *d*$_6$-DMSO.

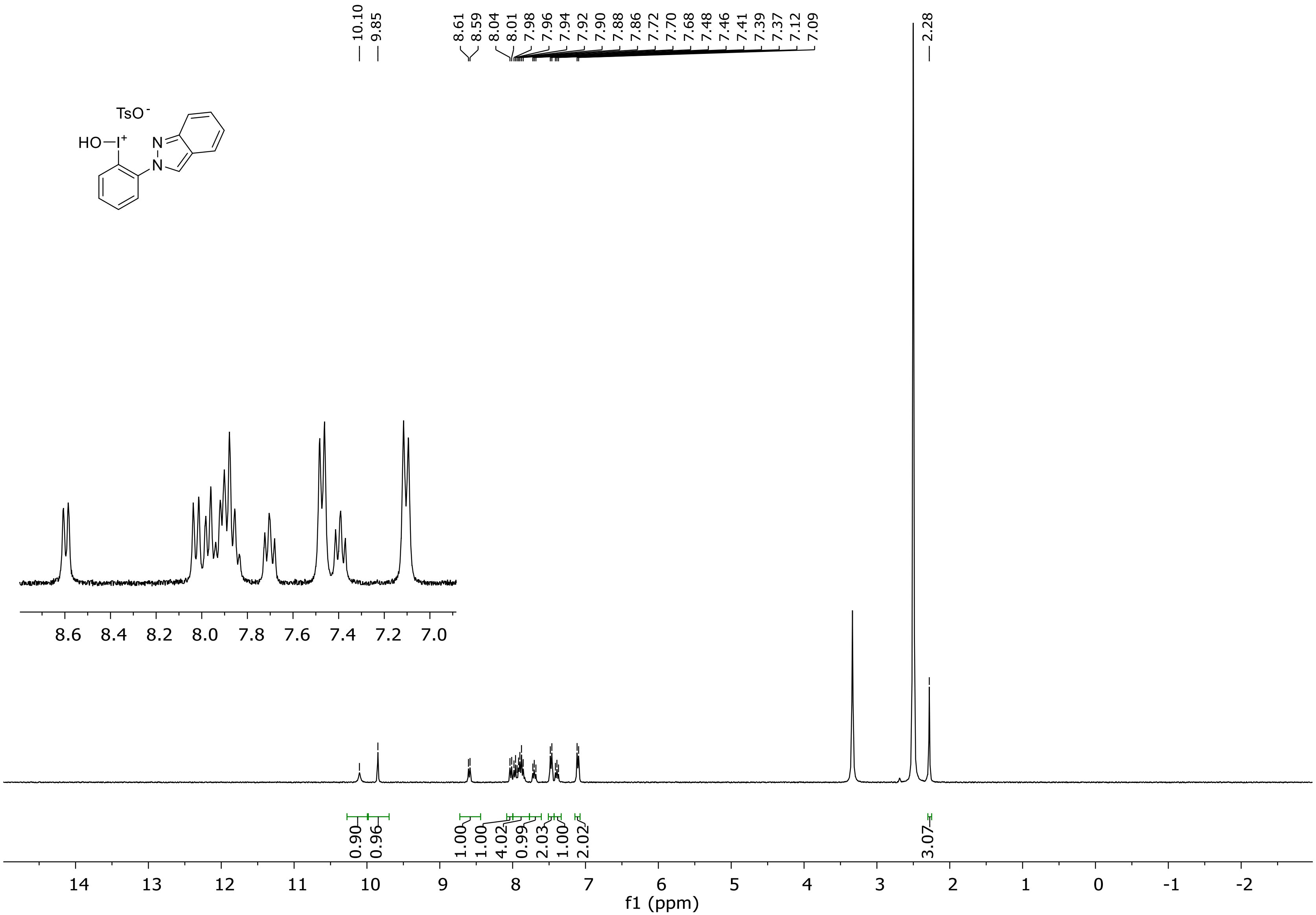

Figure S48: ^{1}H NMR spectra of **8** in d_6-DMSO.

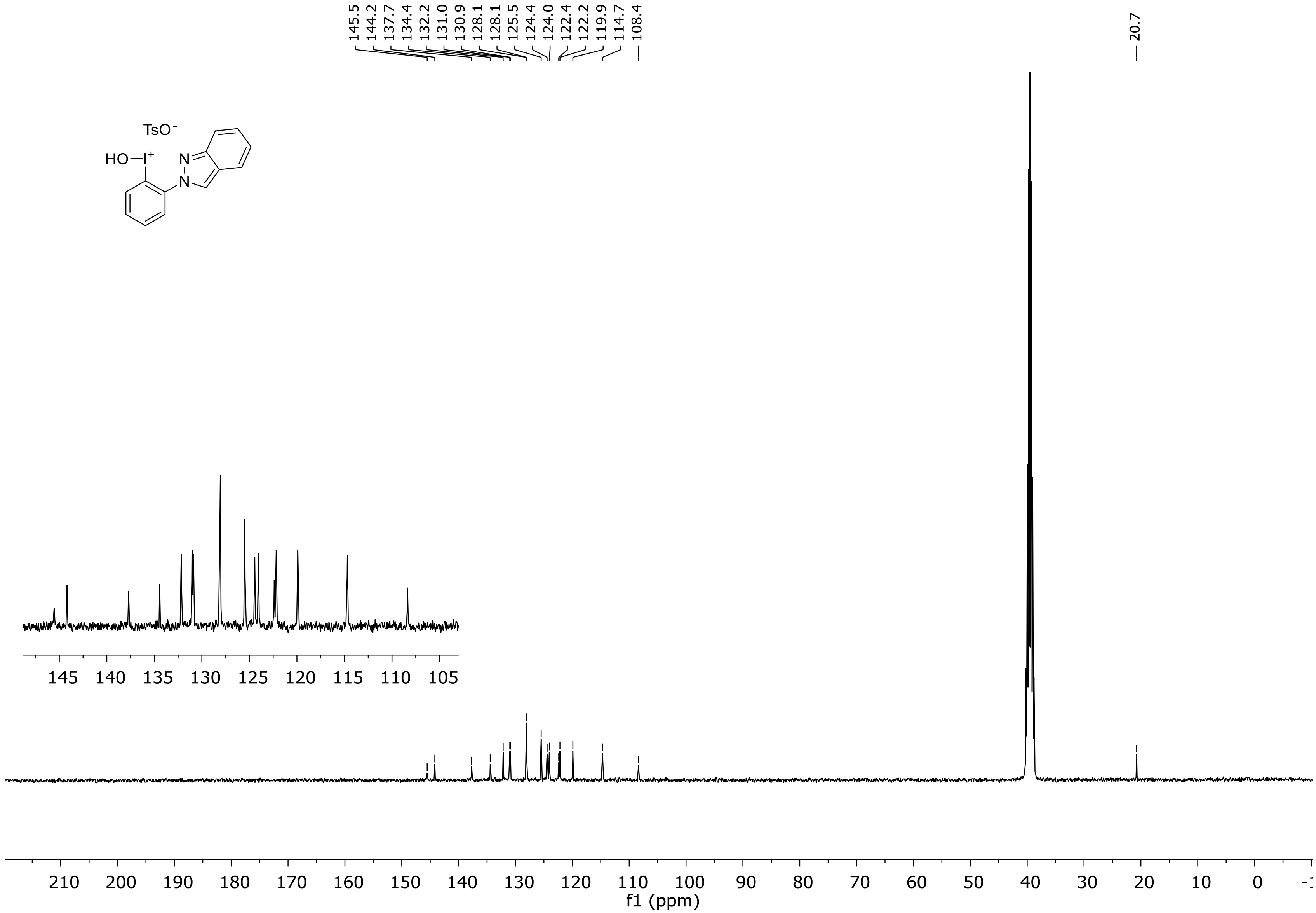

Figure S49: ^{13}C NMR spectra of **8** in d_6-DMSO.

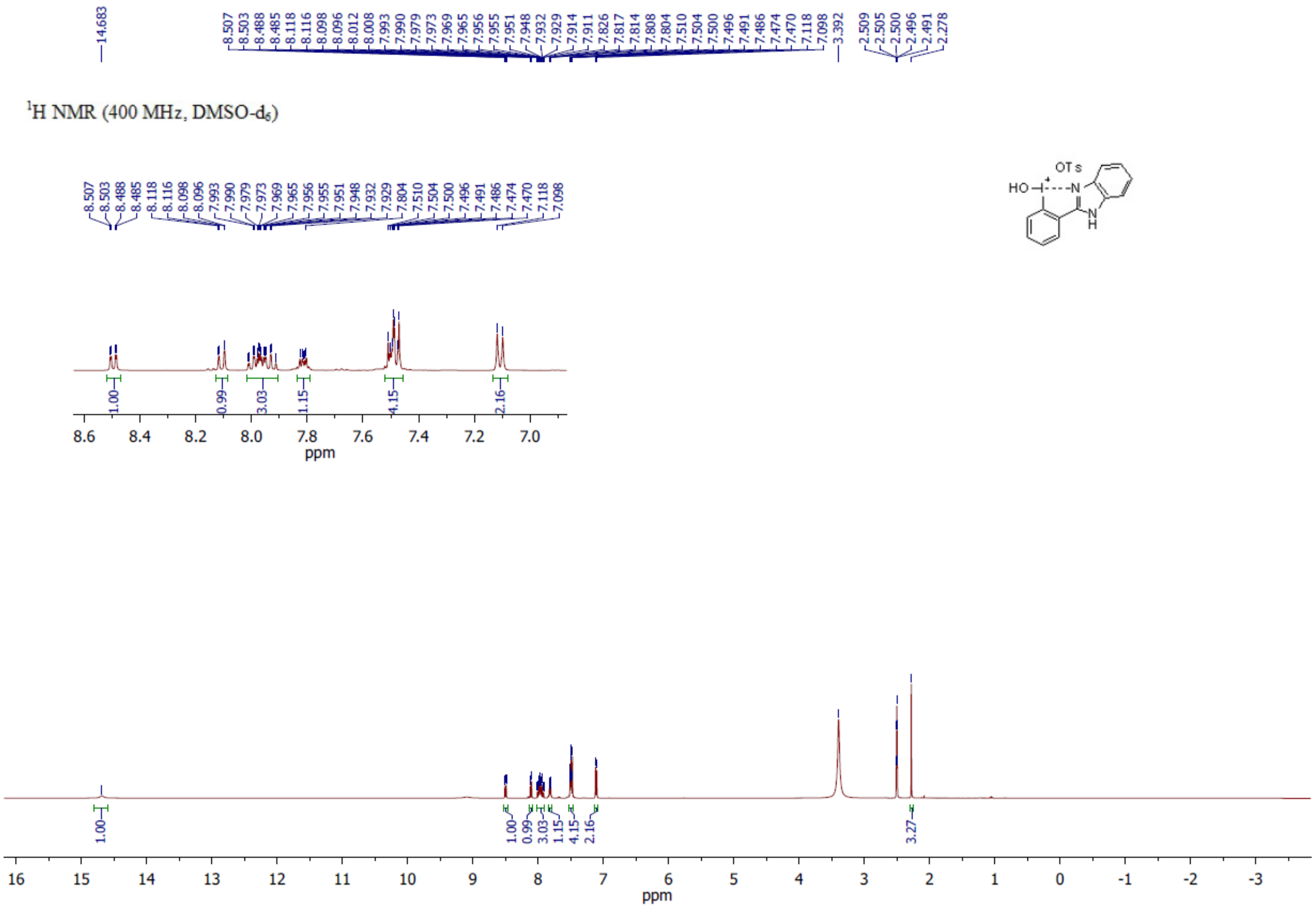

Figure S50: ^{1}H NMR spectra of **9** in d_6-DMSO.

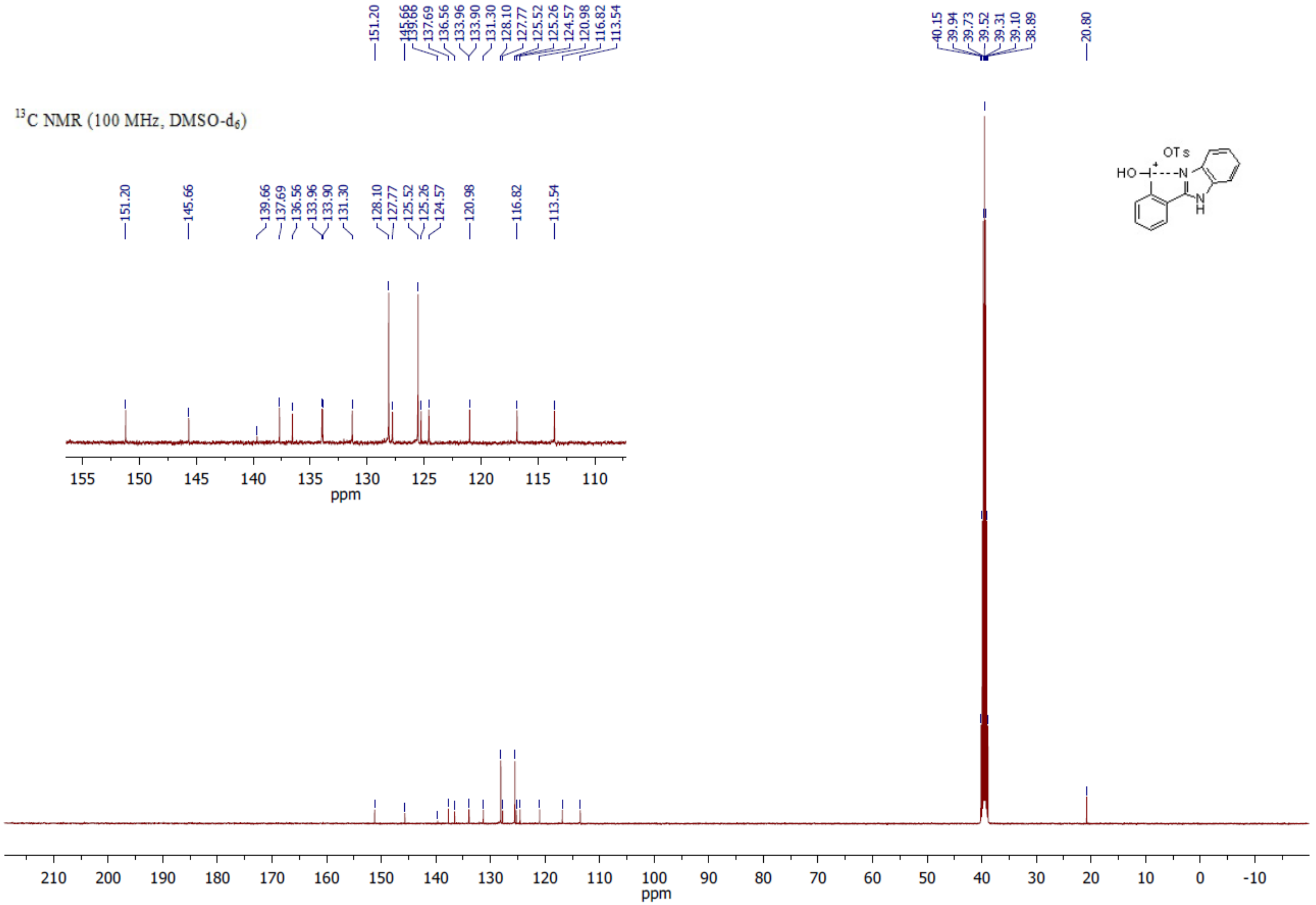

Figure S51: ^{13}C NMR spectra of **9** in d_6-DMSO.

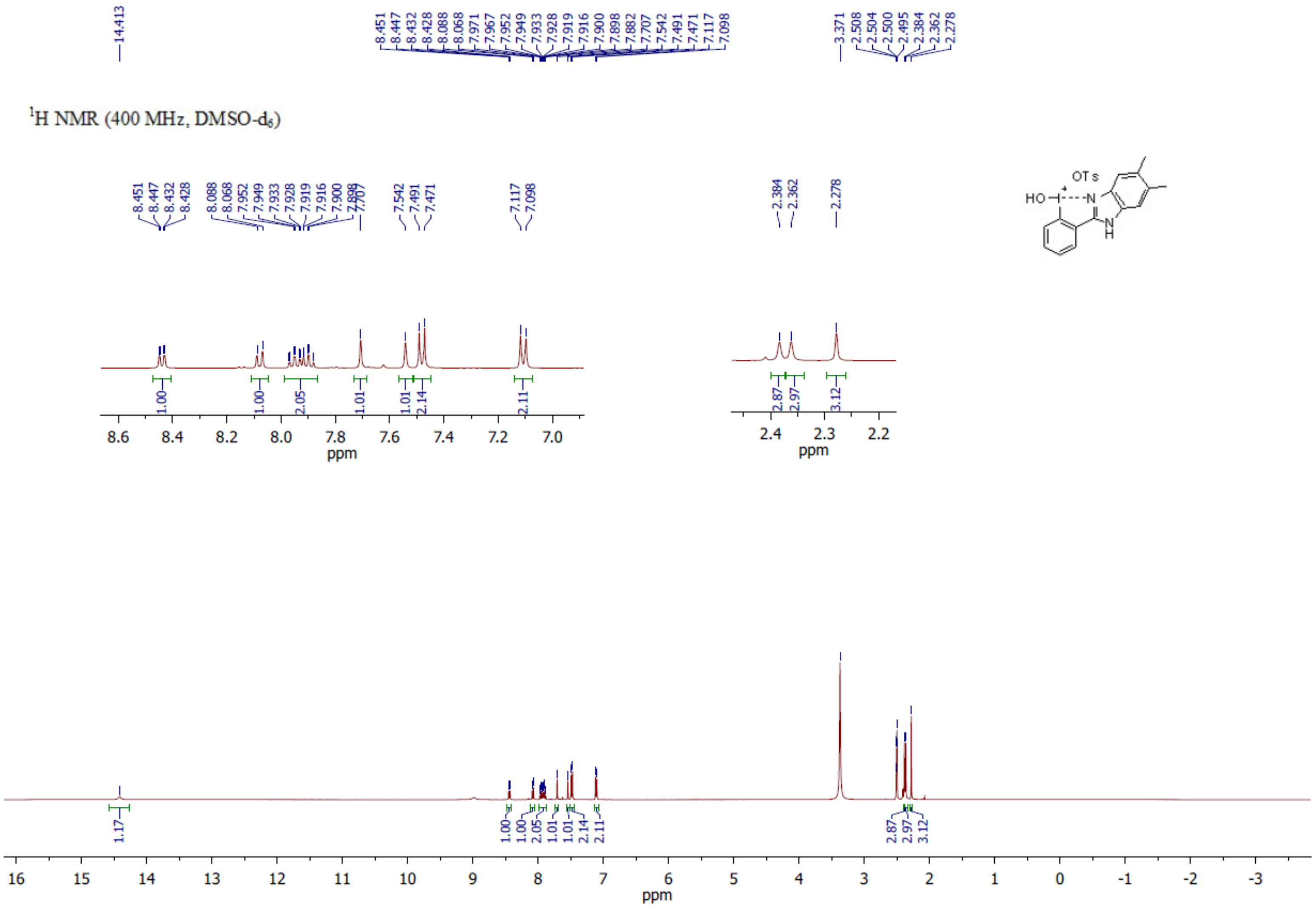

Figure S52: ^{1}H NMR spectra of **10** in d_6-DMSO.

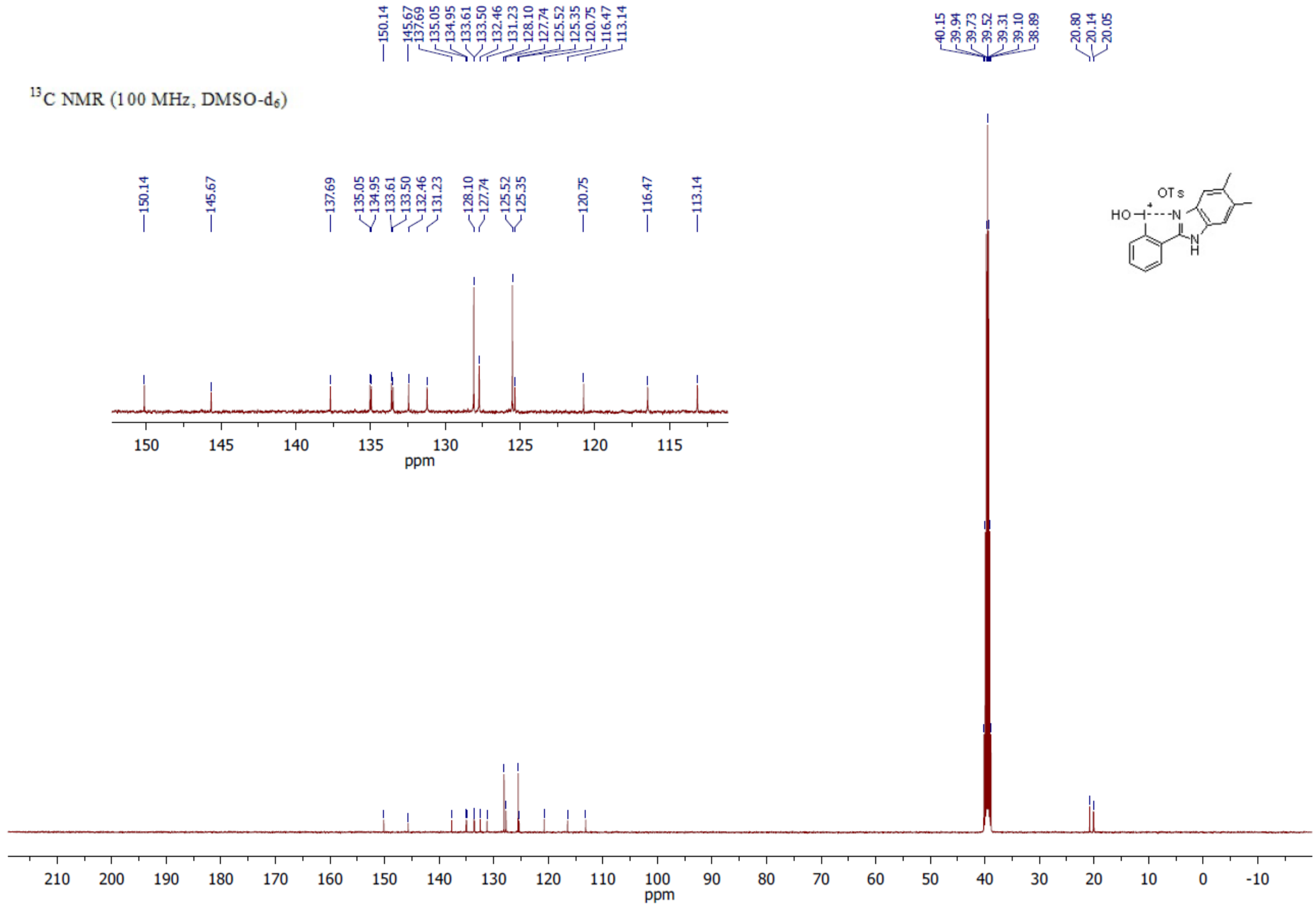

Figure S53: ^{13}C NMR spectra of **10** in d_6-DMSO.

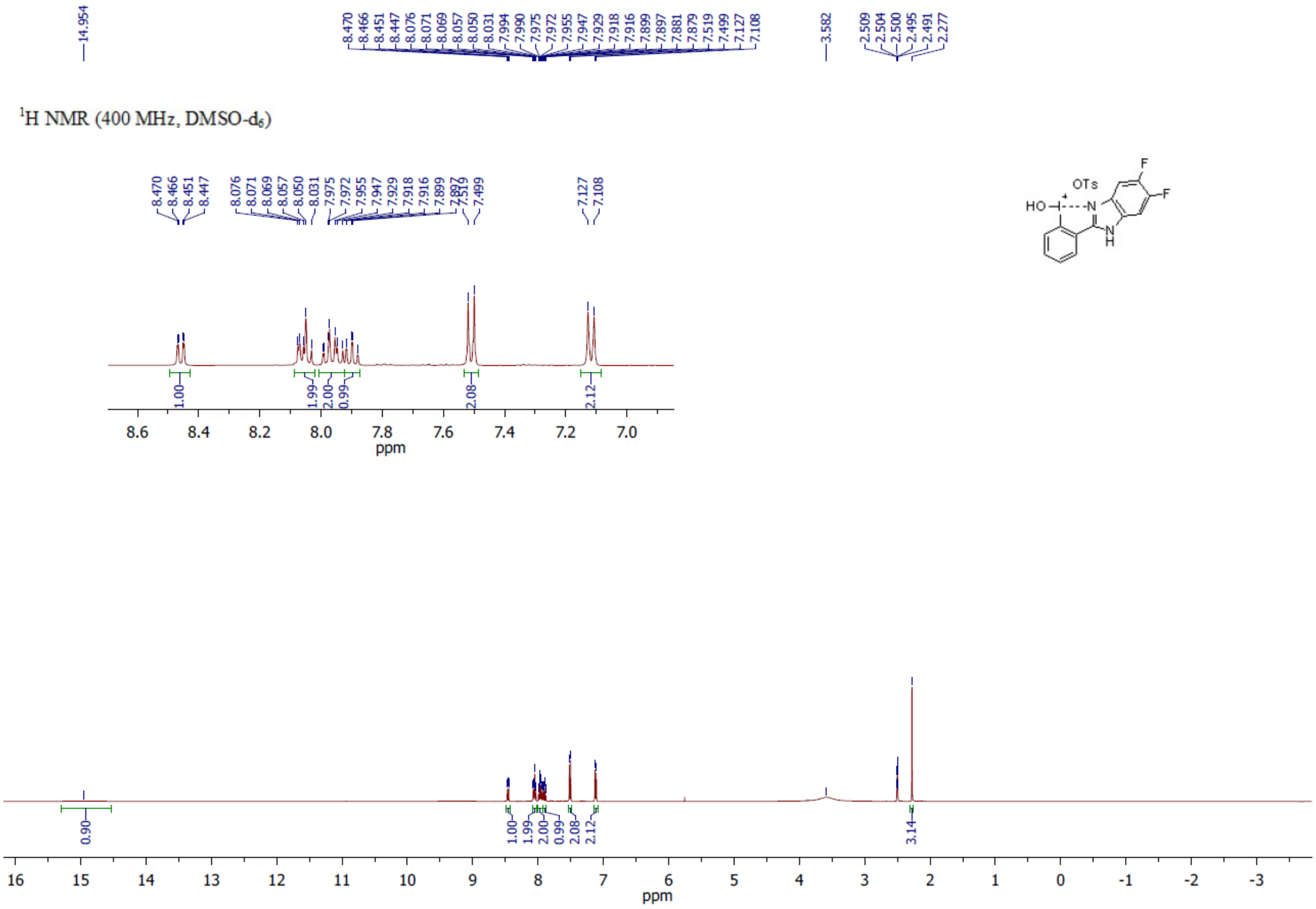

Figure S54: ^{1}H NMR spectra of **11** in d_6-DMSO.

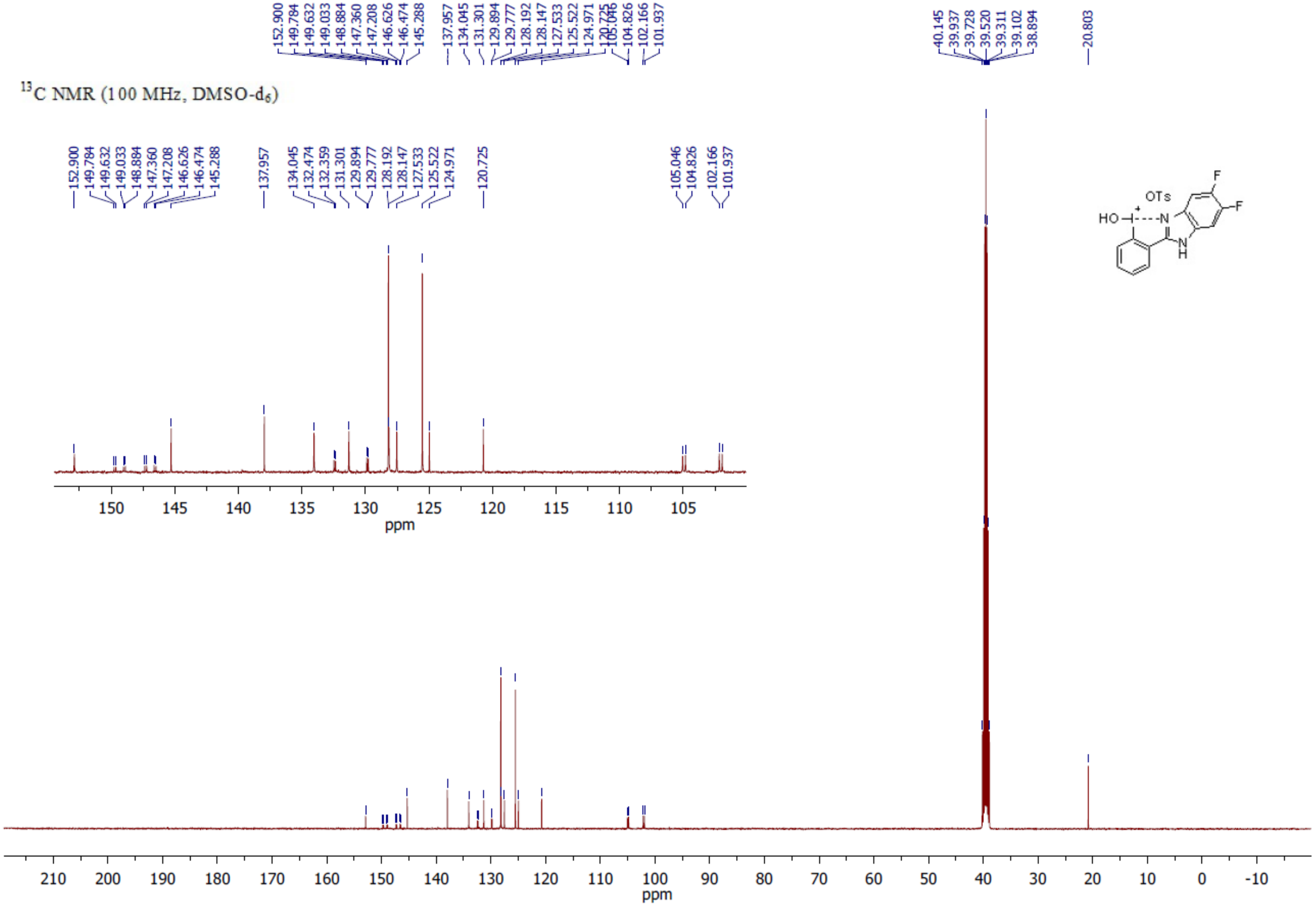

Figure S55: ^{13}C NMR spectra of **11** in d_6-DMSO.

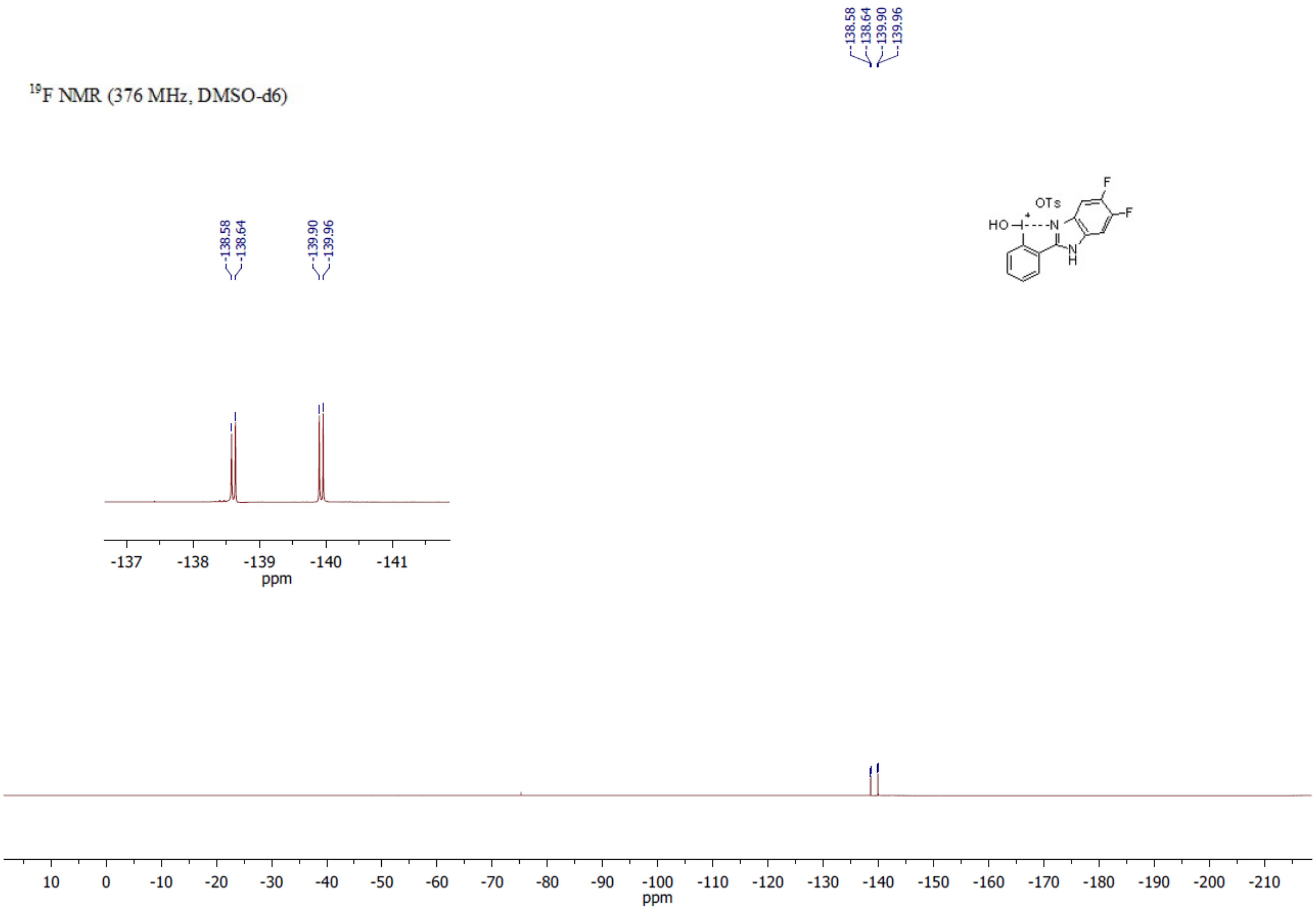

Figure S56: ^{19}F NMR spectra of **11** in d_6-DMSO.

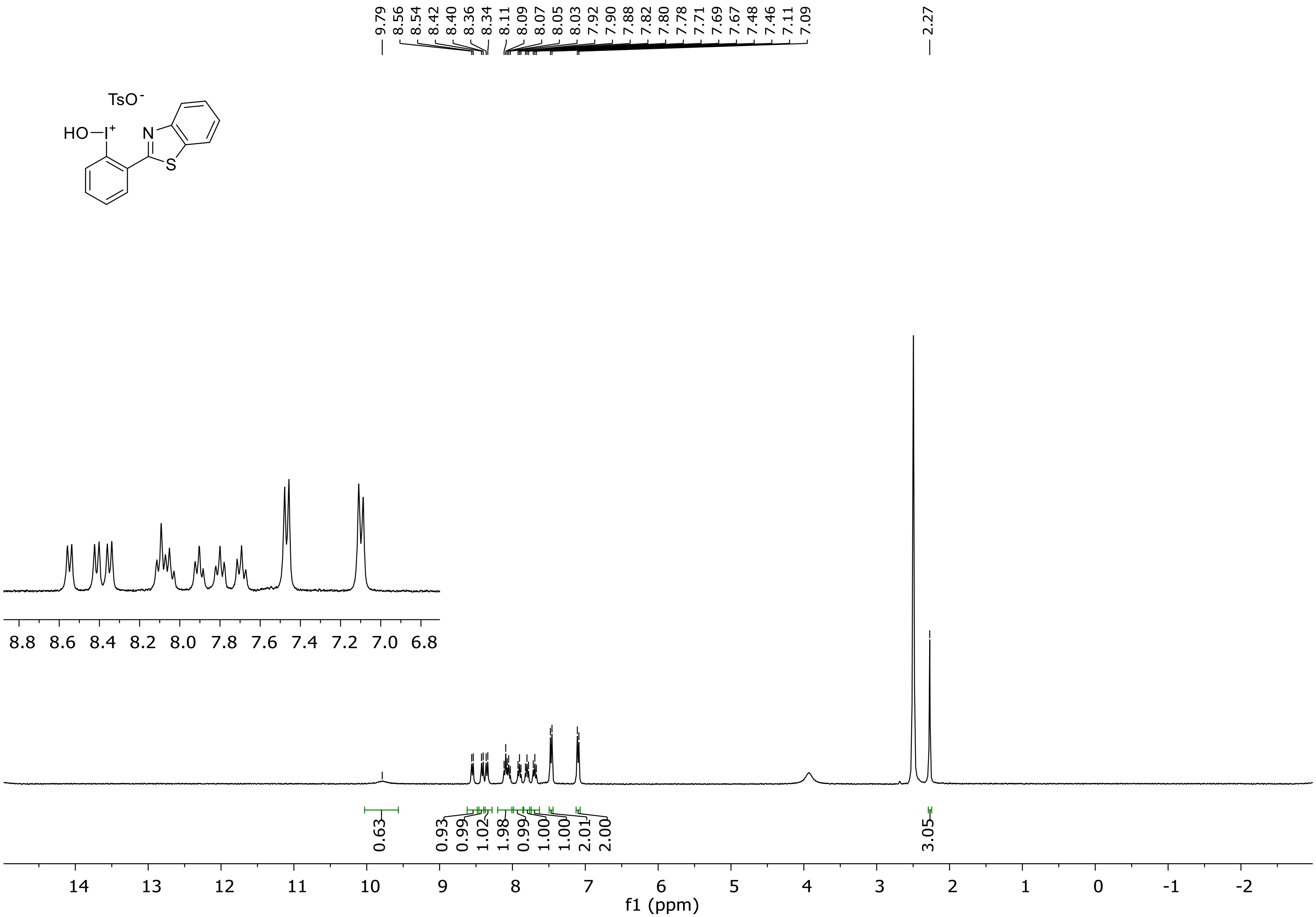

Figure S57: ^{1}H NMR spectra of **12** in d_6-DMSO.

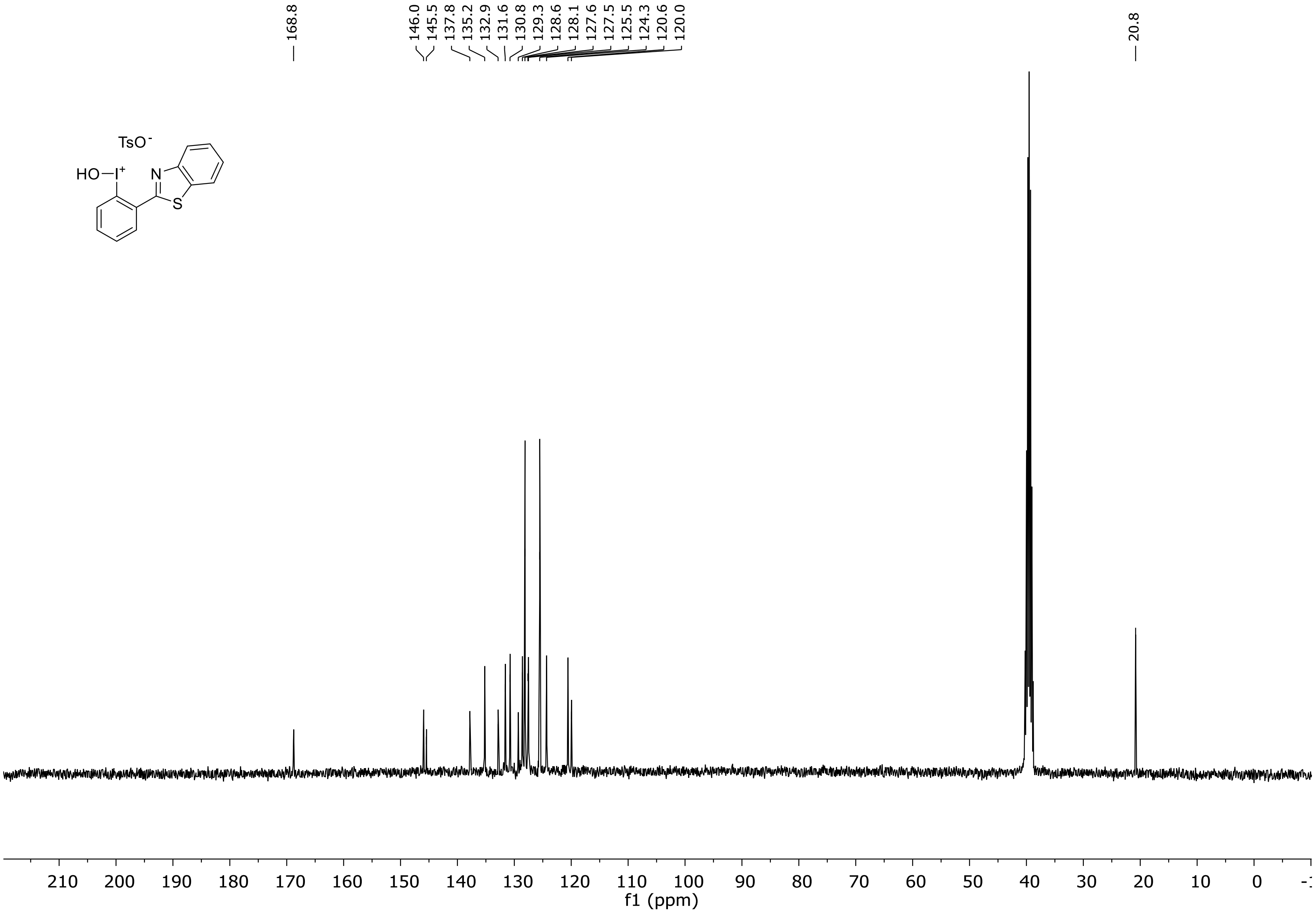

Figure S58: ^{13}C NMR spectra of **12** in d_6-DMSO.

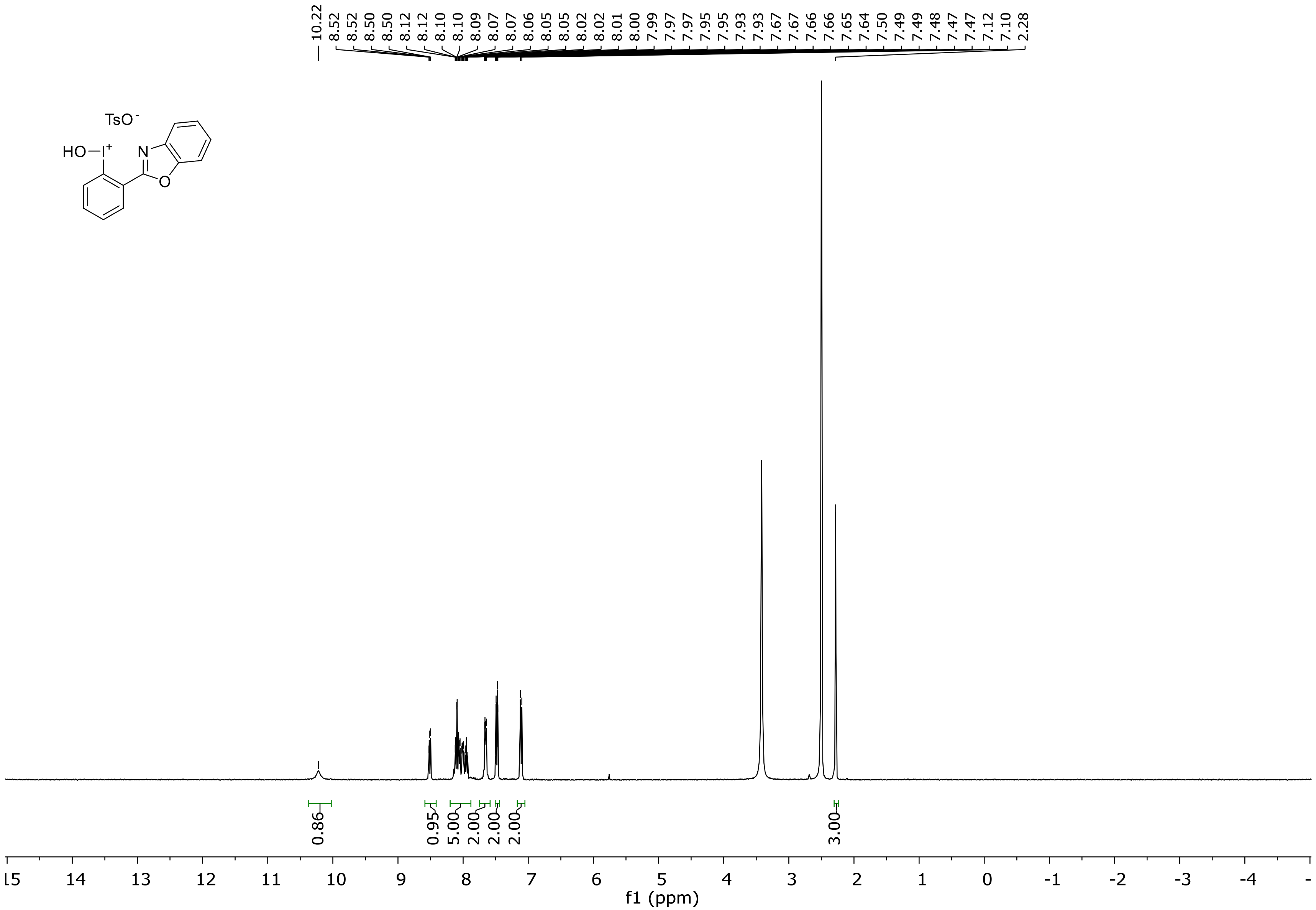

Figure S59: ^{1}H NMR spectra of **13** in d_6-DMSO.

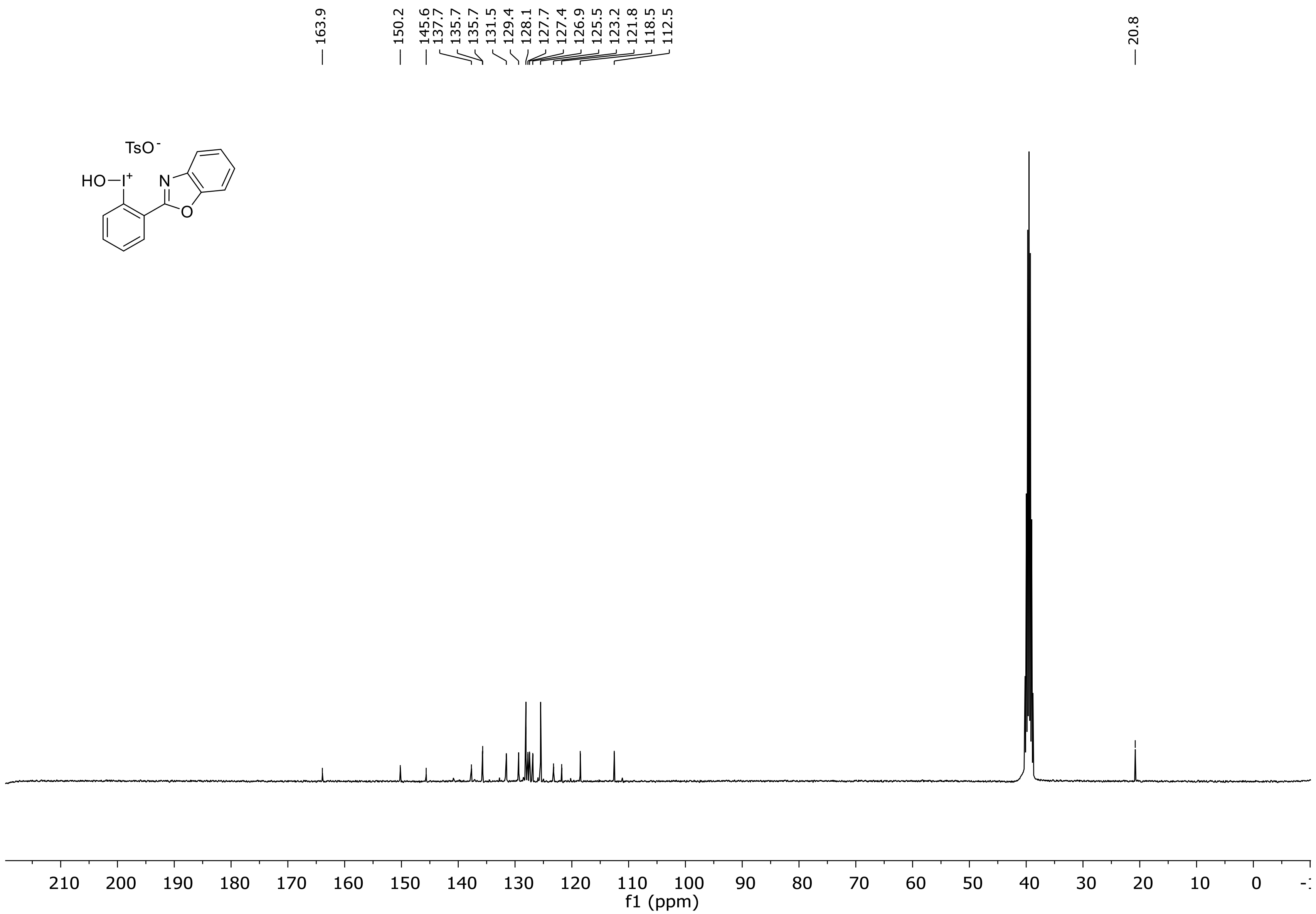

Figure S60: ^{13}C NMR spectra of **13** in d_6-DMSO.

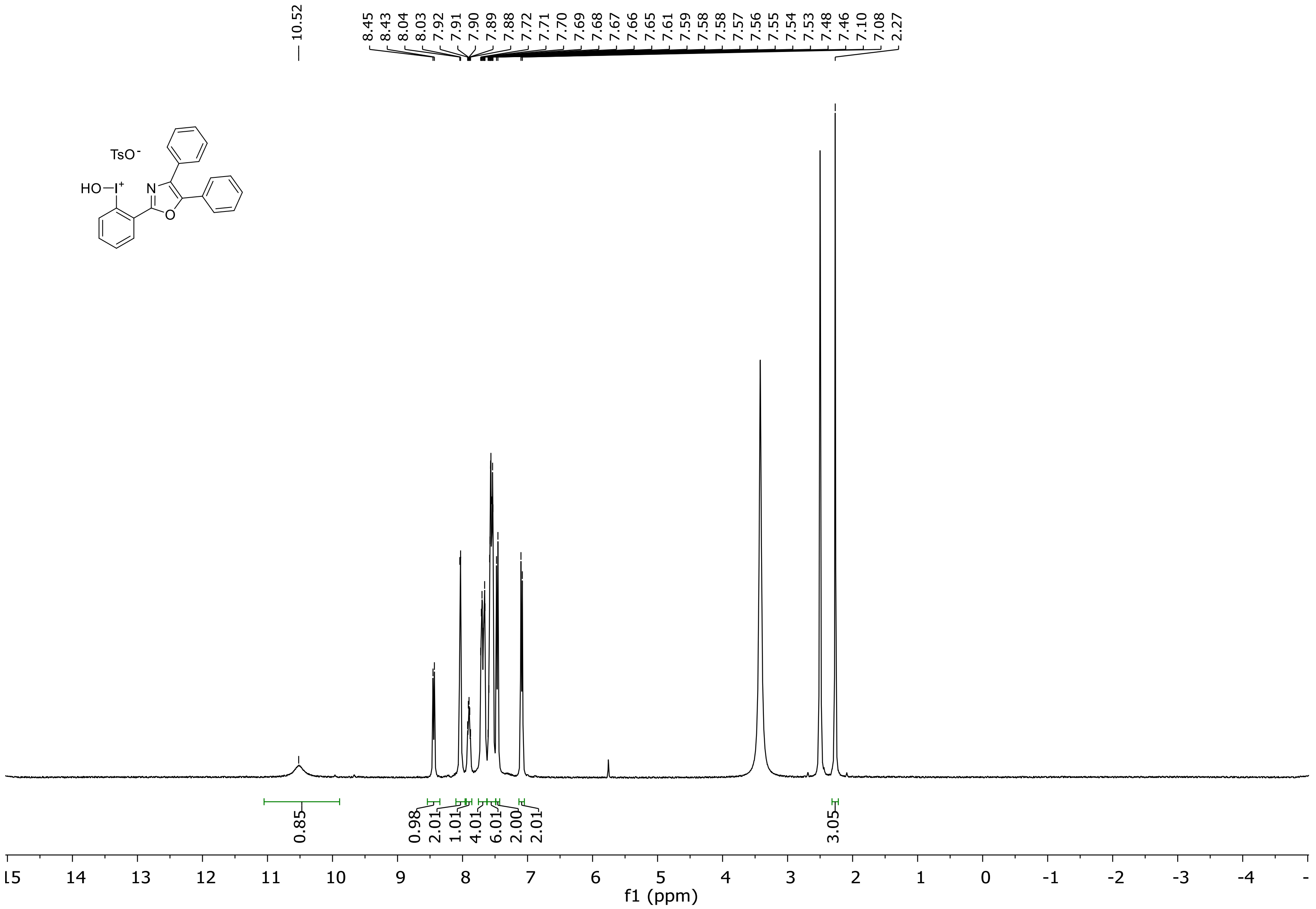

Figure S61: ^{1}H NMR spectra of **14** in d_6-DMSO.

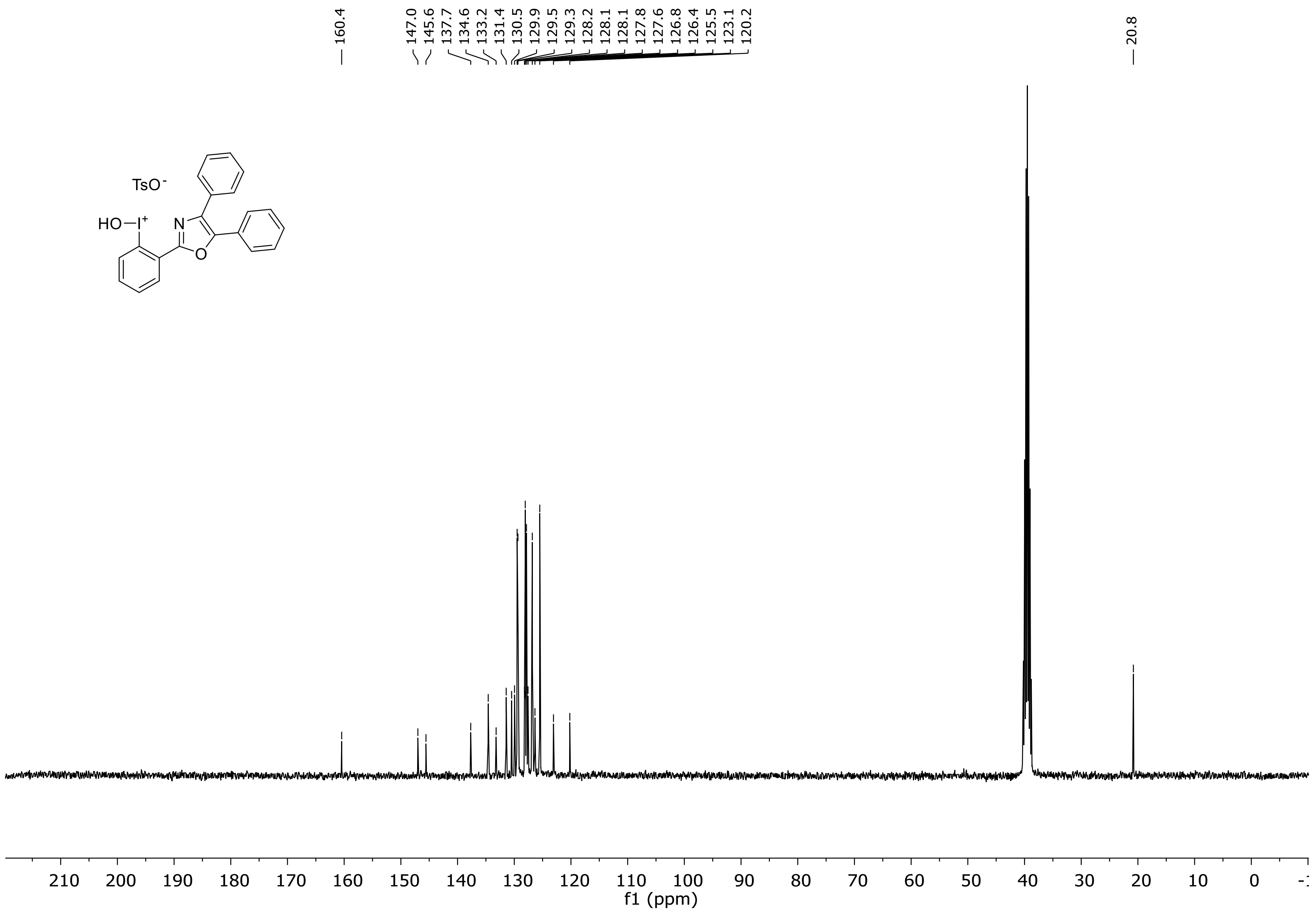

Figure S62: ^{13}C NMR spectra of **14** in d_6-DMSO.

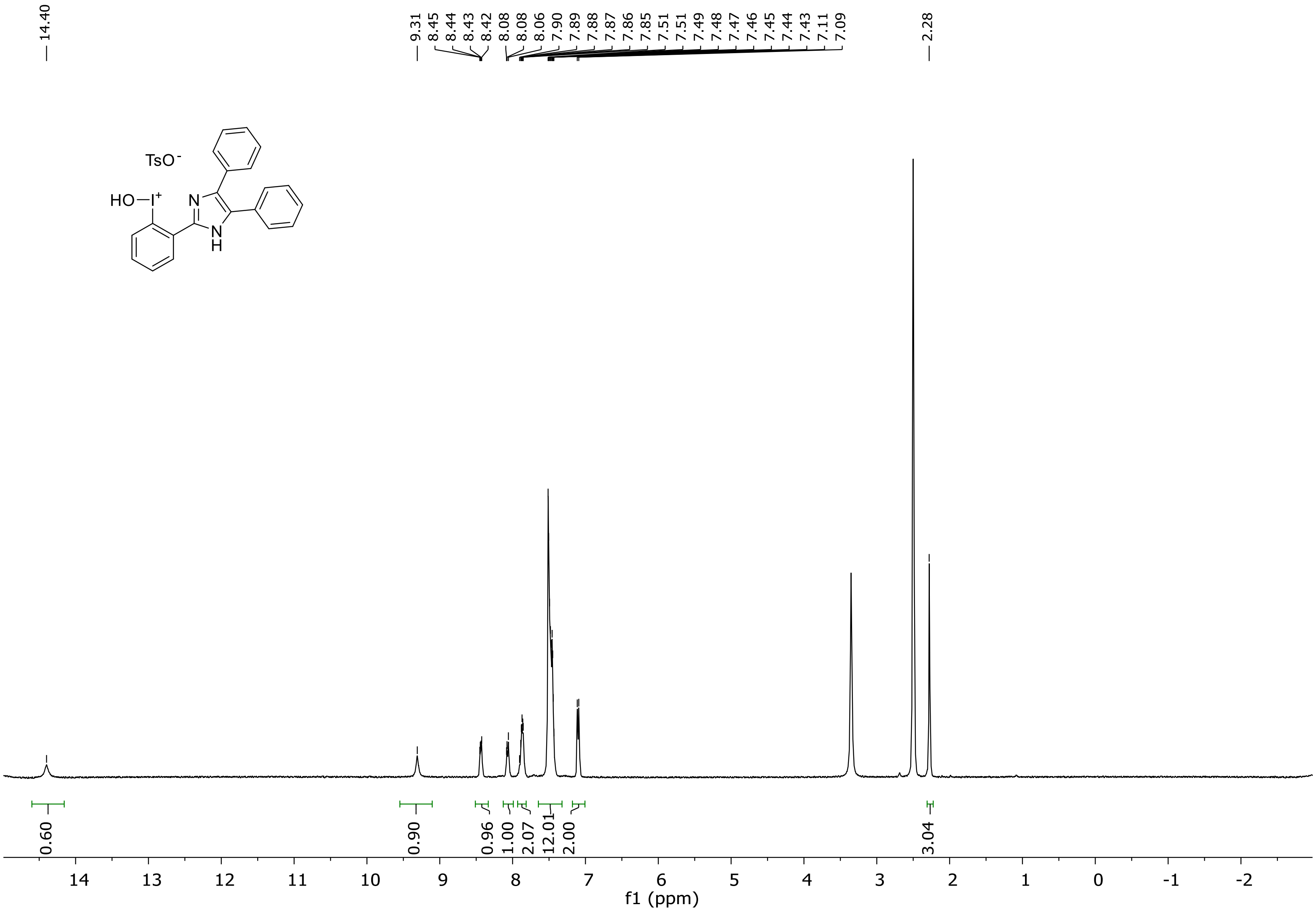

Figure S63: ^{1}H NMR spectra of **15** in d_6-DMSO.

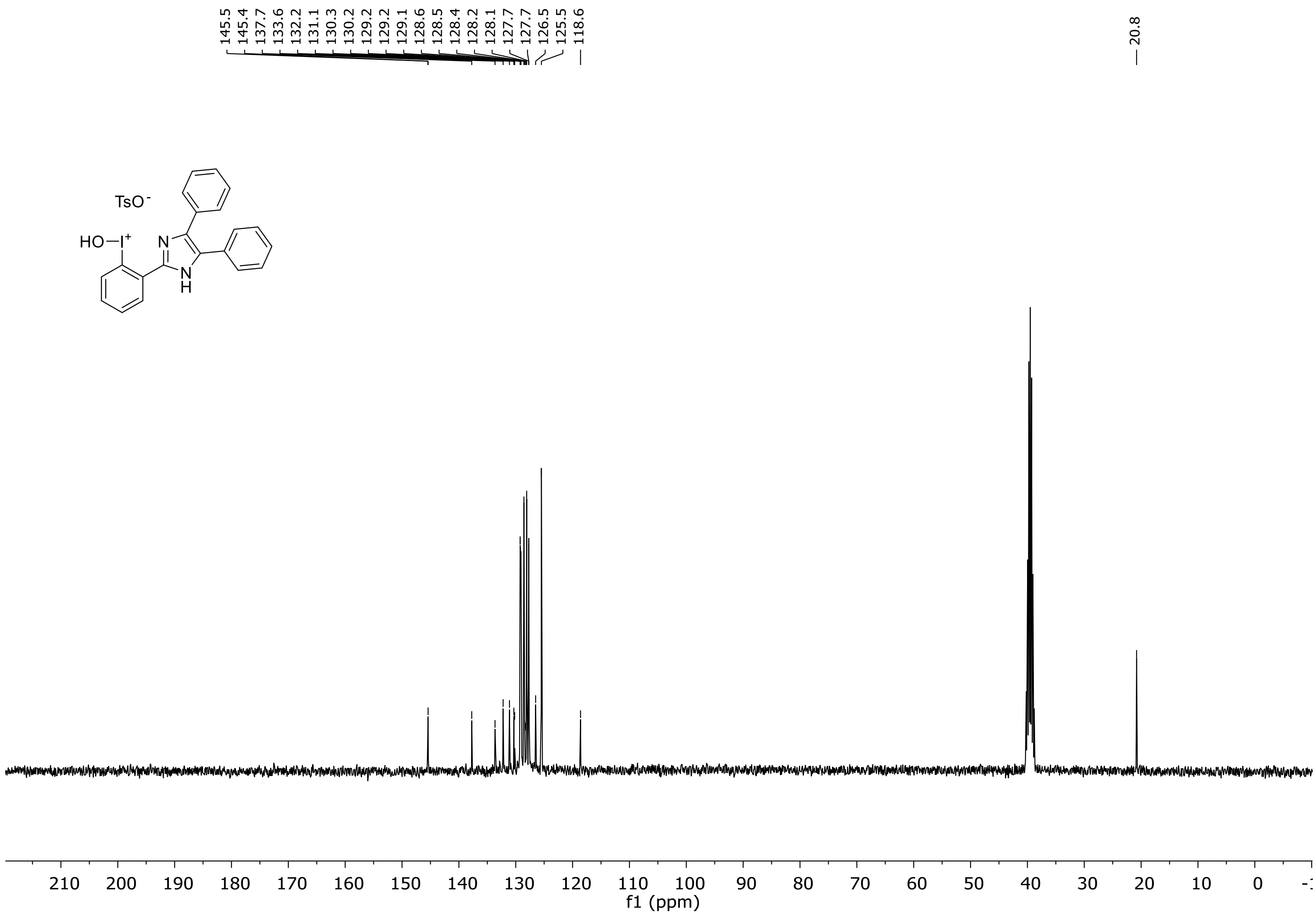

Figure S64: ^{13}C NMR spectra of **15** in d_6-DMSO.

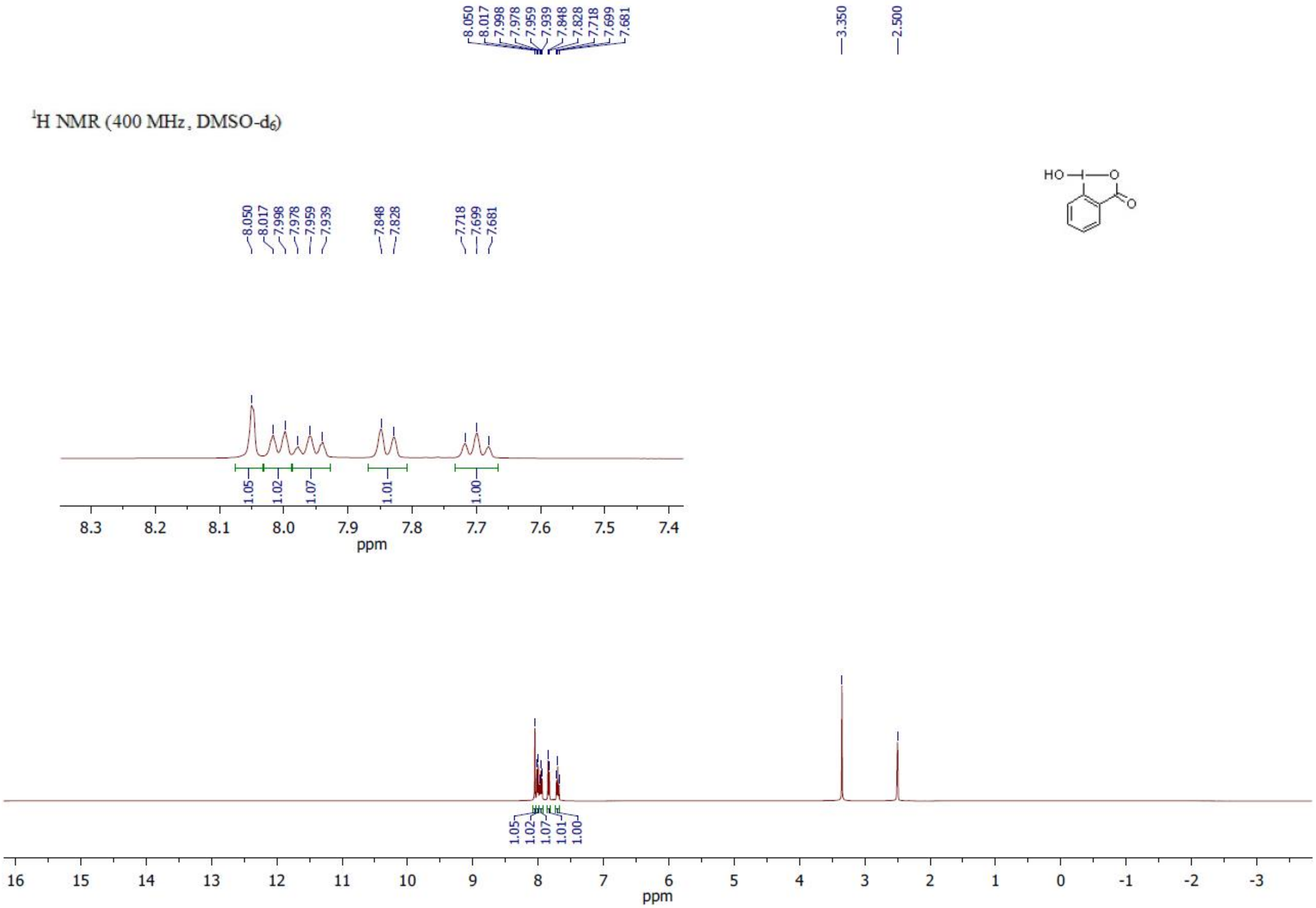

Figure S65: ^{1}H NMR spectra of **16** in d_6-DMSO.

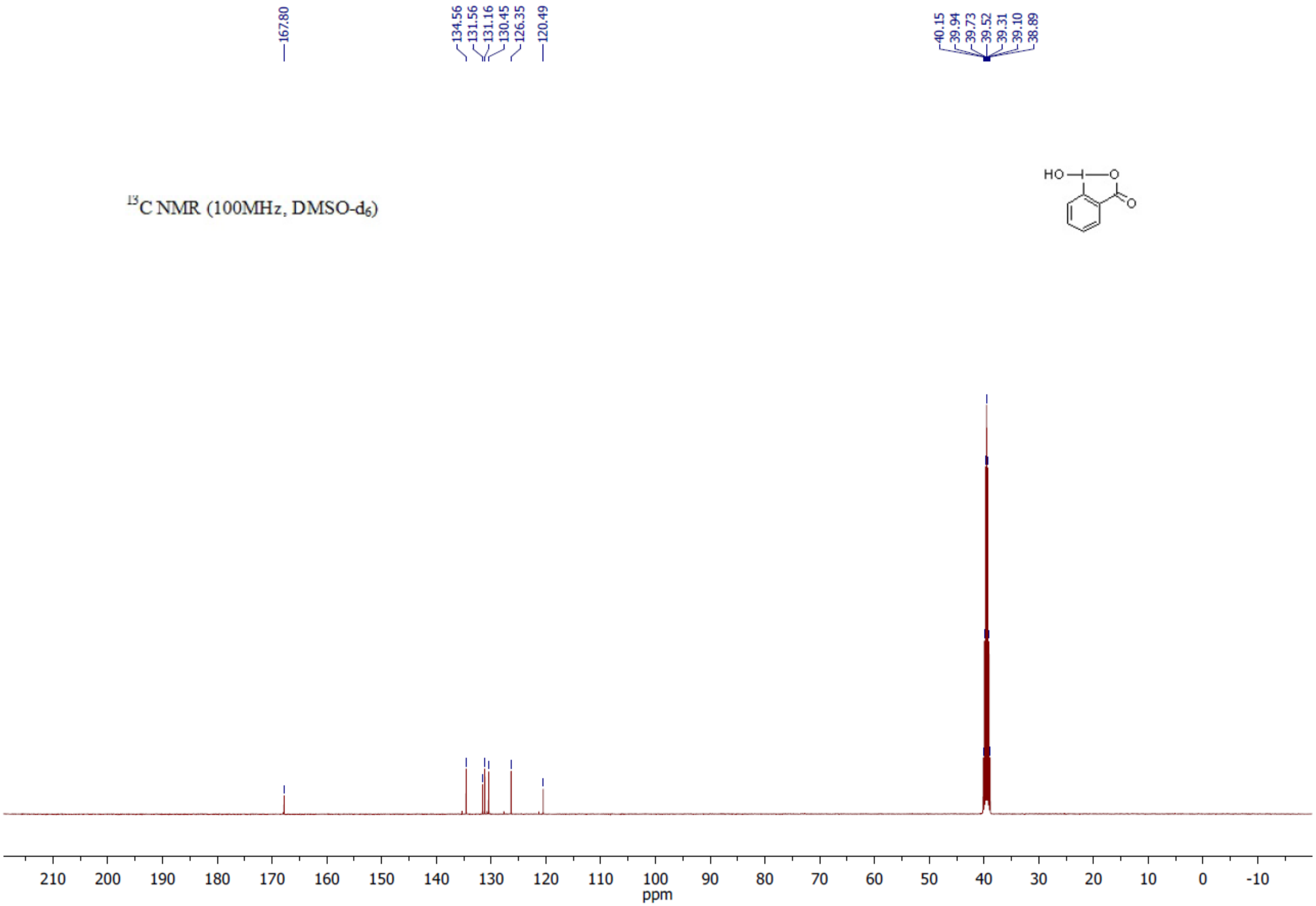

Figure S66: ^{13}C NMR spectra of **16** in d_6-DMSO.

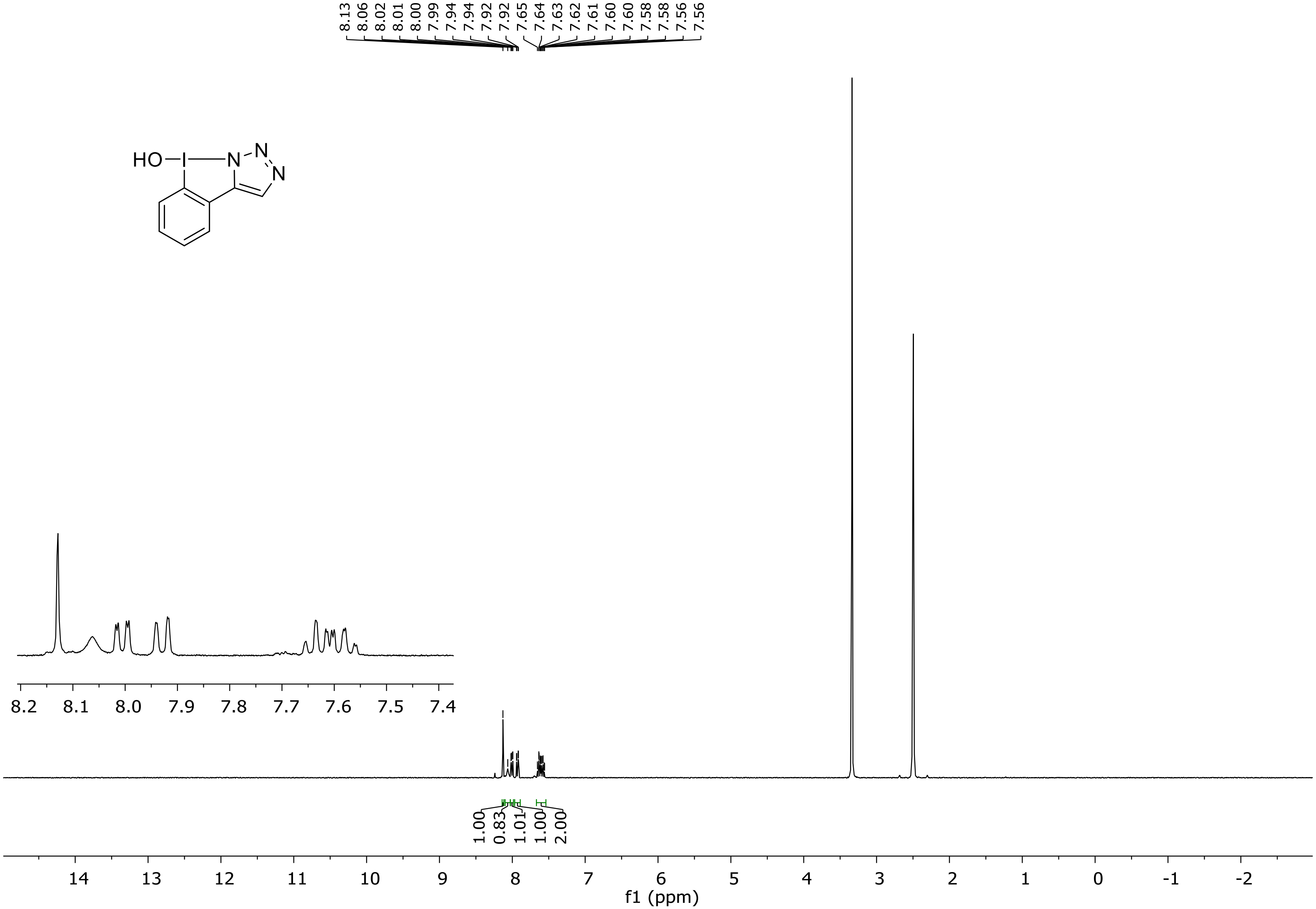

Figure S67: ^{1}H NMR spectra of **17** in d_4-MeOH.

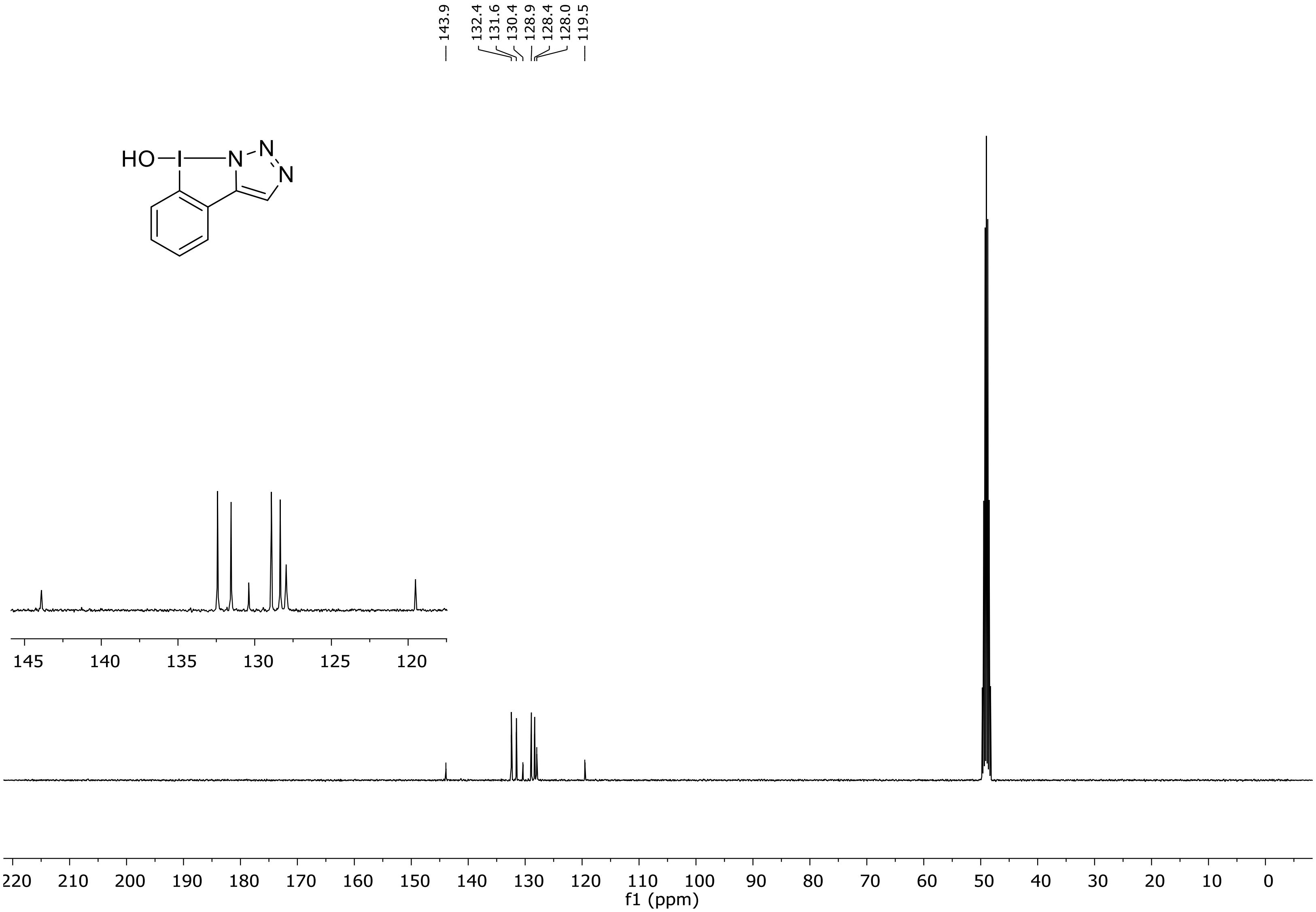

Figure S68: ^{13}C NMR spectra of **17** in d_4-MeOH.

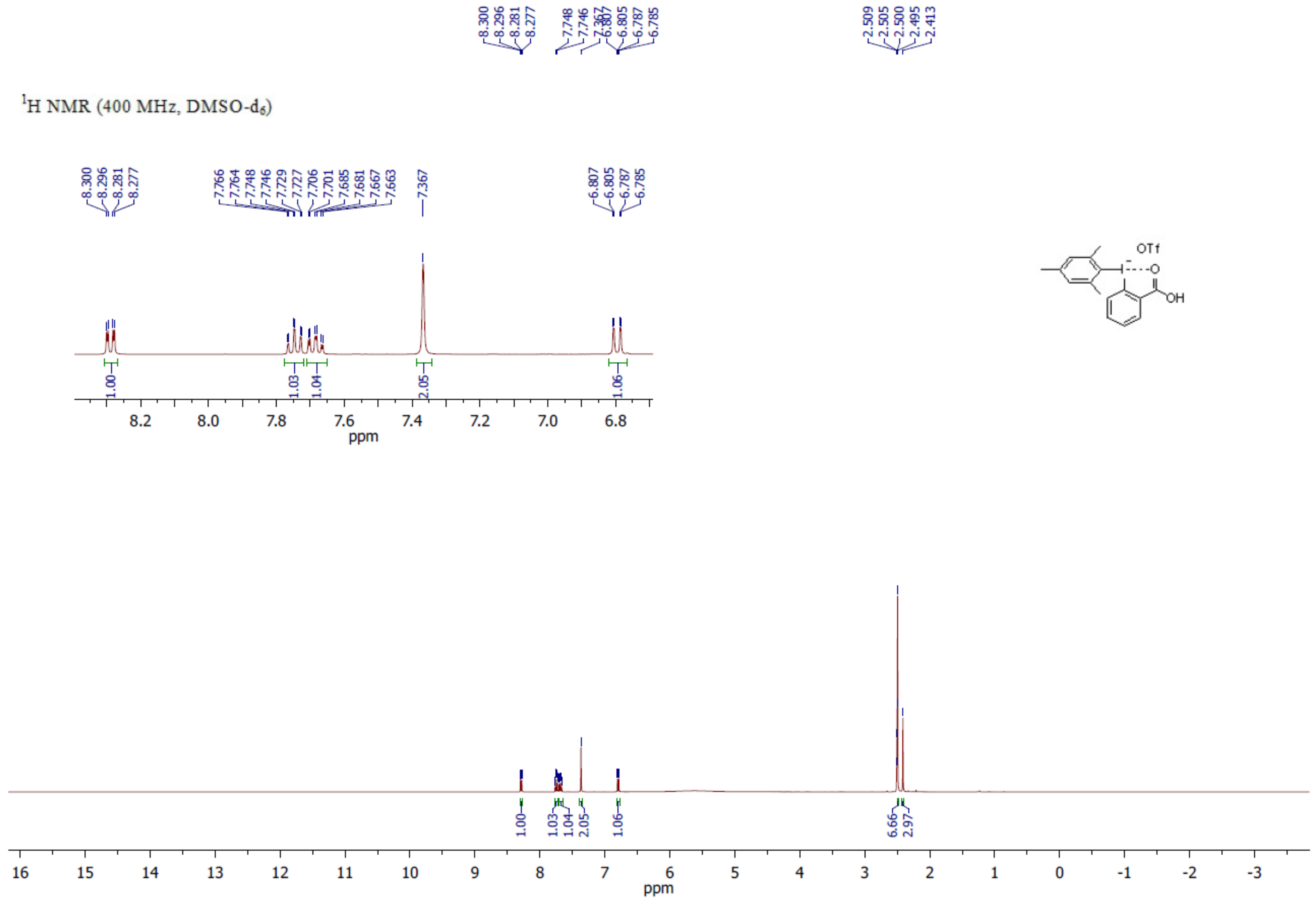

Figure S69: ^{1}H NMR spectra of **18** in d_6-DMSO.

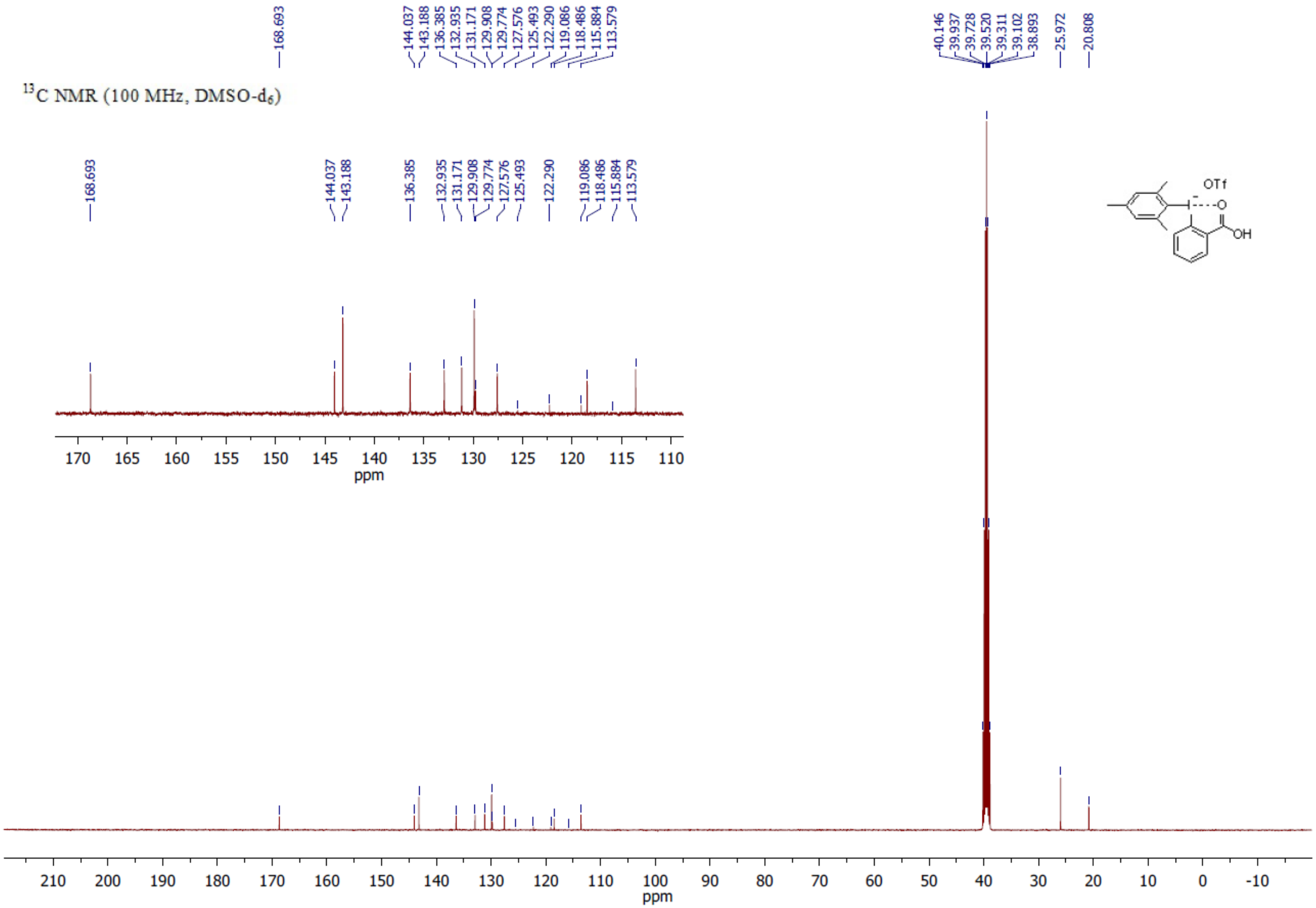

Figure S70: ^{13}C NMR spectra of **18** in d_6-DMSO.

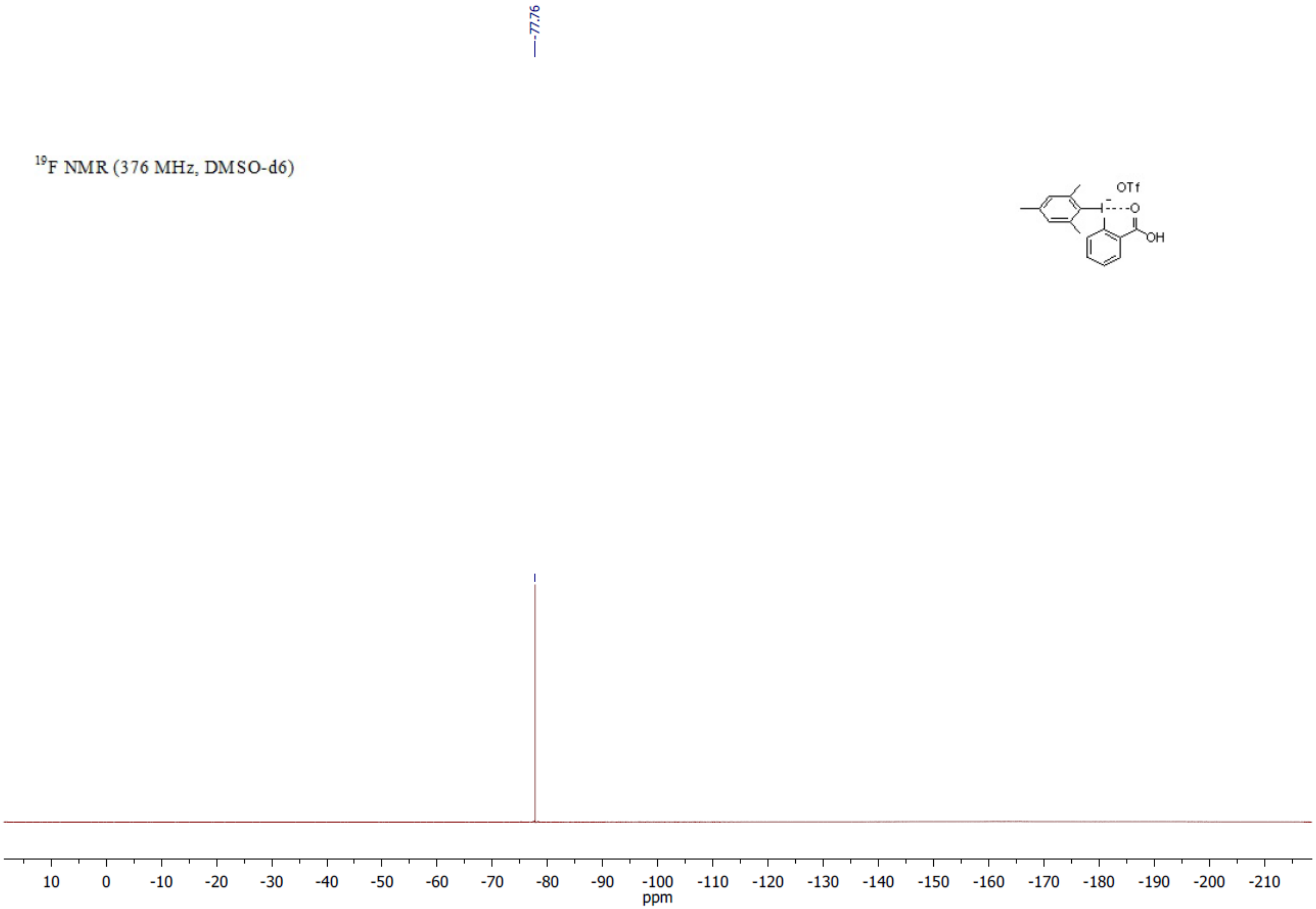

Figure S71: ^{19}F NMR spectra of **18** in d_6-DMSO.

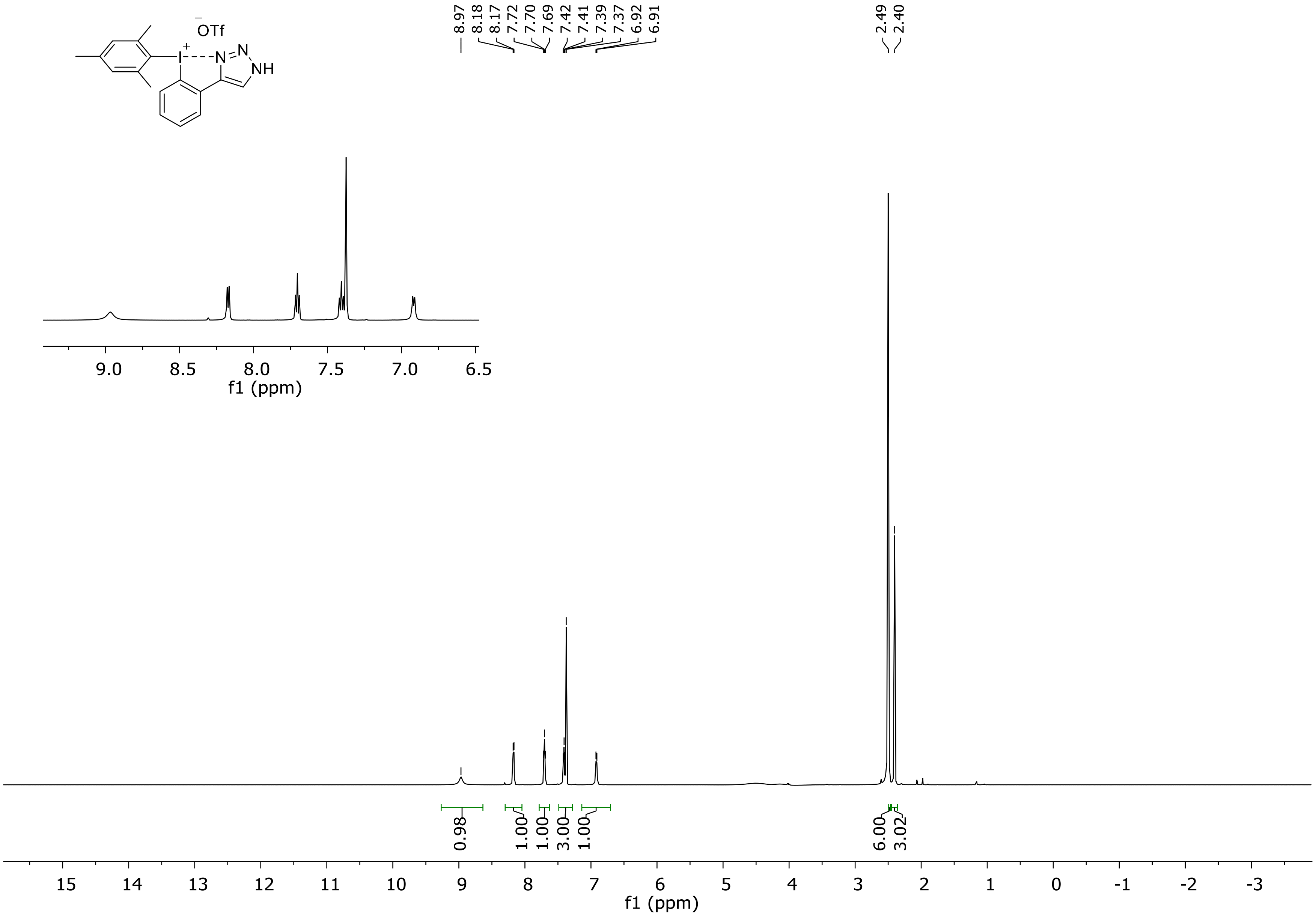

Figure S72: ^{1}H NMR spectra of **19** in d_6-DMSO.

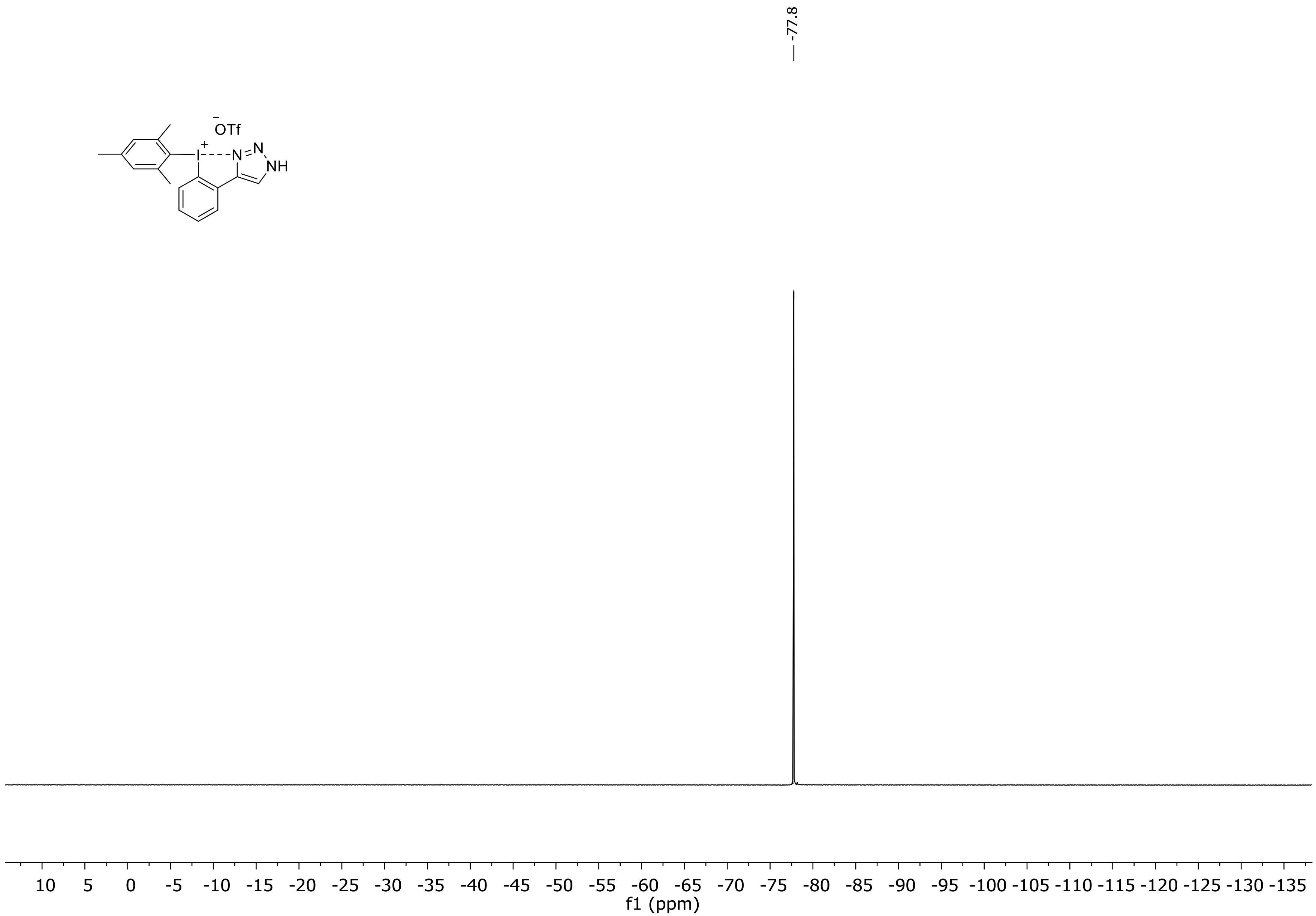

Figure S73: ^{19}F NMR spectra of **19** in d_6-DMSO.

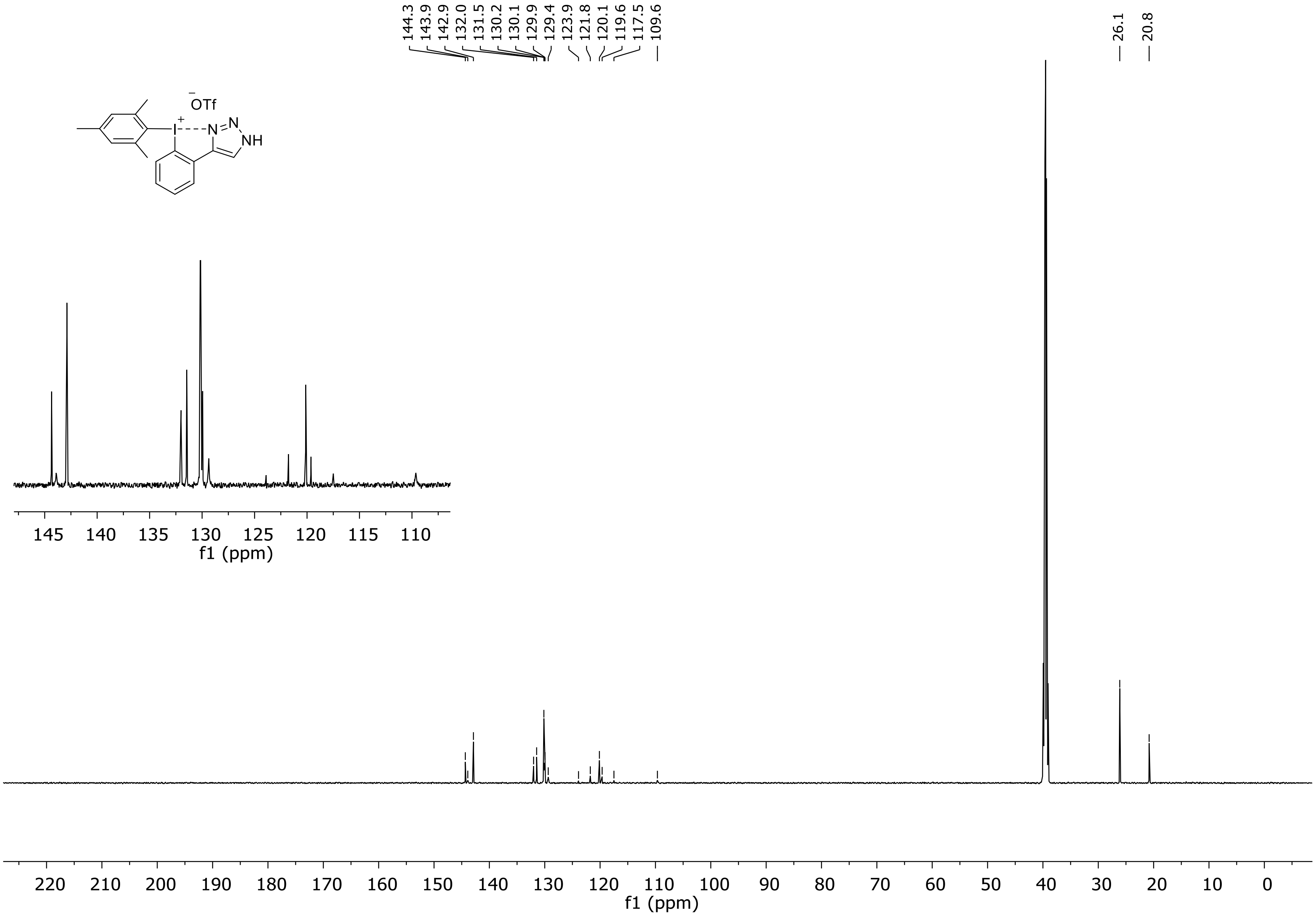

Figure S74: ^{13}C NMR spectra of **19** in d_6-DMSO.

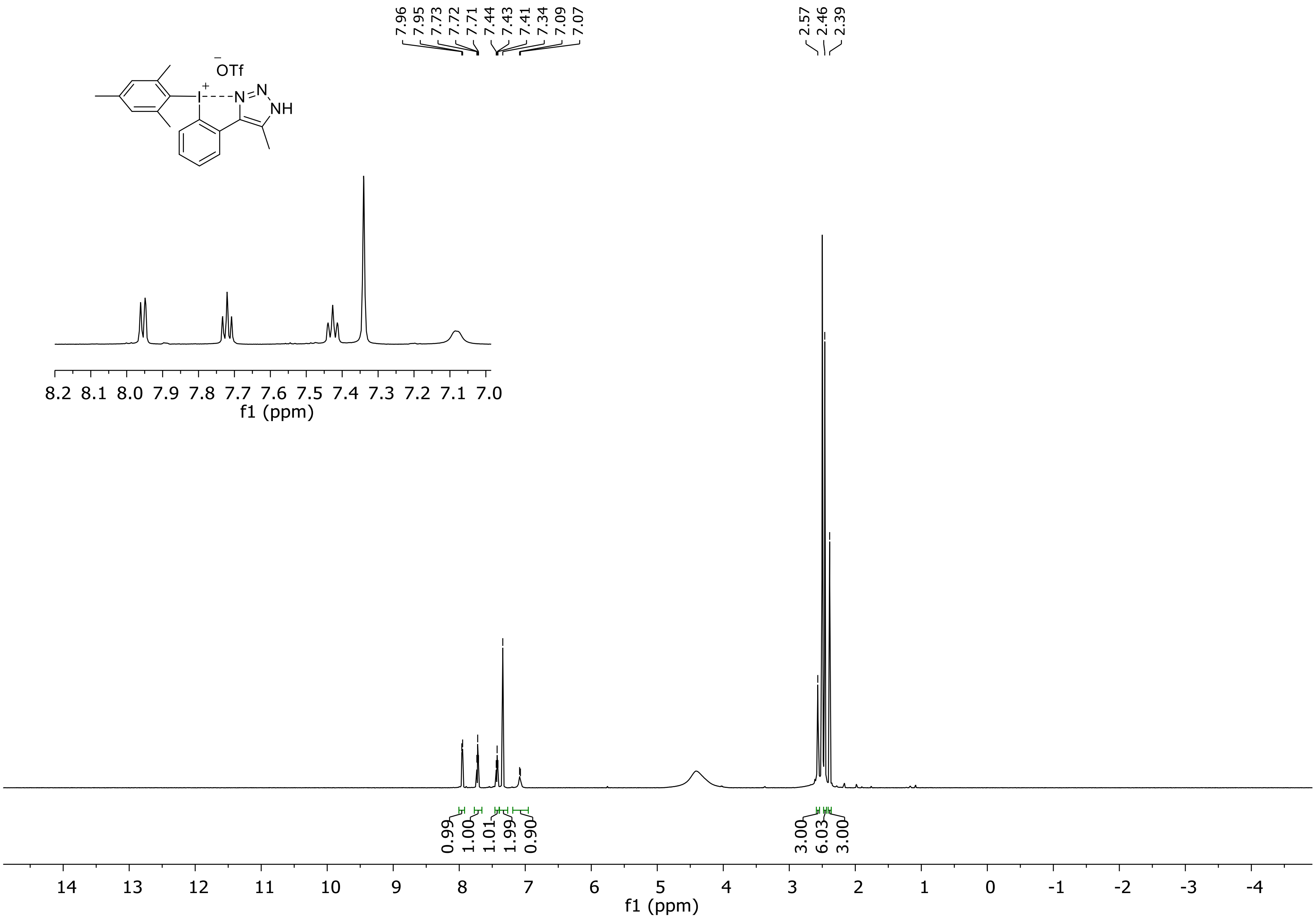

Figure S75: ^{1}H NMR spectra of **20** in d_6-DMSO.

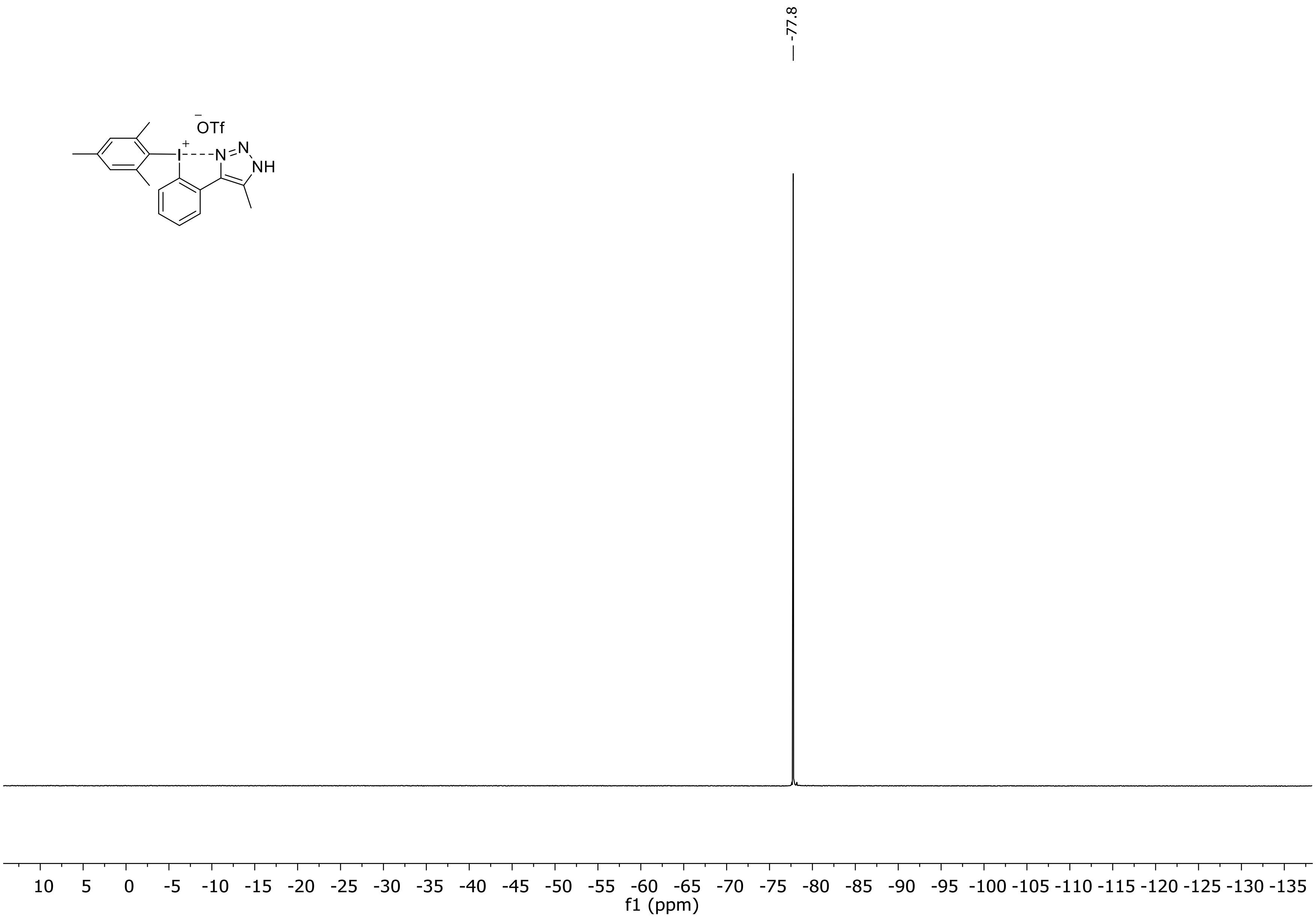

Figure S76: ^{19}F NMR spectra of **20** in d_6-DMSO.

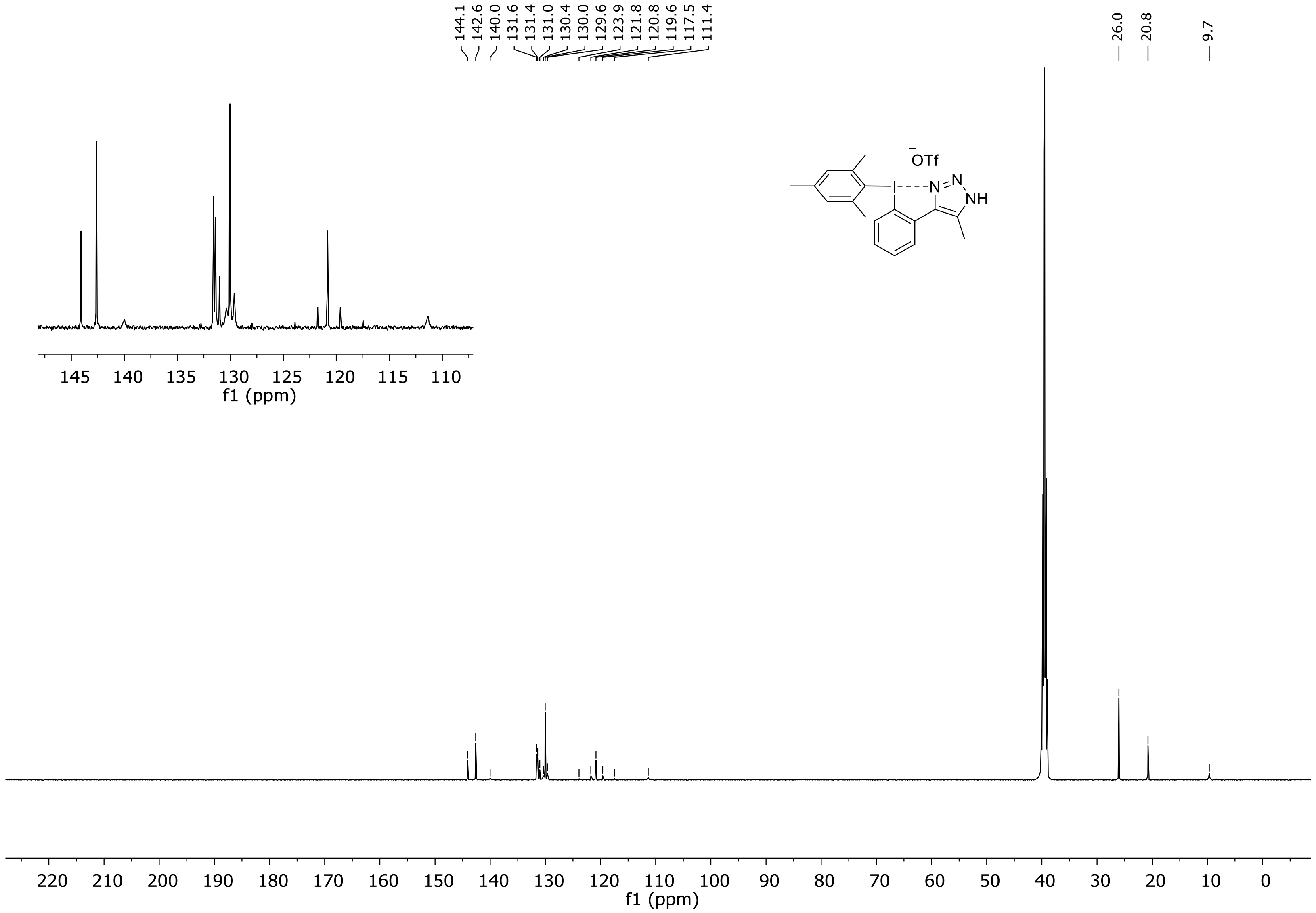

Figure S77: ^{13}C NMR spectra of **20** in d_6-DMSO.

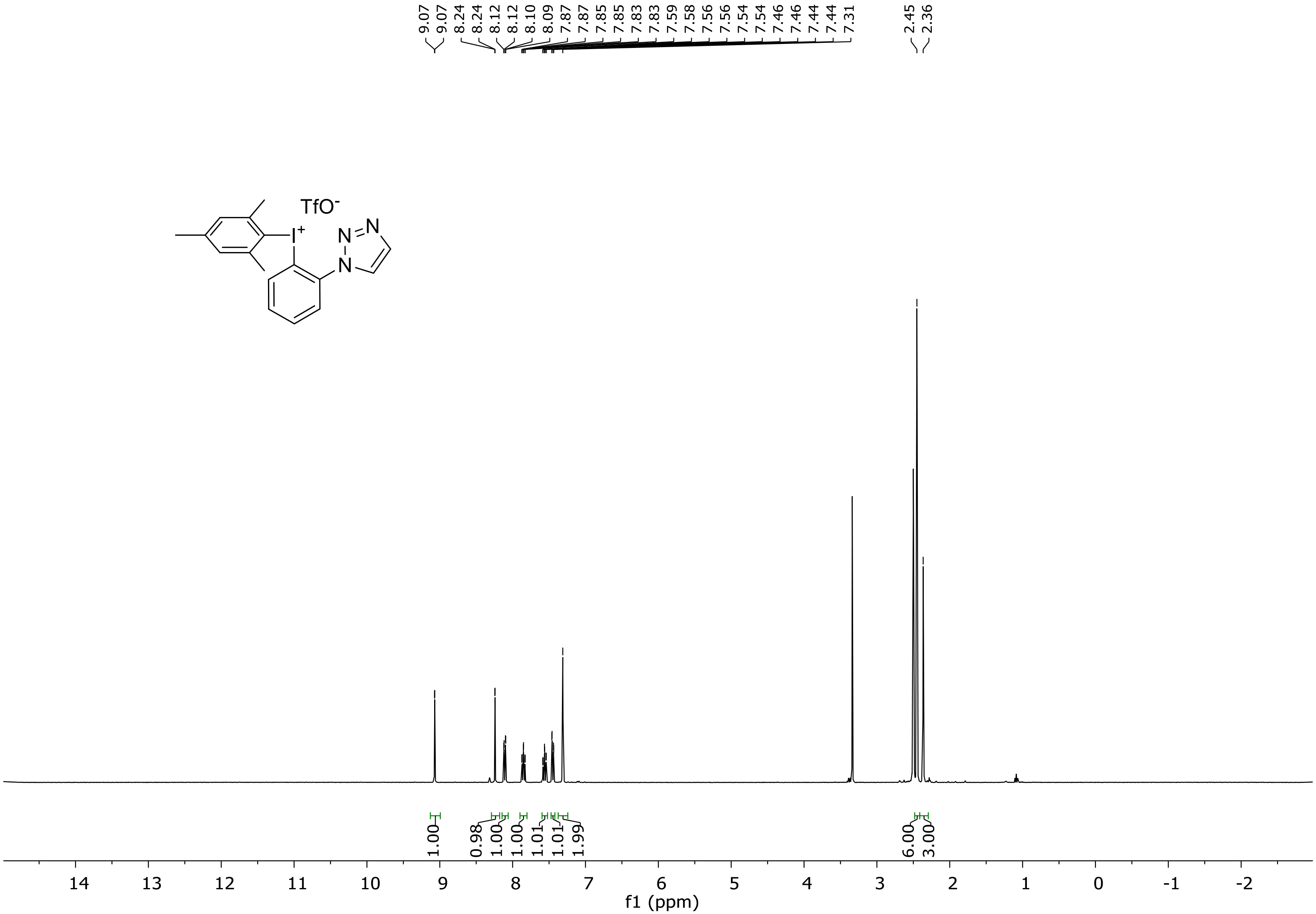

Figure S78: ^{1}H NMR spectra of **21** in d_6-DMSO.

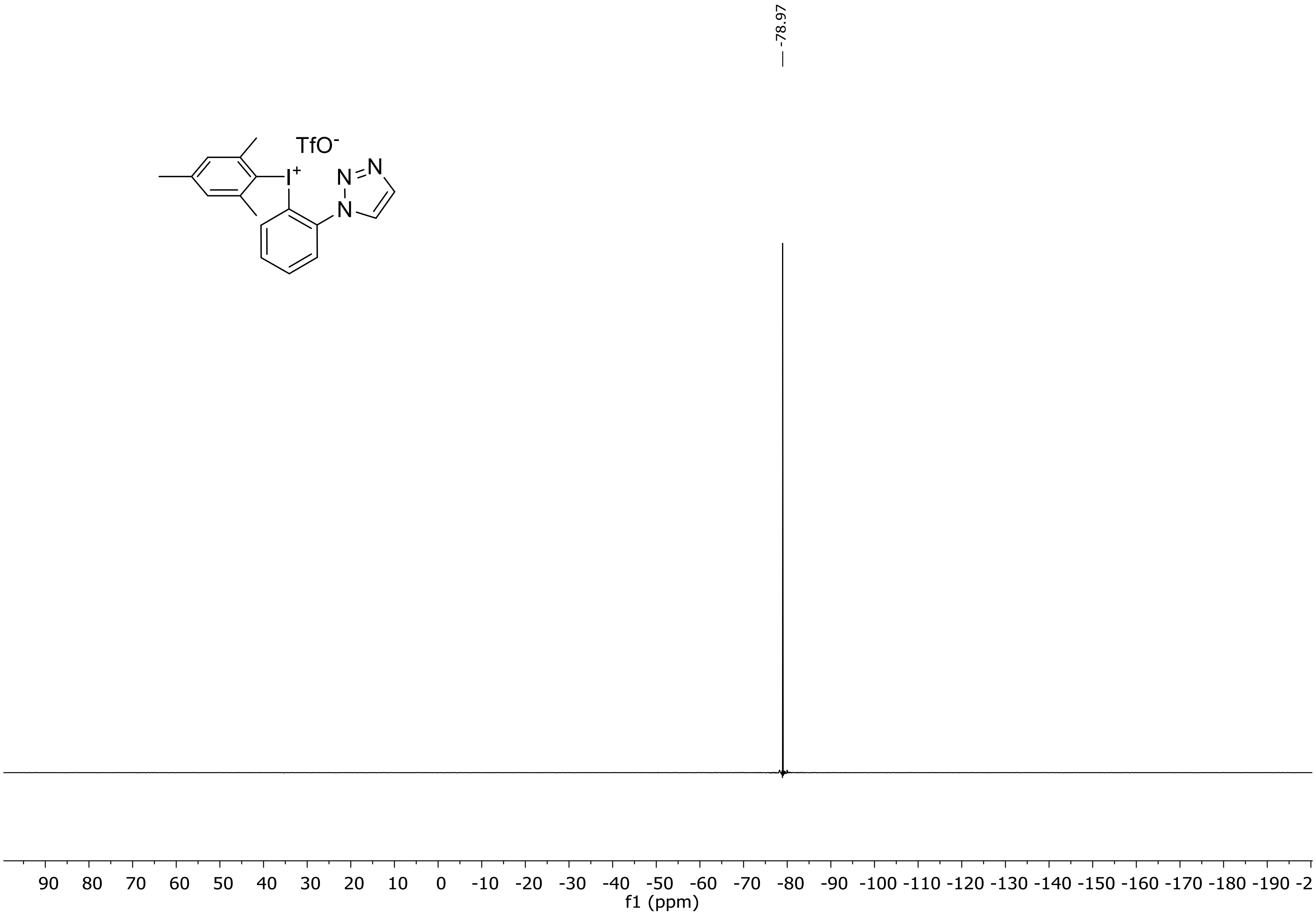

Figure S79: ^{19}F NMR spectra of **21** in d_6-DMSO.

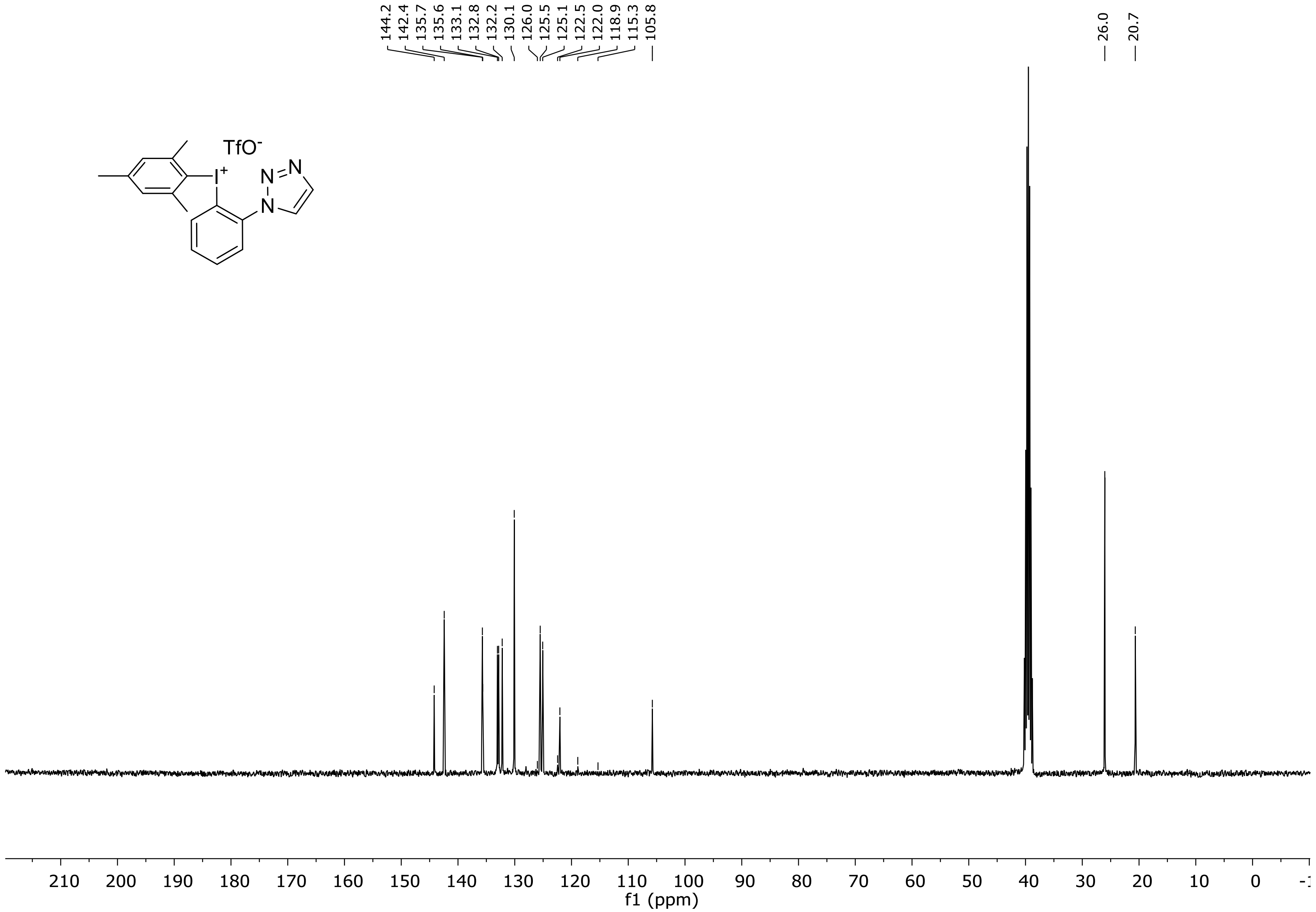

Figure S80: ^{13}C NMR spectra of **21** in d_6-DMSO.

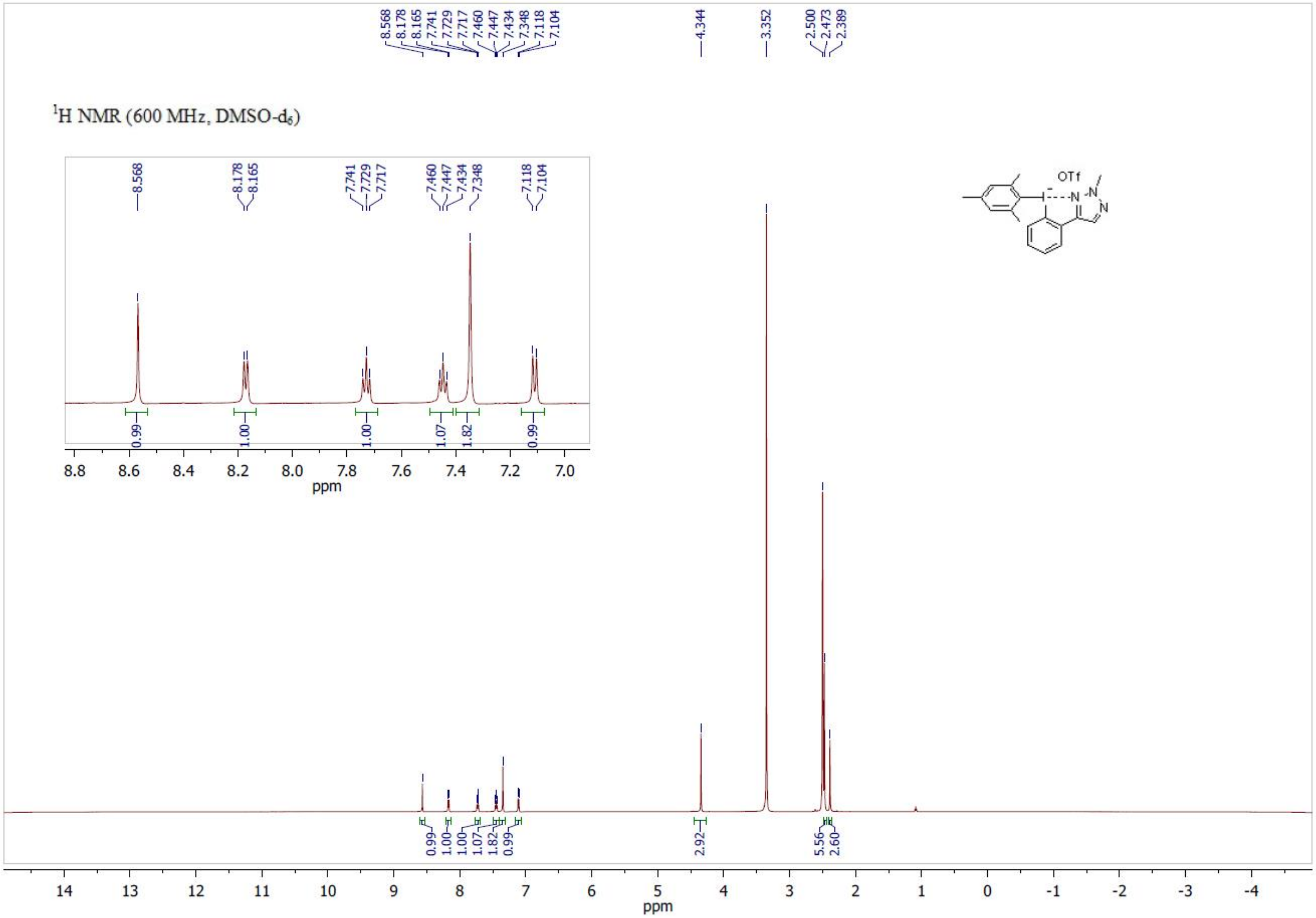

Figure S81: ^{1}H NMR spectra of **22** in d_6-DMSO.

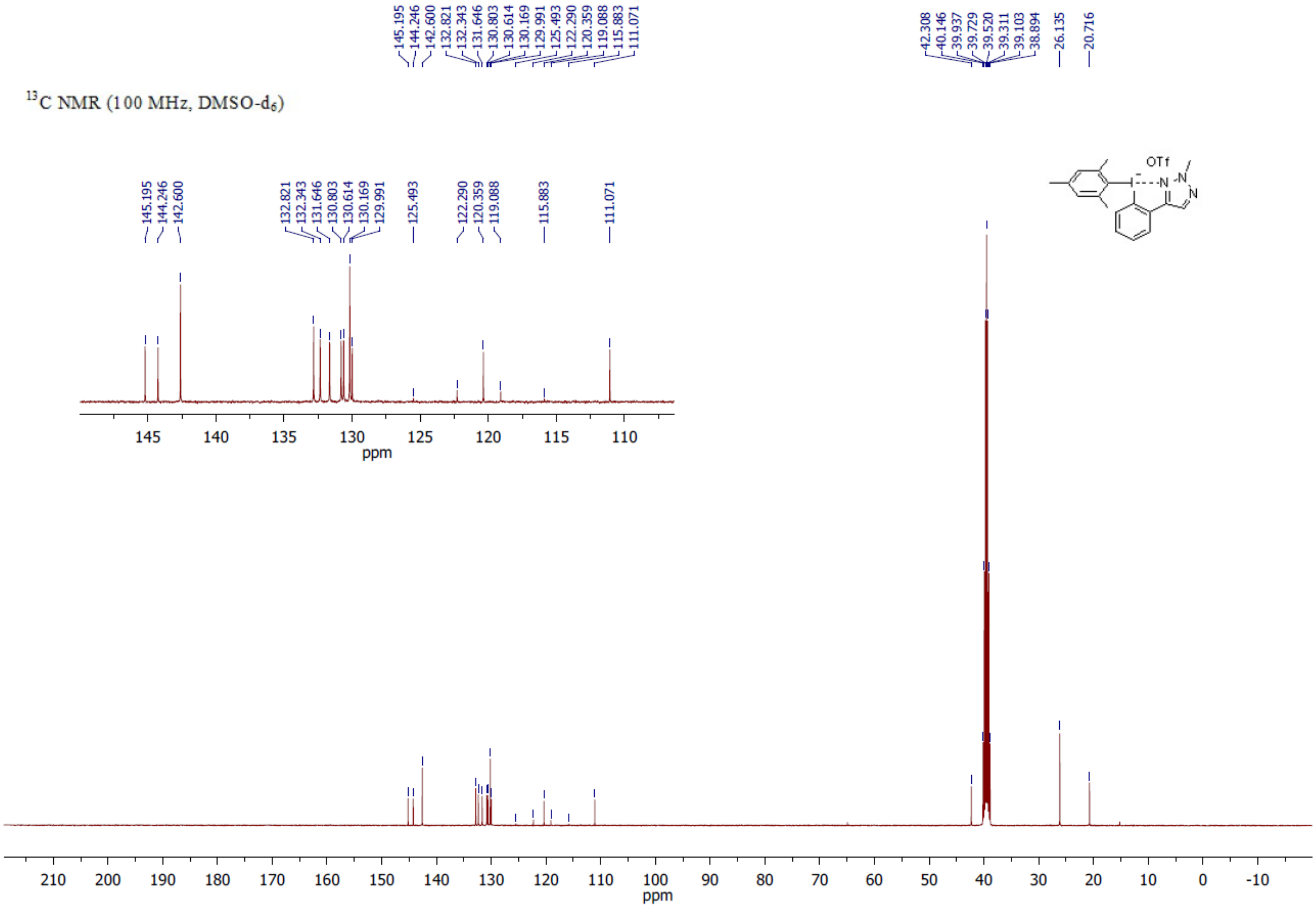

Figure S82: ^{13}C NMR spectra of **22** in d_6-DMSO.

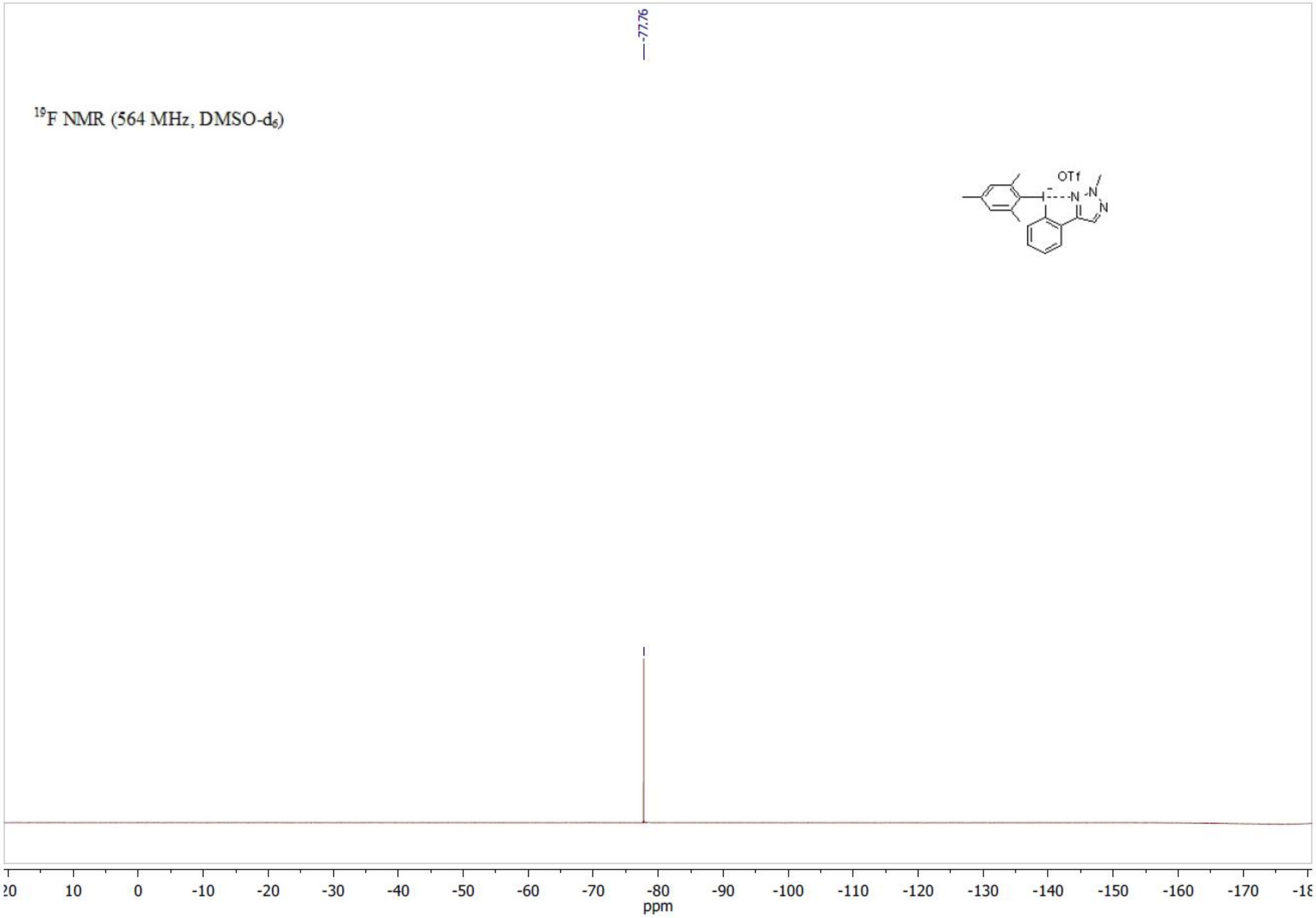

Figure S83: ^{19}F NMR spectra of **22** in d_6-DMSO.

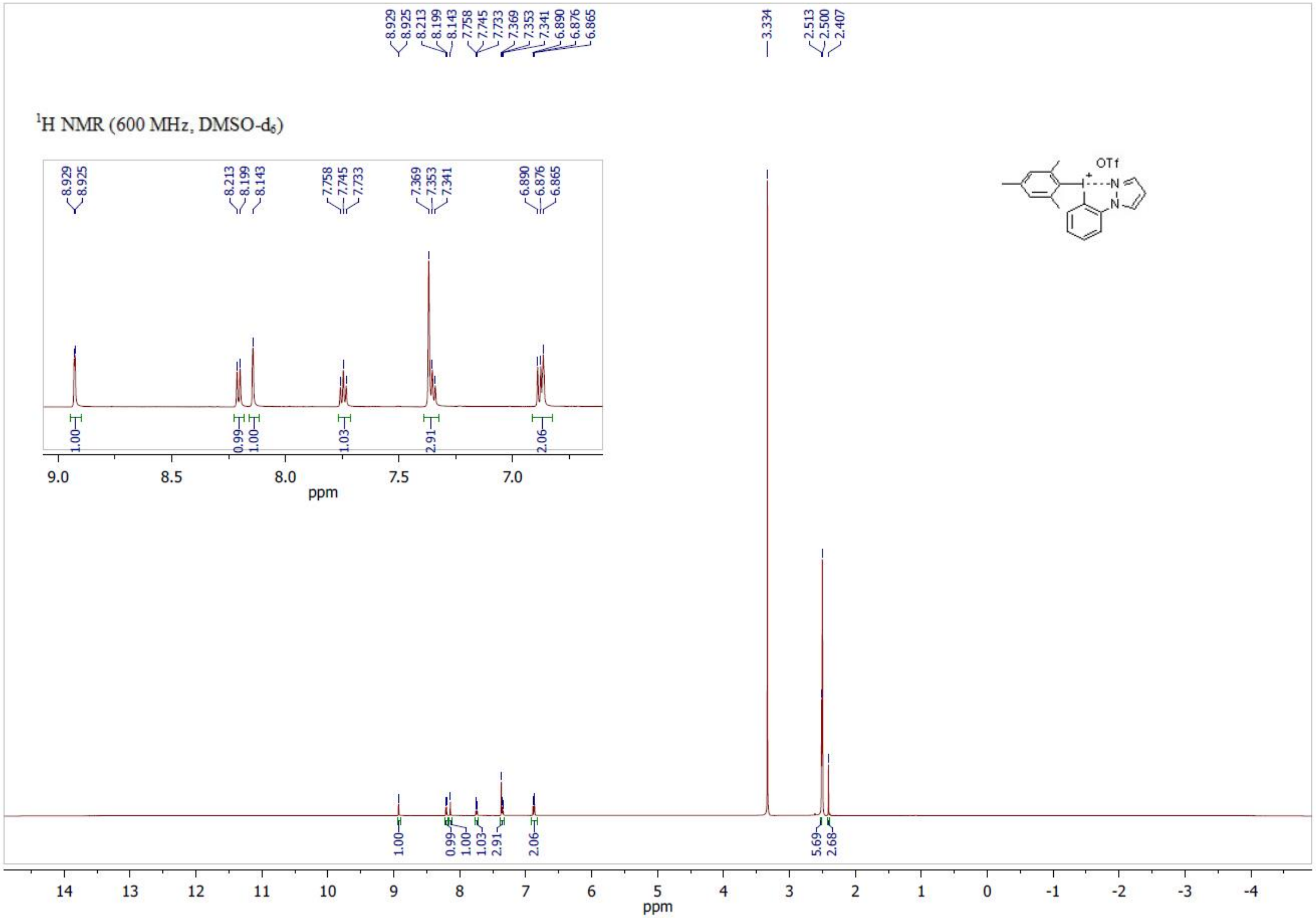

Figure S84: ^{1}H NMR spectra of **23** in d_6-DMSO.

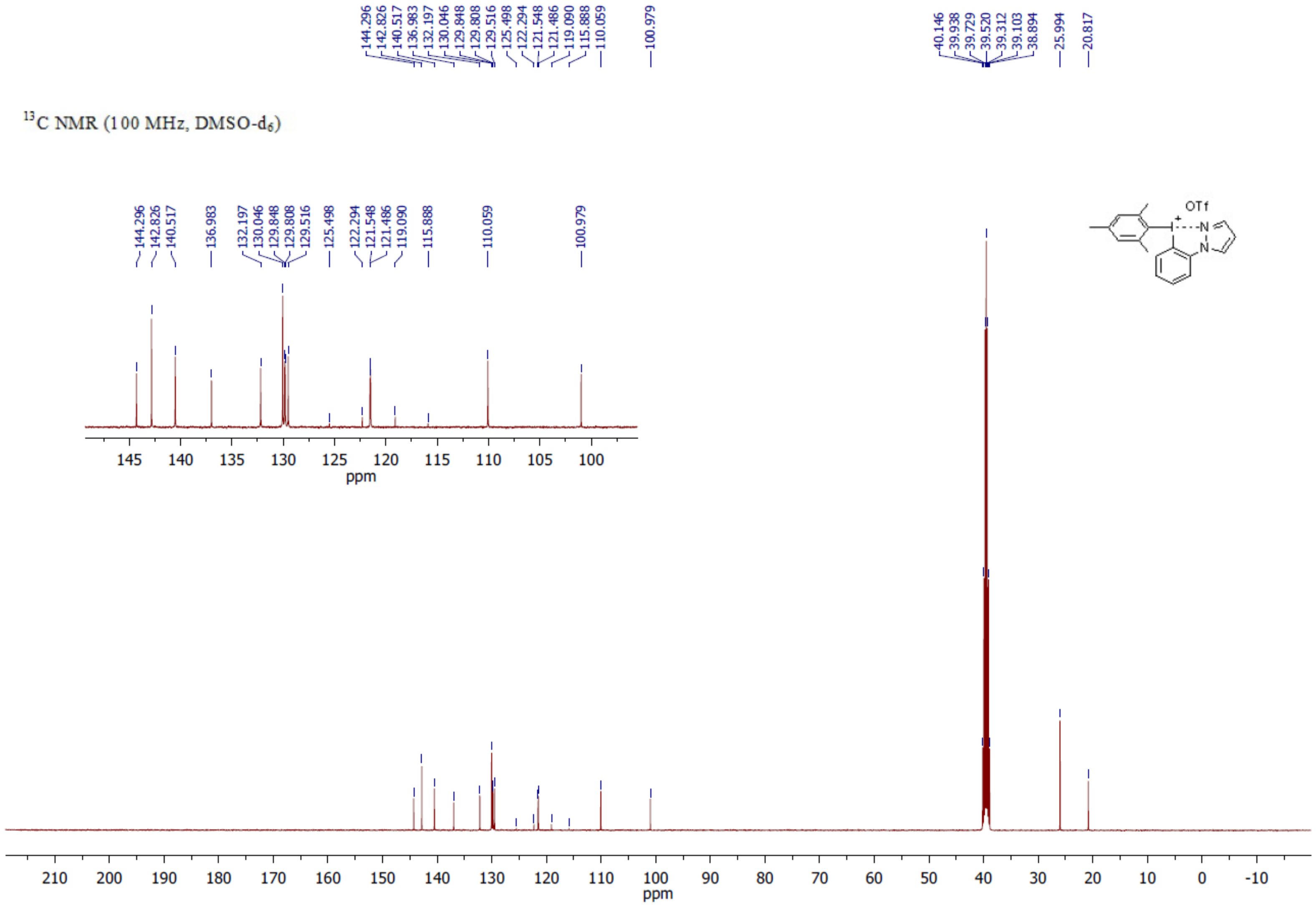

Figure S85: ^{13}C NMR spectra of **23** in d_6-DMSO.

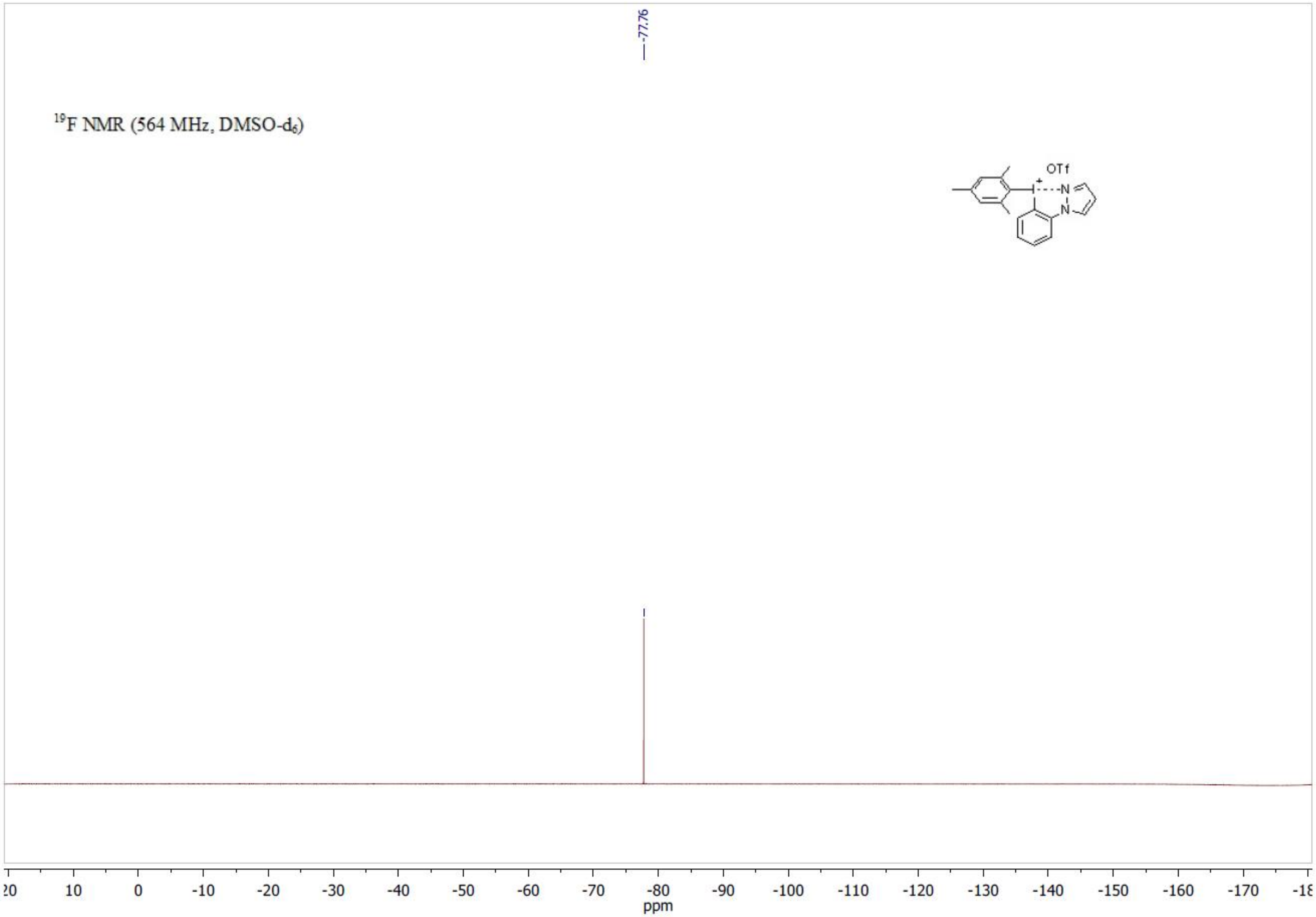

Figure S86: ^{19}F NMR spectra of **23** in d_6-DMSO.

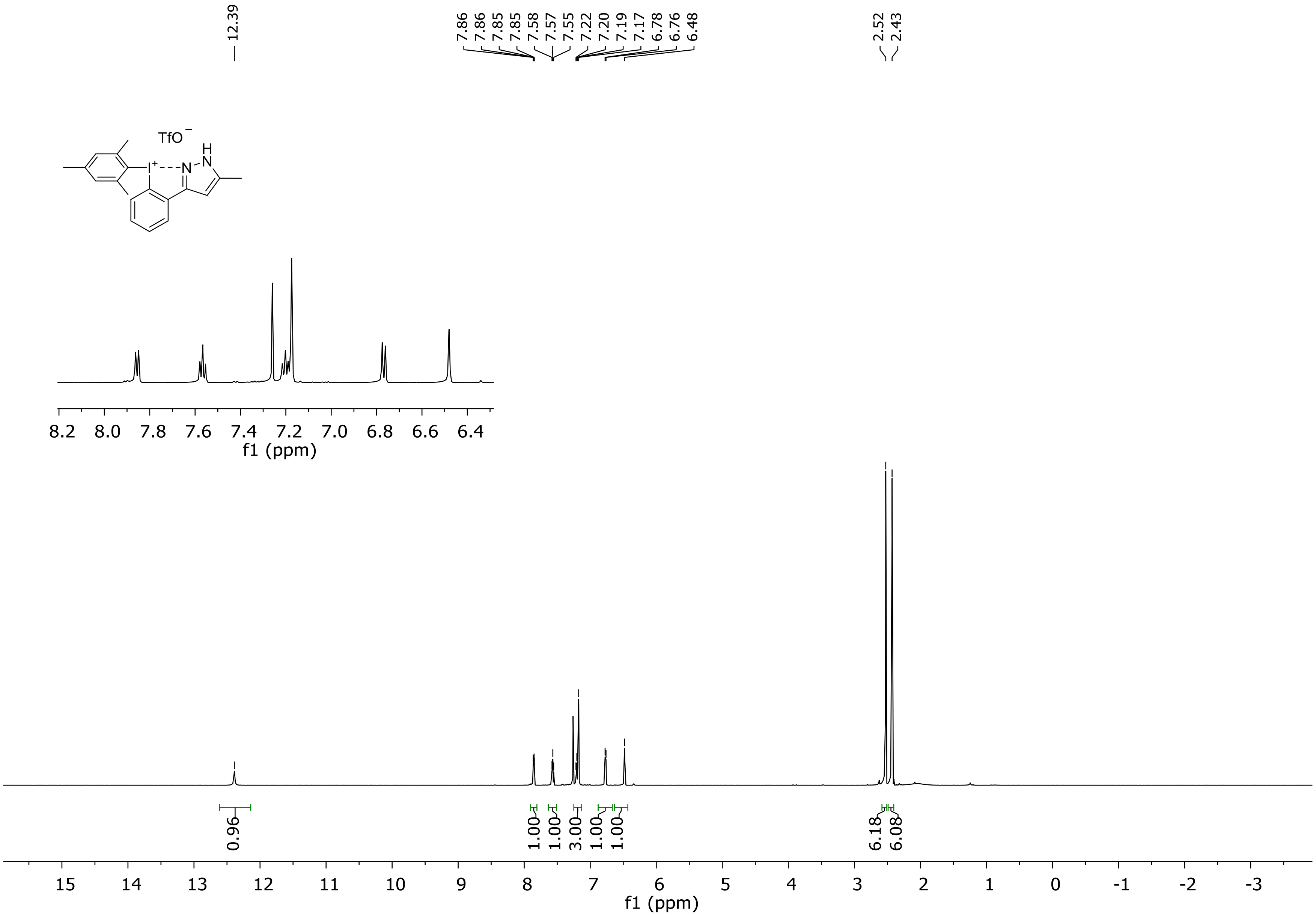

Figure S87: ^{1}H NMR spectra of **24** in $CDCl_3$.

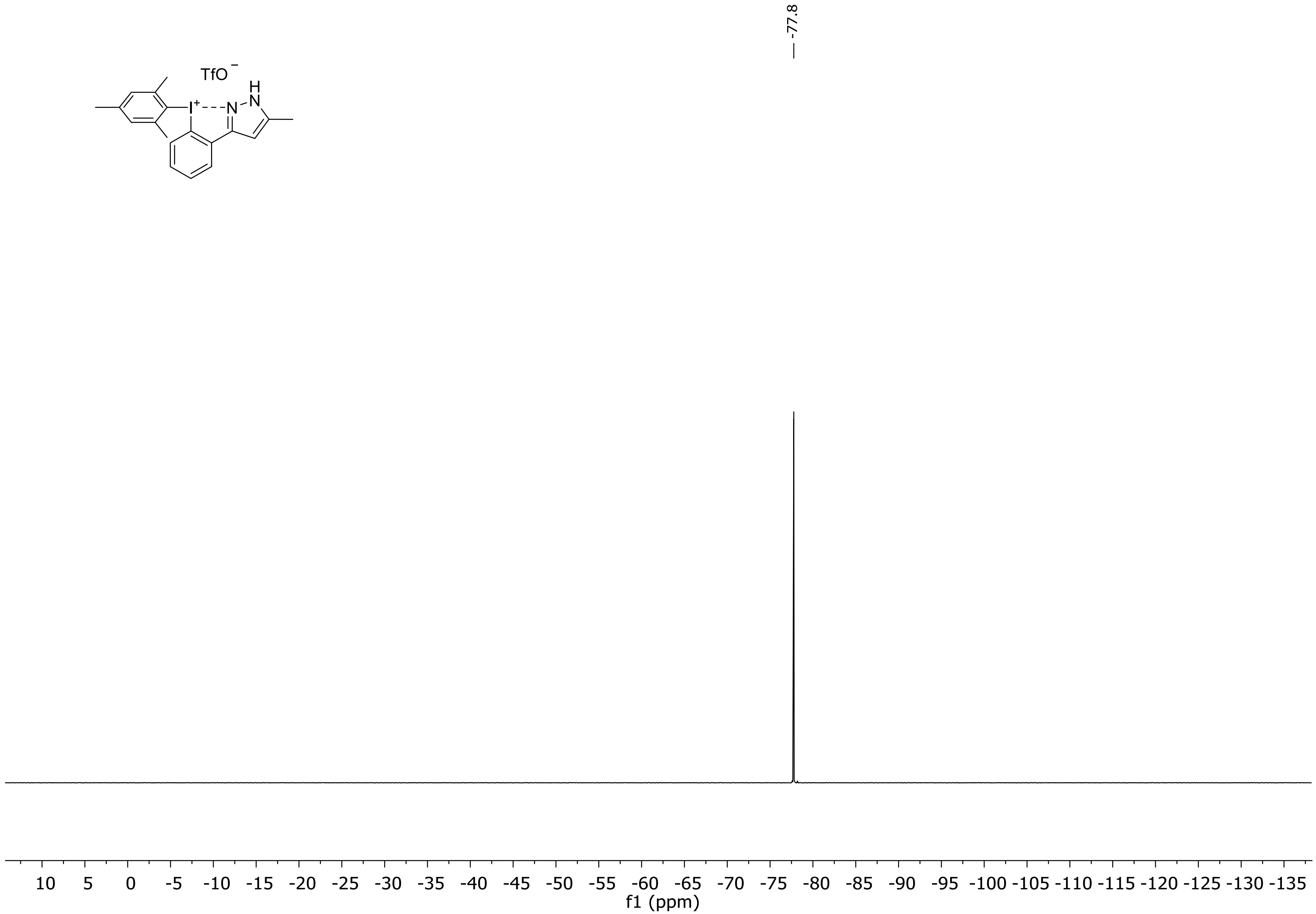

Figure S88: ^{19}F NMR spectra of **24** in $CDCl_3$.

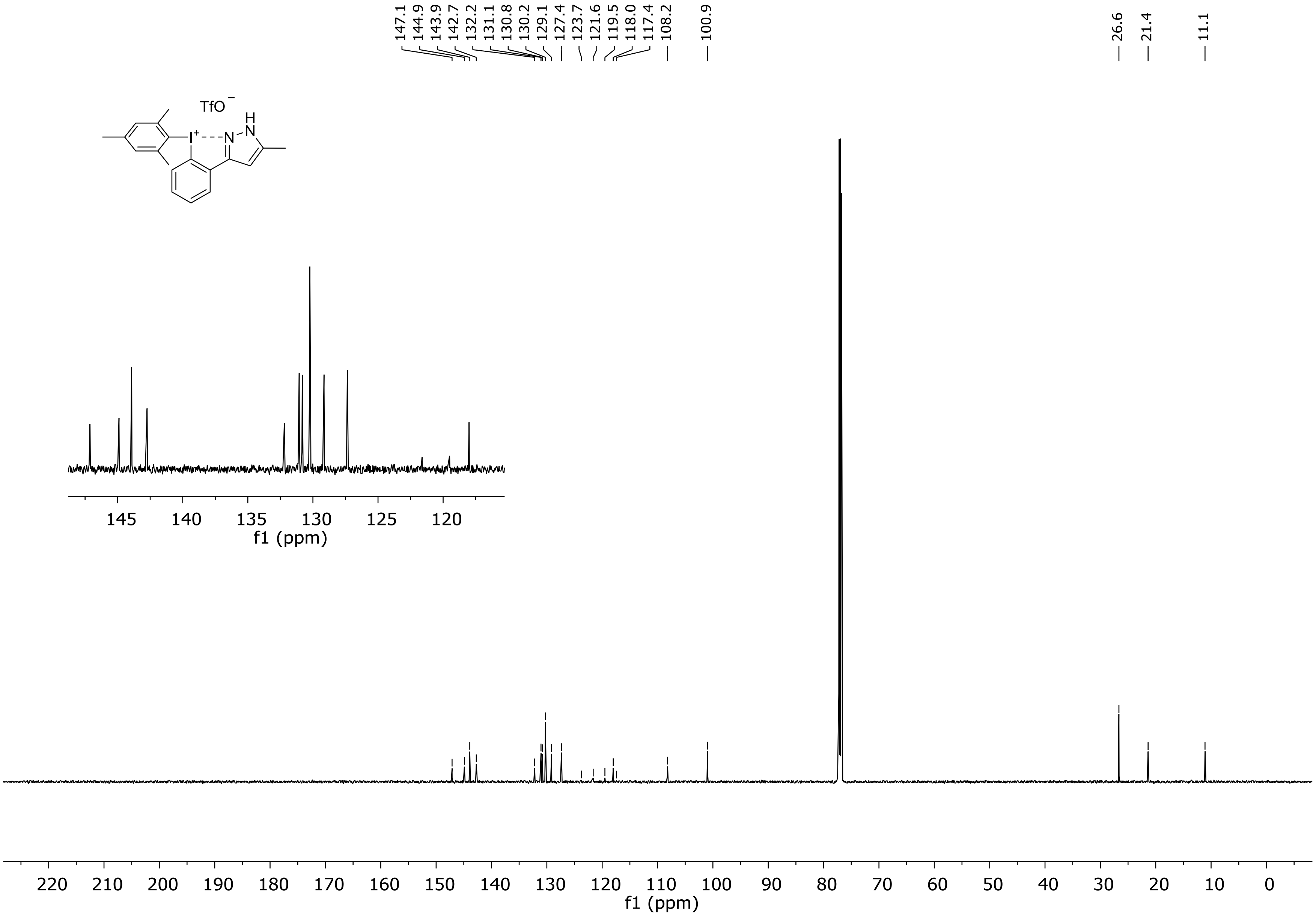

Figure S89: ^{13}C NMR spectra of **24** in $CDCl_3$.

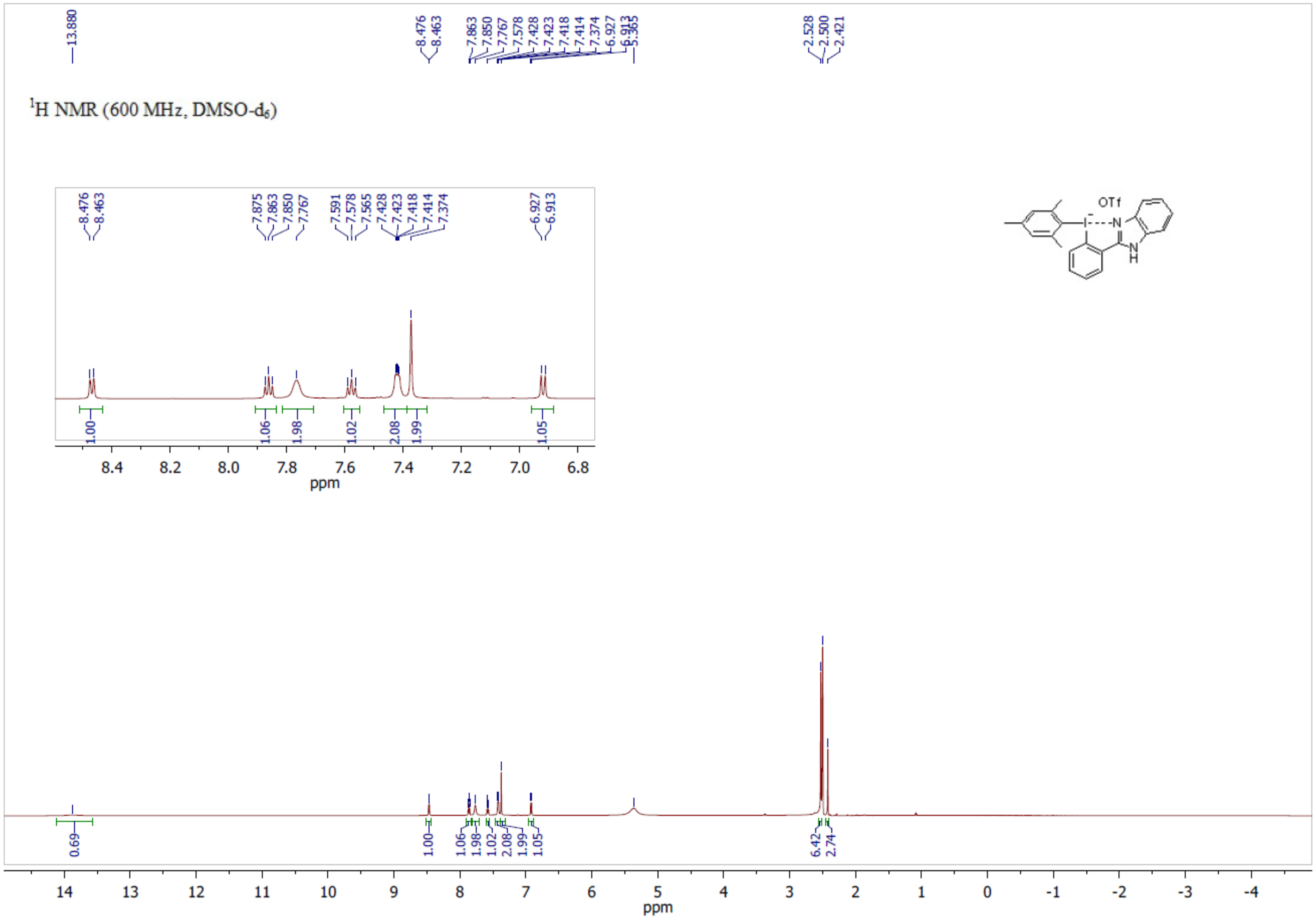

Figure S90: ^{1}H NMR spectra of **25** in d_6-DMSO.

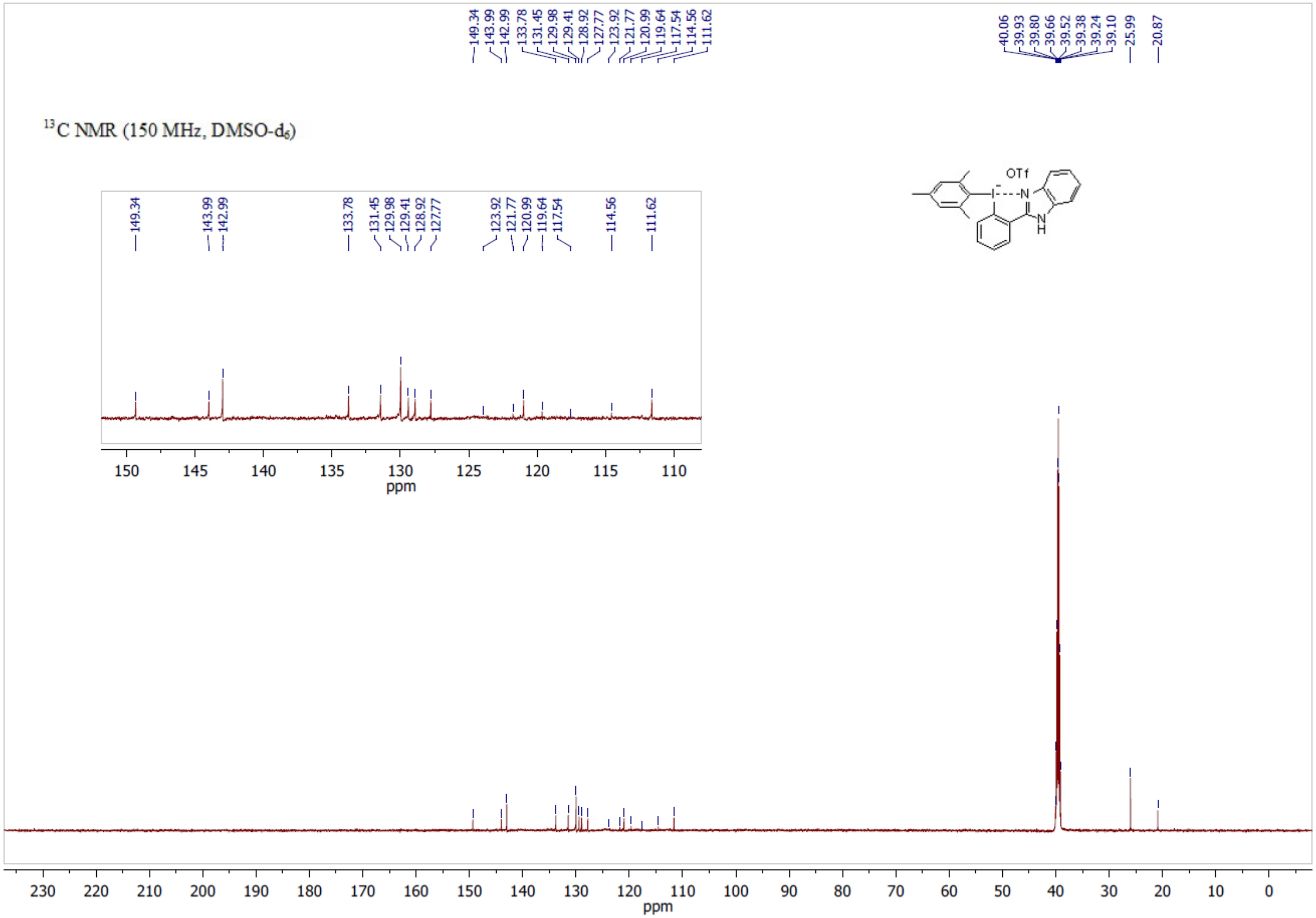

Figure S91: ^{13}C NMR spectra of **25** in d_6-DMSO.

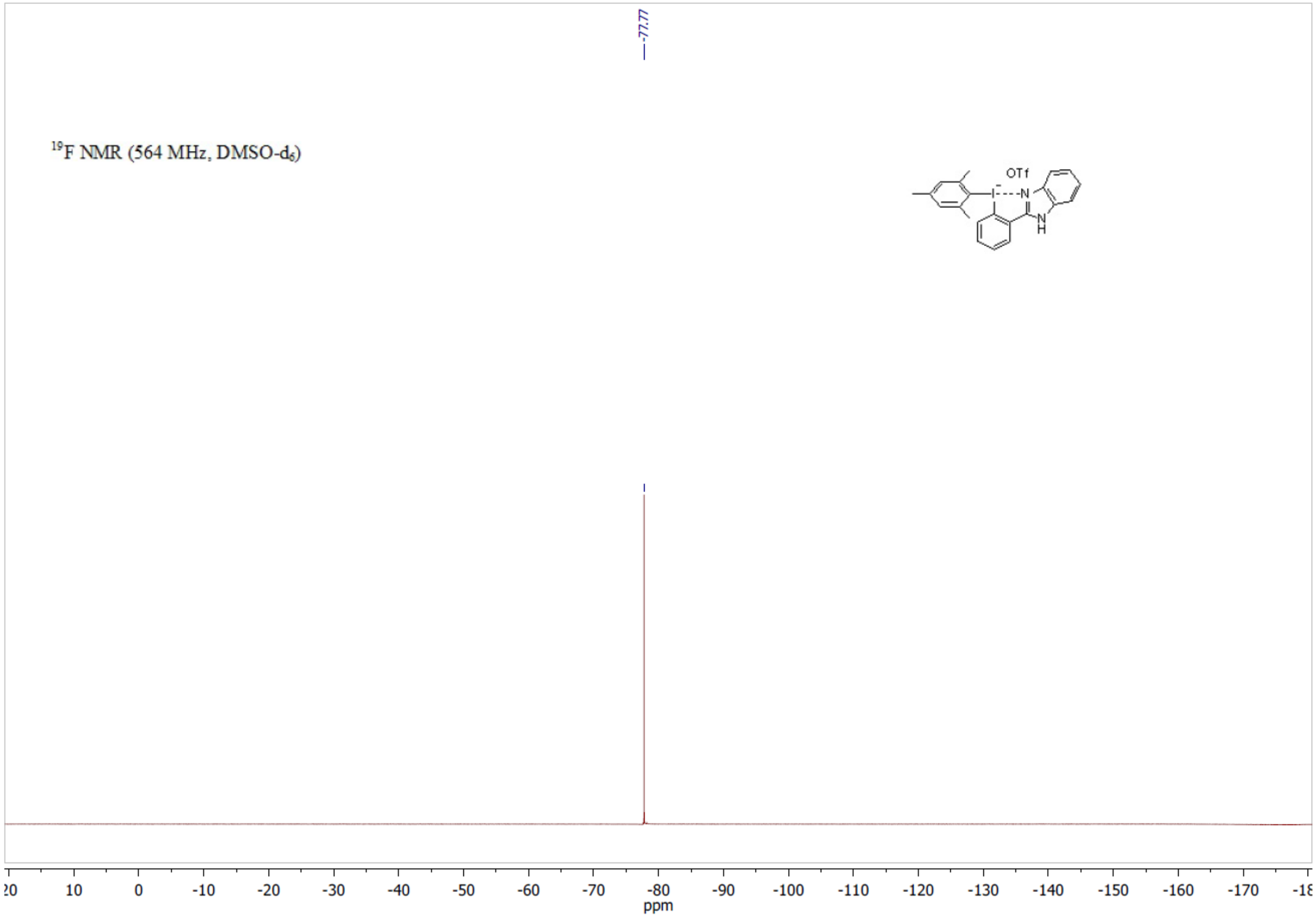

Figure S92: ^{19}F NMR spectra of **25** in d_6-DMSO.

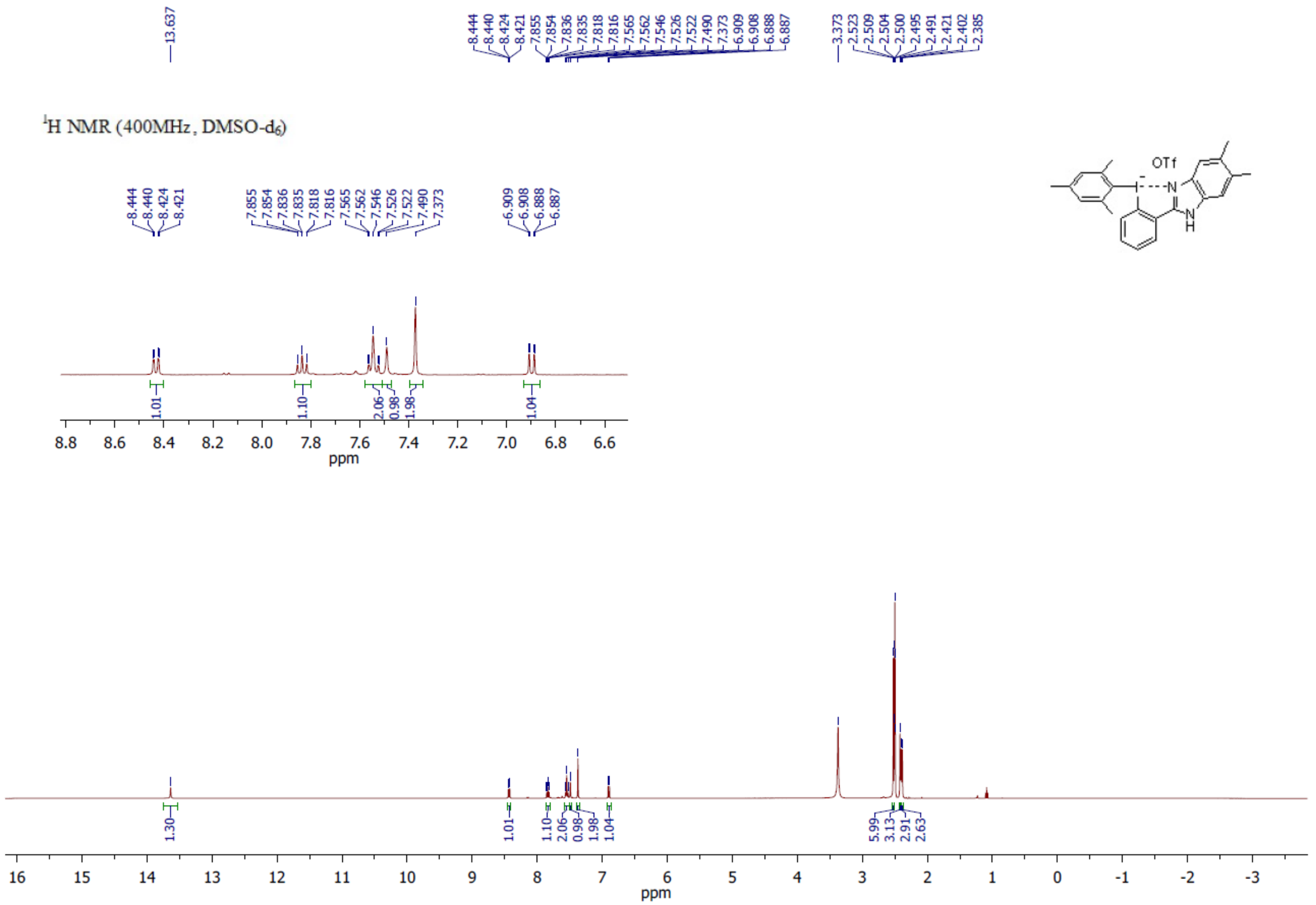

Figure S93: ^{1}H NMR spectra of **26** in d_6-DMSO.

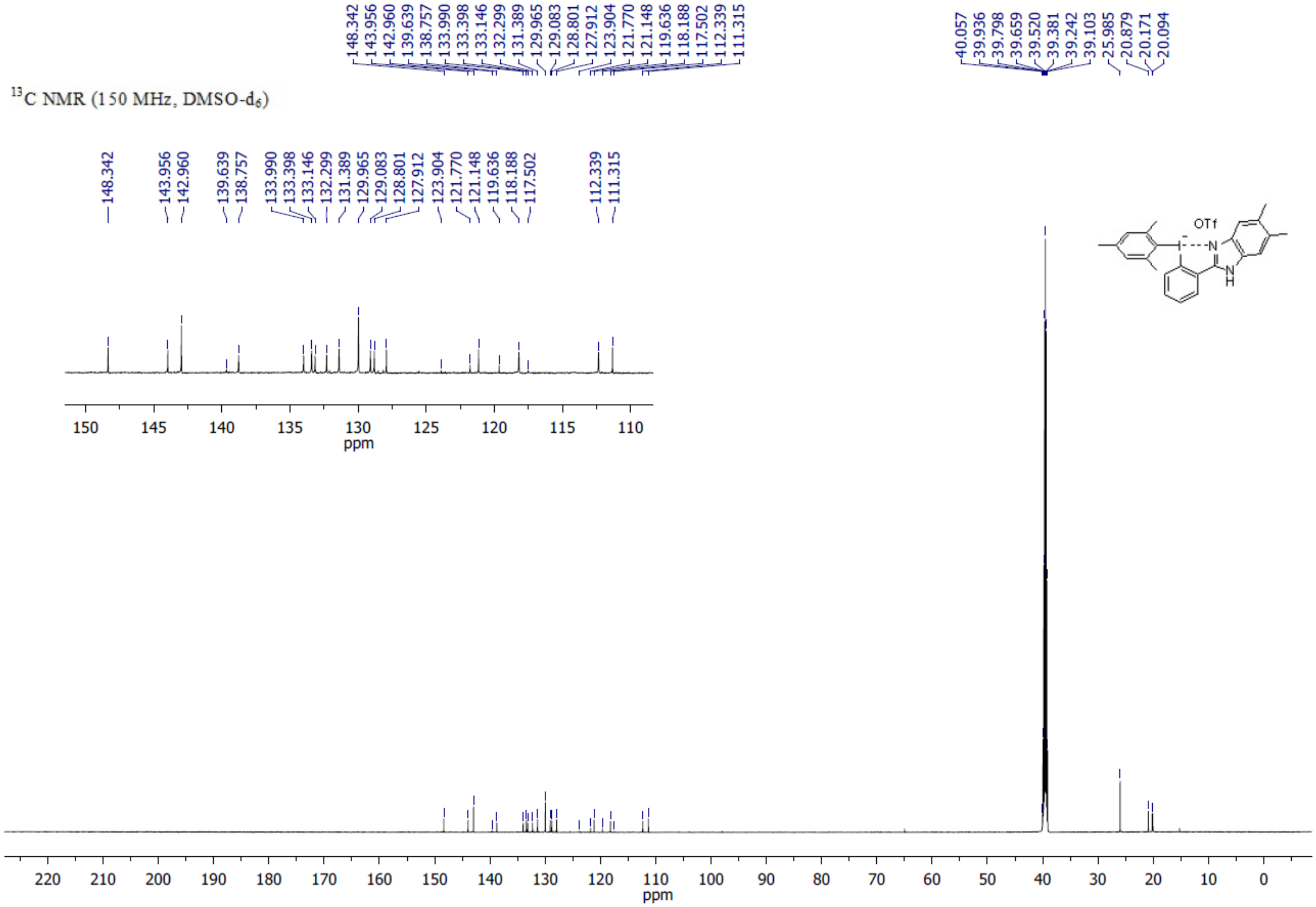

Figure S94: ^{13}C NMR spectra of **26** in d_6-DMSO.

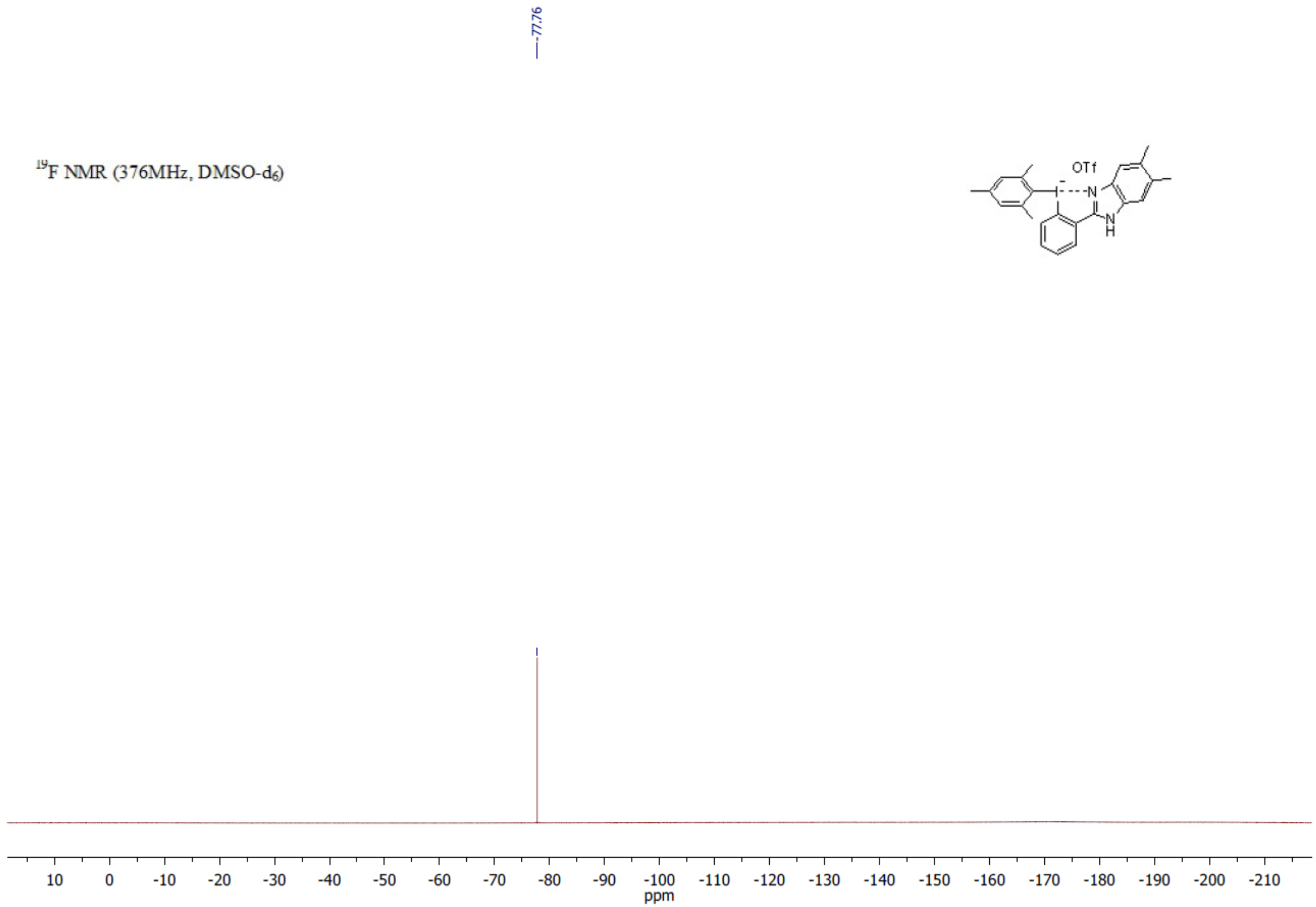

Figure S95: ^{19}F NMR spectra of **26** in d_6-DMSO.

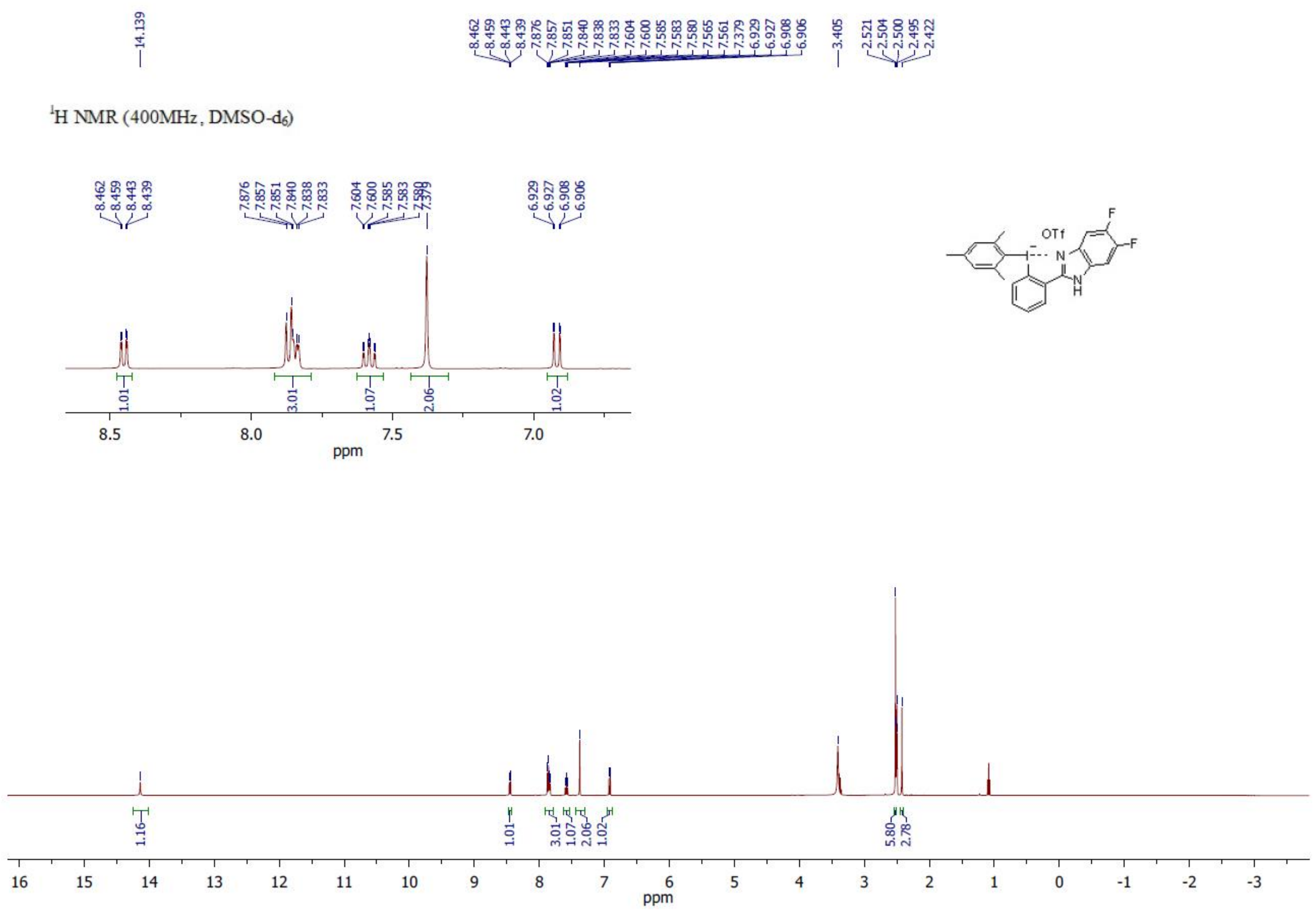

Figure S96: ^{1}H NMR spectra of **27** in d_6-DMSO.

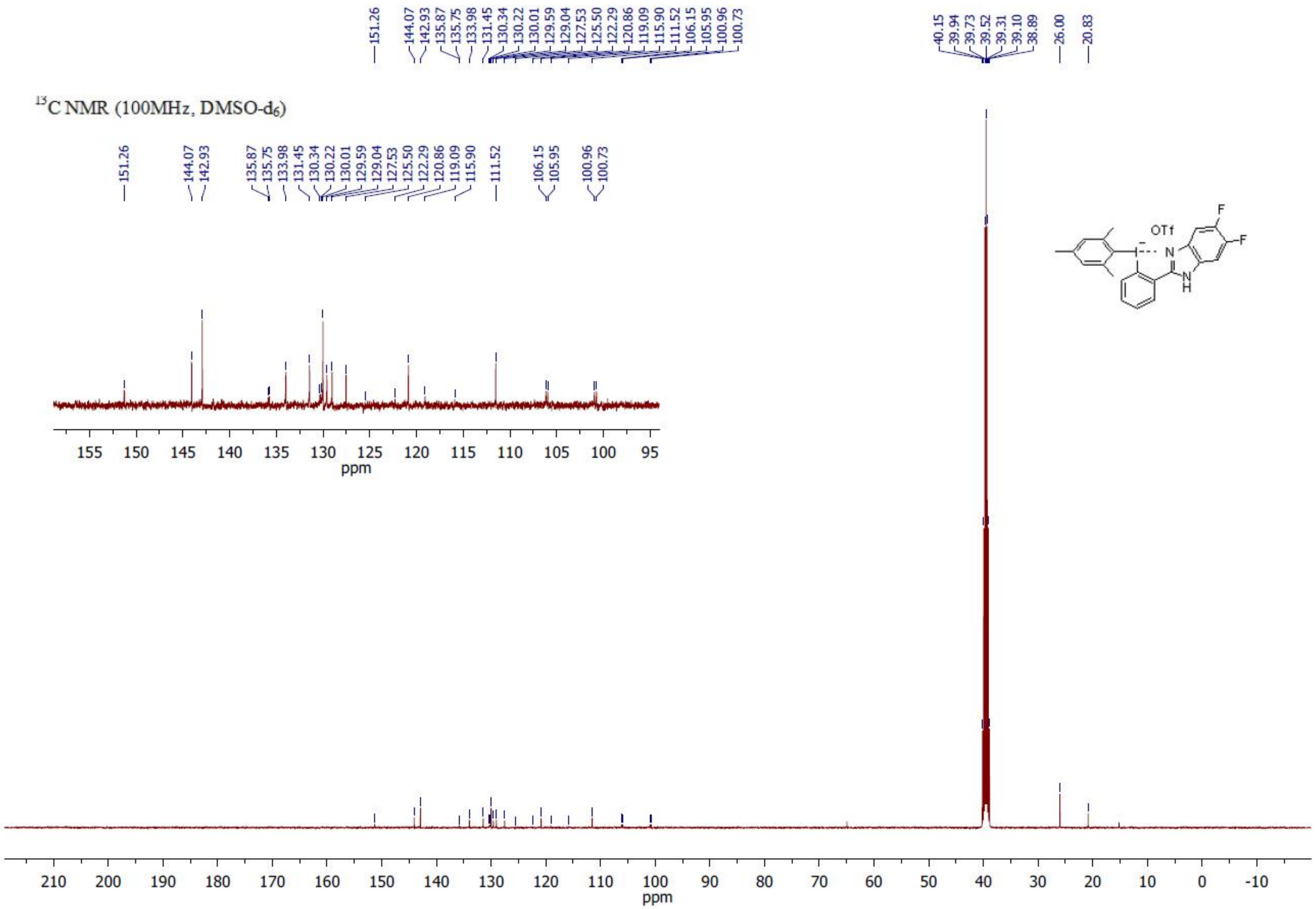

Figure S97: ^{13}C NMR spectra of **27** in d_6-DMSO.

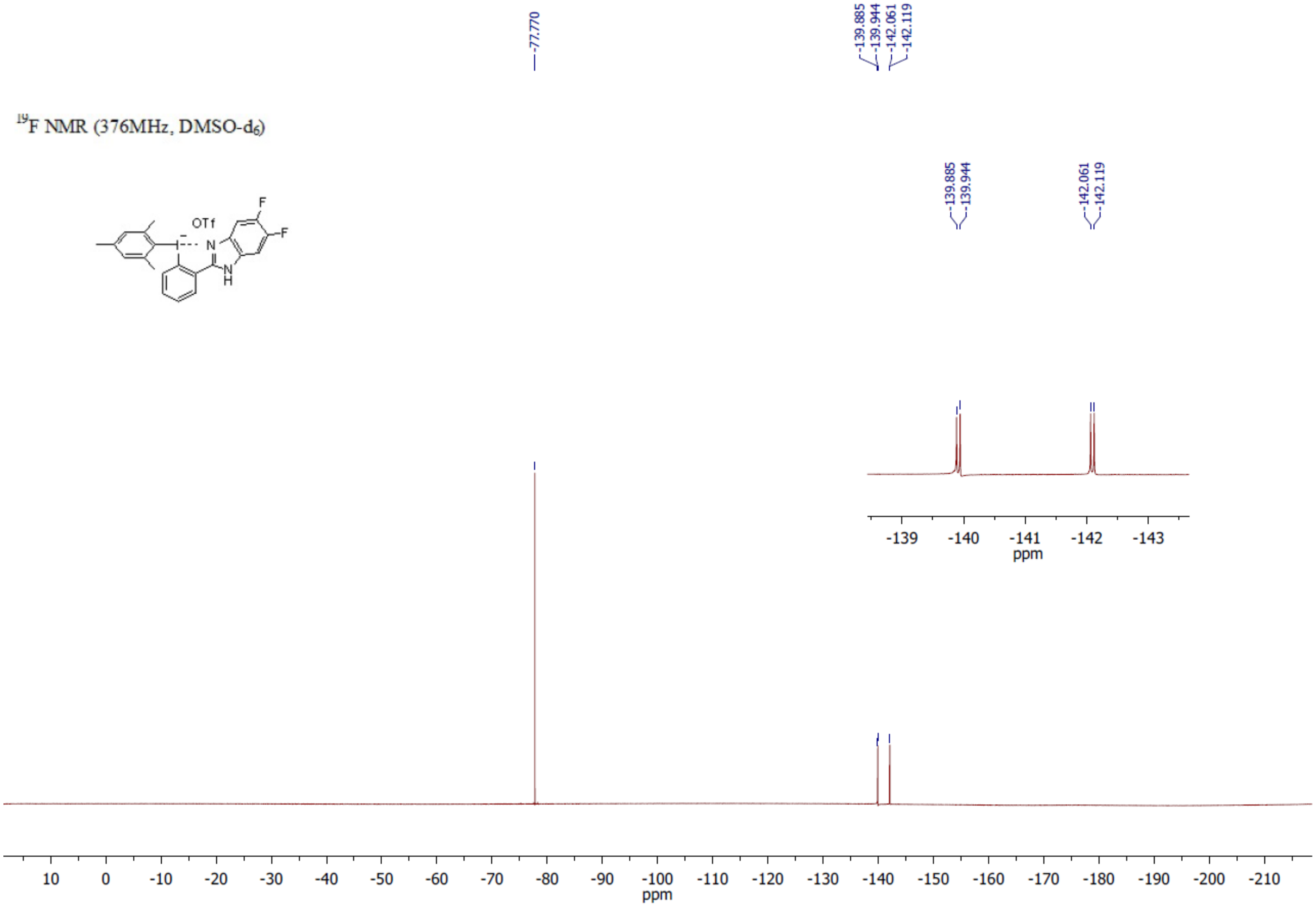

Figure S98: ^{19}F NMR spectra of **27** in d_6-DMSO.

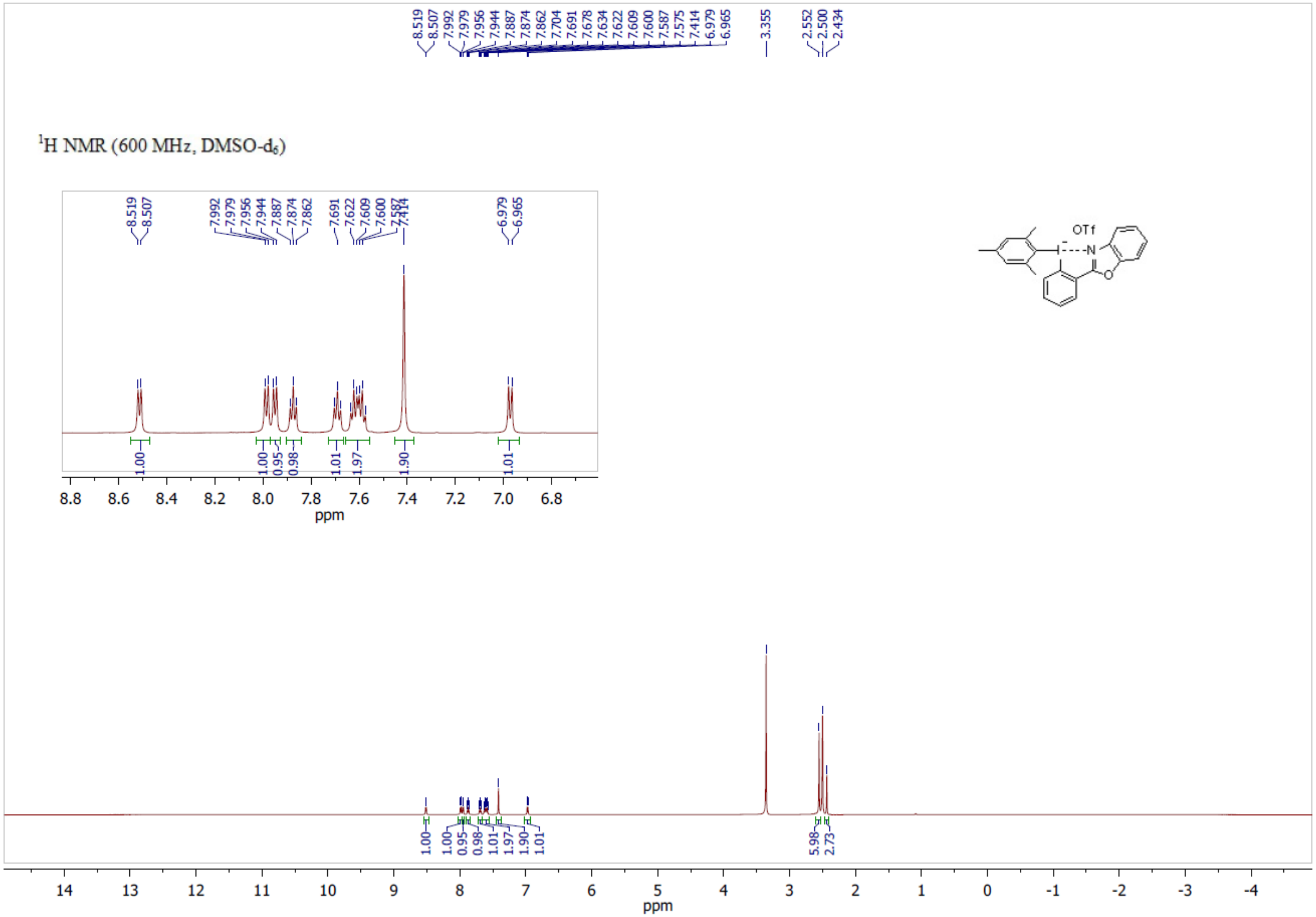

Figure S99: ^{1}H NMR spectra of **28** in d_6-DMSO.

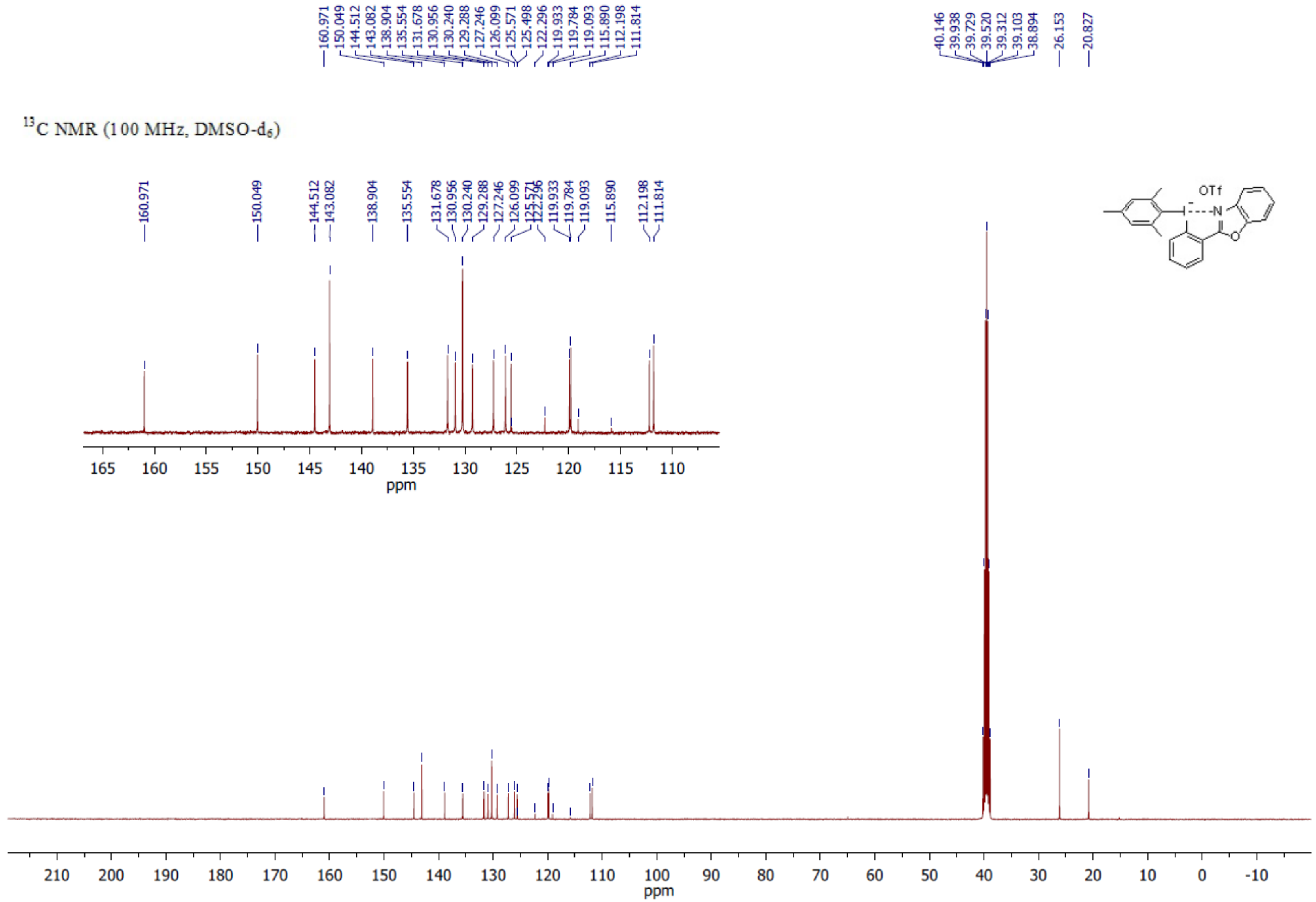

Figure S100: ^{13}C NMR spectra of **28** in d_6-DMSO.

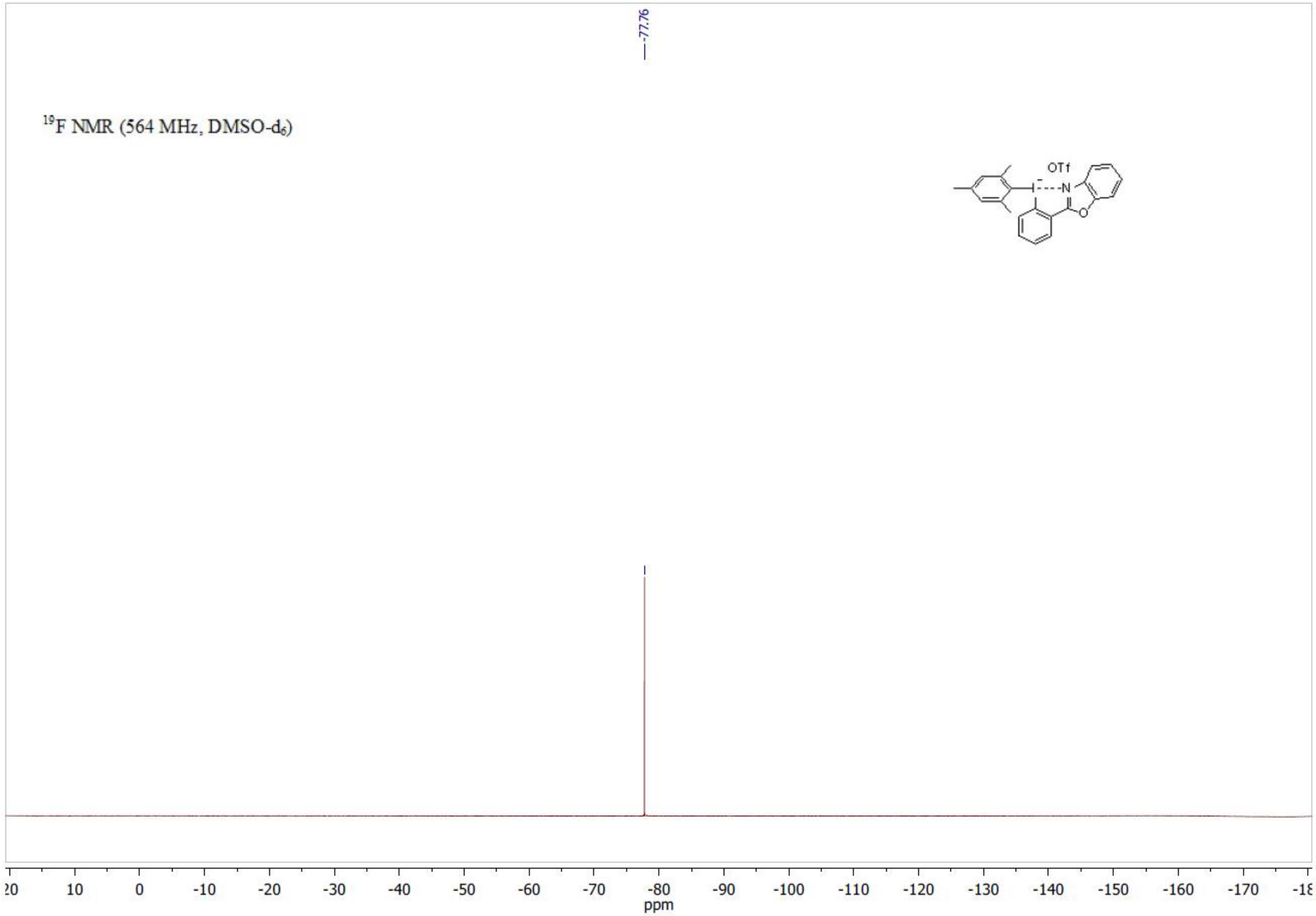

Figure S101: ^{19}F NMR spectra of **28** in d_6-DMSO.

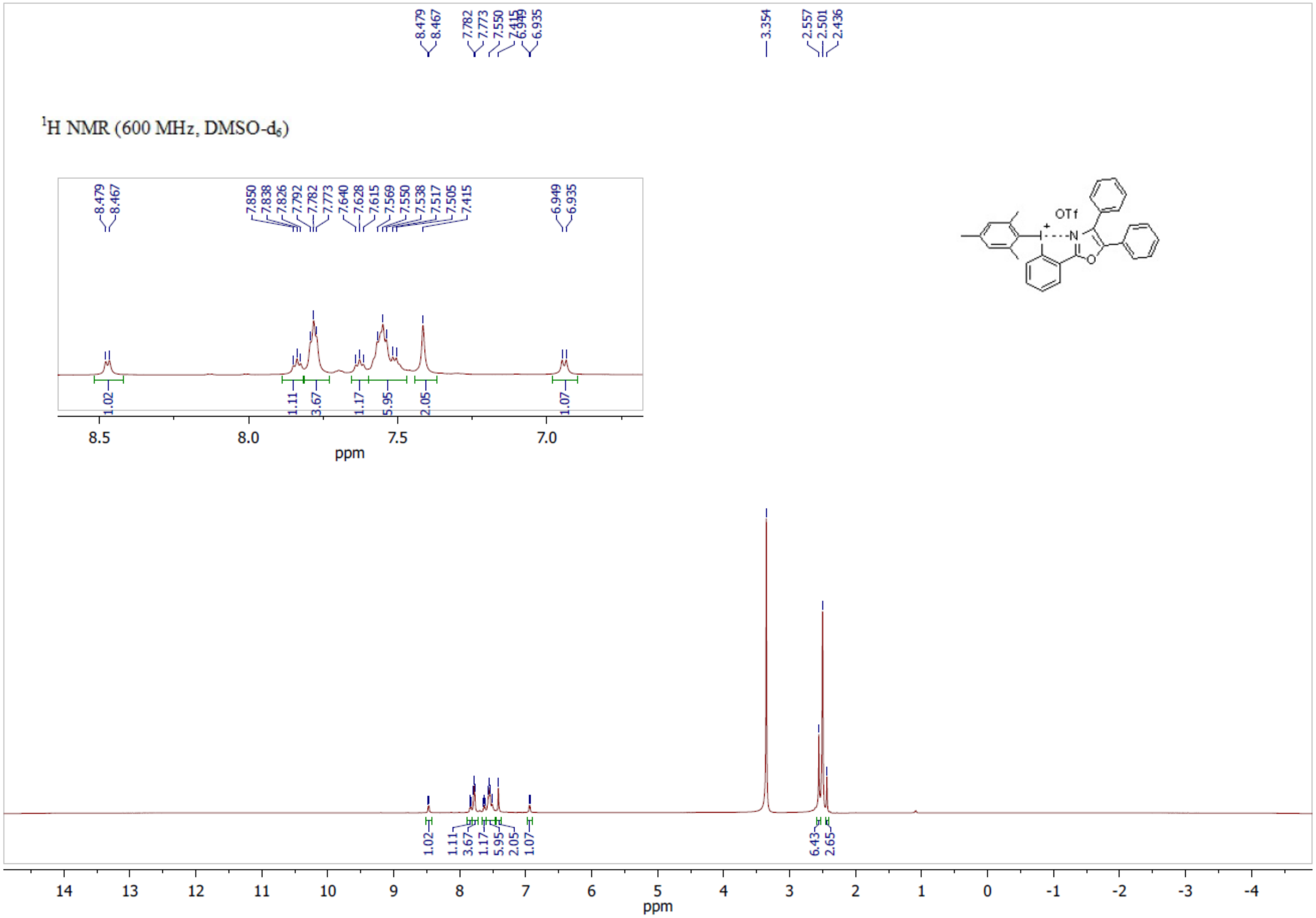

Figure S102: ^{1}H NMR spectra of **29** in d_6-DMSO.

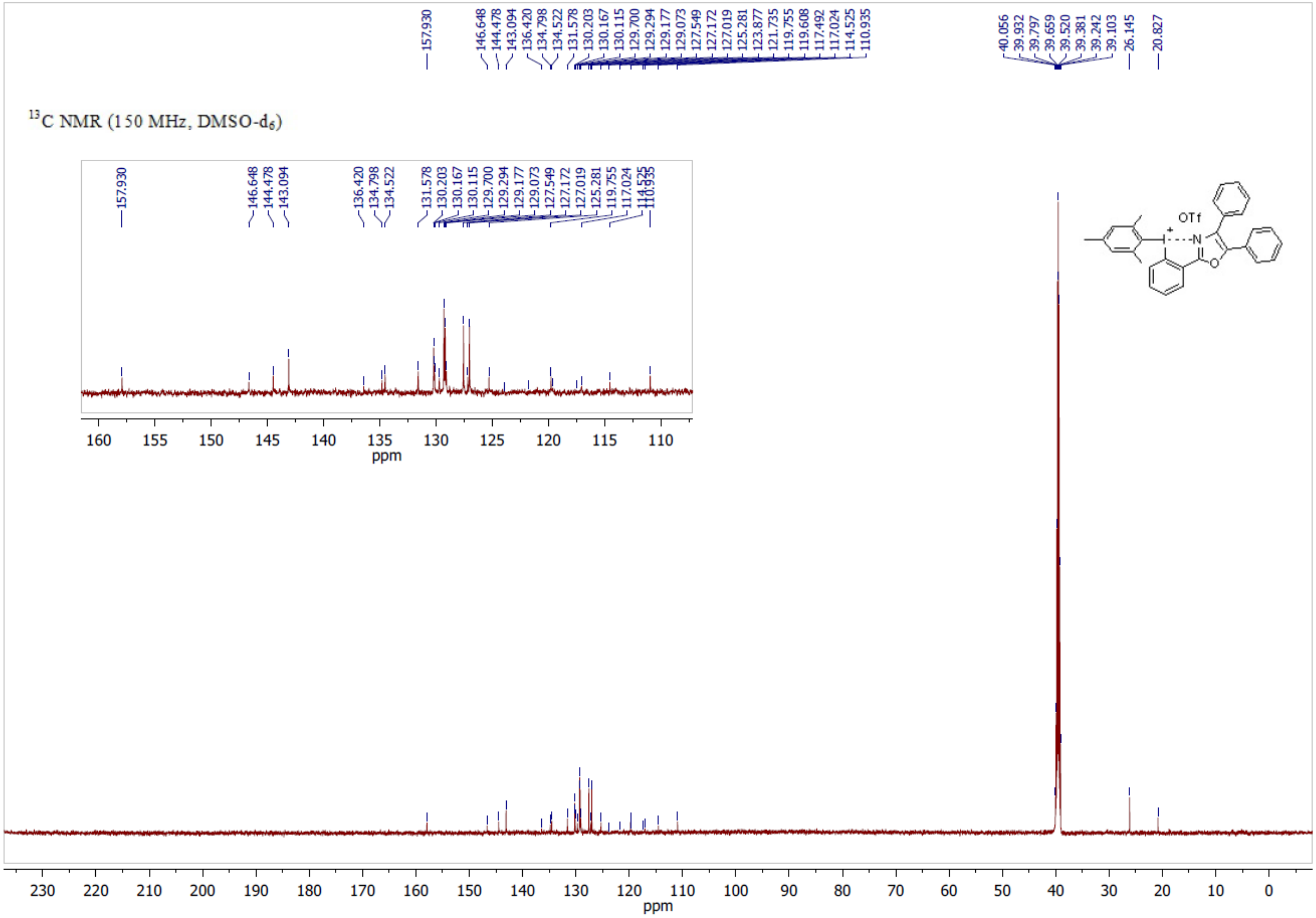

Figure S103: ^{13}C NMR spectra of **29** in d_6-DMSO.

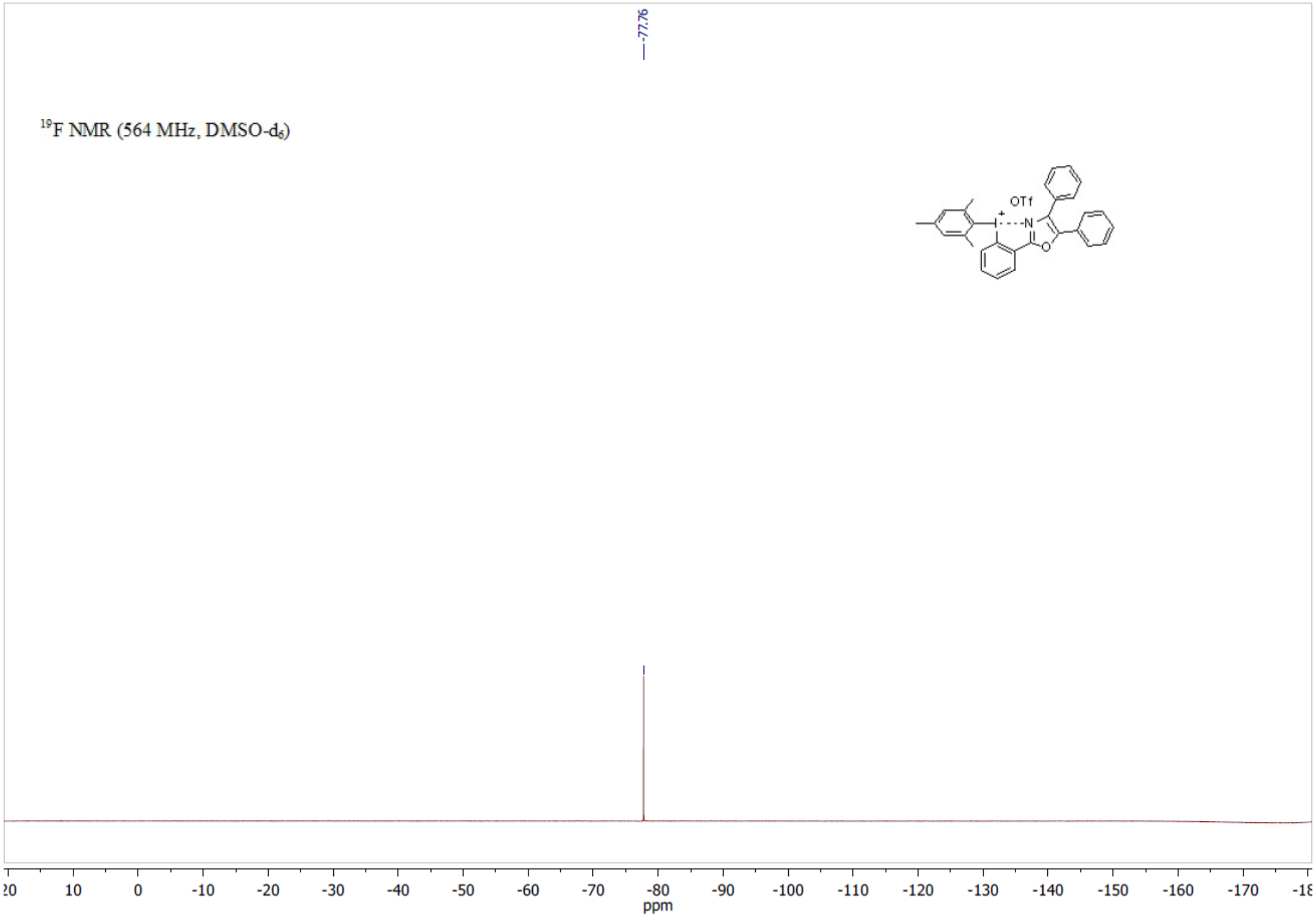

Figure S104: ^{19}F NMR spectra of **29** in d_6-DMSO.

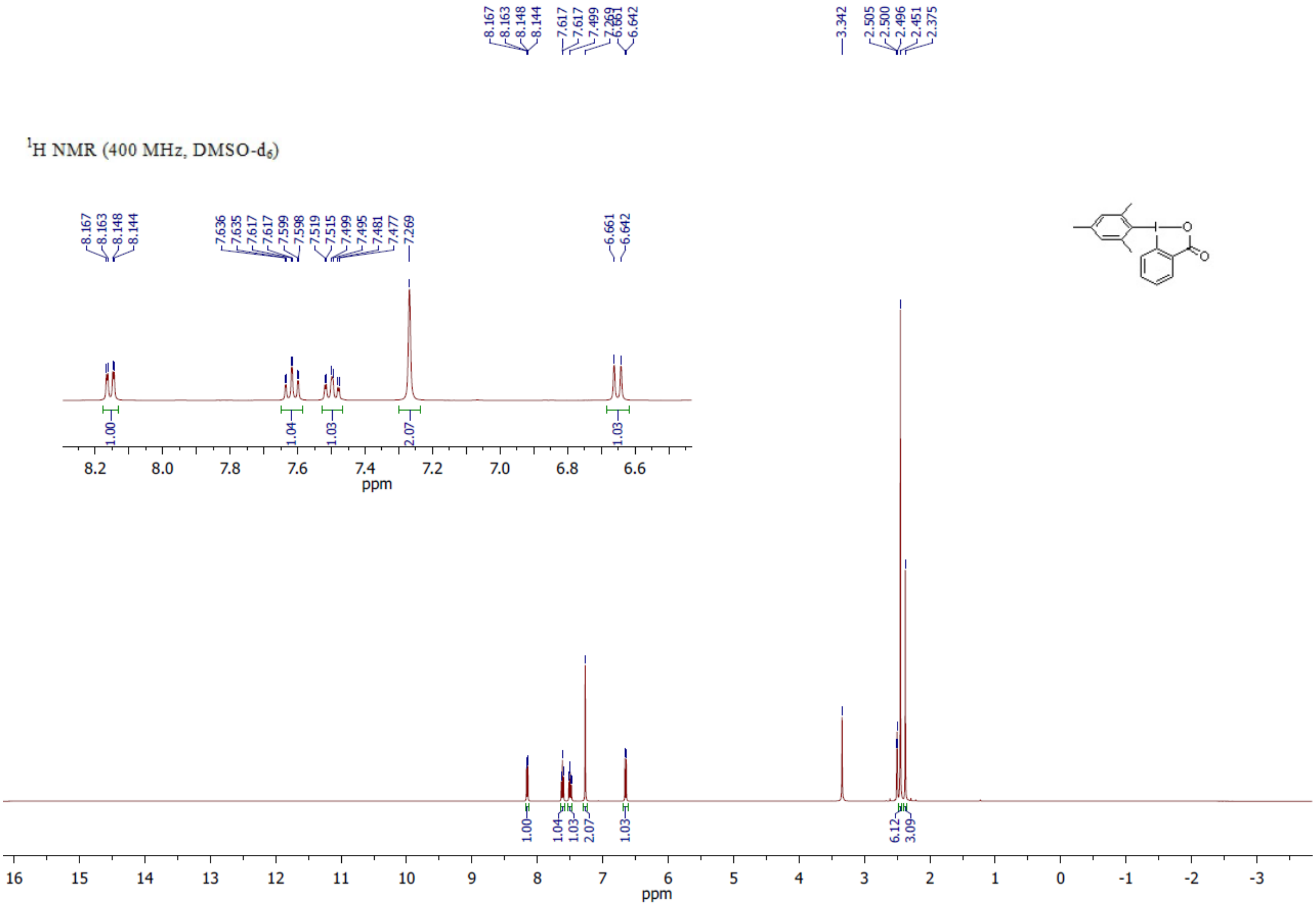

Figure S105: ^{1}H NMR spectra of **30** in d_6-DMSO.

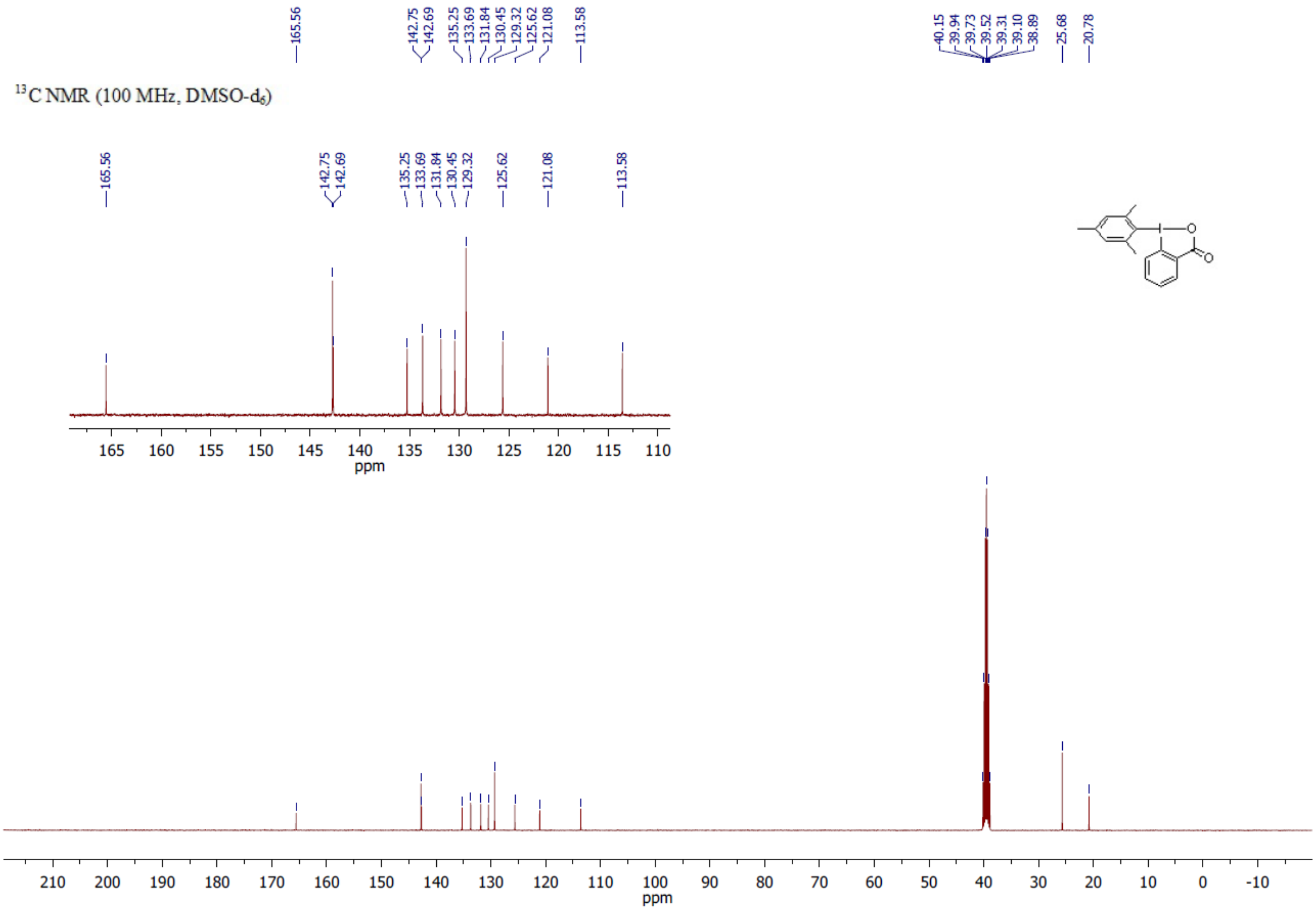

Figure S106: ^{13}C NMR spectra of **30** in d_6-DMSO.

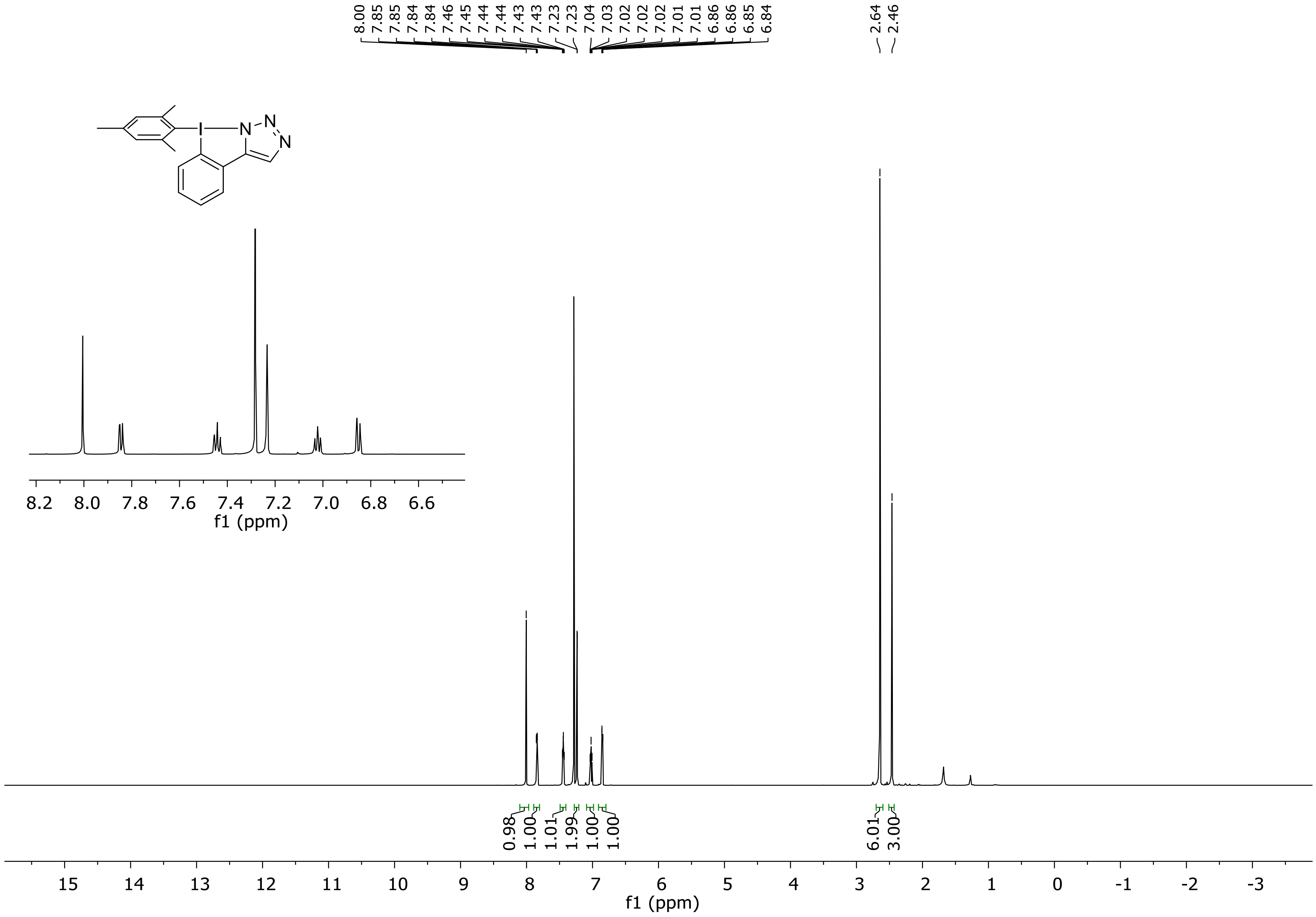

Figure S107: ^{1}H NMR spectra of **31** in $CDCl_3$.

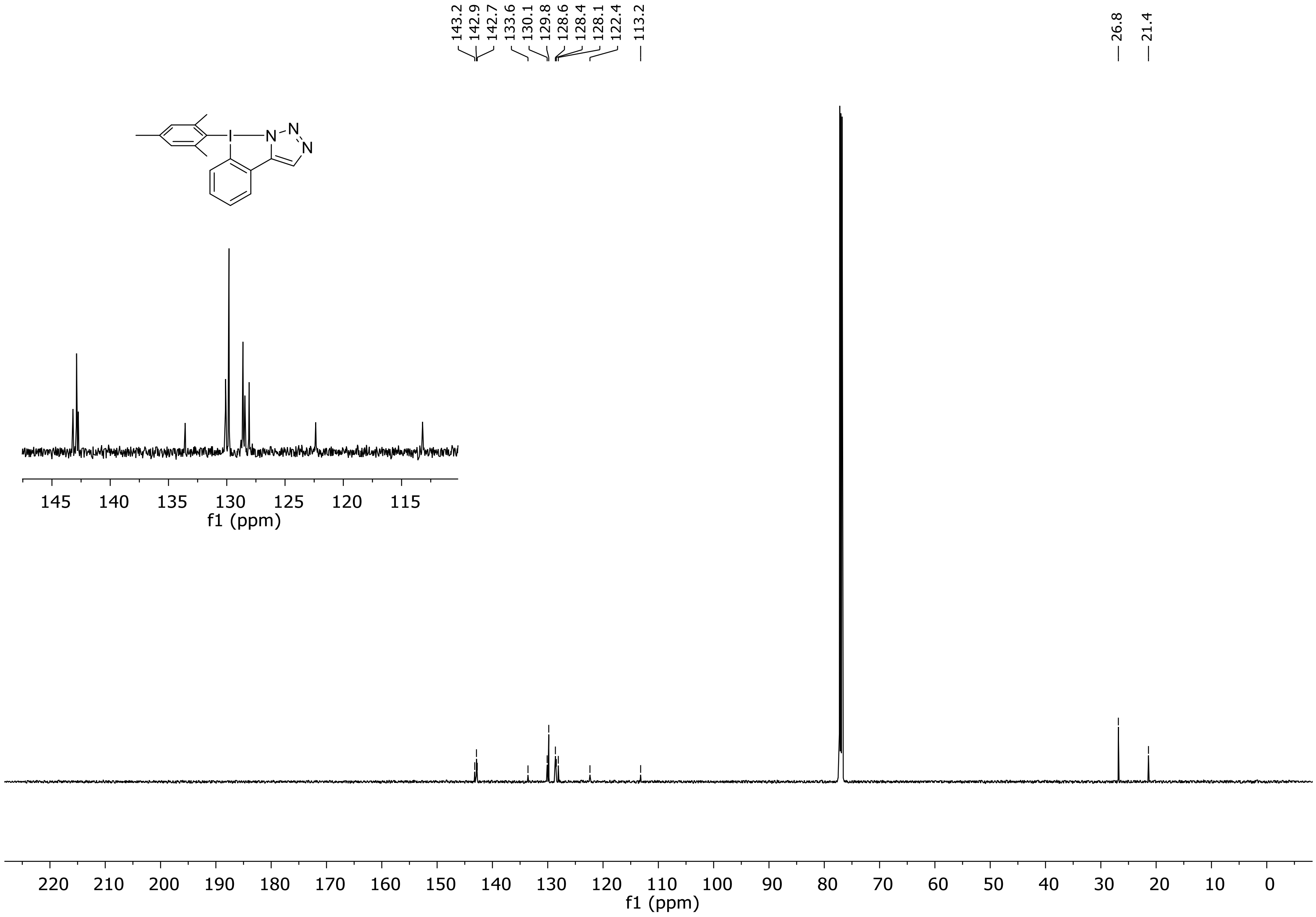

Figure S108: ^{13}C NMR spectra of **31** in $CDCl_3$.

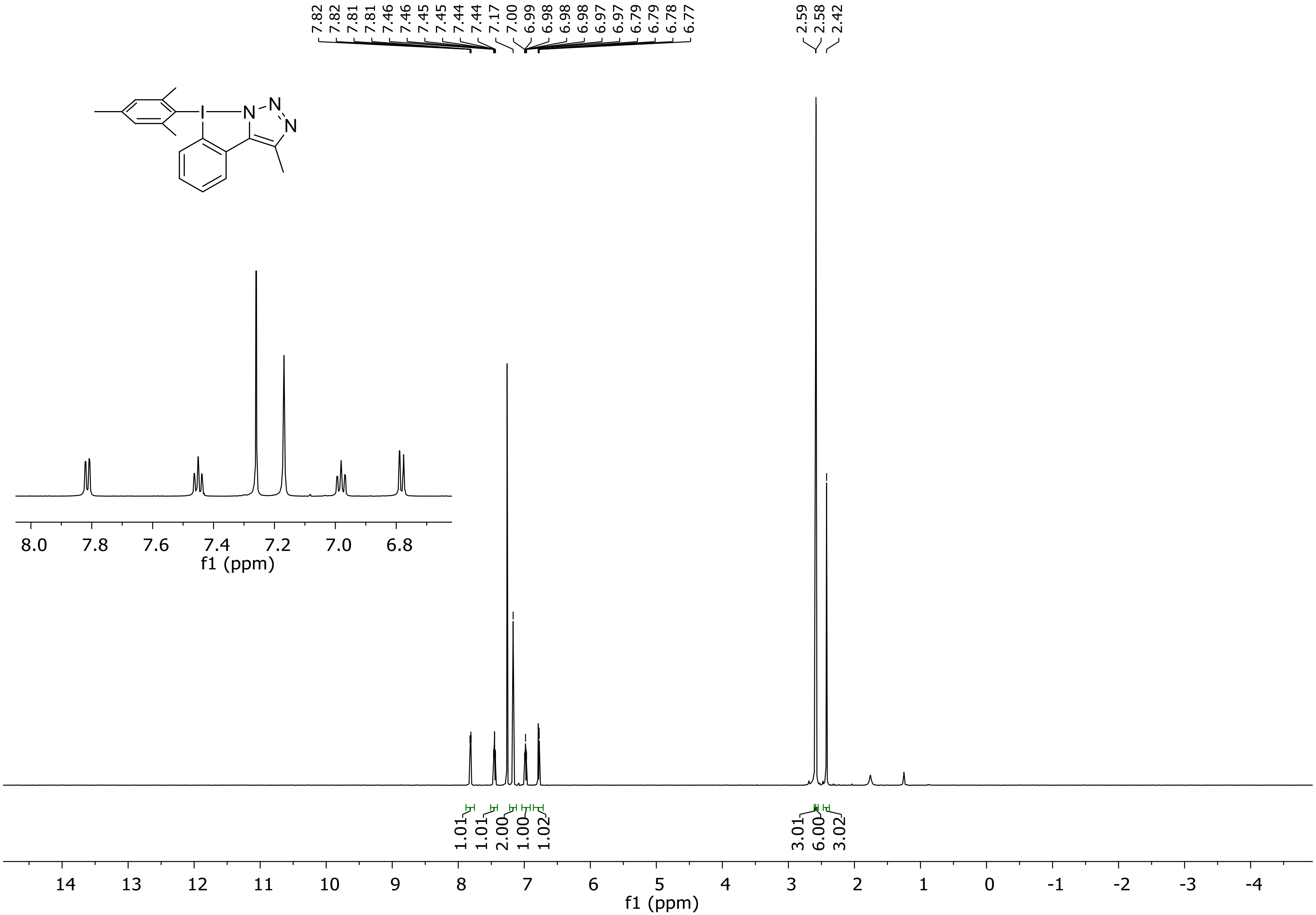

Figure S109: ^{1}H NMR spectra of **32** in $CDCl_3$.

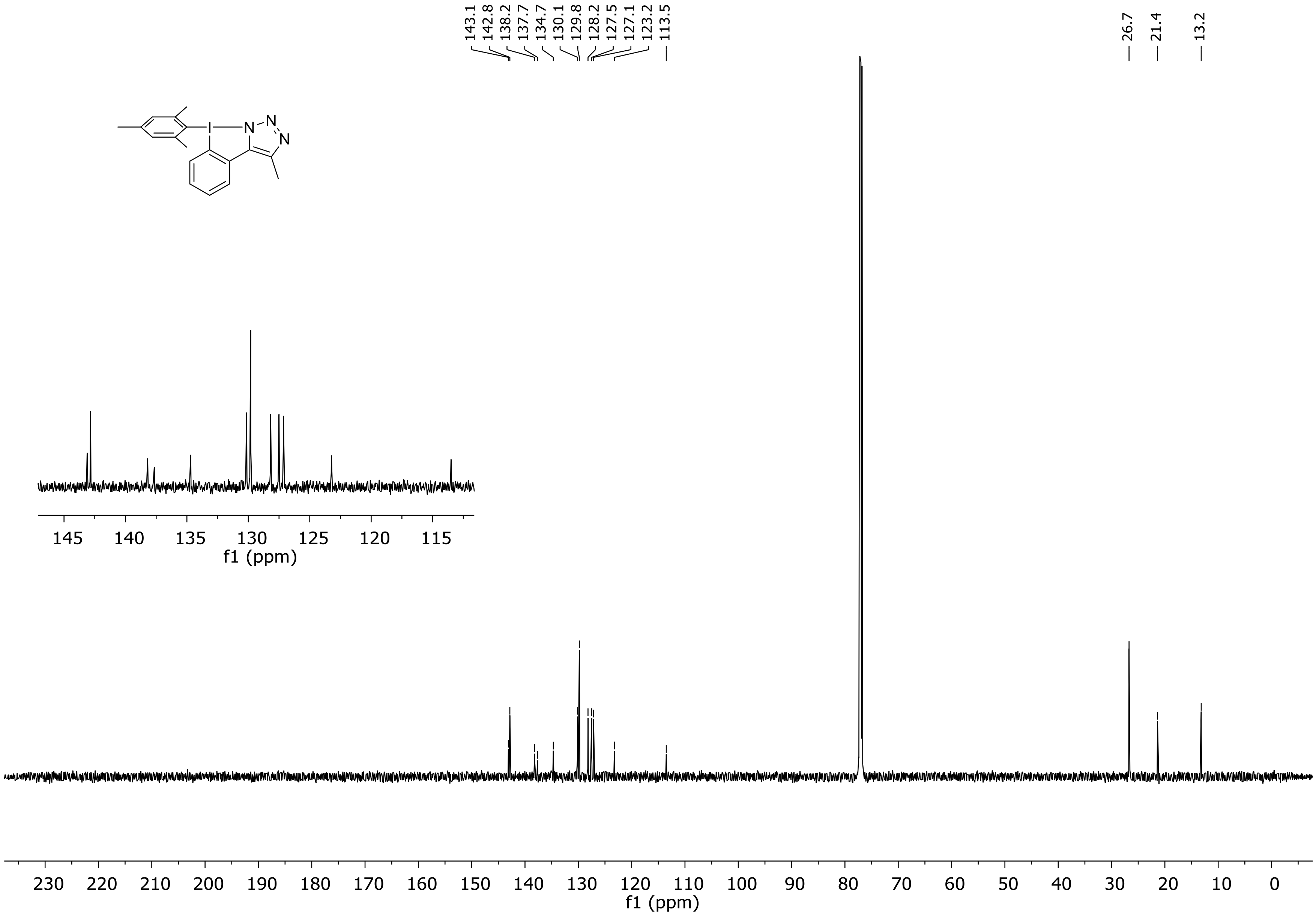

Figure S110: ^{13}C NMR spectra of **32** in $CDCl_3$.

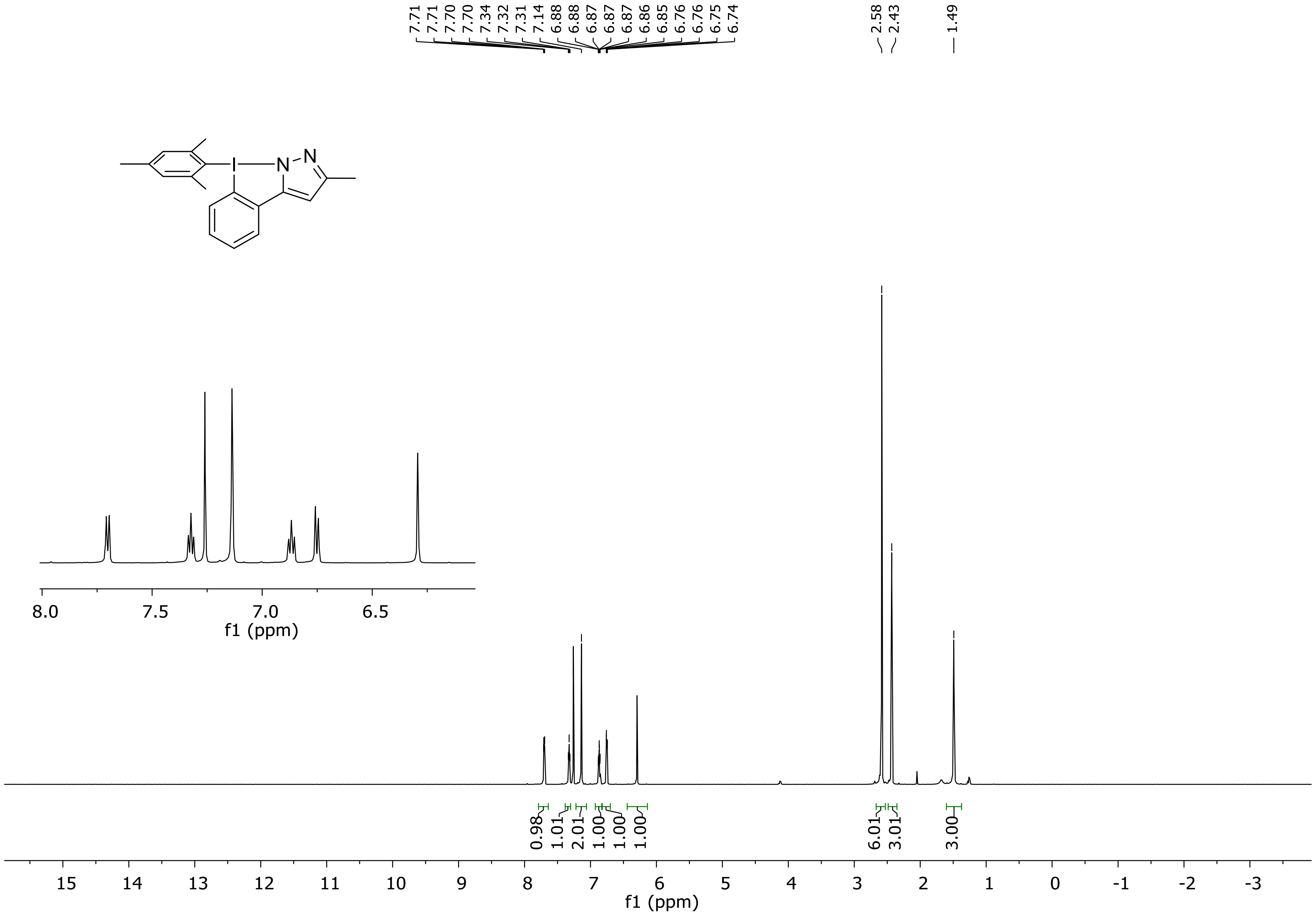

Figure S111: ^{1}H NMR spectra of **33** in $CDCl_3$.

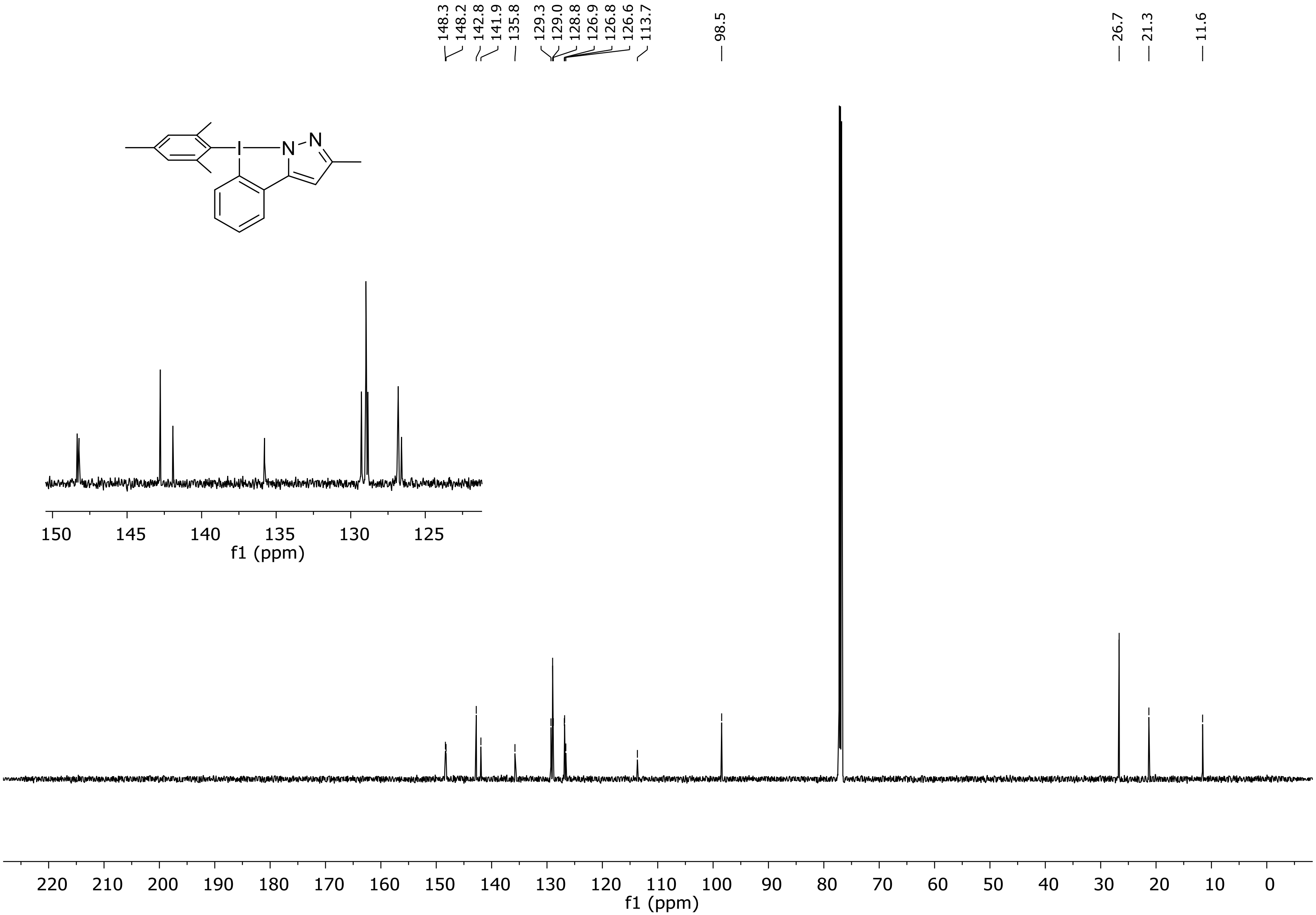

Figure S112: ^{13}C NMR spectra of **33** in $CDCl_3$.
